# Predicting success in Cu-catalyzed C–N coupling reactions using data science

**DOI:** 10.1126/sciadv.adn3478

**Published:** 2024-01-17

**Authors:** Mohammad H. Samha, Lucas J. Karas, David B. Vogt, Emmanuel C. Odogwu, Jennifer Elward, Jennifer M. Crawford, Janelle E. Steves, Matthew S. Sigman

**Affiliations:** ^1^ Department of Chemistry, University of Utah, 315 S. 1400 E., Salt Lake City, UT 84112, USA.; ^2^ Molecular Design, GlaxoSmithKline, 1250 S. Collegeville Rd., Collegeville, PA 19426, USA.; ^3^ Drug Substance Development, GlaxoSmithKline, 1250 S. Collegeville Rd., Collegeville, PA 19426, USA.

## Abstract

Data science is assuming a pivotal role in guiding reaction optimization and streamlining experimental workloads in the evolving landscape of synthetic chemistry. A discipline-wide goal is the development of workflows that integrate computational chemistry and data science tools with high-throughput experimentation as it provides experimentalists the ability to maximize success in expensive synthetic campaigns. Here, we report an end-to-end data-driven process to effectively predict how structural features of coupling partners and ligands affect Cu-catalyzed C–N coupling reactions. The established workflow underscores the limitations posed by substrates and ligands while also providing a systematic ligand prediction tool that uses probability to assess when a ligand will be successful. This platform is strategically designed to confront the intrinsic unpredictability frequently encountered in synthetic reaction deployment.

## Teaser

A data science workflow is developed to address unpredictability in Cu-catalyzed C–N coupling reactions.

## INTRODUCTION

Chemists have long sought experimental and computational platforms that can rapidly identify successful reactions. Recent advances in high-throughput experimentation (HTE) in combination with the emergence of machine learning (ML) algorithms within the field of chemistry provide opportunities to achieve this goal (*1*–*3*). When using ML in synthetic chemistry (*4*), the domain of applicability (i.e., the ability of the model to make accurate predictions) is defined by the experimental search space, which is constrained to a reasonable number of cost-effective experiments. For example, if the aim is to optimize a singular reaction toward a target molecule (e.g., total synthesis or process chemistry), then the goal is the identification of optimal reaction conditions often including a catalyst structure to achieve an excellent result (*5**,* *6*). Bayesian optimizers (*7*) or multivariate linear regression (*8*–*10*) have emerged as ML tools well suited for these tasks. In contrast, another central goal often encountered in synthetic chemistry is exploiting the generality of a reaction or applying the reaction to a wide range of substrates (*3*). For this task, the end user would want to have confidence that a particular reaction would work to give a reasonable yield under a prescribed set of conditions. This is a particularly important part of the drug discovery process wherein a medicinal chemist may be required to prepare libraries of compounds from a central core structure to gain structure-function relationships (*11**,* *12*). However, in most modern synthetic transformations, the scope of the reaction is not necessarily transferable to unseen targets, especially those with added structural complexity (*13*). This is an objective where ML tools have not yet seen widespread use.

**Fig. 1.**
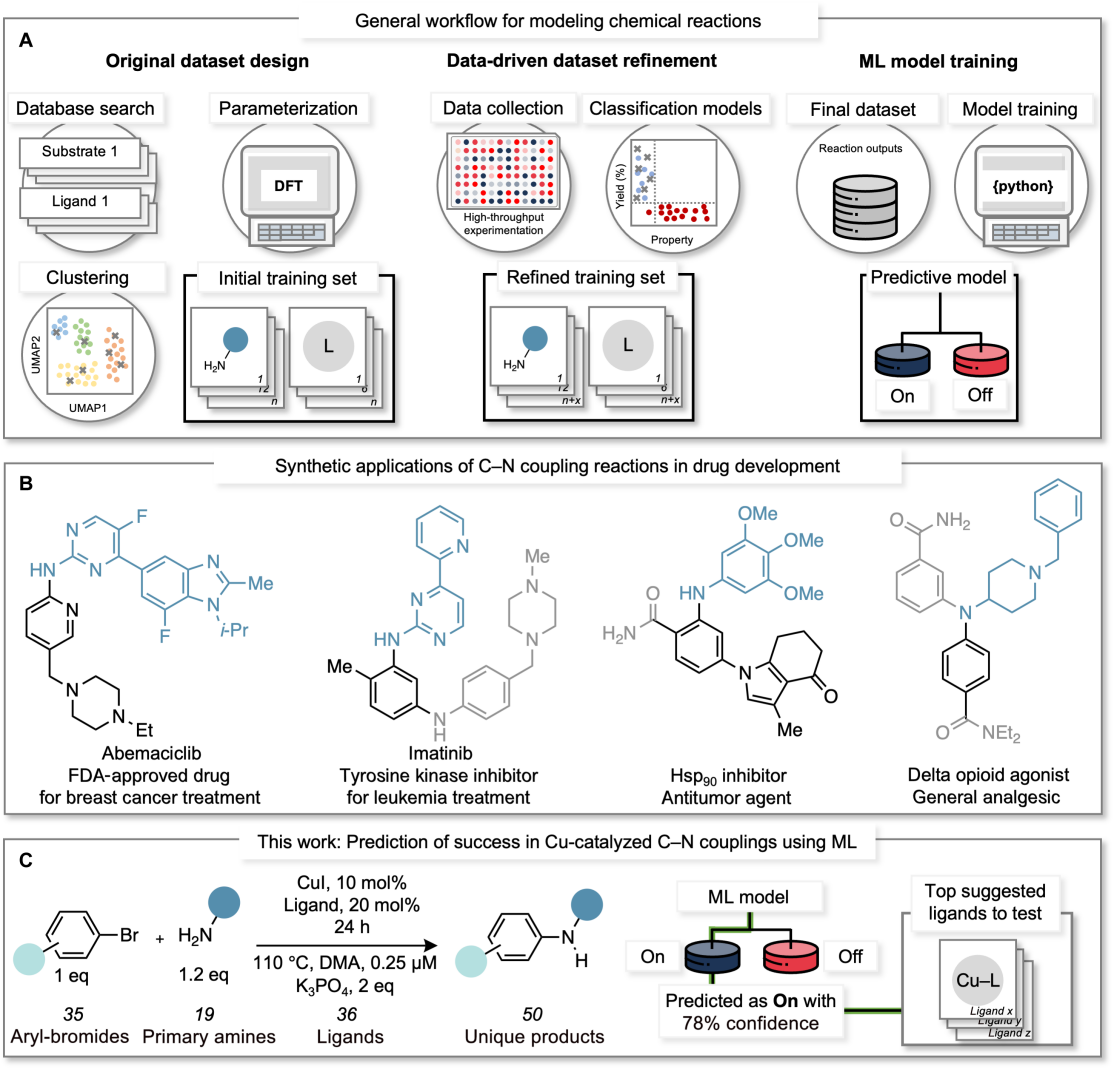
General modeling workflow, application of C–N couplings in drug development, and reaction conditions for this work. (**A**) General workflow for modeling chemical reactions: Dataset design initiated with a database query to create a library of commercially available substrates and ligands. These molecules are then parameterized using quantum-chemical calculations and clustered by similarity using dimensionality reduction and unsupervised ML techniques. Subsequently, molecules from each cluster are selected to form a diverse and representative substrate space for training the ML model. The training sets can be refined using active learning strategies that use classification models to identify substrate and ligand features responsible for the activity. These insights, in turn, guide the selection of additional substrates and/or ligands. The reaction output resulting from the final combination of substrates and ligands is then used to train a predictive ML model. (**B**) Synthetic applications of C–N cross-couplings in drug development. Black and blue atoms and bonds represent moieties originally from the aryl bromide and primary amine, respectively. Gray atoms and bonds indicate chemical transformations made after the C–N cross-coupling. (**C**) Reaction conditions used in this work and illustration of the tool’s utility, including confidence values on the prediction and top-suggested ligands for testing.

With this challenge in mind, we sought to develop an end-to-end workflow that would enable reaction developers and users alike to predict when a reaction would “work” as a function of key reaction conditions. The underlying driver for this effort was to provide a simple, robust, and interpretable tool for the practitioner, often a chemist, who can provide substantial domain expertise, but may be less familiar with the intricacies of ML. Here, we report a data science-driven workflow designed to navigate the intrinsic complexity of generalizing synthetic methods. We selected the Cu-catalyzed Ullmann C–N coupling reaction (*14*–*16*) as our case study due to its reported unpredictability and the limited understanding of its mechanism and substrate limitations (*17*–*19*). In addition, Ullmann couplings hold broad value in pharmaceutical synthesis, particularly in the late stages of drug development processes due to the lower cost and toxicity associated with the use of Cu (Fig. 1B) (*20*).

To address these challenges, we applied dataset design principles to effectively sample a diverse range of substrates and ligands, ensuring an adequate representation of the search space (*21*). HTE was deployed to produce reaction outputs, which were used in an active learning approach to reselect more efficient ligands (*22*). This iterative process led to an inclusive dataset, which, in turn, served as the foundation for training a supervised ML model. The result was the development of a predictive and interpretable classification model establishing connections between coupling partners and ligands that promote successful reactions (*23*). Last, the intrinsic limitations of the model were leveraged to inform a ligand selection platform that can uncover unexpected success for challenging substrates. This is highlighted by the ability of the workflow to identify effective ligands for reactions predicted to have a low probability of success (Fig. 1A).

## RESULTS

### Training set curation

To effectively sample the chemical space related to Ullmann couplings, we used a multi-stage data science process that was initiated by querying the ZINC20 database for structures containing aryl C–N bonds, as this database contains a large collection of accessible compounds (*24*). Curation was performed by filtering these examples according to their Log*P* (<4.0), molecular weight (<400 u), and compatibility with the reaction conditions (see Supplementary Materials for full details). This process yielded a dataset of ~2000 compounds, from which cheminformatics tools were used to fragment the compounds into a library of aryl bromides and primary amines. Last, we selected examples from these libraries according to their commercial availability and available spectral data. This resulted in ~400 unique aryl bromides and ~500 distinct primary amines, whose structures were submitted to quantum-chemical calculations to collect features that capture the electronic and steric properties of each substrate. To select representative examples in an unbiased fashion, the computed structural features were condensed by dimensionality reduction (Uniform Manifold Approximation and Projections, UMAP) to build a chemical space for aryl bromides and primary amines separately. This was followed by unsupervised learning to cluster similar substrates (*21*) (Ward clustering, see details in the Supplementary Materials). From each cluster, we selected molecules based on price and ease of access resulting in a diverse and representative scope of 24 aryl bromides and 12 primary amines for the study. In contrast, ligands were selected by prioritizing their commercial availability and their reported use in the Ullmann literature (*20*). This yielded a diverse collection of 24 ligands to be tested in the initial training set. We conducted the coupling reactions using an HTE setup for micromole-scale experimentation in 96-well plates. Considering the size of this initial search space, an intentional decision to have a constant set of reaction conditions (base, temperature, solvent, and concentrations) was made, although various conditions inspired by the Ullmann literature (*25*) were surveyed for operability and reproducibility before deployment (Fig. 1C; see Supplementary Materials for additional details).

To avoid a resource-prohibitive scenario resulting from the full factorial combination of all substrates, which would warrant the development of 288 quantitative assays, we coupled aryl bromides and primary amines from various clusters within their chemical spaces to initially obtain 37 distinct products (further details available in the Supplementary Materials). All assayed products underwent two distinct control experiments that included a ligand-free condition where no ligand was introduced and a reaction without any ligand or copper source. These measures were essential to ascertain that the observed variances in reactivity were attributed to catalysis rather than S_N_Ar or other side reactions (*26*). Nine of the unique products (**P001** to **P009**) failed the control tests and, therefore, were removed from the training dataset (see details in the Supplementary Materials). For the reactions that showed evidence of catalysis, we set a threshold of two catalytic turnovers (20% yield) to discern reactivity as either “on” or “off.” The choice of two catalytic turnovers was grounded in practical relevance within the realms of reaction/drug development protocols—two catalytic turnovers could provide sufficient material in drug/library synthesis campaigns while also being a plausible lead to initiate optimization campaigns in process chemistry (*27*).

**Fig. 2.**
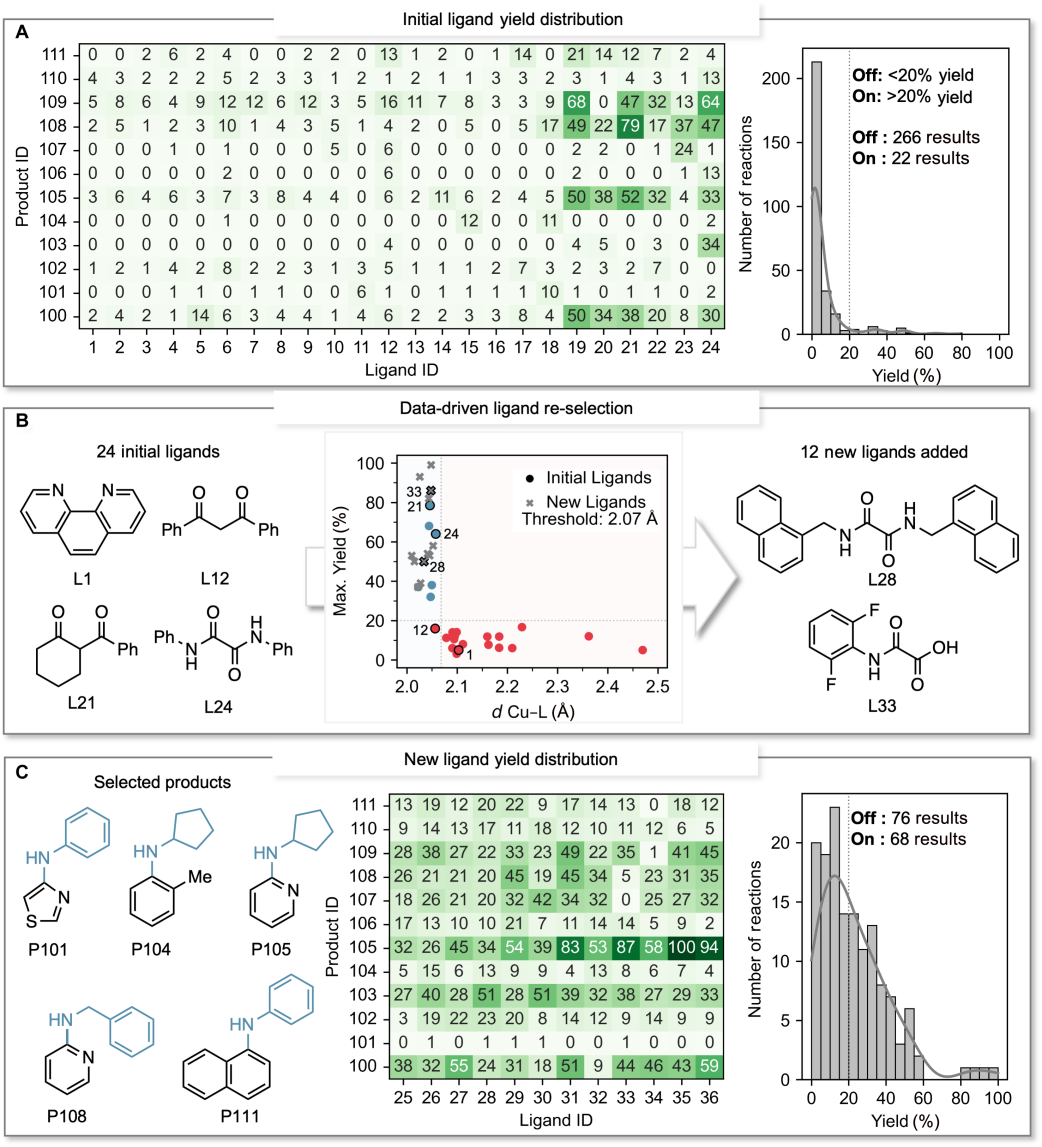
Iterative refinement of the training set. (**A**) Initial training set yields and yield distribution revealing a notable bias towards off reactivity (yield, <20%). (**B**) Analysis of the initial training set with the Cu–L computed interaction distance (*d*). The analysis achieves an accuracy of 0.96 and an F1 score of 0.95. The prediction accuracy for the newly selected ligands is 100%. The dotted lines indicate the 20% yield threshold for on:off reactivity and the 2.07-Å threshold for the computed *d*, signifying the importance of *d* in reaction yields. Red and blue dots correspond to ligands that either fall below or exceed the 20% yield threshold in the ligand training set, respectively. Newly chosen ligands are marked with gray crosses. Selected ligands are depicted with dark contours. (**C**) Representation of selected products. Newly selected ligand yields and yield distribution demonstrating a balanced on:off ratio.

Among the initial products (**P100** to **P111**) assayed, it was apparent that a substantial bias existed in the yield distribution with a ~12:1 ratio favoring off reactivity over on reactivity (Fig. 2A). The initial products were tested against 24 ligands (**L1** to **L24**). Notably, we found that ligands **L19** to **L24** were the only ligands to produce yields exceeding 20%. This observation led us to hypothesize that these ligands must share common structural features that enhance their activity in Ullmann couplings under the conditions used. Therefore, we submitted this dataset to a classification algorithm (single-node decision tree) to identify whether they share structural features (*23*) (see computational details in the Supplementary Materials). This algorithm searches for ligand structural descriptors that provide the most accurate distinction between active and inactive ligands. The findings revealed that the ligands could be effectively binned into two groups according to the computed Cu–ligand (Cu–L) interaction distances (*d*), with high classification accuracy (see Supplementary Materials for all model metrics). Ligands that produced yields exceeding 20% all displayed computed interaction distances measuring less than 2.07 Å (with only **L12** being misclassified; Fig. 2B). A shorter Cu–L distance is indicative of a stronger interaction between the Cu center and the ligand. This observation may connect to the stability of the Cu–L complex in solution and explain its efficacy in engaging in the catalytic cycle (*28*). Note that all ligands with optimal computed distance are anionic in nature, which could potentially play a pivotal role in the effectiveness of these ligands in Ullmann couplings, as recently suggested by Buchwald and Hartwig (*18**,* *19**,* *28*). To leverage this discovery, we adopted an active learning strategy to resample other commercial ligands, identifying 12 examples (**L25** to **L36**) that were predicted to be catalytically active by the classification model (i.e., ligands displaying optimal computed distances). Experimentally, all the newly selected ligands were found to surpass 20% maximum yield, validating the classification model. The newly introduced ligands produced a better-balanced yield distribution across the 12 initial products, ~1:1 ratio between instances of on and off reactivity (Fig. 2C). These results showcase the efficacy of active learning to enhance dataset representation (*29*–*31*).

Using ligands **L19** to **L36**, we evaluated the remaining 16 products defined in the training set. These ligands encompass three classes of ligands commonly used in Ullmann couplings: diketone (*32**,* *33*), oxalamide (*34**,* *35*), and anilino(oxo)acetic acid ligands (*20*). Ultimately, our comprehensive training set encompassed a total of 720 reactions (excluding controls), achieving a satisfactory ratio of 3.5:1 between off and on reactivity (see all yields and dataset distribution in the Supplementary Materials).

### Predictive model training and external validation

**Fig. 3.**
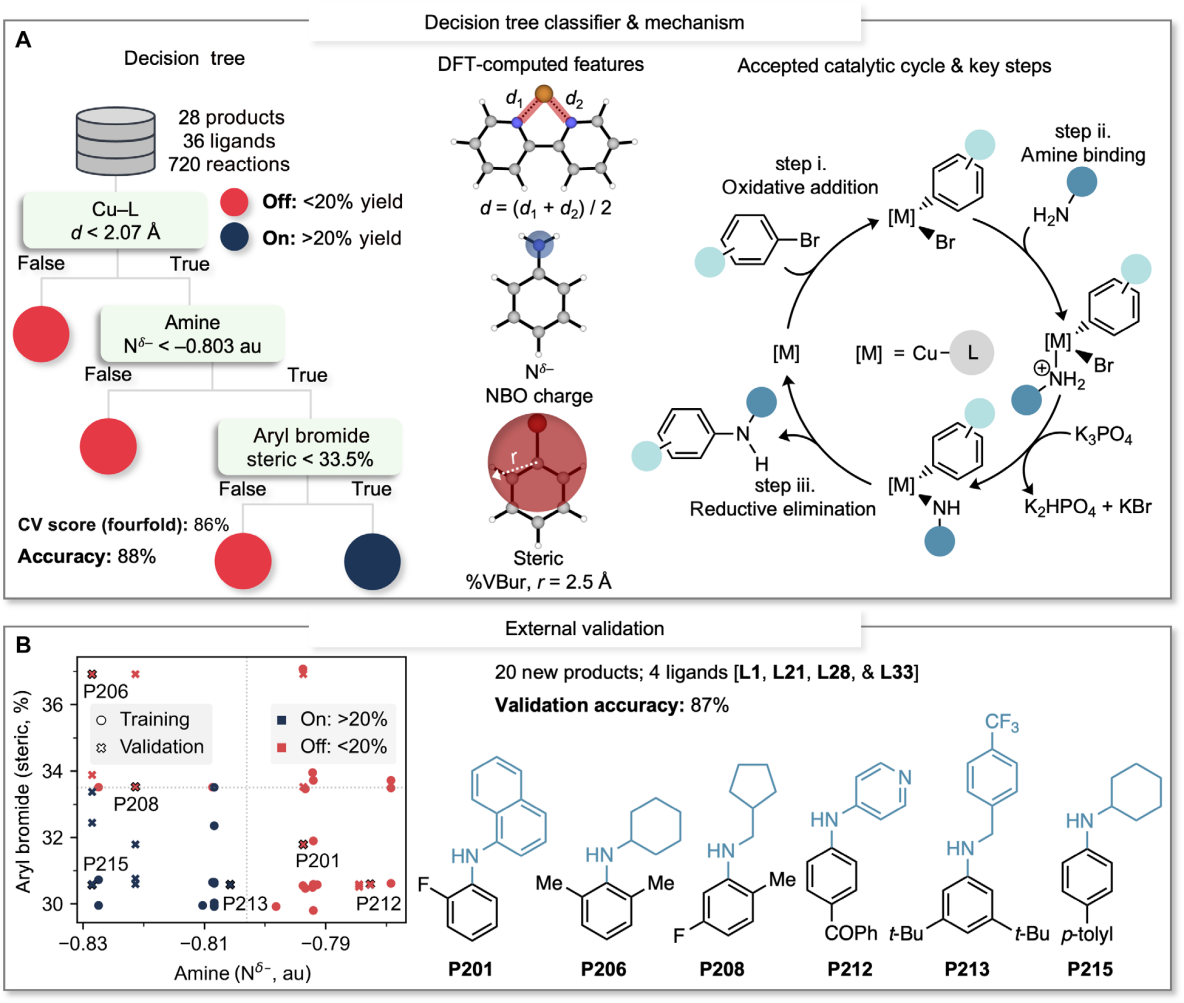
Decision tree classification model and external validation process. (**A**) Decision tree classification model and its accuracy in classifying ligand-substrate combinations for Ullmann C–N couplings into either on or off reactivity; graphic representation of the molecular features present at the decision nodes; and proposed catalytic cycle highlighting key mechanistic steps of Ullmann C–N couplings. (**B**) External validation process of the decision tree classification model using substrates that were not seen during the training phase. The classification results are indicated by the colors blue (representing on-classification) and red (representing off-classification). The training products are depicted as circles, while the validation products are denoted by crosses. Selected validation products are highlighted with dark contours.

As the next step, our goal was to create an ML model characterized by simplicity and robust interpretability resulting in the exploration of decision tree classifiers. These algorithms operate by recursively and procedurally dividing the structural feature space within a training dataset (*36*–*38*). We trained a decision tree using the complete array of descriptors for substrates and ligands, using a random training/test split ratio of 75:25 (for further details, see Supplementary Materials). Our findings revealed that the most basic decision tree, consisting of only three nodes (one corresponding to each reaction component, as described below) is able to classify combinations of aryl bromides, primary amines, and ligands into on or off reactivity with high accuracy (Fig. 3A, left). Specifically, the model achieved 88% accuracy on the test set, along with an average accuracy of 86% across a fourfold cross-validation procedure using the entire dataset.

## DISCUSSION

The trained model is interpretable as the computed molecular features (depicted in Fig. 3A, center) associated with the decision tree nodes exhibit a connection to the stability of Cu species within a possible catalytic cycle (Fig. 3A, right) (*28*). Notably, as recently reported by Hartwig *et al*. (*18*, *19*), different mechanisms can be operative, changing as a function of structural features on the ligand, which also is likely influenced by the nature of the coupling partners. The initial node is reminiscent of the ligand re-selection step above; it splits the dataset according to the computed Cu–L interaction distance (*d*)—reactions using ligands with computed *d* exceeding 2.07 Å are classified as off, yielding <20%. As previously discussed, the computed *d* can be interpreted as the stability of the Cu–L complex in the reaction medium, its capacity to enter the catalytic cycle, or, potentially, its ability to undergo oxidative addition. The subsequent node uses the computed nitrogen atom natural bonding orbital charge of the primary amine substrate (N^δ−^). Reactions involving primary amines with low nucleophilicity [N^δ−^ > −0.803 atomic unit (au)] are identified as unfavorable according to the trained model. This finding aligns with step (ii) in the catalytic cycle, indicating that the success of Ullmann couplings hinges on the primary amines’ capability to bind to the Cu center. The final node uses the computed buried volume (%VBur, 2.5-Å radius) of the aryl bromide. Reactions with aryl bromides exhibiting high steric hindrance (%VBur, >33.5%) are classified as producing yields below 20%. This molecular feature is a measure of the steric environment on the ipso-carbon and could potentially connect to several steps in the catalytic cycle, such as impeding the oxidative addition, amine binding, or reductive elimination in step (iii).

Our model highlights the intricate interplay between the electronic features of the primary amine and the steric hindrance of the aryl bromide, both of which play pivotal roles in determining the success of Ullmann C–N couplings. Moreover, these features offer insights into the substrate chemical space of Ullmann coupling reactions. This is visually represented in the chemical space plot presented in Fig. 3B. It is evident from the plot that our training set (depicted as circles) spans all four quadrants of the decision tree chemical space, with good representation in each. However, there were regions within the plot that remained unexplored, specifically those involving combinations of sterically hindered aryl bromides and nucleophilic primary amines. The chemical space plot was thus used to guide the external validation of the classification model. For validation purposes, we used four in-sample ligands representing all classes of ligands used in this work (neutral ligands: **L1**, diketones: **L21**, oxalamides: **L28**, and anilino(oxo)acetic acids: **L33**) and out-of-sample substrates that were not encountered during the training phase. This involved introducing a set of 12 aryl bromides and six primary amines, all out of sample, culminating in the creation of 20 distinct products for external validation (depicted as crosses in Fig. 3B). This comprehensive approach was designed to span a wide range of the defined chemical space by targeting underrepresented regions with examples of aryl bromides with substantial steric hindrance and amines of varying nucleophilicity. The resulting accuracy of this validation phase was 87%, which is virtually identical to the accuracy achieved in the test set during the training phase (88%) and highlights the robustness and reliability of the classification model.

### Refined workflow incorporating confidence values and ligand suggestion

**Fig. 4.**
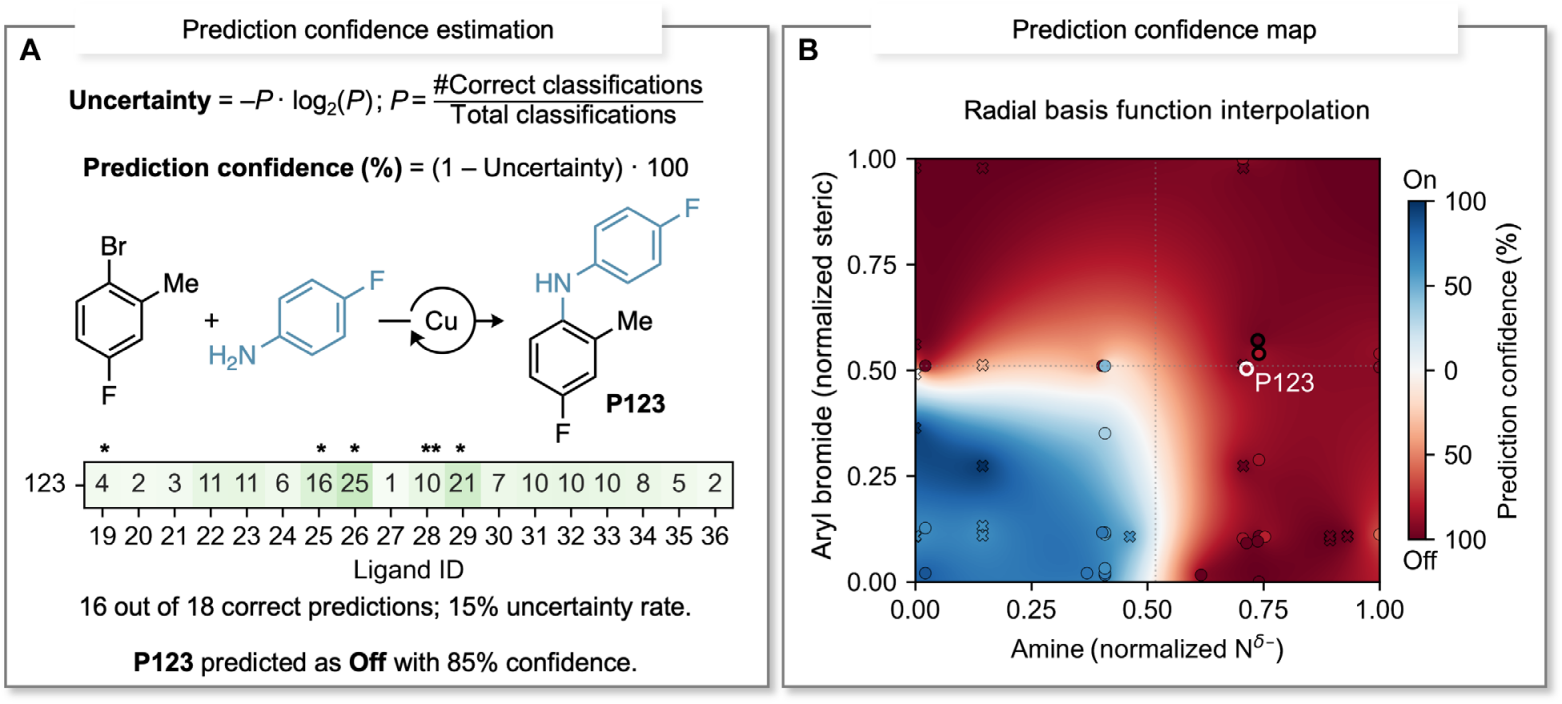
Estimation of confidence in the prediction value and confidence map. (**A**) Example of prediction confidence estimation using product **P123**: Out of the 18 ligands tested, 16 ligands yielded results as predicted (<20%), resulting in an estimated prediction confidence of 85%. (**B**) Prediction confidence map constructed using estimated confidence values for each product within this study (encompassing both training and validation sets) and the radial basis function interpolation method. Areas shaded in darker blue indicate higher confidence in predicting “on” reactivity, whereas darker red denotes greater confidence in predicting “off” reactivity. The targeted product **P123** is highlighted with a white contour, while its two nearest neighbors are highlighted with black contours. The recommended ligands are marked (*) and **L28** appears in both neighbors (**).

While the accuracy achieved with the simple classification model is high, note that misclassifications occur at an overall rate of ~13%. To minimize classification errors, we opted to treat each product as an independent prediction—this was possible because the products were evaluated against multiple ligands, resulting in a multidimensional dataset. This approach enabled us to acquire insights into the prediction uncertainty (*39*) associated with each individual product, offering a broader understanding of Ullmann C–N coupling reactions. For example, consider product **P123** in Fig. 4A, which is anticipated to yield less than 20% according to the classification model. Among the 18 tested ligands, two generated yields exceeding 20% for this specific product. Therefore, the resulting uncertainty, calculated as information entropy (*40*), is estimated at 15% for product **P123** (see Supplementary Materials for details). The prediction confidence can subsequently be determined as 85% for **P123**, which is the complementary value of the uncertainty. We then iterated through this process for each product, resulting in a heatmap of prediction confidence. The gaps between known values (products) were interpolated using a radial basis function, which is particularly well-suited for scattered data and does not necessitate a mesh grid (*41*). This methodology yields a comprehensive map where predictions can be made for any unknown products to the model, and the corresponding confidence levels can then be inferred (Fig. 4B). Through leveraging the error in the initial classification model, we expose the capability of finding potential success even with combinations of substrates considered challenging for Ullmann couplings. For example, combinations that are initially predicted to yield below 20% might still produce the desired results if the correct ligand is used.

Therefore, it was crucial to develop a method for identifying the ligands with the highest likelihood of producing high yields for a given set of coupling partners. This involved determining the ligands that corroborate the prediction when a combination of substrates is anticipated to yield above 20%, as well as identifying the ligands that challenge the prediction when combinations are expected to yield below 20%. We hypothesized that the optimal ligand could be found by searching for the best-performing ligands for substrate combinations with similar properties (determined by the substrate’s structural features found in the decision tree). To accomplish this, we executed a search to identify the three top-performing ligands for each of the two nearest neighbors of a given product. For instance, **P123** was predicted to produce yields below 20% with an 85% confidence in the prediction—this level of confidence indicates that one or two ligands could yield results exceeding 20%. By identifying its two nearest neighbors (**P111** and **P125**, highlighted with bold black contours in Fig. 4B) and the corresponding top-three ligand performers for each of these neighbors (**L19**, **L25**, **L26**, **L28**, and **L29**, with **L28** present in both neighbors), the tool can effectively narrow down the ligand search space for this Ullmann coupling to just five [these ligands are highlighted with asterisk symbols (*) in Fig. 4A]. Remarkably, this ligand suggestion includes the two ligands that exhibited yields above 20% for product **P123** (**L26** and **L29**)—our analysis indicates that in 93% of cases, at least one top-performing ligand for a given product is also a top performer among the nearest neighbors (see details in the Supplementary Materials). This example serves as an illustration of the potential applications of the classification workflow. Simply put, using this approach highlights that all the information embedded into the decision tree is useful—even when the overall prediction is “incorrect,” the predictive workflow can be harnessed to select the most effective ligands.

### Synthetic applications

**Fig. 5.**
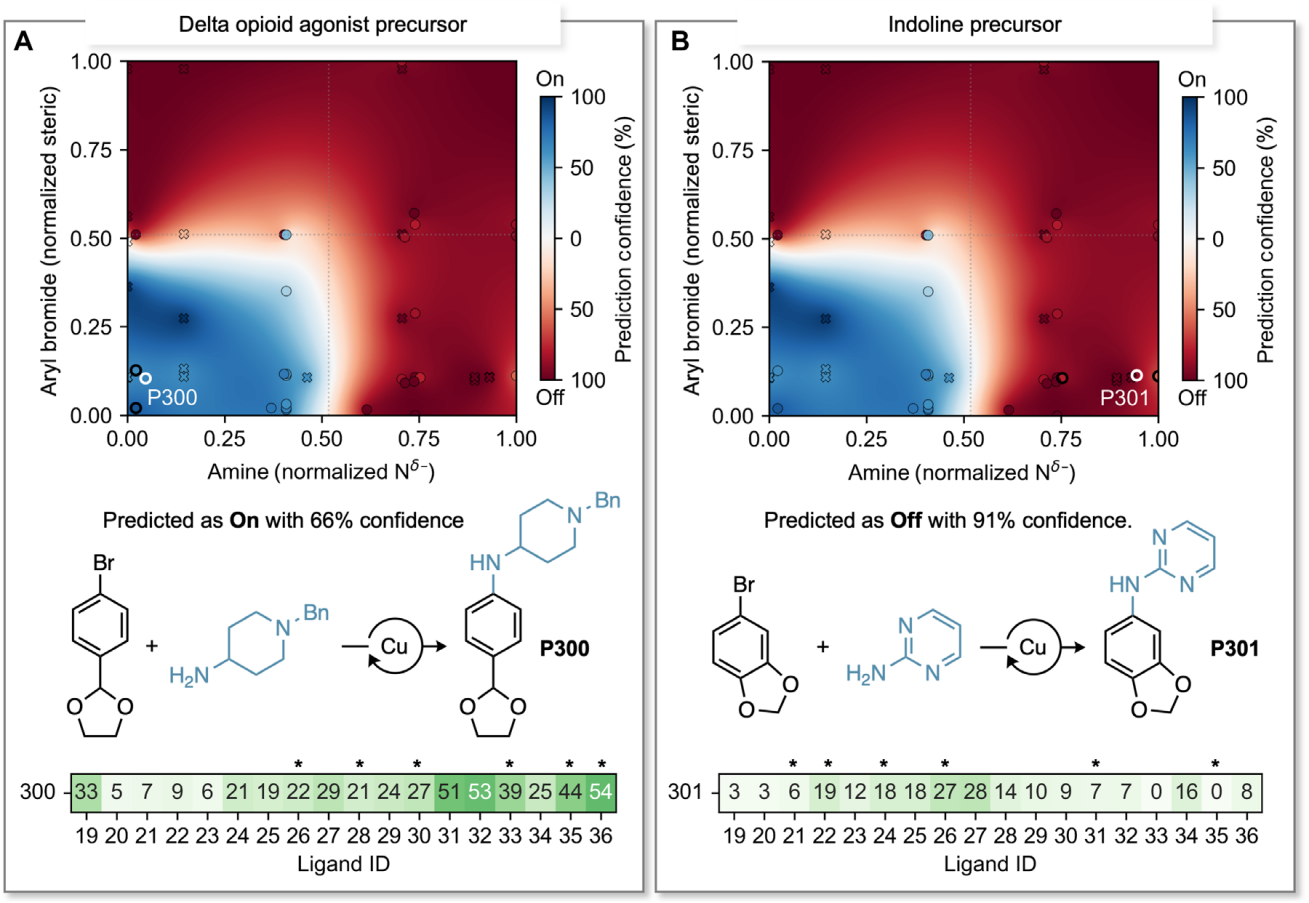
Synthetic applications. (**A**) Prediction of a favorable and (**B**) challenging combination of substrates toward synthetically relevant products through Ullmann C–N couplings. The target compounds are indicated by white outlines, while the two nearest neighbors are highlighted with bold black outlines. The recommended ligands are marked (*).

Having established the workflow, we sought to additionally validate the workflow using two molecules of synthetic interest: **P300**, a delta opioid agonist precursor (*42**,* *43*) (Fig. 5A), and **P301**, an indoline precursor (*44*–*46*) (Fig. 5B). **P300** can be accessed through the coupling of an unhindered aryl bromide with a highly nucleophilic primary amine. This combination is predicted to yield above 20%, with a confidence level of 66% (equivalent to ~12 out of 18 ligands expected to yield the predicted results). The experimental outcomes demonstrated an alignment with the predictions, with precisely 12 out of the 18 tested ligands yielding the anticipated results surpassing 20%. Upon examining the top-performing ligands from the two nearest neighbors, we found that all six suggested ligands (**L26**, **L28**, **L30, L33**, **L35**, and **L36**, indicated by the asterisk symbol in Fig. 5A) yielded the anticipated results, with **L36** being the top-performer for **P300**. This underscores the effectiveness of the workflow and highlights the feasibility of streamlining synthetic efforts to the targeted selection of only six recommended ligands based on the two nearest neighbors in chemical space.

As the next example, **P301** represents a notable challenge, as the primary amine is poorly nucleophilic. This coupling is predicted to result in yields below 20%, with a confidence level of 91%. Nevertheless, this suggests the possibility that one or two ligands could yield results surpassing 20%. This example highlights the value of incorporating confidence into reaction predictions, as without it, our knowledge would be limited to the prediction of low yields for this coupling reaction. The notion that one or two ligands could potentially succeed offers a promising outlook. In fact, among the 18 tested ligands, two produced yields above 20%, thus validating both our prediction and the associated confidence levels. The recommended ligands (**L21**, **L22**, **L24**, **L26**, **L31**, and **L35**, indicated by the asterisk symbol in Fig. 5B) using the two nearest neighbors’ tactic overlooked one of the two ligands that achieved yields surpassing 20% (**L27**). Nonetheless, the method accurately forecasted the virtually top-performing ligand, **L26**. Moreover, it suggested two ligands **L22** and **L24** that produced yields close to the 20% threshold. This approach demonstrates considerable potential as an initial step in optimization campaigns. These two examples demonstrate the utility of the proposed workflow in mitigating risks and enhancing the likelihood of success in Ullmann C–N coupling reactions.

Through the implementation of this predictive workflow, a platform that streamlines reaction development has been formulated to address the inherent unpredictability associated with Ullmann C–N coupling reactions. This strategy provides a framework to advance traditional reaction design methods, enabling the strategic implementation of traditionally unreliable chemical methods. The combination of reaction exploratory assays and ML algorithms presented in this work can considerably enhance the potential for widespread adoption in the advancement of both pharmaceutical and academic interests.

## MATERIALS AND METHODS

Our Ullmann C–N coupling prediction tool named CopperMap is available at https://github.com/SigmanGroup/CopperMap. This tool is designed for predicting yield outcomes in Ullmann couplings and recommending optimal ligands. The detailed workflow used to produce the reported results can be found online at https://github.com/SigmanGroup/ullmann_project/. All materials and methods are available in the Supplementary Materials.

## Supplementary Materials

### This PDF file includes:

Supplementary Text Figs. S1 to S18 Tables S1 to S4 Legends for data S1 and S2 References
