# Supplementary material for: Predicting success in Cu-catalyzed C–N coupling reactions using data science
Source: Sci Adv. 2024 Jan 17;10(3):eadn3478. doi: 10.1126/sciadv.adn3478 (PMC10793951; doi:10.1126/sciadv.adn3478)
Supplement: Supplementary file 1 — Supplementary Text Figs. S1 to S18 Tables S1 to S4 Legends for data S1 and S2 References [file sciadv.adn3478_sm.pdf]

Science Advances
AAAS

Supplementary Materials for

Predicting success in Cu-catalyzed C–N coupling reactions using data science

Mohammad H. Samha *et al.*

Corresponding author: Jennifer M. Crawford, jennifer.crawford2@bms.com; Janelle E. Steves, janelle.e.steves@gsk.com; Matthew S. Sigman, matt.sigman@utah.edu

Sci. Adv. **10**, eadn3478 (2024)
DOI: 10.1126/sciadv.adn3478

The PDF file includes:

Supplementary Text
Figs. S1 to S18
Tables S1 to S4
Legends for data S1 and S2
References

Other Supplementary Material for this manuscript includes the following:

Data S1 and S2

Ligand and substrate library acquisition

Substrate library

We leveraged the ZINC20 [47] database, accessed on August 12, 2022, to compile a library of aryl bromides and primary amines that represent the Ullmann C–N coupling chemical space. We initiated by querying the database using the following criteria:

- LogP (octanol water partition coefficient) < 4.0.
- Molecular weight < 400 Da.

These criteria were selected to exclude complex and large molecules that might not be soluble in organic solvents. Subsequently, we employed RDKit and SMART strings to refine the list of compounds, selectively keeping those compounds containing aryl C–N bonds by partitioning the initial list using the following five filters (code available: https://github.com/SigmanGroup/Commercial_Search/):

- Include compounds containing nitrogen atoms bonded to a hydrogen atom and two aromatic groups (with the bonding atom limited to carbon atoms only).
- Include compounds containing nitrogen atoms bonded to a hydrogen atom, one aromatic rings (with the bonding atom limited to carbon atoms only), and one aliphatic group.
- Include compounds containing nitrogen atoms bonded to three aromatic rings (with the bonding atom limited to carbon atoms only).
- Include compounds containing nitrogen atoms within a ring (where one bonded atom is an aromatic carbon and the other can be any atom), along with a bond to one aromatic group (with the bonding atom limited to carbon atoms only).
- Include compounds containing nitrogen atoms bonded to two aliphatic groups, and one aromatic ring (with the bonding atom being any atom).

This process resulted in ca. 355,000 compounds containing aryl C–N bonds. We then refined the selection by excluding compounds with incompatible functional groups for Ullmann C–N couplings. Compounds with the following functional group were excluded: carboxylic, phosphoric, and boronic acids; organosilicons; azides; alkyl and aryl alcohols; compounds with multiple amines; alkyl and aryl halides; aldehydes; oximes; sulfonyl; and amides.

The resultant list of compounds was subsequently refined for commercial availability of both the C–N product and the fragments, resulting in ca. 2700 compounds containing aryl C–N bonds. Finally, we fragmented the C–N products into the corresponding aryl-bromides and primary amines. Following the removal of duplicates, we obtained libraries of 421 aryl-bromides and 515 primary amines (complete list provided as "computed_data.xlsx; arbr_library and amine_library sheets").

Ligand library

The initial selection of ligands was derived from the Reaxys database, where we searched for commonly used ligands in the Ullmann literature. The database was accessed on August 10, 2022, with the following search criteria:

- Reactions catalyzed by CuI metal source.
- Reactions coming from published articles or patents.
- Single set reactions only.
- All products are available for purchase and not proprietary.

The search yielded ca. 700 reactions, which were subsequently analyzed for ligand selection. We employed RDKit to extract the ligand examples from these reactions, resulting in a compilation of ~100 unique ligands. These ligands were further refined according to commercial availability from one of the following suppliers:

- Sigma-Aldrich
- Combi-Blocks
- Oakwood Chemical
- Ambeed
- TCI Chemicals
- Fisher Scientific

We identified 32 commercially available ligands. Of these, 24 were selected based on their immediate availability and cost for our initial screening, Figure S1.

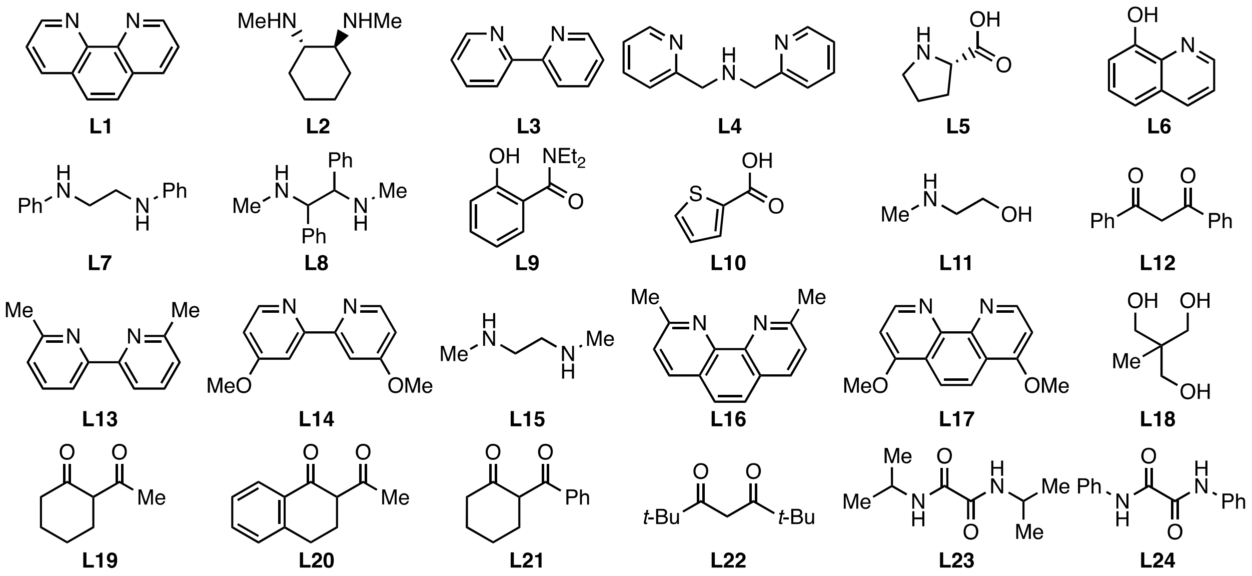

Figure S1. Initial ligands selected.

Conformational search and DFT geometry optimization

Molecular mechanics conformational search employing Schrödinger MacroModel (Release 2023-2)[48] and the OPLS4 force field was performed on each aryl bromide, primary amine, and ligand in the libraries. The conformational searches were performed in gas phase with a maximum of 10,000 interactions and convergence threshold of 0.001 au. The conformer window was restricted to 5.02 kcal/mol of the lowest energy conformer, excluding mirror-image conformers. For compounds that generated over 50 conformers during the conformational search, we clustered the conformers based on their atomic root mean square deviation to the minimum Kelly penalty value and selected the centroid of each cluster to represent the conformational ensemble—all ligands and substrates employed in the training set had less than 50 conformers and, therefore, were not clustered.

Density Functional Theory (DFT) geometry optimizations for all conformers were then performed at the ωB97X-D/def2-SVP level employing Gaussian16 version C.01.[49] Frequency calculations confirmed the first-order nature of each conformer. Energies and molecular features were computed at the ωB97X-D/def2-TZVP level. Natural bond orbital (NBO) analysis was performed using NBO 7.0.

The Cu–L complexes were computed as Cu(I) state, where the Cu center is bound to both the ligand and an iodine atom. The complexes **L1**, **L2**, **L3**, **L4**, **L7**, **L8**, **L13**, **L14**, **L15**, **L16**, **L17**, **L18** were computed as neutral (charge = 0). The complexes **L5**, **L6**, **L9**, **L10**, **L11**, **L12**, **L19**, **L20**, **L21**, and **L22** were computed as monoanionic (charge = –1). The complexes **L23**–**L36** were computed as di-anionic (charge = –2).

Parametrization and molecular descriptors collection

Global and atom-specific (Figure S2) molecular descriptors were collected from Gaussian output files or computed with the Morfeus python packages.[50] For each molecular descriptor, the minimum, maximum, and Boltzmann-weighted average values of the descriptor as well as the descriptor value for lowest energy conformer in the conformational ensemble were collected. Boltzmann-weighted properties were calculated using the Gibbs corrected energies computed using GoodVibes.[51] In total, we collected a total of 190 molecular descriptors for the ligands, 180 molecular descriptors for the aryl bromides, and 204 molecular descriptors for the primary amines. A complete list of these descriptors is available as supporting information as “computed_data.xlsx.”.

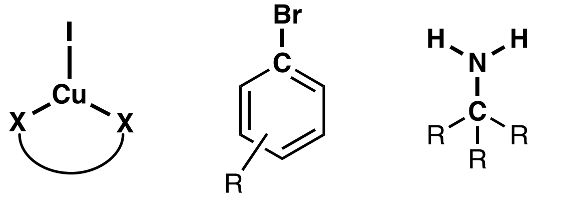

Figure S2: Atoms used for molecular parameter acquisition.

Global descriptors

- HOMO and LUMO energies
- Chemical potential, hardness, and electrophilicity index
- Dipole moment
- Molecular volume
- Polarizability
- Solvent accessible surface area

Atom-specific descriptors

<u>Ligands</u>

- Cu–L distance (*d*), average between the two Cu…X distances
- Bite angle
- Natural Population Analysis charges for Cu. [keyword: POP=NBO7]
- Hirshfeld charges for Cu [keyword: POP=Hirshfeld]
- NMR shielding for Cu [keyword: NMR]
- %VBur for Cu from 2 to 5 Å at 0.5 Å steps, computed with Morfeus
- Sterimol B1, B5, and L values (I–Cu), computed with Morfeus
- Buried Sterimol B1, B5, and L values (I–Cu) within 5.0 Å, computed with Morfeus

<u>Aryl bromides</u>

- C…Br distance (*d*)
- Bite angle
- Natural Population Analysis charges for C and Br [keyword: POP=NBO7]
- Hirshfeld charges for C and Br [keyword: POP=Hirshfeld]
- NMR shielding for C and Br [keyword: NMR]
- %Vbur for C and Br from 2 to 5 Å at 0.5 Å steps, computed with Morfeus
- Sterimol B1, B5, and L values (Br–C), computed with Morfeus
- Buried Sterimol B1, B5, and L values (Br–C) within 5.0 Å, computed with Morfeus

<u>Primary amines</u>

- N…H and C…N distances
- Natural Population Analysis charges for C, N, and H [keyword: POP=NBO7]
- Hirshfeld charges for C, N, and H [keyword: POP=Hirshfeld]
- NMR shielding for C, N, and H [keyword: NMR]
- %Vbur for C and N from 2 to 4 Å at 0.5 Å steps, computed with Morfeus
- Sterimol B1, B5, and L values (C…N), computed with Morfeus
- Buried Sterimol B1, B5, and L values (C…N) within 5.0 Å, computed with Morfeus

Clustering the substrate libraries

In order to select representative examples of aryl bromides and primary amines from the built libraries, we employed dimensionality reduction and clustering techniques. These methods allowed us to group the molecules based on their structural similarities and facilitated the selection of a diverse selection of substrates.

We followed the procedure published by the Doyle lab (code available in ref 21), employing Uniform Manifold Approximation and Projection (UMAP) to reduce data dimensionality and the Ward hierarchal clustering method. We only utilized molecular descriptors from the lowest energy conformer of each substrate to create the UMAPs. These molecular descriptors were subjected to normalization and further refinement, which involved filtering out features with low variance (< 0.025) and highly correlated pairs (> 0.85). Our results indicated that employing 5 UMAPs projections and 6 clusters yielded optimal outcome for aryl bromides, whereas 2 UMAPs projections and 7 clusters proved most effective for primary amines, according to the Silhouette score analysis, Figure S3. The Silhouette score quantifies the degree of similarity among molecules within the same cluster compared to their dissimilarity with molecules in other clusters. Consequently, a higher silhouette score indicates more effective classification of molecules into distinct clusters.

Following this step, we selected aryl bromides and primary amines from the individual clusters to create a diverse training set of aryl bromides and primary amines. We selected 24 aryl bromides representing all six clusters, and 12 primary amines that represent 5 out of the 7 total clusters. The two clusters of primary amines not covered are cluster 3 and 4. Cluster 3 was excluded from the selection of primary amines because it is composed of amines bound to sulfonamides, which would not be suitable for our study. Cluster 4 was omitted since it mainly consisted of chiral amines that were commercially inaccessible.

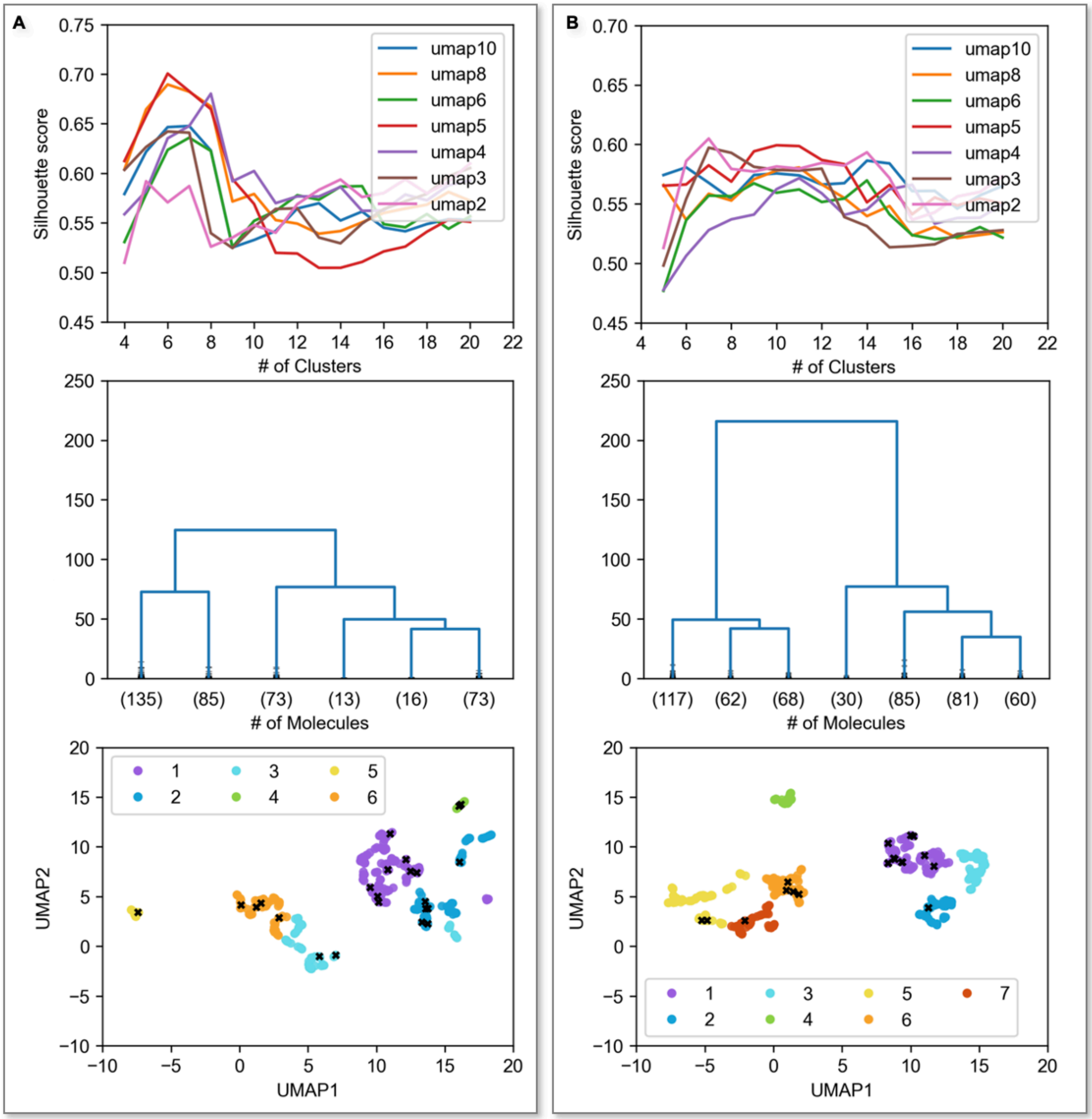

Figure S3. Silhouette score analysis, Ward clustering dendrograms, and UMAP projections, revealing the chemical space of (**A**) aryl bromides and (**B**) primary amines. The selected substrates for the training set are marked with crosses in the projections.

Product selection for training set

The training set was formulated by analyzing the clusters of aryl bromides and primary amines from the chemical space maps above. Representative molecules from each cluster were selected and paired combinatorially. From this process, 37 unique products were synthesized and prepared for HTE analysis, covering a total of 17 different combinations of clusters. Products in the training set are found below (Figures S4–S8).

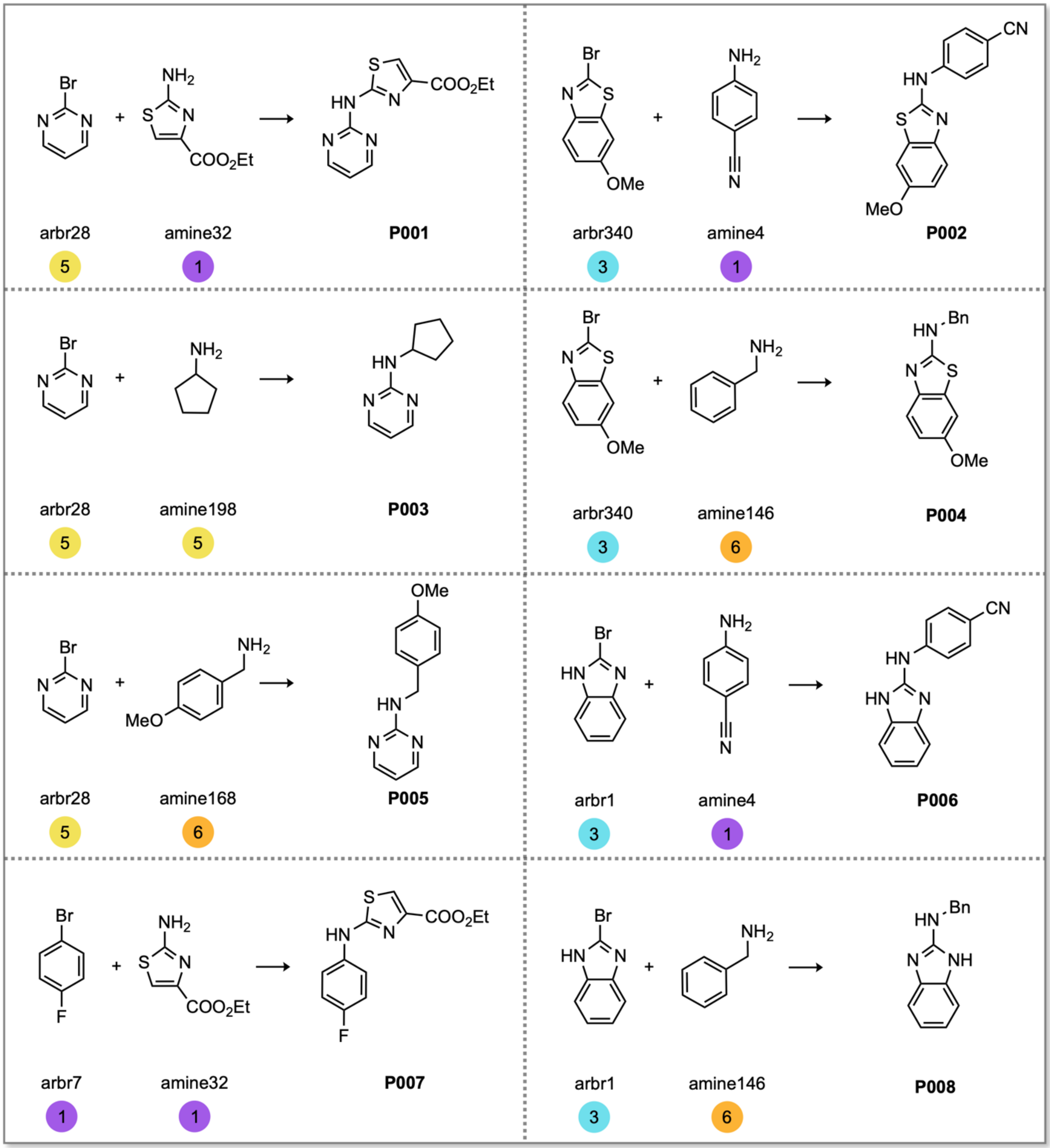

Figure S4. Training set of C–N products (**P001-P008**).

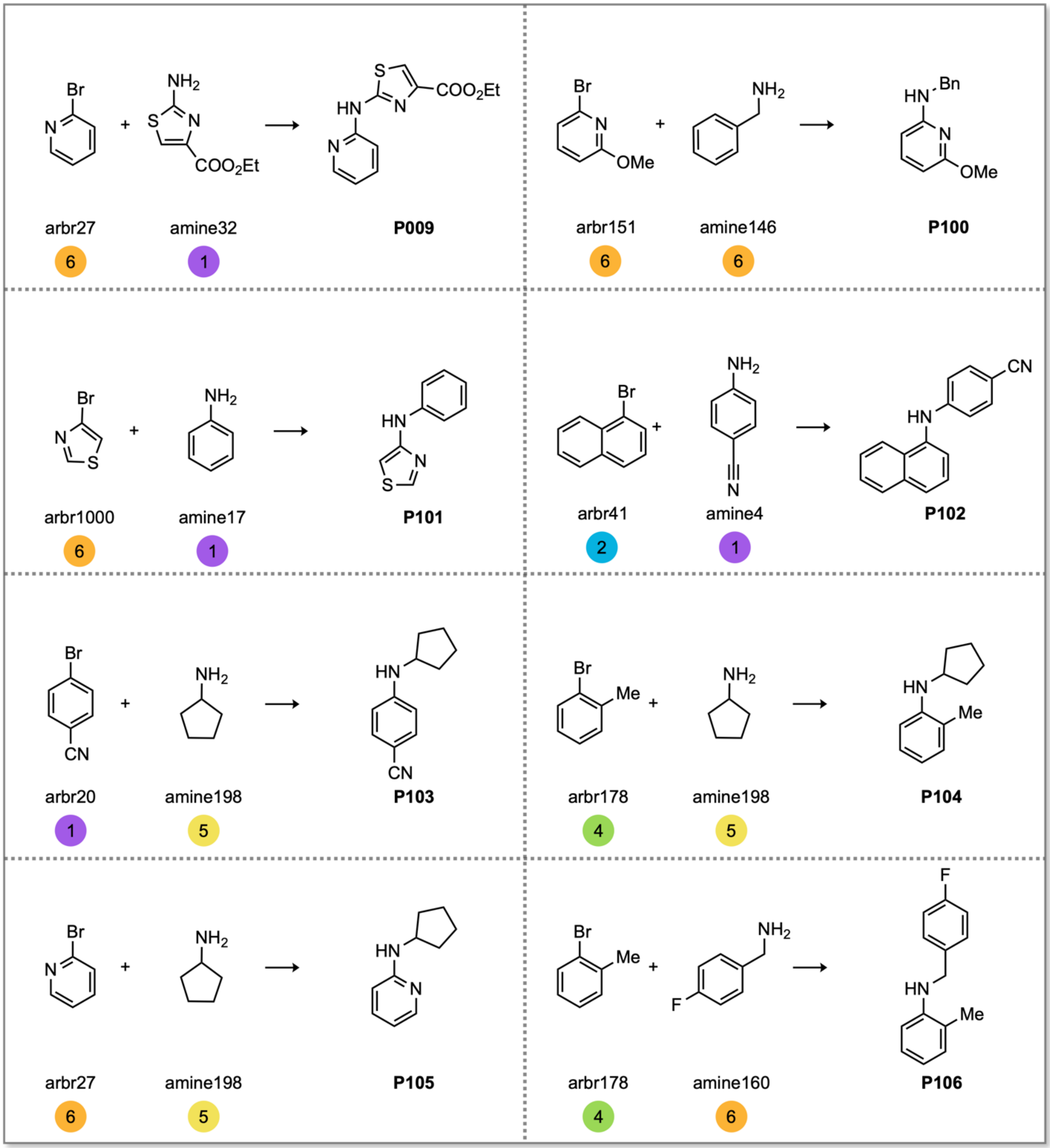

Figure S5. Training set of C–N products (**P009-P106**).

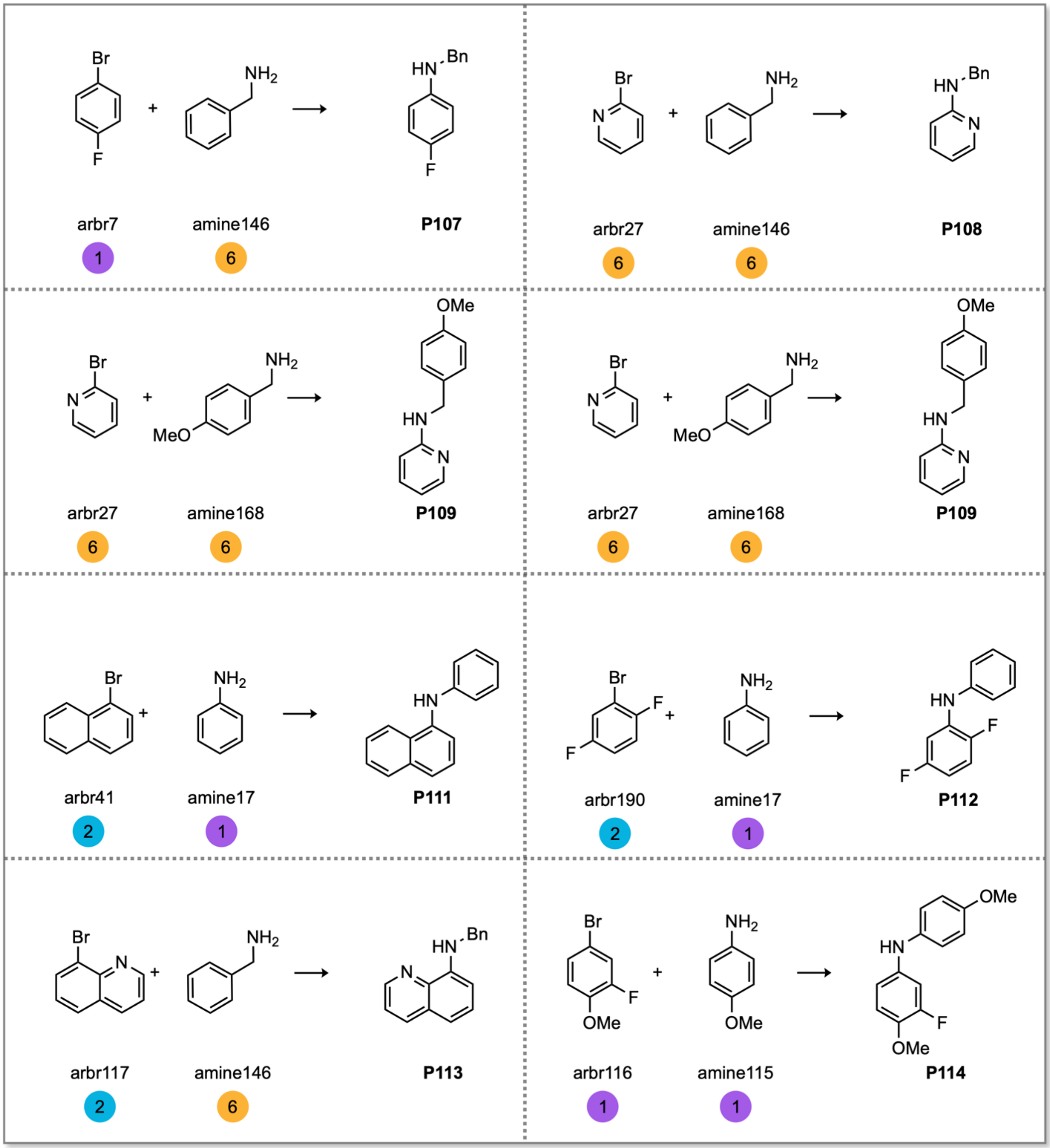

Figure S6. Training set of C–N products (**P107-P114**).

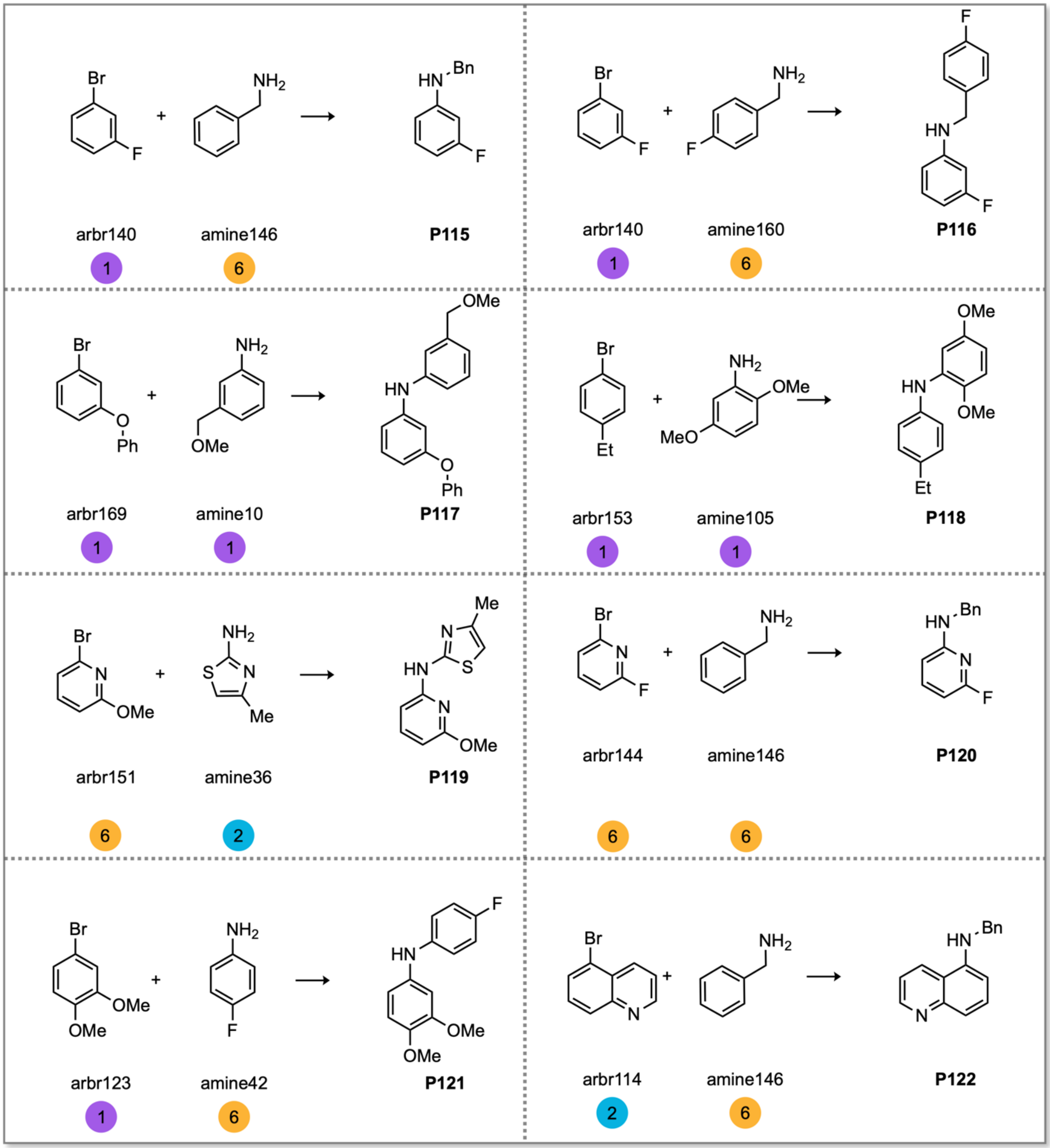

Figure S7. Training set of C–N products (**P115-P122**).

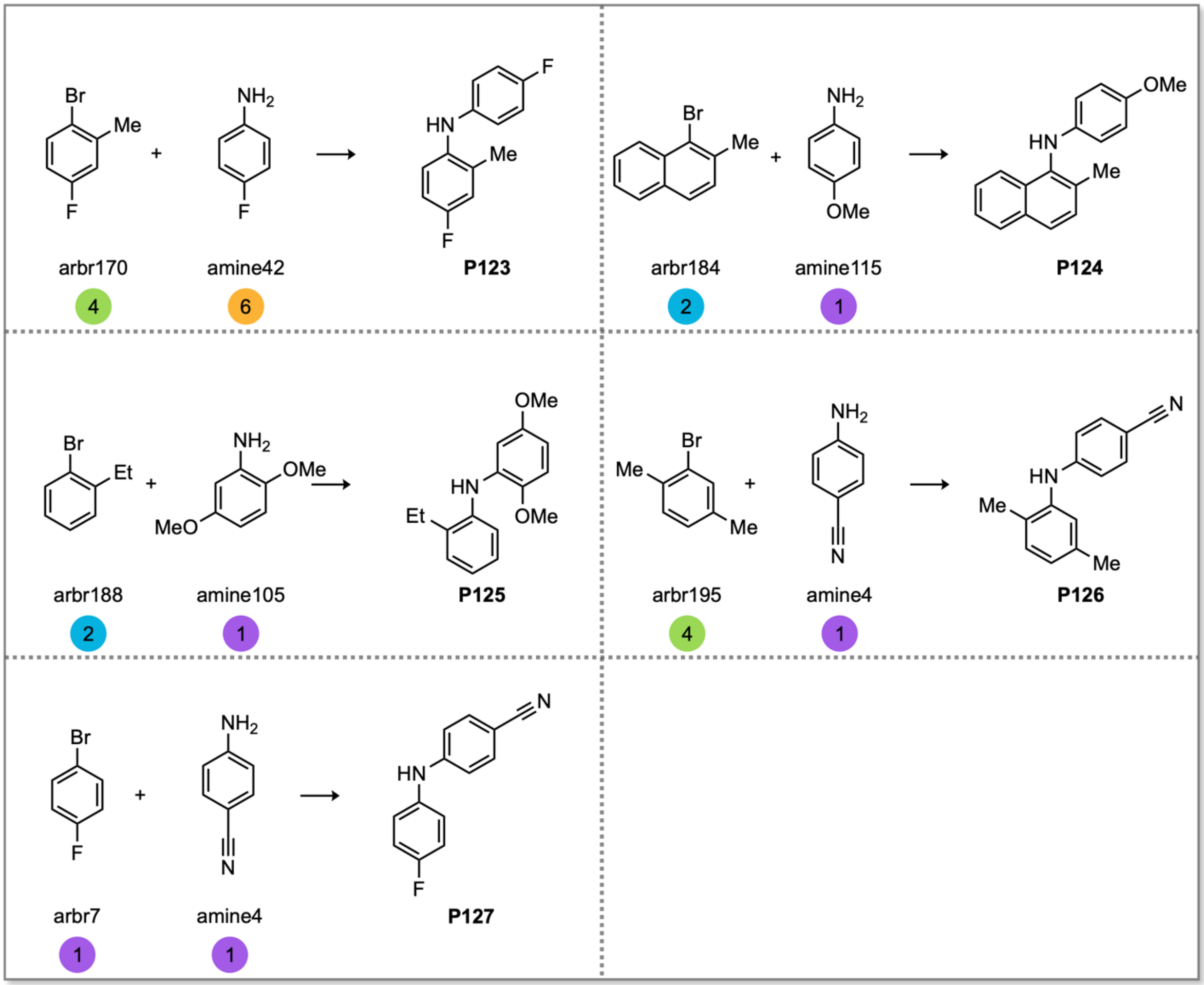

Figure S8. Training set of C–N products (**P123-P127**).

Experimental details

General supplies

All reactions were performed using oven-dried glassware equipped with rubber or PTFE/silicon septa and conducted under a positive nitrogen pressure, unless stated otherwise. Product standards were synthesized in an inert glovebox using 20 mL scintillation vials (KIMBLE® 20 mL Glass Scintillation Vial, Linerless Part Number: 74511-20). All solvents used were either commercially supplied or dried by a PureSolv™ system from Innovative Technology, Inc. Solvents obtained from commercial suppliers underwent freeze pump thawing and dried over activated molecular sieves (4 Å) prior to use. All solvents were tested using a Mettler Toledo Coulometric Karl Fischer Titrator™ and measured to contain less than 20 ppm H_2O. Additionally, all solvents were stored in a nitrogen-filled glovebox and sealed with a Teflon septum wrapped in electrical tape. Stainless steel syringes were utilized for the transfer of air- and moisture-sensitive liquids. Finnpipette™ F2 Variable Volume Pipettes (ThermoFisher Scientific catalog #4642010) were employed for all liquid and stock solution dosing of HTE plates. The volume ranges of pipettes and pipette tips varied across five ranges: 0.2-10 µL, 1-200 µL, 5-300 µL, 100-1000 µL, and 50-1200 µL. All plate dosing was carried out within a nitrogen-filled glovebox using stock solutions of all reaction components, unless otherwise specified. All non-HTE reactions were monitored using thin-layer chromatography (TLC) whenever applicable. Merck Kieselgel 60 F254 fluorescent-treated silica plates were employed for TLC, which were visualized under UV light or stained with aqueous basic potassium permanganate for visualization. Flash column chromatography (FCC) was conducted using Silicycle SiliaFlash® automated column chromatography. For isolation and purification of all product standards, SiliCycle™ SiliaSep™ Premium Flash Cartridges (40 g, 25 µm, 90 Å) were used (SiliCycle Product #FLH-10095D-A-ISO40).

Materials and reagents

All starting materials and explicitly noted product standards were purchased from commercial sources (Millipore-Sigma, Alfa Aesar, Strem, TCI-America, Combi-Blocks, Oakwood Chemical, A2B Chemical or Matrix Scientific) and were used without any further purification. Deuterated Chloroform ($CDCl_3$) was purchased from Cambridge Isotope Laboratories and were dried with activated molecular sieves (4 Å) overnight prior to use. Deuterated Dimethyl sulfoxide (DMSO-d_6) was purchased from Cambridge Isotope Laboratories and was used without further purification. *N*,*N*-Dimethylacetamide (DMA) was purchased from Millipore-Sigma (Part # 271012-1L) and stored in a nitrogen filled glovebox. Smaller aliquots of DMA were taken as needed and dried over activated molecular sieves prior to reaction set up. Toluene (PhMe) was filtered through packed columns of neutral alumina and CuO under a nitrogen atmosphere. Solvents for extractions, crystallizations and precipitations or flash column chromatography were purchased in ACS Reagent Grade from Millipore-Sigma and ThermoFischer Scientific suppliers. Potassium phosphate (K_3PO_4) was purchased from Millipore-Sigma (Part # RDD019-500G) as the anhydrous, free-flowing, Redi-Dri™, reagent grade powered. K_3PO_4 was used as is from the commercial supplier without further purification. Copper iodide (CuI) was purchased from Millipore-Sigma (Part # 215554-25G) as the 99.999% trace metals basis and used without further purification. All components unless otherwise noted were stored permanently inside a nitrogen filled glovebox.

Spectroscopy and analytical equipment

Proton nuclear magnetic resonance (^1H NMR) spectra and carbon nuclear magnetic resonance (^{13}C NMR) spectra were recorded on a Bruker Ascend 500 NMR (500 MHz) spectrometer. Chemical shifts for protons are reported in parts per million (ppm, δ scale) downfield from tetramethylsilane and are referenced to residual protium in the deuterated solvent ($CHCl_3$ = δ 7.26 or $(CH_3)_2SO$ = δ 2.52). Chemical shifts for carbon are reported in parts per million (ppm, δ scale) downfield from tetramethylsilane and are referenced to the carbon resonances of the solvent ($CDCl_3$ = δ 77.2 or $(CD_3)_2SO$ = δ 40.45). ^1H NMR spectroscopic data are reported as follows: chemical shift in ppm (multiplicity, coupling constants J (Hz), integration intensity). The multiplicities are abbreviated as follows: singlet (s), broad singlet

(br s), doublet (d), triplet (t), quartet (q) heptet (h) multiplet (m). ^{13}C NMR spectroscopic data are reported as follows: chemical shift in ppm (multiplicity, coupling constants J (Hz). All fluorine (^{19}F) chemical shifts are expressed in parts per million and are not referenced to any specific resonance. All raw .fid files were processed, and the resulting spectra were analyzed using the program MestReNOVA (Version:14.1.0-24037) (Release: 2019-08-27). Infrared (IR) spectra were obtained using an Agilent CARY 630 FTIR. All IR spectra was obtained with Diamond ATR methods and neat application of materials unless otherwise specified. Only selected absorption maxima (νmax) are reported in wavenumbers (cm^{-1}). High resolution mass spectra (HRMS) were measured on a Waters Xevo G2-XS Quadrupole Time-of-Flight (QTof) Mass Spectrometer with Electrospray Ionization (ESI). All high-throughput reaction assays were developed on a Waters Acquity H Class UPLC Plus system. All screening reactions were assayed on a Acquity UPLC HSS Cyano 1.8µm 2.1mm x 100 mm column (Part #186005988). All assays were developed on a water (H_2O) /acetonitrile (MeCN) gradient (90%-10% H_2O / 10%-90% MeCN gradient. UPLC grade H_2O (Optima LC/MS Suitable for UHPLC-UV Gradient. Peak Height with PDA (200-400 nm) was found to be 2 mAU max. Solvent was 0.03 micron filtered and was purchased through Fischer Scientific. UPLC grade MeCN (Optima LC/MS Suitable for UHPLC-UV Gradient. Peak Height with PDA (200-400 nm) was found to be 2 mAU max. Solvent was 0.1 micron filtered and was purchased through Fischer Scientific. Trifluoroacetic acid (TFA) was used as a buffer for all assayed reactions. TFA was purchased through ThermoScientific and was purchased as the LC/MS grade reagent (Part #85183). TFA was used without further purification or preparation.

General product synthesis for analytical samples

Products were either commercially supplied or synthesized from their respective starting materials. Commercial products are labeled as **P103**, **P104**, **P105**, **P108**, **P109**, **P110** and **P111** (FigureS4–S6). General synthetic procedures were adapted from previous literature techniques[43]. Liquid aryl bromides and primary amines were degassed with argon prior to introduction into the glove box. To an oven-dried scintillation vial the reaction tube was equipped with a Teflon-coated magnetic stir bar. The aryl bromide starting material was then added under a positive flow of nitrogen (aryl bromide, 1.00 equiv.). The aryl or alkyl primary amine was then added under a positive flow of nitrogen (amine, 1.2 equiv.). Under the same positive flow of nitrogen, NaO*t*Bu (1.40 equiv.), and BrettPhos Pd Gen3 catalyst (3 mol%) were added to the reaction vessel. The reaction tube was loosely capped with a screw-thread cap fitted with a Teflon septum. The assembled reaction vessel was brought into a nitrogen-filled glovebox, after which the cap was removed, and anhydrous PhMe (< 20 ppm water by Karl Fisher Titrator™) was added via syringe. The reaction mixture was stirred open to the nitrogen-filled glovebox atmosphere for 30 min. After mixing, the scintillation vial was tightly capped, removed from the glovebox, and placed into an oil bath preheated to 100 °C. After stirring for 24 h at 100 °C, the reaction vessel was removed from the oil bath and allowed to cool to room temperature for 3 h. Then, the reaction solution was diluted with EtOAc (5mL), and the resulting suspension was filtered through a plug of Celite® anchored by a cotton plug. The plug of Celite® was washed with additional aliquots of EtOAc (4 mL) and dichloromethane (CH_2Cl_2) (4 mL). The combined filtrates were concentrated under reduced pressure with the aid of rotary evaporation and the crude residue was purified by automated column chromatography using SiliCycle™ prepacked Flash Cartridges. Column chromatography using SiO_2 supported columns with gradients of CH_2Cl_2 or EtOAc in hexanes were used to yield the C–N coupling product in isolated yields ranging from 35-98%.

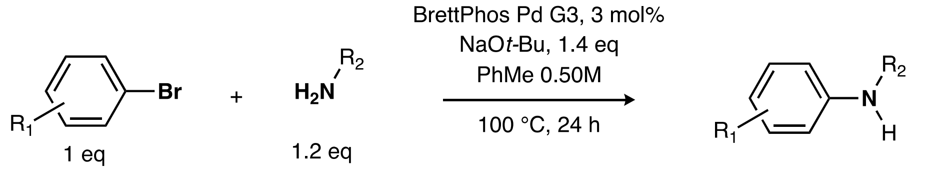

Figure S9. General product synthesis procedure.

Reaction development

Literature Ullmann conditions

The selection of reaction conditions was informed by their prevalence in the Reaxys database as of 10 August 2022. We gathered a library of Ullmann reactions from the Reaxys database using a search for "Ullmann", and subsequently narrowed down the results to those employing copper-catalyzed protocols. This refinement process prioritized conditions cited frequently in literature and took into account practical considerations such as cost and compatibility with our assays.

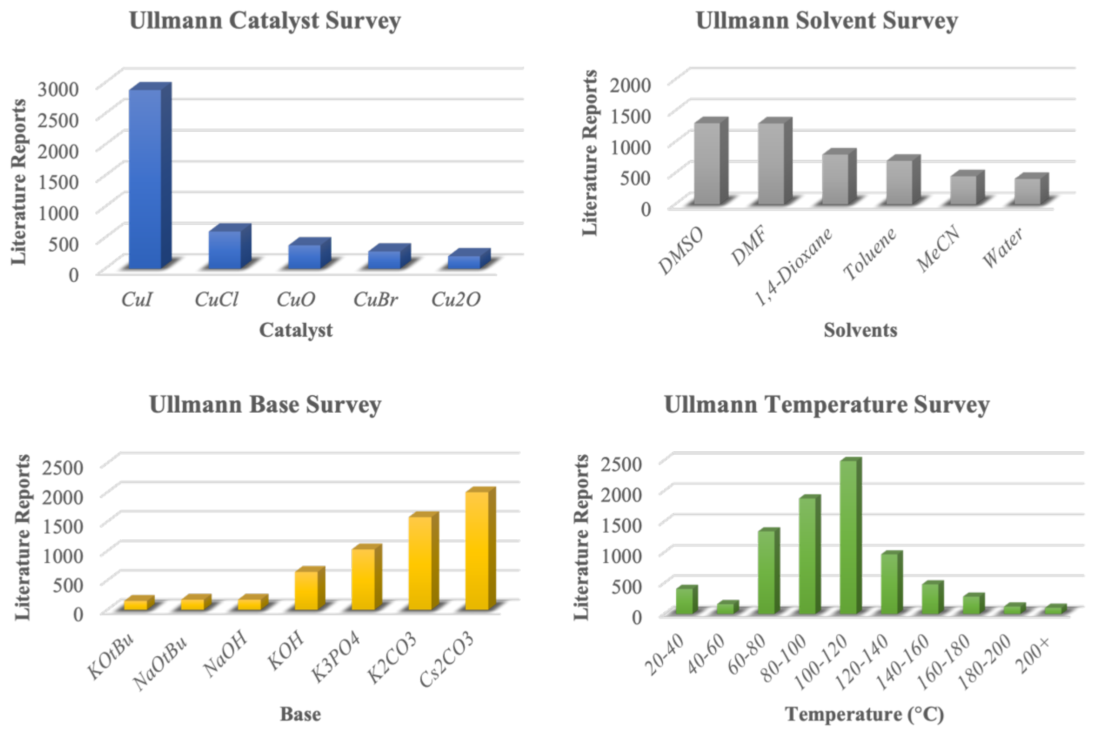

Figure S10. Reaction surveys for Ullmann type couplings.

High-Throughput Screening Information

HTE plate details

High-throughput (HTE) screening assays were developed using oven-dried 1 mL clear glass shell vials measuring 8 x 30 mm (Analytical Sales Part Number: 84001-Case). Prior to use, all vials were oven dried at 120 °C for 48 hours. High-throughput aluminum analytical plates, top/bottom mats, and screw placements were purchased from Analytical Sales (96-Well Aluminum Reaction Block for Glass Inserts Catalog #96973, Top Film Replacement for 96-Well Block Catalog #96967, Top Mats for 96-Well Block Catalog #96965). Refer to FigureS11 for the dimensions of the analytical plate and screw placements. For high-throughput experiments, parylene-coated stir bars with dimensions of 1.67 mm x 2.01 mm x 3.0 mm (Purchased from VP Scientific Part # VP712-1) were used for stirring. The heating block and INKBIRD Temperature controller (Part #741D) from VP Scientific was used for all HTE reaction setups. The tumble stirrer and control unit (Part # VP710C5-7A-CC) were used to stir and heat all analytical plates at a constant speed of 1200 RPM and constant temperature of 110 °C. All reaction blocks were stirred for 24 hours at 100 °C, and then subsequently allowed to cool to room temperature over the course of one hour.

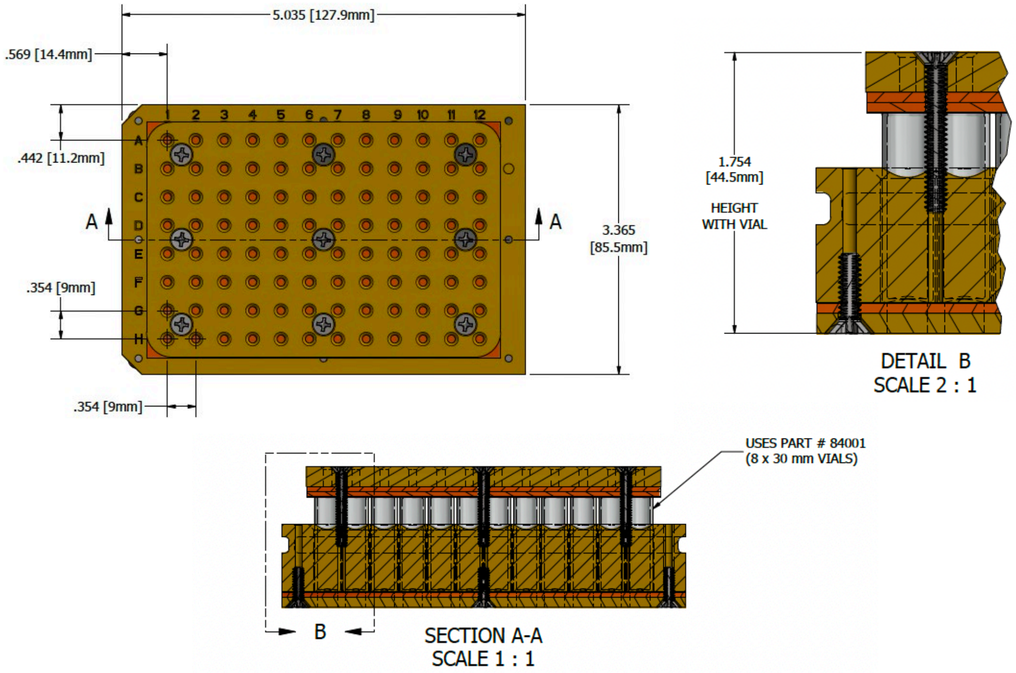

Figure S11. 96-well plate depiction and measurements. Schematic was adopted from Analytical Sales aluminum reaction block (Catalog #96965).

Assay development and control experiments

All reaction screening was assayed on a Acquity UPLC HSS Cyano 1.8µm 2.1mm x 100 mm column. All assays were developed on a Waters Acquity H Class UPLC Plus system using H_2O / MeCN mobile phases (90%-10% H_2O / 10%-90% MeCN). Product ratios were measured by integration of UV absorbance peaks at 254 nm. Product UPLC traces were checked with mass spectral analysis (UPLC-MS) to ensure a matching product mass with the assay developed. UPLC assays were developed with authentic product standards synthesized or purchased as described in the general synthesis section. Pure product samples obtained from chromatography or commercial vendors were used to create calibration curves relating the absorbance ratios of the product with a selected internal standard. Retention times of all products were gathered with a 0.005 M sample of the respective product dissolved in MeCN. Calibration curves were constructed to interpolate mol ratios from measured absorbance ratios from the reaction test tubes [4]. All reactions were dosed with internal standards post reaction time completion. The internal standard of choice varied depending on the assay developed for each product. Phenanthrene and 9-phenylphenanthrene internal standard stock solutions were freshly made for every HTE plate analyzed. Stock solutions were made with 0.1 equivalents of an internal standard dissolved in 80 mL of MeCN and 20 mL DMSO (0.005 M). MeCN and DMSO were both ACS Reagent Grade solvents and were used without further purification. Stock solutions were made in bulk to support 96-well plate distributions and dosing (500 µl doses of internal stock solution injected in each reaction well). UPLC analysis was performed with 50 µl aliquots of the reaction mixture dissolved in 780 µl acetonitrile in an HPLC vial.

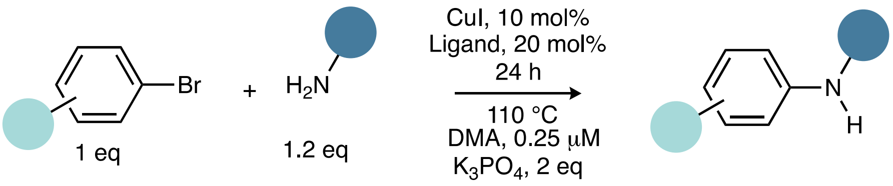

Figure S12. High-throughput screening conditions.

Screening conditions selected for Ullmann catalysis are highlighted in FigureS12. All products underwent S_NAr and ligand-less control experiments to ensure proper analysis of the Ullmann reaction products. S_NAr controls involved the aryl bromide and primary amine components mixed with the respective solvent and base. Ligand-less controls involved the aryl bromide, primary amine, base and copper in the reaction mixture. Products that failed either of these two control tests (i.e., yields > 5%) were removed from the library.

Statistical modeling

Ligand classification model

To reselect ligands based on the initial experimental results for **P100–P111**, we searched for structural similarities among effective ligands using classification models.[23] We determined the maximum yield produced by each ligand and employed single-node decision trees to identify structural features capable of effectively classifying ligands that yielded above 20% from those yielding below 20%. We only utilized the minimum and maximum values of each molecular descriptors in the conformational ensemble because these values can directly relate to the active conformer in a reaction. We identified two structural features that exhibited high classification accuracy (0.96 accuracy and 0.95 F1 score): the minimum Cu–L distance (*d*) within the conformer ensemble and the maximum highest occupied molecular orbital (HOMO) energy within the conformer ensemble. We selected the Cu–L distance as the most suitable descriptor because it resulted in a more even data distribution, reduced sensitivity to the DFT level of theory, and potential extractability from X-ray crystal structures when available. The classification model led to the selection of 12 new ligands, all of which met the threshold and yielded above 20%. The code was adapted from ref 23.

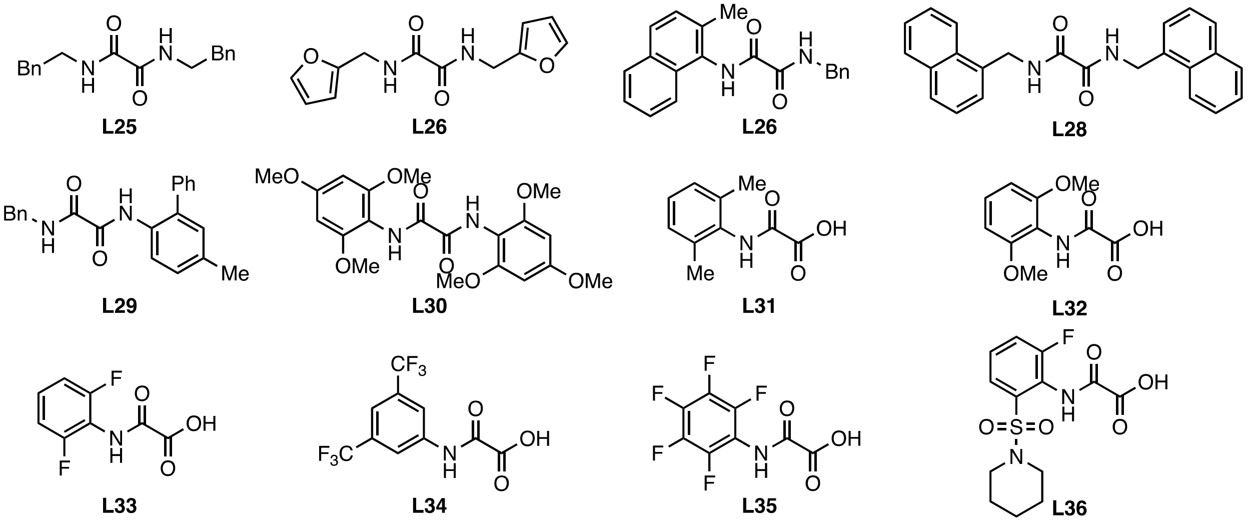

Figure S13. New ligands selected.

Decision tree (training and validation)

We used a dataset consisting of 720 experimental yields to train a decision tree classifier algorithm. The yields were categorized into off (< 20% yield) and on (> 20% yield) classes. Before training the decision tree, we streamlined the pool of molecular descriptors through a series of filters. This involved excluding descriptors with low interpretability (μ, η, ω, and polarizability), descriptors that could be captured by a different molecular descriptor (NMR shielding, Hirshfeld charge, sterimol, and %VBur within 3.0 – 4.0Å), and descriptors exhibiting high co-linear correlations (R^2 > 0.95) with other descriptors. (code available: https://github.com/SigmanGroup/ullmann_project/):

To train the decision tree classifier, we began by conducting a grid search. During the search, we evaluated the accuracy of multiple trees across various hyperparameter configurations, including criteria (gini or entropy), class weights (1:3, 1:4, or 1:5, between off and on classes), and maximum depths (ranging from 2 to 5). This evaluation was performed on a training set consisting of 75% of the total training dataset (random split, random state = 1), and evaluated with 4-fold cross-validation. The outcome of our analysis revealed that a decision tree with the criterion set to entropy, a class weight ratio of 1:3 (off:on), and a maximum depth of 3 yielded the highest cross-validation accuracy, which was 0.85.

In the test set, the trained decision tree exhibited an accuracy of 0.88. Additionally, it achieved a precision of 0.89, recall of 0.88, and F1 score of 0.84. The confusion matrix of the test set demonstrates that the decision tree performs similarly for both classes. It achieved an accuracy of 0.88 for predicting “off” reactivity and an accuracy of 0.86 for predicting “on” reactivity, Figure S14. Furthermore, to assess the consistency of our data split, we employed 4-fold cross-validation on the entire dataset. The average cross-validation score across the folds was found to be 0.86. The individual scores for each fold were as follows: 0.88, 0.82, 0.87, and 0.86, indicating a stable and reliable performance of the trained model across different data splits.

The decision tree consisted of three nodes, one for each component of the reaction. The initial node is defined by the minimum Cu–L distance (*d*) within the conformational ensemble. The subsequent node relies on the maximum primary amine nitrogen NBO charge within the ensemble. Lastly, the terminal node is determined by the minimum %VBur computed on the ipso-carbon of the aryl bromides within the conformational ensemble. Table S1 provides a comprehensive overview of statistics for individual nodes, including accuracy, precision for predicting "off" reactivity, and precision for predicting "on" reactivity. Notably, the precision for predicting "off" reactions demonstrates a consistent high performance across all nodes. As one progresses down the decision tree, there is a noticeable enhancement in precision for predicting "on" reactions. This suggests that the decision tree is effectively learning and refining its predictions at each node, with each node being important.

Additionally, we performed an accuracy test (Table S2) to investigate the potential artificial inflation of overall accuracy by Node 1. By focusing solely on nodes 2 and 3 and excluding reactions involving ligands L1–L18, we observed an accuracy of 81%. This result suggests that Node 1 contributes approximately 5% to the overall accuracy.

Table S1. Statistics for the individual nodes.

Node	Accuracy	Precision (Off predictions)	Precision (On predictions)
Node 1	0.51	1.00	0.31
Node 2	0.74	0.91	0.55
Node 3	0.71	0.97	0.65

Table S2. Accuracies for all nodes vs. accuracy considering only nodes 2 and 3.

Predictions	All Nodes	Nodes 2 and 3
True "Off"	369	213
False "Off"	19	19
True "On"	99	99
False "On"	53	53
Accuracy	86.7%	81.3%

After training the model, we leveraged the substrate molecular descriptors present in the decision tree nodes to guide our selection of molecules for external validation. We incorporated 12 new aryl bromides and six new primary amines resulting in a combined total of 20 new products for validation, Figure S15–S17. In total, 80 reactions were conducted, involving 20 different products, each reacting with 4 different ligands (**L1**, **L21**, **L28**, and **L33**). These reactions resulted in 62 yields below 20% and 18 yields above 20%. The decision tree achieved an accuracy of 0.87 on the validation set, accompanied by a weighted-precision of 0.90, a recall of 0.88, and F1-score of 0.88. The confusion matrix shows that the model performance is well-balanced for both classes, Figure S14.

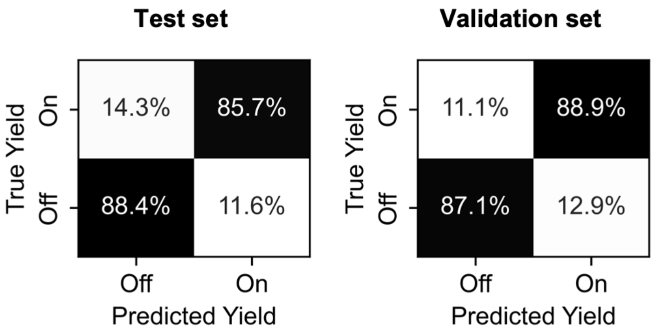

Figure S14. Confusion matrix for the test and validation sets.

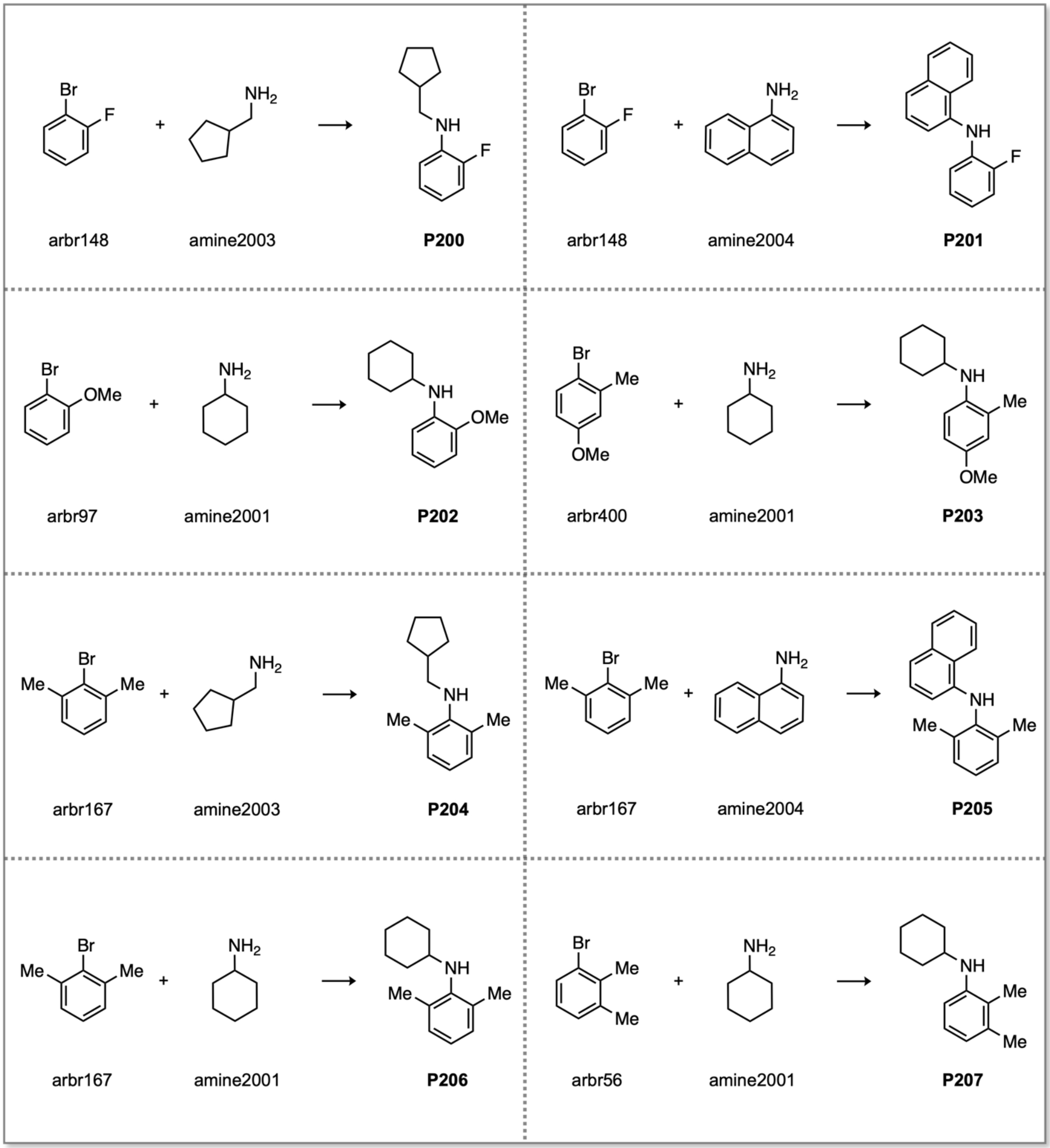

Figure S15. Validation set of C–N products (**P200-P207**).

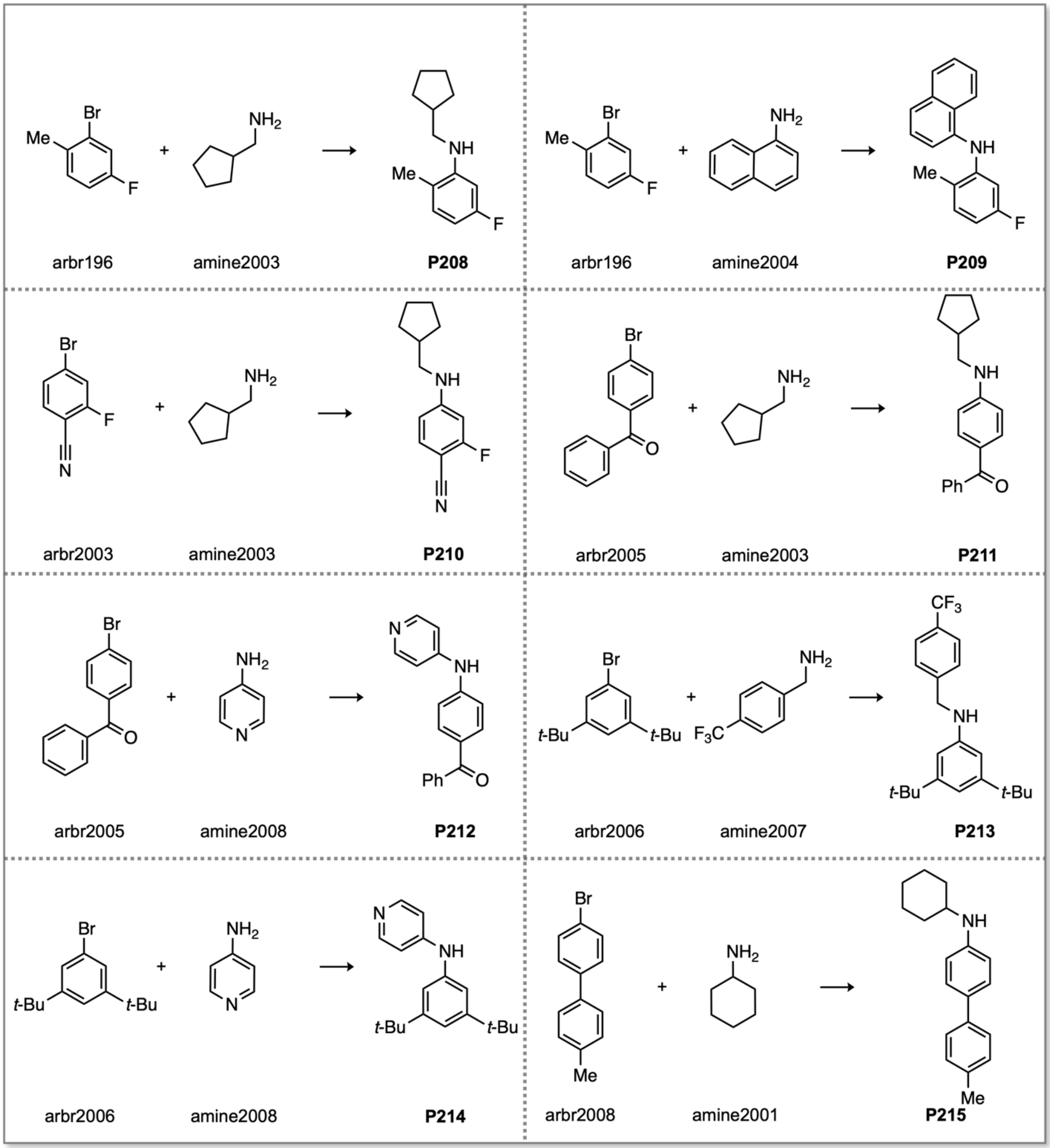

Figure S16. Validation set of C–N products (**P208-P215**).

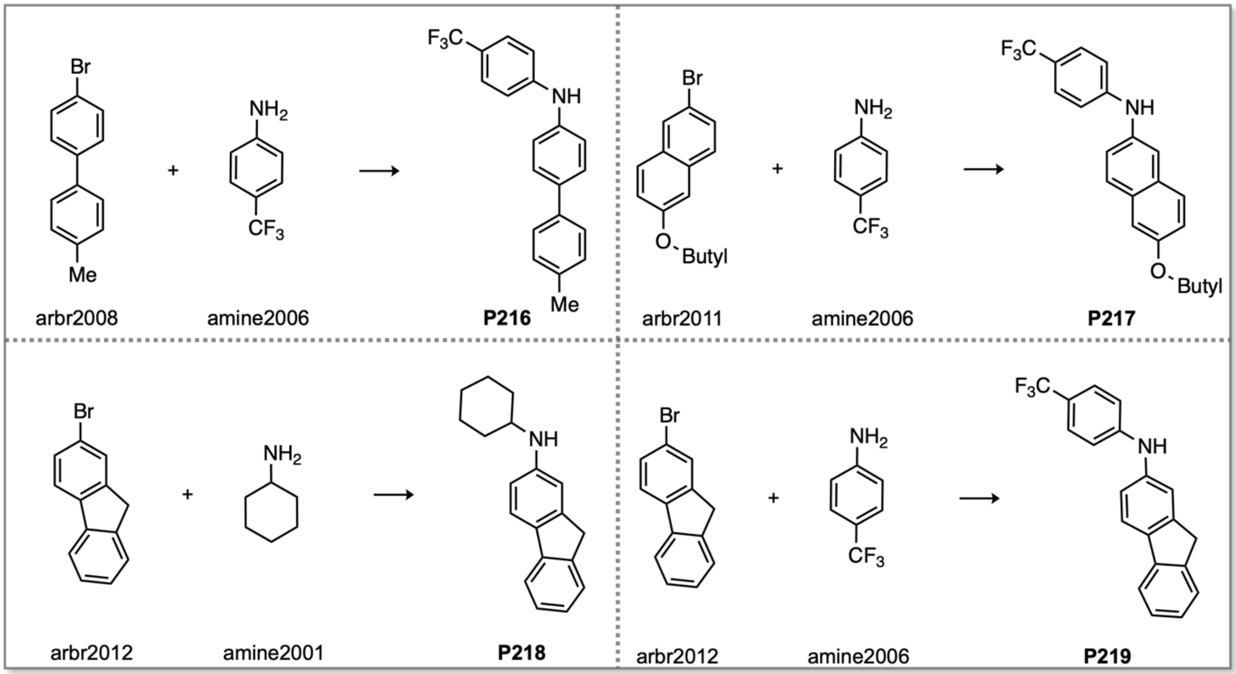

Figure S17. Validation set of C–N products (**P216-P219**).

Confidence map

To address the limitations of the classification model, we searched for ways to quantify and minimize the uncertainty in product predictions. According to the decision tree, the predicted outcome for a combination of aryl bromide and primary amine is consistent across all ligands that satisfy the initial decision node criteria (i.e., ligands with computed $d < 2.07$ Å). For instance, if a combination of substrates is predicted to yield over 20%, this prediction remains the same across 19 different ligands. We envisioned that we could use this observation to calculate the uncertainty in the prediction for each product and later generate a map of uncertainty (or confidence) across the chemical space. We calculated uncertainty using information entropy, a metric that assesses the ratio of correct prediction to the total number of predictions [uncertainty = $-p \cdot \log_2(p)$, where "p" represents the proportion of accurate classifications relative to the overall classifications made]. Subsequently, we converted uncertainty into confidence (confidence = 1 – uncertainty) for enhanced comprehension and interpretation. The confidence value for each product is presented in Table S3. To create the confidence map, we utilized radial basis function interpolation with the SciPy module, employing specific parameters: a linear function, an epsilon value of 0.1, and a smoothing factor of 0.02. The confidence map allows us to predict the confidence value for new combinations of aryl bromide and primary amines.

K-nearest neighbors

To identify the optimal ligands for a specific combination of aryl bromide and primary amine, we developed a tool that search for the two nearest products (determined by the chemical space), and subsequently identifies the top three performing ligands for each of these nearest products. Our findings show that in 22 out of 28 cases (~79%), at least one of the top three performing ligands for a product within the training set also ranks among the top three performing ligands for the two nearest neighbors. The percentage increases to 93% (26 out 28 cases) when we expand our analysis to include the top five performing ligands for a product within the top three performing ligands for the two nearest neighbors, Table S4. This analysis shows the potential of using two nearest neighbors to recommend ligands for a new prediction, thus effectively reducing the initial ligand screening workload and enhancing the prospects for successful reactions.

Table S3. Steric hindrance of the aryl bromide (%VBur computed at the ipso-carbon within 2.5Å), $N^{\delta-}$ (nitrogen NBO charge) of the primary amine, prediction values for the combination of substrates (either "on" or "off"), and the associated confidence values. Positive confidence values indicate the level of confidence in the "on" reactivity prediction, while negative confidence values indicate the level of confidence in the "off" reactivity prediction.

Product	Steric	$N^{\delta-}$	Prediction	Correct predictions	Total predictions	Confidence [%]
**P100**	29.913	–0.808	On	15	19	73.1
**P101**	29.796	–0.792	Off	19	19	–100.0
**P102**	33.719	–0.779	Off	17	19	–85.6
**P103**	30.720	–0.827	On	13	19	62.5
**P104**	33.510	–0.827	Off	19	19	–100.0
**P105**	29.945	–0.827	On	17	19	85.6
**P106**	33.510	–0.809	Off	18	19	–92.6
**P107**	30.609	–0.808	On	10	19	51.3
**P108**	29.945	–0.808	On	14	19	67.5
**P109**	29.945	–0.810	On	15	19	73.1
**P110**	30.580	–0.792	Off	19	19	–100.0
**P111**	33.719	–0.792	Off	16	19	–79.1
**P112**	31.892	–0.792	Off	16	18	–84.9
**P113**	32.351	–0.808	On	6	18	47.2
**P114**	30.541	–0.794	Off	16	18	–84.9
**P115**	30.644	–0.808	On	11	18	56.6
**P116**	30.644	–0.809	On	14	18	71.8
**P117**	30.570	–0.791	Off	15	18	–78.1
**P118**	30.491	–0.792	Off	18	18	–100.0
**P119**	29.913	–0.798	Off	18	18	–100.0
**P120**	30.027	–0.808	On	12	18	61.0
**P121**	30.461	–0.793	Off	18	18	–100.0
**P122**	33.507	–0.808	On	8	18	48.0
**P123**	33.460	–0.793	Off	16	18	–84.9
**P124**	37.078	–0.794	Off	16	18	–84.9
**P125**	33.950	–0.792	Off	18	18	–100.0
**P126**	33.487	–0.779	Off	17	18	–92.2
**P127**	30.609	–0.779	Off	11	18	–56.6
**P200**	31.788	–0.821	On	3	3	100.0
**P201**	31.788	–0.794	Off	3	3	–100.0
**P202**	32.441	–0.828	On	3	3	100.0
**P203**	33.369	–0.828	On	0	3	0.04
**P204**	36.916	–0.821	Off	3	3	–100.0
**P205**	36.916	–0.794	Off	3	3	–100.0
**P206**	36.916	–0.828	Off	3	3	–100.0
**P207**	33.881	–0.828	Off	3	3	–100.0
**P208**	33.522	–0.821	Off	1	3	–47.2
**P209**	33.522	–0.794	Off	3	3	–100.0
**P210**	30.754	–0.821	On	2	3	61.0
**P211**	30.588	–0.821	On	2	3	61.0
**P212**	30.588	–0.783	Off	3	3	–100.0
**P213**	30.574	–0.806	On	2	3	61.0
**P214**	30.574	–0.783	Off	3	3	–100.0
**P215**	30.569	–0.828	On	2	3	61.0
**P216**	30.569	–0.784	Off	3	3	–100.0
**P217**	30.507	–0.784	Off	3	3	–100.0
**P218**	30.582	–0.828	On	2	3	61.0
**P219**	30.582	–0.784	Off	3	3	–100.0

Table S4. Top 5 ligands, wo nearest products, and whether at least one of the top 5 ligands is in the top 3 ligands of the two nearest products for each product in the training set.

Product	Top 1	Top 2	Top 3	Top 4	Top 5	1st nearest product	2nd nearest product	Top 5 in the 2-NN?
P100	L36	L27	L31	L19	L34	P108	P120	Yes
P101	L18	L11	L24	L21	L22	P121	P118	No
P102	L28	L27	L29	L26	L31	P126	P111	Yes
P103	L30	L28	L26	L31	L33	P105	P109	Yes
P104	L26	L32	L28	L15	L18	P103	P106	Yes
P105	L35	L36	L33	L31	L34	P103	P109	Yes
P106	L29	L25	L33	L32	L24	P122	P113	Yes
P107	L30	L31	L36	L32	L29	P115	P116	Yes
P108	L21	L19	L24	L31	L29	P100	P120	Yes
P109	L19	L24	L31	L21	L36	P108	P100	Yes
P110	L30	L28	L26	L24	L27	P118	P117	Yes
P111	L29	L19	L28	L26	L35	P125	P123	Yes
P112	L24	L23	L20	L22	L36	P110	P117	Yes
P113	L28	L26	L27	L30	L31	P122	P106	Yes
P114	L30	L31	L32	L33	L26	P121	P118	Yes
P115	L30	L36	L28	L27	L29	P107	P116	Yes
P116	L30	L36	L29	L26	L28	P115	P107	Yes
P117	L22	L24	L21	L29	L23	P110	P118	No
P118	L28	L26	L32	L33	L20	P110	P117	Yes
P119	L29	L34	L31	L27	L24	P121	P114	Yes
P120	L33	L36	L24	L31	L32	P108	P100	Yes
P121	L30	L27	L31	L28	L25	P114	P118	Yes
P122	L30	L32	L33	L31	L36	P106	P113	Yes
P123	L26	L29	L25	L23	L22	P111	P125	Yes
P124	L28	L27	L29	L26	L25	P125	P111	Yes
P125	L25	L28	L26	L23	L27	P111	P123	Yes
P126	L26	L22	L34	L29	L25	P102	P111	Yes
P127	L26	L31	L35	L29	L27	P117	P110	Yes

Condition Survey

To evaluate the robustness of our model in predicting yields under varying conditions featured in Ullmann literature [20], we studied the impact of different reaction conditions on the reaction yields. Due to laboratory constraints, the data set was tested under a consistent set of conditions. However, we foresee this model's application under varied reaction scenarios. We examined different conditions using **P105** as a benchmark target (refer to Figure S5). This screening was conducted using the HTE setup previously detailed in Figure S11. We varied the conditions including the choice of base, solvent, and temperature. Our findings indicated that certain conditions were replicable for this specific reference. The conditions screened for the library is presented in the top row of Figure S18. While a subset of the conditions tested were found to be unsuitable, our observations suggest that operable differential conditions can be used instead of the general conditions described in Figure S12.

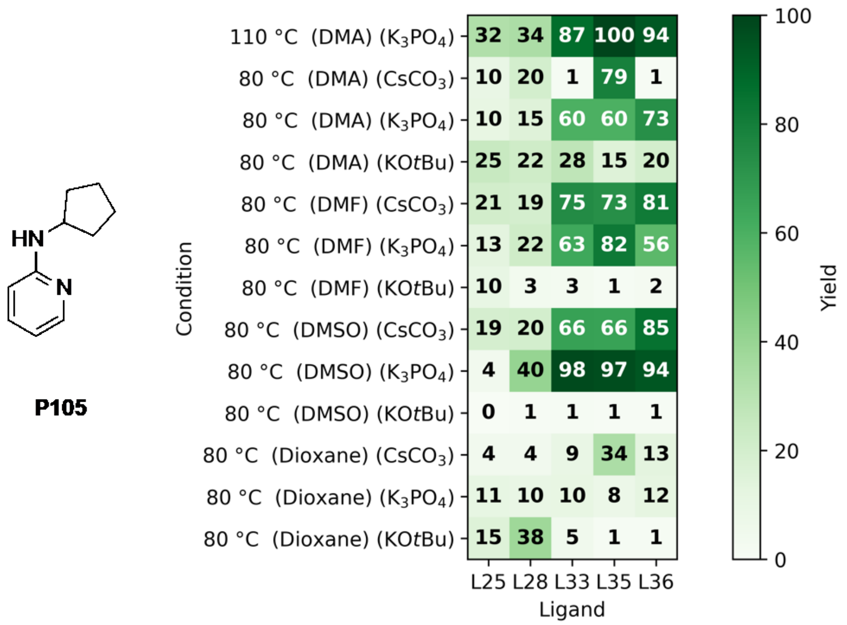

Figure S18. Condition Survey screening using **P105** as a reference.

Data S1: Experimental Screening excel file.

Data S2: Computational and statistical modeling details.

Spectral Analysis Information

Table of Contents

Characterization of C–N Coupling Products……………………………………………………S29

NMR Spectra of C–N Coupling Products…………………………………………………...S77

IR Spectra of C–N Coupling Products…………………………………………………...S175

Mass Analysis Spectra of C–N Coupling Products…………………………………………...S192

UPLC Chromatogram of Products……………………………………………………………S205

Characterization of C–N Coupling Products

Synthesis of *N*-benzyl-6-methoxypyridin-2-amine (P100)

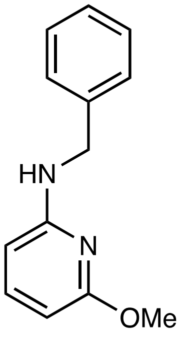

To an oven-dried and torched scintillation vial the reaction tube was equipped with a Teflon-coated magnetic stir bar. 2-Bromo-6-methoxypyridine was then added under a positive flow of nitrogen (2.0 mmol, 1.00 equiv). Phenylmethanamine was then added under a positive flow of nitrogen (2.4 mmol, 1.2 equiv). Under the same positive flow of nitrogen, the addition of NaO*t*-Bu (2.8 mmol, 1.40 equiv), and BrettPhos Pd Gen3 catalyst (0.06 mmol, 3 mol%) were added to the reaction vessel. The reaction tube was loosely capped with a screw-thread caps fitted with Teflon/SIL septa. The assembled reaction vessel was brought into a nitrogen-filled glovebox, after which the cap was removed, and 4 mL of anhydrous toluene (PhMe) (>30ppm water by Karl Fisher Titrator^{TM}) was added *via* syringe. The reaction mixture was stirred open to the nitrogen-filled glovebox atmosphere for 30 min. After complete homogeneity occurred within the reaction vessel, the scintillation vial was tightly capped and brought out of the glovebox, and placed into an oil bath preheated to 100 °C. After stirring for 24 h at 100 °C, the reaction vessel was removed from the oil bath and allowed to cool to room temperature for 3 h. Then, the reaction solution was diluted with EtOAc (5mL), and the resulting suspension was filtered through a plug of celite anchored by a cotton plug. The plug of celite was washed with an additional EtOAc (4mL) and Dichloromethane (CH_2Cl_2) (4mL). The combined filtrates were concentrated under reduced pressure with the aid of a rotary evaporator and the crude residue was purified by automated column chromatography using SiliCycle^{TM} prepacked Flash Cartridges. Purification yielded the C–N coupled product as an off-white solid in 68% isolated yield. Analytical data matched literature references [52].

Chromatography Conditions: SiO_2 supported columns with a gradient of 10% to 100% EtOAc in Hexanes.

^{1}H NMR: (500 MHz, $CDCl_3$): δ 7.48 – 7.41 (m, 1H), 7.37 (dd, J = 7.1, 1.2 Hz, 4H), 7.33 – 7.29 (m, 2H), 6.05 (dd, J = 15.7, 8.1 Hz, 2H), 4.52 (s, 2H), 3.92 (s, 3H).

FT-IR (Diamond-ATR, neat, cm^{-1}): 3224, 2846, 1610, 1245, 1073, 997.

HRMS (ES+): calc: $C_{13}H_{14}N_2O$ $[M+H]^+$: 215.2680.; found: 215.1462.

Synthesis of *N*-phenylthiazol-4-amine (P101)

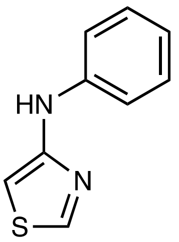

To an oven-dried and torched scintillation vial the reaction tube was equipped with a Teflon-coated magnetic stir bar. 4-Bromothiazole was then added under a positive flow of nitrogen (2.0 mmol, 1.00 equiv). Aniline was then added under a positive flow of nitrogen (2.4 mmol, 1.2 equiv). Under the same positive flow of nitrogen, the addition of NaO*t*-Bu (2.8 mmol, 1.40 equiv), and BrettPhos Pd Gen3 catalyst (0.06 mmol, 3 mol%) were added to the reaction vessel. The reaction tube was loosely capped with a screw-thread caps fitted with Teflon/SIL septa. The assembled reaction vessel was brought into a nitrogen-filled glovebox, after which the cap was removed, and 4 mL of anhydrous toluene (PhMe) (>30ppm water by Karl Fisher Titrator™) was added *via* syringe. The reaction mixture was stirred open to the nitrogen-filled glovebox atmosphere for 30 min. After complete homogeneity occurred within the reaction vessel, the scintillation vial was tightly capped and brought out of the glovebox, and placed into an oil bath preheated to 100 °C. After stirring for 24 h at 100 °C, the reaction vessel was removed from the oil bath and allowed to cool to room temperature for 3 h. Then, the reaction solution was diluted with EtOAc (5mL), and the resulting suspension was filtered through a plug of celite anchored by a cotton plug. The plug of celite was washed with an additional EtOAc (4mL) and Dichloromethane (CH_2Cl_2) (4mL). The combined filtrates were concentrated under reduced pressure with the aid of a rotary evaporator and the crude residue was purified by automated column chromatography using SiliCycle™ prepacked Flash Cartridges. Purification yielded the C–N coupled product as a grey solid in 43% isolated yield. Analytical data matched literature references [53].

Chromatography Conditions: SiO_2 supported columns with a gradient of 5% to 60% EtOAc in Hexanes.

^{1}H NMR: (500 MHz, $CDCl_3$): δ 8.62 (d, J = 2.1 Hz, 1H), 7.34 – 7.28 (m, 2H), 7.17 – 7.11 (m, 2H), 6.96 (tt, J = 7.5, 1.2 Hz, 1H), 6.58 (s, 1H), 6.50 (d, J = 2.1 Hz, 1H).

FT-IR (Diamond-ATR, neat, cm^{-1}): 3241, 2807, 1597, 1536, 1023, 962.

HRMS (ES+): calc: $C_9H_8N_2S$ [M+H]$^+$: 177.2370.; found: 177.1741.

Synthesis of 4-(naphthalen-1-ylamino)benzonitrile (P102)

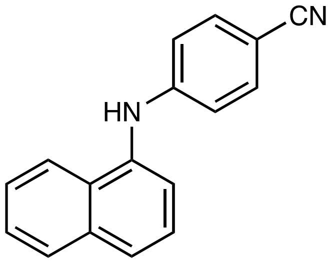

To an oven-dried and torched scintillation vial the reaction tube was equipped with a Teflon-coated magnetic stir bar. 1-Bromonaphthalene was then added under a positive flow of nitrogen (2.0 mmol, 1.00 equiv). 4-Aminobenzonitrile was then added under a positive flow of nitrogen (2.4 mmol, 1.2 equiv). Under the same positive flow of nitrogen, the addition of NaO*t*-Bu (2.8 mmol, 1.40 equiv), and BrettPhos Pd Gen3 catalyst (0.06 mmol, 3 mol%) were added to the reaction vessel. The reaction tube was loosely capped with a screw-thread caps fitted with Teflon/SIL septa. The assembled reaction vessel was brought into a nitrogen-filled glovebox, after which the cap was removed, and 4 mL of anhydrous toluene (PhMe) (>30ppm water by Karl Fisher Titrator^{TM}) was added *via* syringe. The reaction mixture was stirred open to the nitrogen-filled glovebox atmosphere for 30 min. After complete homogeneity occurred within the reaction vessel, the scintillation vial was tightly capped and brought out of the glovebox, and placed into an oil bath preheated to 100 °C. After stirring for 24 h at 100 °C, the reaction vessel was removed from the oil bath and allowed to cool to room temperature for 3 h. Then, the reaction solution was diluted with EtOAc (5mL), and the resulting suspension was filtered through a plug of celite anchored by a cotton plug. The plug of celite was washed with an additional EtOAc (4mL) and Dichloromethane (CH_2Cl_2) (4mL). The combined filtrates were concentrated under reduced pressure with the aid of a rotary evaporator and the crude residue was purified by automated column chromatography using SiliCycle^{TM} prepacked Flash Cartridges. Purification yielded the C–N coupled product as a yellow solid in 52% isolated yield. Analytical data matched literature references [54].

Chromatography Conditions: SiO_2 supported columns with a gradient of 5% to 50% EtOAc in Hexanes.

^{1}H NMR: (500 MHz, $CDCl_3$): δ 7.87 (t, J = 9.2 Hz, 2H), 7.71 (d, J = 7.1 Hz, 1H), 7.54 – 7.34 (m, 6H), 6.78 – 6.70 (m, 2H).

FT-IR (Diamond-ATR, neat, cm^{-1}): 3334, 2219, 1593, 1329, 1142, 969.

HRMS (ES+): calc: $C_{17}H_{12}N_2$ [M+H] $^+$: 245.2970.; found: 245.3950.

Synthesis of *N*-(4-fluorobenzyl)-2-methylaniline (P106)

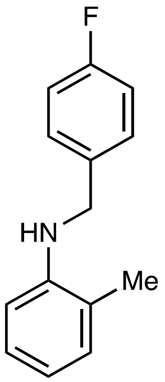

To an oven-dried and torched scintillation vial the reaction tube was equipped with a Teflon-coated magnetic stir bar. 1-Bromo-2-methylbenzene was then added under a positive flow of nitrogen (2.0 mmol, 1.00 equiv). (4-fluorophenyl)methanamine was then added under a positive flow of nitrogen (2.4 mmol, 1.2 equiv). Under the same positive flow of nitrogen, the addition of NaO*t*-Bu (2.8 mmol, 1.40 equiv), and BrettPhos Pd Gen3 catalyst (0.06 mmol, 3 mol%) were added to the reaction vessel. The reaction tube was loosely capped with a screw-thread caps fitted with Teflon/SIL septa. The assembled reaction vessel was brought into a nitrogen-filled glovebox, after which the cap was removed, and 4 mL of anhydrous toluene (PhMe) (>30ppm water by Karl Fisher Titrator™) was added *via* syringe. The reaction mixture was stirred open to the nitrogen-filled glovebox atmosphere for 30 min. After complete homogeneity occurred within the reaction vessel, the scintillation vial was tightly capped and brought out of the glovebox, and placed into an oil bath preheated to 100 °C. After stirring for 24 h at 100 °C, the reaction vessel was removed from the oil bath and allowed to cool to room temperature for 3 h. Then, the reaction solution was diluted with EtOAc (5mL), and the resulting suspension was filtered through a plug of celite anchored by a cotton plug. The plug of celite was washed with an additional EtOAc (4mL) and Dichloromethane (CH_2Cl_2) (4mL). The combined filtrates were concentrated under reduced pressure with the aid of a rotary evaporator and the crude residue was purified by automated column chromatography using SiliCycle™ prepacked Flash Cartridges. Purification yielded the C–N coupled product as a yellow oil in 72% isolated yield. Analytical data matched literature references [55].

Chromatography Conditions: SiO_2 supported columns with a gradient of 5% to 50% EtOAc in Hexanes.

^{1}H NMR: (500 MHz, $CDCl_3$): δ 7.35 (dd, J = 8.6, 5.4 Hz, 2H), 7.14 – 7.07 (m, 2H), 7.07 – 6.98 (m, 2H), 6.73 (t, J = 7.4 Hz, 1H), 6.64 (d, J = 7.9 Hz, 1H), 4.36 (s, 2H), 2.19 (s, 3H).

FT-IR (Diamond-ATR, neat, cm^{-1}): 3438, 3053, 1605, 1506, 1219, 874.

HRMS (ES+): calc: $C_{14}H_{14}FN$ $[M+H]^+$: 216.2714.; found: 216.1396.

Synthesis of *N*-(4-fluorobenzyl)-2-methylaniline (P107)

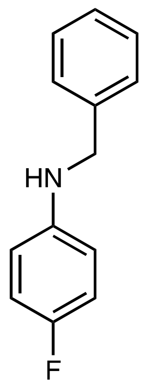

To an oven-dried and torched scintillation vial the reaction tube was equipped with a Teflon-coated magnetic stir bar. 1-Bromo-4-fluorobenzene was then added under a positive flow of nitrogen (2.0 mmol, 1.00 equiv). Phenylmethanamine was then added under a positive flow of nitrogen (2.4 mmol, 1.2 equiv). Under the same positive flow of nitrogen, the addition of NaO*t*-Bu (2.8 mmol, 1.40 equiv), and BrettPhos Pd Gen3 catalyst (0.06 mmol, 3 mol%) were added to the reaction vessel. The reaction tube was loosely capped with a screw-thread caps fitted with Teflon/SIL septa. The assembled reaction vessel was brought into a nitrogen-filled glovebox, after which the cap was removed, and 4 mL of anhydrous toluene (PhMe) (>30ppm water by Karl Fisher Titrator™) was added *via* syringe. The reaction mixture was stirred open to the nitrogen-filled glovebox atmosphere for 30 min. After complete homogeneity occurred within the reaction vessel, the scintillation vial was tightly capped and brought out of the glovebox, and placed into an oil bath preheated to 100 °C. After stirring for 24 h at 100 °C, the reaction vessel was removed from the oil bath and allowed to cool to room temperature for 3 h. Then, the reaction solution was diluted with EtOAc (5mL), and the resulting suspension was filtered through a plug of celite anchored by a cotton plug. The plug of celite was washed with an additional EtOAc (4mL) and Dichloromethane (CH_2Cl_2) (4mL). The combined filtrates were concentrated under reduced pressure with the aid of a rotary evaporator and the crude residue was purified by automated column chromatography using SiliCycle™ prepacked Flash Cartridges. Purification yielded the C–N coupled product as a yellow oil in 87% isolated yield. Analytical data matched literature references [56].

Chromatography Conditions: SiO_2 supported columns with a gradient of 5% to 50% EtOAc in Hexanes.

^{1}H NMR: (500 MHz, $CDCl_3$): δ 7.32 – 7.24 (m, 4H), 7.20 (dd, J = 11.8, 4.9 Hz, 1H), 6.81 (dd, J = 14.7, 6.1 Hz, 2H), 6.53 (dd, J = 8.8, 4.4 Hz, 2H), 4.22 (s, 2H).

FT-IR (Diamond-ATR, neat, cm^{-1}): 3424, 3062, 2864, 1511, 1325, 1005, 991.

HRMS (ES+): calc: $C_{13}H_{12}FN$ $[M+H]^+$: 202.2444.; found: 202.1947.

Synthesis of 2,5-difluoro-*N*-phenylaniline (P112)

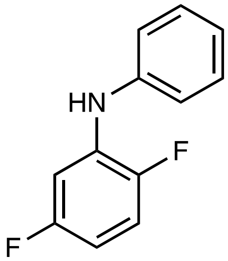

To an oven-dried and torched scintillation vial the reaction tube was equipped with a Teflon-coated magnetic stir bar. 2-bromo-1,4-difluorobenzene was then added under a positive flow of nitrogen (2.0 mmol, 1.00 equiv). Aniline was then added under a positive flow of nitrogen (2.4 mmol, 1.2 equiv). Under the same positive flow of nitrogen, the addition of NaO*t*-Bu (2.8 mmol, 1.40 equiv), and BrettPhos Pd Gen3 catalyst (0.06 mmol, 3 mol%) were added to the reaction vessel. The reaction tube was loosely capped with a screw-thread caps fitted with Teflon/SIL septa. The assembled reaction vessel was brought into a nitrogen-filled glovebox, after which the cap was removed, and 4 mL of anhydrous toluene (PhMe) (>30ppm water by Karl Fisher Titrator™) was added *via* syringe. The reaction mixture was stirred open to the nitrogen-filled glovebox atmosphere for 30 min. After complete homogeneity occurred within the reaction vessel, the scintillation vial was tightly capped and brought out of the glovebox, and placed into an oil bath preheated to 100 °C. After stirring for 24 h at 100 °C, the reaction vessel was removed from the oil bath and allowed to cool to room temperature for 3 h. Then, the reaction solution was diluted with EtOAc (5mL), and the resulting suspension was filtered through a plug of celite anchored by a cotton plug. The plug of celite was washed with an additional EtOAc (4mL) and Dichloromethane (CH_2Cl_2) (4mL). The combined filtrates were concentrated under reduced pressure with the aid of a rotary evaporator and the crude residue was purified by automated

column chromatography using SiliCycle™ prepacked Flash Cartridges. Purification yielded the C–N coupled product as a white solid in 88% isolated yield.

Chromatography Conditions: SiO_2 supported columns with a gradient of 10% to 50% CH_2Cl_2 in Hexanes.

^{1}H NMR: (500 MHz, $CDCl_3$): δ 7.34 (t, J = 7.9 Hz, 2H), 7.17 (dd, J = 9.1, 8.3 Hz, 2H), 7.10 – 7.04 (m, 1H), 7.04 – 6.92 (m, 2H), 6.51 – 6.40 (m, 1H), 5.94 – 5.80 (m, 1H).

^{13}C NMR: (126 MHz, $CDCl_3$): δ 160.11, 158.20, 149.45, 147.58, 140.69, 133.42, 129.58, 123.04, 120.01, 115.60, 105.34, 105.15, 102.75, 102.52.

^{19}F NMR: ($CDCl_3$, 471 MHz): δ -117.29, -139.71.

FT-IR (Diamond-ATR, neat, cm^{-1}): 3440, 3051, 1590, 1521. 1079, 842.

HRMS (ES+): calc: $C_{12}H_9F_2N$ $[M+H]^+$: 206.2078.; found: 206.0777.

Synthesis of *N*-benzylquinolin-8-amine (P113)

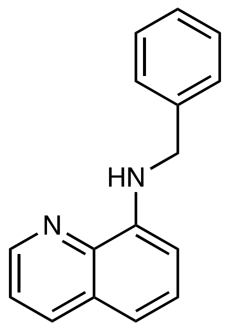

To an oven-dried and torched scintillation vial the reaction tube was equipped with a Teflon-coated magnetic stir bar. 8-bromoquinoline was then added under a positive flow of nitrogen (2.0 mmol, 1.00 equiv). Phenylmethanamine was then added under a positive flow of nitrogen (2.4 mmol, 1.2 equiv). Under the same positive flow of nitrogen, the addition of NaO*t*-Bu (2.8 mmol, 1.40 equiv), and BrettPhos Pd Gen3 catalyst (0.06 mmol, 3 mol%) were added to the reaction vessel. The reaction tube was loosely capped with a screw-thread caps fitted with Teflon/SIL septa. The assembled reaction vessel was brought into a nitrogen-filled glovebox, after which the cap was removed, and 4 mL of anhydrous toluene (PhMe) (>30ppm water by Karl Fisher Titrator™) was added *via* syringe. The reaction mixture was stirred open to the nitrogen-filled glovebox atmosphere for 30 min. After complete homogeneity occurred within the reaction vessel, the scintillation vial was tightly capped and brought out of the glovebox, and placed into an oil bath preheated to 100 °C. After stirring for 24 h at 100 °C, the reaction vessel was removed from the oil bath and allowed to cool to room temperature for 3 h. Then, the reaction solution was diluted with EtOAc (5mL), and the resulting suspension was filtered through a plug of celite anchored by a cotton plug. The plug of celite was washed with an additional EtOAc (4mL) and Dichloromethane (CH_2Cl_2) (4mL). The combined filtrates were concentrated under reduced

pressure with the aid of a rotary evaporator and the crude residue was purified by automated column chromatography using SiliCycle™ prepacked Flash Cartridges. Purification yielded the C–N coupled product as a purple oil in 98% isolated yield.

Chromatography Conditions: SiO_2 supported columns with a gradient of 60% to 100% EtOAc in Hexanes.

^{1}H NMR: (500 MHz, $CDCl_3$): δ 8.63 (dd, J = 4.1, 1.4 Hz, 1H), 7.97 (dd, J = 8.2, 1.3 Hz, 1H), 7.36 (d, J = 7.5 Hz, 2H), 7.30 – 7.16 (m, 5H), 6.97 (d, J = 8.2 Hz, 1H), 6.55 (t, J = 8.0 Hz, 1H), 6.52 (s, 1H), 4.47 (d, J = 5.7 Hz, 2H).

^{13}C NMR: (126 MHz, $CDCl_3$): δ 146.94, 144.62, 139.27, 138.27, 136.03, 128.65, 127.78, 127.44, 127.16, 121.43, 114.17, 105.14, 47.74.

FT-IR (Diamond-ATR, neat, cm^{-1}): 3397, 3062, 1608, 1575, 788, 736.

HRMS (ES+): calc: $C_{16}H_{14}N_2$ $[M+H]^+$: 235.3020.; found: 235.1233.

Synthesis of 3-fluoro-4-methoxy-*N*-(4-methoxyphenyl)aniline (P114)

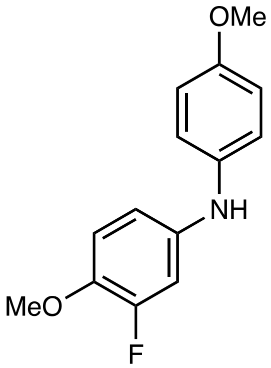

To an oven-dried and torched scintillation vial the reaction tube was equipped with a Teflon-coated magnetic stir bar. 4-Bromo-2-fluoro-1-methoxybenzene was then added under a positive flow of nitrogen (2.0 mmol, 1.00 equiv). 4-Methoxyaniline was then added under a positive flow of nitrogen (2.4 mmol, 1.2 equiv). Under the same positive flow of nitrogen, the addition of NaO*t*-Bu (2.8 mmol, 1.40 equiv), and BrettPhos Pd Gen3 catalyst (0.06 mmol, 3 mol%) were added to the reaction vessel. The reaction tube was loosely capped with a screw-thread caps fitted with Teflon/SIL septa. The assembled reaction vessel was brought into a nitrogen-filled glovebox, after which the cap was removed, and 4 mL of anhydrous toluene (PhMe) (>30ppm water by Karl Fisher Titrator™) was added *via* syringe. The reaction mixture was stirred open to the nitrogen-filled glovebox atmosphere for 30 min. After complete homogeneity occurred within the reaction vessel, the scintillation vial was tightly capped and brought out of the glovebox, and placed into an oil bath preheated to 100 °C. After stirring for 24 h at 100 °C, the reaction vessel was removed from the oil bath and allowed to cool to room temperature for 3 h. Then, the reaction solution was diluted with EtOAc (5mL), and the resulting suspension was filtered through a plug of celite anchored by

a cotton plug. The plug of celite was washed with an additional EtOAc (4mL) and Dichloromethane (CH_2Cl_2) (4mL). The combined filtrates were concentrated under reduced pressure with the aid of a rotary evaporator and the crude residue was purified by automated column chromatography using SiliCycle™ prepacked Flash Cartridges. Purification yielded the C–N coupled product as a black oil in 80% isolated yield.

Chromatography Conditions: SiO_2 supported columns with a gradient of 60% to 100% EtOAc in Hexanes.

^{1}H NMR: (500 MHz, $CDCl_3$): δ 10.19 – 10.11 (m, 3H), 6.99 (d, J = 8.8 Hz, 2121H), 6.85 (dt, J = 8.7, 4.4 Hz, 3494H), 6.72 (dt, J = 7.7, 3.9 Hz, 1316H), 6.64 – 6.58 (m, 1217H), 5.35 (s, 1128H), 3.84 (s, 3585H), 3.79 (s, 3695H).

^{13}C NMR: (126 MHz, $CDCl_3$): δ 155.11, 154.16, 152.22, 141.38, 141.30, 139.56, 139.49, 136.33, 121.27, 115.37, 115.34, 114.79, 112.07, 112.05, 105.62, 105.44, 57.23, 55.61.

^{19}F NMR: ($CDCl_3$, 471 MHz): δ -133.22.

FT-IR (Diamond-ATR, neat, cm^{-1}): 3390. 2837, 1441, 1260, 1118, 826.

HRMS (ES+): calc: $C_{14}H_{14}FNO_2$ $[M+H]^+$: 248.2694.; found: 248.1091.

Synthesis of *N*-benzyl-3-fluoroaniline (P115)

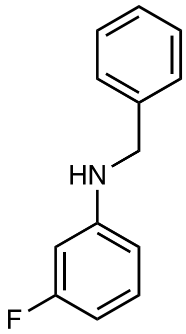

To an oven-dried and torched scintillation vial the reaction tube was equipped with a Teflon-coated magnetic stir bar. 1-Bromo-3-fluorobenzene was then added under a positive flow of nitrogen (2.0 mmol, 1.00 equiv). Phenylmethanamine was then added under a positive flow of nitrogen (2.4 mmol, 1.2 equiv). Under the same positive flow of nitrogen, the addition of NaO*t*-Bu (2.8 mmol, 1.40 equiv), and BrettPhos Pd Gen3 catalyst (0.06 mmol, 3 mol%) were added to the reaction vessel. The reaction tube was loosely capped with a screw-thread caps fitted with Teflon/SIL septa. The assembled reaction vessel was brought into a nitrogen-filled glovebox, after which the cap was removed, and 4 mL of anhydrous toluene (PhMe) (>30ppm water by Karl Fisher Titrator™) was added *via* syringe. The reaction mixture was stirred open to the nitrogen-filled glovebox atmosphere for 30 min. After complete homogeneity occurred within the reaction vessel, the scintillation vial was tightly capped and brought out of the glovebox, and

placed into an oil bath preheated to 100 °C. After stirring for 24 h at 100 °C, the reaction vessel was removed from the oil bath and allowed to cool to room temperature for 3 h. Then, the reaction solution was diluted with EtOAc (5mL), and the resulting suspension was filtered through a plug of celite anchored by a cotton plug. The plug of celite was washed with an additional EtOAc (4mL) and Dichloromethane (CH_2Cl_2) (4mL). The combined filtrates were concentrated under reduced pressure with the aid of a rotary evaporator and the crude residue was purified by automated column chromatography using SiliCycle™ prepacked Flash Cartridges. Purification yielded the C–N coupled product as colorless oil in 77% isolated yield.

Chromatography Conditions: SiO_2 supported columns with a gradient of 10% to 50% CH_2Cl_2 in Hexanes.

^{1}H NMR: (500 MHz, $CDCl_3$): δ 7.36 (dd, J = 9.3, 3.4 Hz, 4H), 7.33 – 7.28 (m, 1H), 7.10 (dd, J = 15.0, 8.0 Hz, 1H), 6.44 – 6.37 (m, 2H), 6.33 (dt, J = 11.6, 2.2 Hz, 1H), 4.32 (d, J = 5.4 Hz, 2H), 4.16 (s, 1H).

^{13}C NMR: (126 MHz, $CDCl_3$): δ 165.11, 163.18, 149.95, 149.87, 138.83, 130.35, 130.27, 128.77, 128.74, 128.72, 127.51, 127.48, 127.43, 127.41, 108.74, 108.72, 104.07, 103.89, 99.64, 99.44, 48.21.

^{19}F NMR: ($CDCl_3$, 471 MHz): δ -112.82.

FT-IR (Diamond-ATR, neat, cm^{-1}): 3424, 2851, 1620, 1148, 971, 826.

HRMS (ES+): calc: $C_{13}H_{12}FN$ $[M+H]^+$: 202.2444.; found: 202.1035.

Synthesis of 3-fluoro-*N*-(4-fluorobenzyl)aniline (P116)

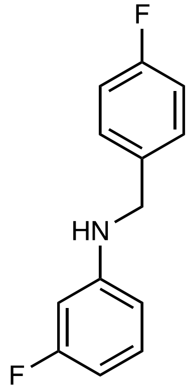

To an oven-dried and torched scintillation vial the reaction tube was equipped with a Teflon-coated magnetic stir bar. 1-Bromo-3-fluorobenzene was then added under a positive flow of nitrogen (2.0 mmol, 1.00 equiv). (4-fluorophenyl)methanamine was then added under a positive flow of nitrogen (2.4 mmol, 1.2 equiv). Under the same positive flow of nitrogen, the addition of NaO*t*-Bu (2.8 mmol, 1.40 equiv), and BrettPhos Pd Gen3 catalyst (0.06 mmol, 3 mol%) were added to the reaction vessel. The reaction tube was loosely capped with a screw-thread caps fitted with Teflon/SIL septa. The assembled reaction vessel was brought into a nitrogen-filled glovebox, after which the cap was removed, and 4 mL of anhydrous toluene

(PhMe) (>30ppm water by Karl Fisher Titrator™) was added *via* syringe. The reaction mixture was stirred open to the nitrogen-filled glovebox atmosphere for 30 min. After complete homogeneity occurred within the reaction vessel, the scintillation vial was tightly capped and brought out of the glovebox, and placed into an oil bath preheated to 100 °C. After stirring for 24 h at 100 °C, the reaction vessel was removed from the oil bath and allowed to cool to room temperature for 3 h. Then, the reaction solution was diluted with EtOAc (5mL), and the resulting suspension was filtered through a plug of celite anchored by a cotton plug. The plug of celite was washed with an additional EtOAc (4mL) and Dichloromethane (CH_2Cl_2) (4mL). The combined filtrates were concentrated under reduced pressure with the aid of a rotary evaporator and the crude residue was purified by automated column chromatography using SiliCycle™ prepacked Flash Cartridges. Purification yielded the C–N coupled product as a colorless oil in 69% isolated yield.

Chromatography Conditions: SiO_2 supported columns with a gradient of 10% to 50% CH_2Cl_2 in Hexanes.

^{1}H NMR: (500 MHz, $CDCl_3$): δ 7.32 (dd, J = 8.1, 5.6 Hz, 2H), 7.10 (dd, J = 15.0, 8.0 Hz, 1H), 7.04 (t, J = 8.6 Hz, 2H), 6.45 – 6.35 (m, 2H), 6.30 (d, J = 11.5 Hz, 1H), 4.29 (d, J = 4.8 Hz, 2H), 4.10 (d, J = 37.0 Hz, 1H).

^{13}C NMR: (126 MHz, $CDCl_3$): δ 165.08, 163.14, 163.13, 161.18, 149.73, 149.64, 134.51, 134.49, 130.38, 130.30, 129.02, 128.97, 128.96, 115.65, 115.48, 108.76, 108.74, 104.25, 104.08, 99.69, 99.49, 47.50.

^{19}F NMR: ($CDCl_3$, 471 MHz): δ -112.72, -115.30.

FT-IR (Diamond-ATR, neat, cm^{-1}): 3429, 2855, 1620, 1508, 1219, 820.

HRMS (ES+): calc: $C_{13}H_{11}F_2N$ $[M+H]^+$: 220.2348.; found: 220.0940.

Synthesis of 3-fluoro-*N*-(4-fluorobenzyl)aniline (P117)

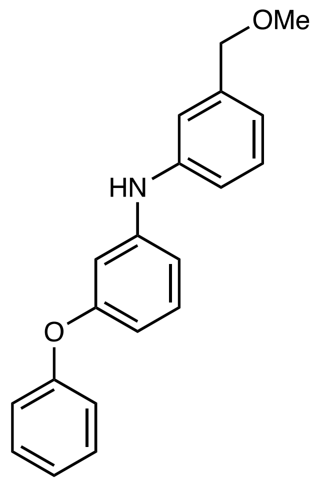

To an oven-dried and torched scintillation vial the reaction tube was equipped with a Teflon-coated magnetic stir bar. 1-Bromo-3-phenoxybenzene was then added under a positive flow of nitrogen (2.0 mmol, 1.00 equiv). 3-(Methoxymethyl)aniline was then added under a positive flow of nitrogen (2.4 mmol, 1.2 equiv). Under the same positive flow of nitrogen, the addition of NaO*t*-Bu (2.8 mmol, 1.40 equiv), and BrettPhos Pd Gen3 catalyst (0.06 mmol, 3 mol%) were added to the reaction vessel. The reaction tube was loosely capped with a screw-thread caps fitted with Teflon/SIL septa. The assembled reaction vessel was brought into a nitrogen-filled glovebox, after which the cap was removed, and 4 mL of anhydrous toluene (PhMe) (>30ppm water by Karl Fisher Titrator™) was added *via* syringe. The reaction mixture was stirred open to the nitrogen-filled glovebox atmosphere for 30 min. After complete homogeneity occurred within the reaction vessel, the scintillation vial was tightly capped and brought out of the glovebox, and placed into an oil bath preheated to 100 °C. After stirring for 24 h at 100 °C, the reaction vessel was removed from the oil bath and allowed to cool to room temperature for 3 h. Then, the reaction solution was diluted with EtOAc (5mL), and the resulting suspension was filtered through a plug of celite anchored by a cotton plug. The plug of celite was washed with an additional EtOAc (4mL) and Dichloromethane (CH_2Cl_2) (4mL). The combined filtrates were concentrated under reduced pressure with the aid of a rotary evaporator and the crude residue was purified by automated column chromatography using SiliCycle™ prepacked Flash Cartridges. Purification yielded the C–N coupled product as a yellow oil in 35% isolated yield.

Chromatography Conditions: SiO_2 supported columns with a gradient of 10% to 50% EtOAc in Hexanes.

^{1}H NMR: (500 MHz, $CDCl_3$): δ 7.24 (t, J = 7.9 Hz, 2H), 7.18 – 7.07 (m, 2H), 7.04 – 6.97 (m, 1H), 6.95 (dt, J = 7.7, 4.5 Hz, 3H), 6.91 (d, J = 8.0 Hz, 1H), 6.85 – 6.78 (m, 1H), 6.72 – 6.66 (m, 1H), 6.62 (s, 1H), 6.45 (ddd, J = 12.0, 5.0, 1.5 Hz, 1H), 5.66 (s, 1H), 4.31 (s, 2H), 3.29 (s, 3H).

^{13}C NMR: (126 MHz, $CDCl_3$) δ 158.46, 157.09, 144.79, 142.68, 139.65, 130.37, 129.73, 129.41, 123.31, 120.76, 119.10, 117.53, 117.46, 112.22, 111.00, 107.72, 74.58, 58.15.

FT-IR (Diamond-ATR, neat, cm^{-1}): 3394, 2820, 1582, 1213, 1083, 688.

HRMS (ES+): calc: $C_{20}H_{19}NO_2$ [M+H] $^+$: 306.3770.; found: 306.1494.

Synthesis of *N*-(4-ethylphenyl)-2,5-dimethoxyaniline (P118)

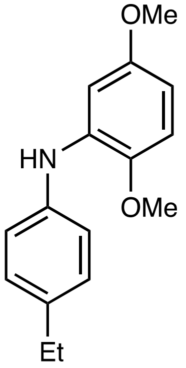

To an oven-dried and torched scintillation vial the reaction tube was equipped with a Teflon-coated magnetic stir bar. 1-Bromo-4-ethylbenzene was then added under a positive flow of nitrogen (2.0 mmol, 1.00 equiv). 2,5-Dimethoxyaniline was then added under a positive flow of nitrogen (2.4 mmol, 1.2 equiv). Under the same positive flow of nitrogen, the addition of NaO*t*-Bu (2.8 mmol, 1.40 equiv), and BrettPhos Pd Gen3 catalyst (0.06 mmol, 3 mol%) were added to the reaction vessel. The reaction tube was loosely capped with a screw-thread caps fitted with Teflon/SIL septa. The assembled reaction vessel was brought into a nitrogen-filled glovebox, after which the cap was removed, and 4 mL of anhydrous toluene (PhMe) (>30ppm water by Karl Fisher Titrator^{TM}) was added *via* syringe. The reaction mixture was stirred open to the nitrogen-filled glovebox atmosphere for 30 min. After complete homogeneity occurred within the reaction vessel, the scintillation vial was tightly capped and brought out of the glovebox, and placed into an oil bath preheated to 100 °C. After stirring for 24 h at 100 °C, the reaction vessel was removed from the oil bath and allowed to cool to room temperature for 3 h. Then, the reaction solution was diluted with EtOAc (5mL), and the resulting suspension was filtered through a plug of celite anchored by a cotton plug. The plug of celite was washed with an additional EtOAc (4mL) and Dichloromethane (CH_2Cl_2) (4mL). The combined filtrates were concentrated under reduced pressure with the aid of a rotary evaporator and the crude residue was purified by automated column chromatography using SiliCycle^{TM} prepacked Flash Cartridges. Purification yielded the C–N coupled product as a yellow oil in 72% isolated yield.

Chromatography Conditions: SiO_2 supported columns with a gradient of 10% to 50% CH_2Cl_2 in Hexanes.

^{1}H NMR: (500 MHz, $CDCl_3$): δ 7.18 – 7.07 (m, 4H), 6.86 (d, J = 2.9 Hz, 1H), 6.79 (d, J = 8.7 Hz, 1H), 6.32 (dd, J = 8.7, 2.9 Hz, 1H), 6.14 (s, 1H), 3.86 (s, 3H), 3.74 (s, 3H), 2.63 (q, J = 7.6 Hz, 2H), 1.25 (t, J = 7.6 Hz, 3H).

^{13}C NMR: (126 MHz, $CDCl_3$): δ 154.30, 142.32, 139.68, 137.92, 134.97, 128.67, 120.04, 111.09, 102.20, 100.92, 56.26, 55.63, 28.22, 15.76.

FT-IR (Diamond-ATR, neat, cm^{-1}): 3412, 2833, 1601, 1161, 824, 777.

HRMS (ES+): calc: $C_{16}H_{19}NO_2$ $[M+H]^+$: 258.3330.; found: 258.1497.

Synthesis of *N*-(6-methoxypyridin-2-yl)-4-methylthiazol-2-amine (P119)

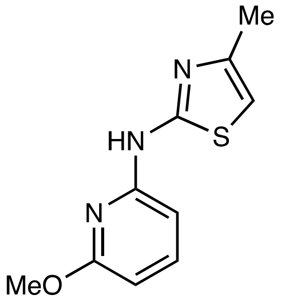

To an oven-dried and torched scintillation vial the reaction tube was equipped with a Teflon-coated magnetic stir bar. 2-Bromo-6-methoxypyridine was then added under a positive flow of nitrogen (2.0 mmol, 1.00 equiv). 4-Methylthiazol-2-amine was then added under a positive flow of nitrogen (2.4 mmol, 1.2 equiv). Under the same positive flow of nitrogen, the addition of NaO*t*-Bu (2.8 mmol, 1.40 equiv), and BrettPhos Pd Gen3 catalyst (0.06 mmol, 3 mol%) were added to the reaction vessel. The reaction tube was loosely capped with a screw-thread caps fitted with Teflon/SIL septa. The assembled reaction vessel was brought into a nitrogen-filled glovebox, after which the cap was removed, and 4 mL of anhydrous toluene (PhMe) (>30ppm water by Karl Fisher Titrator™) was added *via* syringe. The reaction mixture was stirred open to the nitrogen-filled glovebox atmosphere for 30 min. After complete homogeneity occurred within the reaction vessel, the scintillation vial was tightly capped and brought out of the glovebox, and placed into an oil bath preheated to 100 °C. After stirring for 24 h at 100 °C, the reaction vessel was removed from the oil bath and allowed to cool to room temperature for 3 h. Then, the reaction solution was diluted with EtOAc (5mL), and the resulting suspension was filtered through a plug of celite anchored by a cotton plug. The plug of celite was washed with an additional EtOAc (4mL) and Dichloromethane (CH_2Cl_2) (4mL). The combined filtrates were concentrated under reduced pressure with the aid of a rotary evaporator and the crude residue was purified by automated column chromatography using SiliCycle™ prepacked Flash Cartridges. Purification yielded the C–N coupled product as an off-white solid in 44% isolated yield.

Chromatography Conditions: SiO_2 supported columns with a gradient of 60% to 80% EtOAc in Hexanes.

^{1}H NMR: (500 MHz, $CDCl_3$): δ 8.51 – 8.32 (m, 1H), 7.40 (dd, J = 10.0, 5.8 Hz, 1H), 6.29 (s, 1H), 6.23 (dd, J = 11.6, 7.9 Hz, 2H), 4.02 (s, 3H), 2.26 (s, 3H).

^{13}C NMR: (126 MHz, $CDCl_3$): δ 163.53, 159.77, 147.04, 140.22, 105.59, 101.69, 101.18, 54.93, 17.19.

FT-IR (Diamond-ATR, neat, cm^{-1}): 3241, 2842, 1539, 1394, 1135, 779.

HRMS (ES+): calc: $C_{10}H_{11}N_3OS$ $[M+H]^+$: 222.2780.; found: 222.0701.

Synthesis of *N*-benzyl-6-fluoropyridin-2-amine (P120)

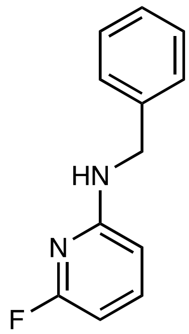

To an oven-dried and torched scintillation vial the reaction tube was equipped with a Teflon-coated magnetic stir bar. 2-Bromo-6-fluoropyridine was then added under a positive flow of nitrogen (2.0 mmol, 1.00 equiv). Phenylmethanamine was then added under a positive flow of nitrogen (2.4 mmol, 1.2 equiv). Under the same positive flow of nitrogen, the addition of NaO*t*-Bu (2.8 mmol, 1.40 equiv), and BrettPhos Pd Gen3 catalyst (0.06 mmol, 3 mol%) were added to the reaction vessel. The reaction tube was loosely capped with a screw-thread caps fitted with Teflon/SIL septa. The assembled reaction vessel was brought into a nitrogen-filled glovebox, after which the cap was removed, and 4 mL of anhydrous toluene (PhMe) (>30ppm water by Karl Fisher Titrator^{TM}) was added *via* syringe. The reaction mixture was stirred open to the nitrogen-filled glovebox atmosphere for 30 min. After complete homogeneity occurred within the reaction vessel, the scintillation vial was tightly capped and brought out of the glovebox, and placed into an oil bath preheated to 100 °C. After stirring for 24 h at 100 °C, the reaction vessel was removed from the oil bath and allowed to cool to room temperature for 3 h. Then, the reaction solution was diluted with EtOAc (5mL), and the resulting suspension was filtered through a plug of celite anchored by a cotton plug. The plug of celite was washed with an additional EtOAc (4mL) and Dichloromethane (CH_2Cl_2) (4mL). The combined filtrates were concentrated under reduced pressure with the aid of a rotary evaporator and the crude residue was purified by automated column chromatography using SiliCycle^{TM} prepacked Flash Cartridges. Purification yielded the C–N coupled product as a white solid in 33% isolated yield.

Chromatography Conditions: SiO_2 supported columns with a gradient of 10% to 80% EtOAc in Hexanes.

^{1}H NMR: (500 MHz, $CDCl_3$): δ 7.46 (q, J = 8.1 Hz, 1H), 7.34 (t, J = 6.0 Hz, 4H), 7.29 (dt, J = 11.9, 3.7 Hz, 1H), 6.22 – 6.13 (m, 2H), 4.96 – 4.83 (m, 1H), 4.49 (d, J = 5.8 Hz, 2H).

^{13}C NMR: (126 MHz, $CDCl_3$): δ 198.44, 157.89, 157.75, 141.85, 141.78, 138.55, 128.72, 127.43, 102.71, 96.25, 95.96, 46.30.

^{19}F NMR: ($CDCl_3$, 471 MHz): δ -69.67.

FT-IR (Diamond-ATR, neat, cm^{-1}): 3276, 2905, 1580, 1228, 736.

HRMS (ES+): calc: $C_{12}H_{11}FN_2$ $[M+H]^+$: 203.2324.; found: 203.0990.

Synthesis of *N*-(4-fluorophenyl)-3,4-dimethoxyaniline (P121)

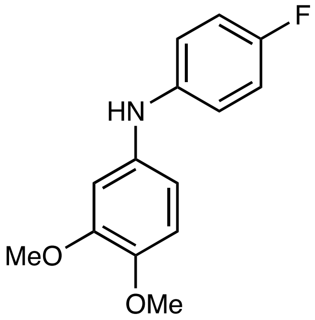

To an oven-dried and torched scintillation vial the reaction tube was equipped with a Teflon-coated magnetic stir bar. 4-Bromo-1,2-dimethoxybenzene was then added under a positive flow of nitrogen (2.0 mmol, 1.00 equiv). 4-Fluoroaniline was then added under a positive flow of nitrogen (2.4 mmol, 1.2 equiv). Under the same positive flow of nitrogen, the addition of NaO*t*-Bu (2.8 mmol, 1.40 equiv), and BrettPhos Pd Gen3 catalyst (0.06 mmol, 3 mol%) were added to the reaction vessel. The reaction tube was loosely capped with a screw-thread caps fitted with Teflon/SIL septa. The assembled reaction vessel was brought into a nitrogen-filled glovebox, after which the cap was removed, and 4 mL of anhydrous toluene (PhMe) (>30ppm water by Karl Fisher Titrator^{TM}) was added *via* syringe. The reaction mixture was stirred open to the nitrogen-filled glovebox atmosphere for 30 min. After complete homogeneity occurred within the reaction vessel, the scintillation vial was tightly capped and brought out of the glovebox, and placed into an oil bath preheated to 100 °C. After stirring for 24 h at 100 °C, the reaction vessel was removed from the oil bath and allowed to cool to room temperature for 3 h. Then, the reaction solution was diluted with EtOAc (5mL), and the resulting suspension was filtered through a plug of celite anchored by a cotton plug. The plug of celite was washed with an additional EtOAc (4mL) and Dichloromethane (CH_2Cl_2) (4mL). The combined filtrates were concentrated under reduced pressure with the aid of a rotary evaporator and the crude residue was purified by automated column chromatography

using SiliCycle™ prepacked Flash Cartridges. Purification yielded the C–N coupled product as a black oil in 74% isolated yield.

Chromatography Conditions: SiO_2 supported columns with a gradient of 10% to 80% EtOAc in Hexanes.

^{1}H NMR: (500 MHz, $CDCl_3$): δ 6.97 – 6.87 (m, 4H), 6.81 (t, J = 8.1 Hz, 1H), 6.62 (t, J = 5.6 Hz, 1H), 6.59 (dd, J = 8.5, 2.3 Hz, 1H), 5.41 (s, 1H), 3.85 (s, 3H), 3.83 (s, 3H).

^{13}C NMR: (126 MHz, $CDCl_3$): δ 158.30, 156.41, 149.79, 144.47, 140.80, 140.78, 137.21, 118.39, 118.33, 115.95, 115.78, 112.39, 111.06, 104.50, 56.36, 55.88.

^{19}F NMR: ($CDCl_3$, 471 MHz): δ -123.88.

FT-IR (Diamond-ATR, neat, cm^{-1}): 3368, 2835, 1498, 1210, 1131, 1023, 826.

HRMS (ES+): calc: $C_{14}H_{14}FNO_2$ $[M+H]^+$: 248.2694.; found: 248.1090.

Synthesis of *N*-benzylquinolin-5-amine (P122)

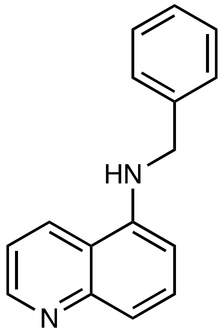

To an oven-dried and torched scintillation vial the reaction tube was equipped with a Teflon-coated magnetic stir bar. 5-Bromoquinoline was then added under a positive flow of nitrogen (2.0 mmol, 1.00 equiv). Phenylmethanamine was then added under a positive flow of nitrogen (2.4 mmol, 1.2 equiv). Under the same positive flow of nitrogen, the addition of NaO*t*-Bu (2.8 mmol, 1.40 equiv), and BrettPhos Pd Gen3 catalyst (0.06 mmol, 3 mol%) were added to the reaction vessel. The reaction tube was loosely capped with a screw-thread caps fitted with Teflon/SIL septa. The assembled reaction vessel was brought into a nitrogen-filled glovebox, after which the cap was removed, and 4 mL of anhydrous toluene (PhMe) (>30ppm water by Karl Fisher Titrator™) was added *via* syringe. The reaction mixture was stirred open to the nitrogen-filled glovebox atmosphere for 30 min. After complete homogeneity occurred within the reaction vessel, the scintillation vial was tightly capped and brought out of the glovebox, and placed into an oil bath preheated to 100 °C. After stirring for 24 h at 100 °C, the reaction vessel was removed from the oil bath and allowed to cool to room temperature for 3 h. Then, the reaction solution was diluted with EtOAc (5mL), and the resulting suspension was filtered through a plug of celite anchored by a cotton plug. The plug of celite was washed with an additional EtOAc (4mL) and

Dichloromethane (CH_2Cl_2) (4mL). The combined filtrates were concentrated under reduced pressure with the aid of a rotary evaporator and the crude residue was purified by automated column chromatography using SiliCycle™ prepacked Flash Cartridges. Purification yielded the C–N coupled product as a yellow solid in 26% isolated yield.

Chromatography Conditions: SiO_2 supported columns with a gradient of 10% to 80% EtOAc in Hexanes.

^{1}H NMR: (500 MHz, $CDCl_3$): δ 8.89 (d, J = 4.0 Hz, 1H), 8.19 (d, J = 8.5 Hz, 1H), 7.54 (dt, J = 16.5, 8.2 Hz, 2H), 7.45 (d, J = 7.5 Hz, 2H), 7.39 (t, J = 7.5 Hz, 2H), 7.33 (dd, J = 7.9, 5.0 Hz, 2H), 6.68 (d, J = 7.4 Hz, 1H), 4.66 (d, J = 20.1 Hz, 1H), 4.50 (d, J = 5.2 Hz, 2H).

^{13}C NMR: (126 MHz, $CDCl_3$): δ 150.06, 149.25, 143.37, 138.58, 130.36, 128.83, 128.63, 127.78, 127.63, 119.39, 118.94, 118.45, 105.15, 48.69.

FT-IR (Diamond-ATR, neat, cm^{-1}): 3260, 2911, 1418, 1126, 1010, 706.

HRMS (ES+): calc: $C_{16}H_{14}N_2$ $[M+H]^+$: 235.3020.; found: 235.1238.

Synthesis of 4-fluoro-*N*-(4-fluorophenyl)-2-methylaniline (P123)

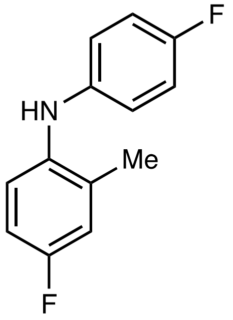

To an oven-dried and torched scintillation vial the reaction tube was equipped with a Teflon-coated magnetic stir bar. 1-Bromo-4-fluoro-2-methylbenzene was then added under a positive flow of nitrogen (2.0 mmol, 1.00 equiv). 4-Fluoroaniline was then added under a positive flow of nitrogen (2.4 mmol, 1.2 equiv). Under the same positive flow of nitrogen, the addition of NaO*t*-Bu (2.8 mmol, 1.40 equiv), and BrettPhos Pd Gen3 catalyst (0.06 mmol, 3 mol%) were added to the reaction vessel. The reaction tube was loosely capped with a screw-thread caps fitted with Teflon/SIL septa. The assembled reaction vessel was brought into a nitrogen-filled glovebox, after which the cap was removed, and 4 mL of anhydrous toluene (PhMe) (>30ppm water by Karl Fisher Titrator™) was added *via* syringe. The reaction mixture was stirred open to the nitrogen-filled glovebox atmosphere for 30 min. After complete homogeneity occurred within the reaction vessel, the scintillation vial was tightly capped and brought out of the glovebox, and placed into an oil bath preheated to 100 °C. After stirring for 24 h at 100 °C, the reaction vessel was removed from the oil bath and allowed to cool to room temperature for 3 h. Then, the reaction solution was diluted with EtOAc (5mL), and the resulting suspension was filtered through

a plug of celite anchored by a cotton plug. The plug of celite was washed with an additional EtOAc (4mL) and Dichloromethane (CH_2Cl_2) (4mL). The combined filtrates were concentrated under reduced pressure with the aid of a rotary evaporator and the crude residue was purified by automated column chromatography using SiliCycle™ prepacked Flash Cartridges. Purification yielded the C–N coupled product as a black oil in 72% isolated yield.

Chromatography Conditions: SiO_2 supported columns with a gradient of 20% to 50% CH_2Cl_2 in Hexanes.

^{1}H NMR: (500 MHz, $CDCl_3$): δ 7.05 (dd, J = 8.7, 5.2 Hz, 1H), 6.95 (dd, J = 14.6, 5.9 Hz, 3H), 6.87 – 6.81 (m, 1H), 6.79 (dt, J = 6.5, 4.0 Hz, 2H), 5.13 (s, 1H), 2.23 (s, 3H).

^{13}C NMR: (126 MHz, $CDCl_3$): δ 159.53, 158.31, 157.61, 156.42, 140.92, 140.90, 137.55, 137.53, 131.82, 131.76, 121.60, 121.54, 118.26, 118.20, 117.53, 117.36, 115.98, 115.80, 113.34, 113.17, 17.99.

^{19}F NMR: ($CDCl_3$, 471 MHz): δ -121.11, -123.98.

FT-IR (Diamond-ATR, neat, cm^{-1}): 3414, 1495, 1269, 1150, 1210, 863, 721.

HRMS (ES+): calc: $C_{13}H_{11}F_2N$ $[M+H]^+$: 220.2348.; found: 220.0943.

Synthesis of *N*-(4-methoxyphenyl)-2-methylnaphthalen-1-amine (P124)

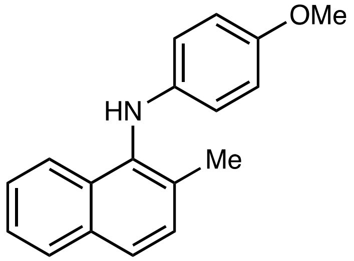

To an oven-dried and torched scintillation vial the reaction tube was equipped with a Teflon-coated magnetic stir bar. 1-Bromo-2-methylnaphthalene was then added under a positive flow of nitrogen (2.0 mmol, 1.00 equiv). 4-Methoxyaniline was then added under a positive flow of nitrogen (2.4 mmol, 1.2 equiv). Under the same positive flow of nitrogen, the addition of NaO*t*-Bu (2.8 mmol, 1.40 equiv), and BrettPhos Pd Gen3 catalyst (0.06 mmol, 3 mol%) were added to the reaction vessel. The reaction tube was loosely capped with a screw-thread caps fitted with Teflon/SIL septa. The assembled reaction vessel was brought into a nitrogen-filled glovebox, after which the cap was removed, and 4 mL of anhydrous toluene (PhMe) (>30ppm water by Karl Fisher Titrator™) was added *via* syringe. The reaction mixture was stirred open to the nitrogen-filled glovebox atmosphere for 30 min. After complete homogeneity occurred within the reaction vessel, the scintillation vial was tightly

capped and brought out of the glovebox, and placed into an oil bath preheated to 100 °C. After stirring for 24 h at 100 °C, the reaction vessel was removed from the oil bath and allowed to cool to room temperature for 3 h. Then, the reaction solution was diluted with EtOAc (5mL), and the resulting suspension was filtered through a plug of celite anchored by a cotton plug. The plug of celite was washed with an additional EtOAc (4mL) and Dichloromethane (CH_2Cl_2) (4mL). The combined filtrates were concentrated under reduced pressure with the aid of a rotary evaporator and the crude residue was purified by automated column chromatography using SiliCycle™ prepacked Flash Cartridges. Purification yielded the C–N coupled product as a purple solid in 89% isolated yield.

Chromatography Conditions: SiO_2 supported columns with a gradient of 20% to 50% CH_2Cl_2 in Hexanes.

^{1}H NMR: (500 MHz, $CDCl_3$): δ 7.96 (dd, J = 7.6, 5.6 Hz, 1H), 7.86 – 7.80 (m, 1H), 7.67 (d, J = 8.4 Hz, 1H), 7.42 (dt, J = 11.2, 6.8 Hz, 3H), 6.77 – 6.68 (m, 2H), 6.52 (t, J = 6.1 Hz, 2H), 5.40 (s, 1H), 3.73 (s, 3H), 2.39 (s, 3H).

^{13}C NMR: (126 MHz, $CDCl_3$): δ 152.72, 140.99, 135.38, 133.38, 131.72, 131.29, 129.30, 128.14, 126.09, 125.51, 125.15, 123.31, 115.27, 114.79, 55.71, 18.42.

FT-IR (Diamond-ATR, neat, cm^{-1}): 3390, 2831, 1387, 1239, 1180, 1036.

HRMS (ES+): calc: $C_{18}H_{17}NO$ $[M+H]^+$: 264.3400.; found: 264.1391.

Synthesis of *N*-(2-ethylphenyl)-2,5-dimethoxyaniline (P125)

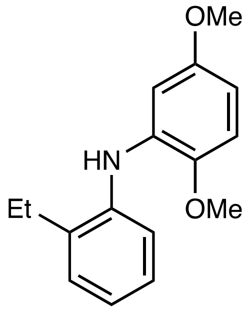

To an oven-dried and torched scintillation vial the reaction tube was equipped with a Teflon-coated magnetic stir bar. 1-Bromo-2-ethylbenzene was then added under a positive flow of nitrogen (2.0 mmol, 1.00 equiv). 2,5-Dimethoxyaniline was then added under a positive flow of nitrogen (2.4 mmol, 1.2 equiv). Under the same positive flow of nitrogen, the addition of NaO*t*-Bu (2.8 mmol, 1.40 equiv), and BrettPhos Pd Gen3 catalyst (0.06 mmol, 3 mol%) were added to the reaction vessel. The reaction tube was loosely capped with a screw-thread caps fitted with Teflon/SIL septa. The assembled reaction vessel was brought into a nitrogen-filled glovebox, after which the cap was removed, and 4 mL of anhydrous toluene (PhMe) (>30ppm water by Karl Fisher Titrator™) was added *via* syringe. The reaction mixture was

stirred open to the nitrogen-filled glovebox atmosphere for 30 min. After complete homogeneity occurred within the reaction vessel, the scintillation vial was tightly capped and brought out of the glovebox, and placed into an oil bath preheated to 100 °C. After stirring for 24 h at 100 °C, the reaction vessel was removed from the oil bath and allowed to cool to room temperature for 3 h. Then, the reaction solution was diluted with EtOAc (5mL), and the resulting suspension was filtered through a plug of celite anchored by a cotton plug. The plug of celite was washed with an additional EtOAc (4mL) and Dichloromethane (CH_2Cl_2) (4mL). The combined filtrates were concentrated under reduced pressure with the aid of a rotary evaporator and the crude residue was purified by automated column chromatography using SiliCycle^{TM} prepacked Flash Cartridges. Purification yielded the C–N coupled product as a colorless oil in 44% isolated yield.

Chromatography Conditions: SiO_2 supported columns with a gradient of 10% to 40% CH_2Cl_2 in Hexanes.

^{1}H NMR: (500 MHz, $CDCl_3$): δ 7.36 (d, J = 7.9 Hz, 1H), 7.25 (d, J = 7.0 Hz, 1H), 7.18 (t, J = 7.6 Hz, 1H), 7.04 (t, J = 7.4 Hz, 1H), 6.79 (d, J = 8.7 Hz, 1H), 6.60 (d, J = 2.9 Hz, 1H), 6.31 (dd, J = 8.7, 2.9 Hz, 1H), 5.97 (s, 1H), 3.88 (s, 3H), 3.71 (s, 3H), 2.65 (q, J = 7.5 Hz, 2H), 1.25 (t, J = 7.6 Hz, 3H).

^{13}C NMR: (126 MHz, $CDCl_3$): δ 154.35, 142.36, 139.70, 136.17, 135.68, 129.06, 126.65, 123.14, 121.39, 111.04, 101.96, 101.10, 56.35, 55.59, 24.39, 14.06.

FT-IR (Diamond-ATR, neat, cm^{-1}): 3424, 2833, 1515, 1211, 1163, 1025, 749.

HRMS (ES+): calc: $C_{16}H_{19}NO_2$ $[M+H]^+$: 258.3330.; found: 258.1494.

Synthesis of 4-((2,5-dimethylphenyl)amino)benzonitrile (P126)

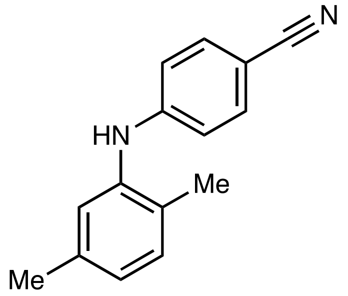

To an oven-dried and torched scintillation vial the reaction tube was equipped with a Teflon-coated magnetic stir bar. 2-Bromo-1,4-dimethylbenzene was then added under a positive flow of nitrogen (2.0 mmol, 1.00 equiv). 4-Aminobenzonitrile was then added under a positive flow of nitrogen (2.4 mmol, 1.2 equiv). Under the same positive flow of nitrogen, the addition of NaO*t*-Bu (2.8 mmol, 1.40 equiv), and BrettPhos Pd Gen3 catalyst (0.06 mmol, 3 mol%) were added to the reaction vessel. The reaction tube was loosely capped with a screw-thread caps fitted with Teflon/SIL septa. The

assembled reaction vessel was brought into a nitrogen-filled glovebox, after which the cap was removed, and 4 mL of anhydrous toluene (PhMe) (>30ppm water by Karl Fisher Titrator™) was added *via* syringe. The reaction mixture was stirred open to the nitrogen-filled glovebox atmosphere for 30 min. After complete homogeneity occurred within the reaction vessel, the scintillation vial was tightly capped and brought out of the glovebox, and placed into an oil bath preheated to 100 °C. After stirring for 24 h at 100 °C, the reaction vessel was removed from the oil bath and allowed to cool to room temperature for 3 h. Then, the reaction solution was diluted with EtOAc (5mL), and the resulting suspension was filtered through a plug of celite anchored by a cotton plug. The plug of celite was washed with an additional EtOAc (4mL) and Dichloromethane (CH_2Cl_2) (4mL). The combined filtrates were concentrated under reduced pressure with the aid of a rotary evaporator and the crude residue was purified by automated column chromatography using SiliCycle™ prepacked Flash Cartridges. Purification yielded the C–N coupled product as a white solid in 72% isolated yield.

Chromatography Conditions: SiO_2 supported columns with a gradient of 0% to 30% CH_2Cl_2 in Hexanes.

^{1}H NMR: (500 MHz, $CDCl_3$): δ 7.45 (d, J = 8.7 Hz, 2H), 7.15 (d, J = 7.7 Hz, 1H), 7.07 (d, J = 10.9 Hz, 1H), 6.94 (t, J = 9.2 Hz, 1H), 6.74 (d, J = 8.7 Hz, 2H), 5.77 – 5.63 (m, 1H), 2.31 (s, 3H), 2.18 (s, 3H).

^{13}C NMR: (126 MHz, $CDCl_3$): δ 149.22, 137.75, 136.90, 133.75, 131.18, 129.47, 126.38, 124.68, 120.07, 114.28, 100.70, 20.97, 17.43.

FT-IR (Diamond-ATR, neat, cm^{-1}): 3343, 1582, 1523, 1172, 1118, 833.

HRMS (ES+): calc: $C_{15}H_{14}N_2$ $[M+H]^+$: 223.2910.; found: 223.1240.

Synthesis of 4-((4-fluorophenyl)amino)benzonitrile (P127)

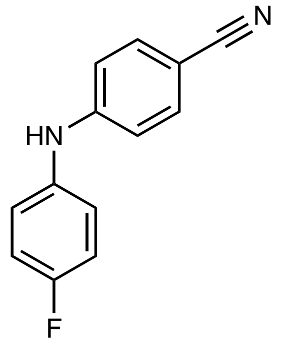

To an oven-dried and torched scintillation vial the reaction tube was equipped with a Teflon-coated magnetic stir bar. 1-Bromo-4-fluorobenzene was then added under a positive flow of nitrogen (2.0 mmol, 1.00 equiv). 4-Aminobenzonitrile was then added under a positive flow of nitrogen (2.4 mmol, 1.2 equiv). Under the same positive flow of nitrogen, the addition of NaO*t*-Bu (2.8 mmol, 1.40 equiv), and BrettPhos Pd Gen3 catalyst (0.06 mmol, 3 mol%) were added to the reaction vessel. The reaction tube was loosely capped with a screw-thread caps fitted with Teflon/SIL septa. The assembled reaction vessel was brought into a nitrogen-filled glovebox, after which the cap was removed, and 4 mL of anhydrous toluene (PhMe) (>30ppm water by Karl Fisher Titrator™) was added *via* syringe. The reaction mixture was stirred open to the nitrogen-filled glovebox atmosphere for 30 min. After complete homogeneity occurred within the reaction vessel, the scintillation vial was tightly capped and brought out of the glovebox, and placed into an oil bath preheated to 100 °C. After stirring for 24 h at 100 °C, the reaction vessel was removed from the oil bath and allowed to cool to room temperature for 3 h. Then, the reaction solution was diluted with EtOAc (5mL), and the resulting suspension was filtered through a plug of celite anchored by a cotton plug. The plug of celite was washed with an additional EtOAc (4mL) and Dichloromethane (CH_2Cl_2) (4mL). The combined filtrates were concentrated under reduced pressure with the aid of a rotary evaporator and the crude residue was purified by automated column chromatography using SiliCycle™ prepacked Flash Cartridges. Purification yielded the C–N coupled product as a off-white solid in 77% isolated yield.

Chromatography Conditions: SiO_2 supported columns with a gradient of 15% to 50% CH_2Cl_2 in Hexanes.

^{1}H NMR: (500 MHz, $CDCl_3$): δ 7.47 (d, J = 8.7 Hz, 2H), 7.14 (dt, J = 8.0, 4.0 Hz, 2H), 7.10 – 7.03 (m, 2H), 6.86 (d, J = 8.7 Hz, 2H), 5.98 – 5.87 (m, 1H).

^{13}C NMR: (126 MHz, $CDCl_3$): δ 160.70, 158.76, 148.63, 133.84, 124.34, 124.27, 116.57, 116.39, 114.35, 101.51.

^{19}F NMR: ($CDCl_3$, 471 MHz): δ -117.55.

FT-IR (Diamond-ATR, neat, cm^{-1}): 336, 2214, 1510, 1172, 779, 744.

HRMS (ES+): calc: $C_{13}H_9FN_2$ [M+H]$^+$: 213.2274.; found: 213.0825.

Synthesis of *N*-(cyclopentylmethyl)-2-fluoroaniline (P200)

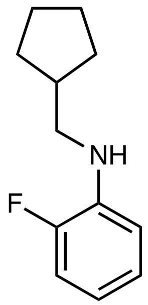

To an oven-dried and torched scintillation vial the reaction tube was equipped with a Teflon-coated magnetic stir bar. 1-Bromo-2-fluorobenzene was then added under a positive flow of nitrogen (2.0 mmol, 1.00 equiv). Cyclopentylmethanamine was then added under a positive flow of nitrogen (2.4 mmol, 1.2 equiv). Under the same positive flow of nitrogen, the addition of NaO*t*-Bu (2.8 mmol, 1.40 equiv), and BrettPhos Pd Gen3 catalyst (0.06 mmol, 3 mol%) were added to the reaction vessel. The reaction tube was loosely capped with a screw-thread caps fitted with Teflon/SIL septa. The assembled reaction vessel was brought into a nitrogen-filled glovebox, after which the cap was removed, and 4 mL of anhydrous toluene (PhMe) (>30ppm water by Karl Fisher Titrator™) was added *via* syringe. The reaction mixture was stirred open to the nitrogen-filled glovebox atmosphere for 30 min. After complete homogeneity occurred within the reaction vessel, the scintillation vial was tightly capped and brought out of the glovebox, and placed into an oil bath preheated to 100 °C. After stirring for 24 h at 100 °C, the reaction vessel was removed from the oil bath and allowed to cool to room temperature for 3 h. Then, the reaction solution was diluted with EtOAc (5mL), and the resulting suspension was filtered through a plug of celite anchored by a cotton plug. The plug of celite was washed with an additional EtOAc (4mL) and Dichloromethane (CH_2Cl_2) (4mL). The combined filtrates were concentrated under reduced pressure with the aid of a rotary evaporator and the crude residue was purified by automated column chromatography using SiliCycle™ prepacked Flash Cartridges. Purification yielded the C–N coupled product as a yellow oil in 53% isolated yield.

Chromatography Conditions: SiO_2 supported columns with a gradient of 15% to 50% EtOAc in Hexanes.

^{1}H NMR: (500 MHz, $CDCl_3$): δ 7.07 – 6.97 (m, 2H), 6.74 (t, J = 8.4 Hz, 1H), 6.65 (td, J = 7.6, 3.9 Hz, 1H), 3.96 (s, 1H), 3.10 (d, J = 7.2 Hz, 2H), 2.30 – 2.17 (m, 1H), 1.89 (td, J = 11.8, 7.1 Hz, 2H), 1.78 – 1.57 (m, 4H), 1.40 – 1.27 (m, 2H).

^{13}C NMR: (126 MHz, $CDCl_3$): δ 152.49, 150.60, 137.22, 137.13, 124.61, 124.58, 116.20, 116.14, 114.38, 114.23, 112.03, 112.00, 49.06, 39.52, 30.68, 25.33.

^{19}**F NMR:** ($CDCl_3$, 471 MHz): δ -137.06.

FT-IR (Diamond-ATR, neat, cm^{-1}): 2335, 2950, 2866, 1511, 1187, 1034, 736.

HRMS (ES+): calc: $C_{12}H_{16}FN$ $[M+H]^+$: 194.2654.; found: 194.1349.

Synthesis of *N*-(2-fluorophenyl)naphthalen-1-amine (P201)

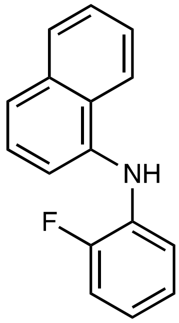

To an oven-dried and torched scintillation vial the reaction tube was equipped with a Teflon-coated magnetic stir bar. 1-Bromo-2-fluorobenzene was then added under a positive flow of nitrogen (2.0 mmol, 1.00 equiv). Naphthalen-1-amine was then added under a positive flow of nitrogen (2.4 mmol, 1.2 equiv). Under the same positive flow of nitrogen, the addition of NaO*t*-Bu (2.8 mmol, 1.40 equiv), and BrettPhos Pd Gen3 catalyst (0.06 mmol, 3 mol%) were added to the reaction vessel. The reaction tube was loosely capped with a screw-thread caps fitted with Teflon/SIL septa. The assembled reaction vessel was brought into a nitrogen-filled glovebox, after which the cap was removed, and 4 mL of anhydrous toluene (PhMe) (>30ppm water by Karl Fisher Titrator^{TM}) was added *via* syringe. The reaction mixture was stirred open to the nitrogen-filled glovebox atmosphere for 30 min. After complete homogeneity occurred within the reaction vessel, the scintillation vial was tightly capped and brought out of the glovebox, and placed into an oil bath preheated to 100 °C. After stirring for 24 h at 100 °C, the reaction vessel was removed from the oil bath and allowed to cool to room temperature for 3 h. Then, the reaction solution was diluted with EtOAc (5mL), and the resulting suspension was filtered through a plug of celite anchored by a cotton plug. The plug of celite was washed with an additional EtOAc (4mL) and Dichloromethane (CH_2Cl_2) (4mL). The combined filtrates were concentrated under reduced pressure with the aid of a rotary evaporator and the crude residue was purified by automated column chromatography using SiliCycle^{TM} prepacked Flash Cartridges. Purification yielded the C–N coupled product as a brown solid in 25% isolated yield.

Chromatography Conditions: SiO_2 supported columns with a gradient of 15% to 50% EtOAc in Hexanes.

^{1}**H NMR:** (500 MHz, $CDCl_3$): δ 8.07 (dd, J = 8.0, 6.8 Hz, 1H), 7.95 – 7.89 (m, 1H), 7.66 (d, J = 7.9 Hz, 1H), 7.54 (pd, J = 6.7, 1.2 Hz, 2H), 7.49 – 7.38 (m, 2H), 7.17 (ddd, J = 11.4, 8.2, 1.0 Hz, 1H), 7.01 (tdd, J = 15.4, 11.3, 4.4 Hz, 2H), 6.89 – 6.79 (m, 1H), 6.27 – 5.92 (m, 1H).

^{13}C NMR: (126 MHz, $CDCl_3$): δ 153.70, 151.79, 137.57, 134.76, 133.52, 133.44, 128.60, 128.41, 126.29, 126.01, 125.96, 124.41, 124.38, 123.97, 121.95, 119.90, 119.84, 117.30, 117.01, 117.00, 115.43, 115.28.

^{19}F NMR: ($CDCl_3$, 471 MHz): δ -133.78.

FT-IR (Diamond-ATR, neat, cm^{-1}): 3389, 1500, 1251, 1189, 1016, 753, 710.

HRMS (ES+): calc: $C_{16}H_{12}FN$ $[M+H]^+$: 238.2774.; found: 238.1033.

Synthesis of *N*-cyclohexyl-2-methoxyaniline (P202)

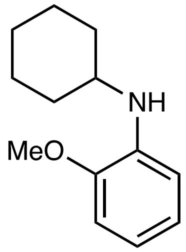

To an oven-dried and torched scintillation vial the reaction tube was equipped with a Teflon-coated magnetic stir bar. 1-Bromo-2-methoxybenzene was then added under a positive flow of nitrogen (2.0 mmol, 1.00 equiv). Cyclohexanamine was then added under a positive flow of nitrogen (2.4 mmol, 1.2 equiv). Under the same positive flow of nitrogen, the addition of NaO*t*-Bu (2.8 mmol, 1.40 equiv), and BrettPhos Pd Gen3 catalyst (0.06 mmol, 3 mol%) were added to the reaction vessel. The reaction tube was loosely capped with a screw-thread caps fitted with Teflon/SIL septa. The assembled reaction vessel was brought into a nitrogen-filled glovebox, after which the cap was removed, and 4 mL of anhydrous toluene (PhMe) (>30ppm water by Karl Fisher Titrator^{TM}) was added *via* syringe. The reaction mixture was stirred open to the nitrogen-filled glovebox atmosphere for 30 min. After complete homogeneity occurred within the reaction vessel, the scintillation vial was tightly capped and brought out of the glovebox, and placed into an oil bath preheated to 100 °C. After stirring for 24 h at 100 °C, the reaction vessel was removed from the oil bath and allowed to cool to room temperature for 3 h. Then, the reaction solution was diluted with EtOAc (5mL), and the resulting suspension was filtered through a plug of celite anchored by a cotton plug. The plug of celite was washed with an additional EtOAc (4mL) and Dichloromethane (CH_2Cl_2) (4mL). The combined filtrates were concentrated under reduced pressure with the aid of a rotary evaporator and the crude residue was purified by automated column chromatography using SiliCycle^{TM} prepacked Flash Cartridges. Purification yielded the C–N coupled product as a colorless oil in 52% isolated yield.

Chromatography Conditions: SiO_2 supported columns with a gradient of 20% to 50% EtOAc in Hexanes.

^{1}H NMR: (500 MHz, $CDCl_3$): δ 6.93 (dd, J = 11.0, 4.3 Hz, 1H), 6.83 (d, J = 7.4 Hz, 1H), 6.70 (t, J = 7.1 Hz, 2H), 4.23 (s, 1H), 3.90 (s, 3H), 3.39 – 3.27 (m, 1H), 2.21 – 2.10 (m, 2H), 1.91 – 1.80 (m, 2H), 1.78 – 1.69 (m, 1H), 1.54 – 1.40 (m, 2H), 1.38 – 1.20 (m, 3H).

^{13}C NMR: (126 MHz, $CDCl_3$): δ 146.80, 137.36, 121.33, 115.84, 110.27, 109.64, 55.41, 51.41, 33.52, 26.12, 25.17.

FT-IR (Diamond-ATR, neat, cm^{-1}): 3422, 2926, 2853, 1601, 1219, 1124, 1029, 980.

HRMS (ES+): calc: $C_{13}H_{19}NO$ $[M+H]^+$: 206.3010.; found: 206.1546.

Synthesis of *N*-cyclohexyl-4-methoxy-2-methylaniline (P203)

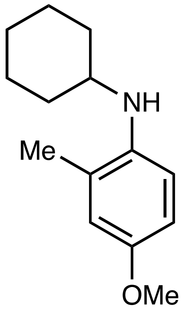

To an oven-dried and torched scintillation vial the reaction tube was equipped with a Teflon-coated magnetic stir bar. 1-Bromo-4-methoxy-2-methylbenzene was then added under a positive flow of nitrogen (2.0 mmol, 1.00 equiv). Cyclohexanamine was then added under a positive flow of nitrogen (2.4 mmol, 1.2 equiv). Under the same positive flow of nitrogen, the addition of NaO*t*-Bu (2.8 mmol, 1.40 equiv), and BrettPhos Pd Gen3 catalyst (0.06 mmol, 3 mol%) were added to the reaction vessel. The reaction tube was loosely capped with a screw-thread caps fitted with Teflon/SIL septa. The assembled reaction vessel was brought into a nitrogen-filled glovebox, after which the cap was removed, and 4 mL of anhydrous toluene (PhMe) (>30ppm water by Karl Fisher Titrator™) was added *via* syringe. The reaction mixture was stirred open to the nitrogen-filled glovebox atmosphere for 30 min. After complete homogeneity occurred within the reaction vessel, the scintillation vial was tightly capped and brought out of the glovebox, and placed into an oil bath preheated to 100 °C. After stirring for 24 h at 100 °C, the reaction vessel was removed from the oil bath and allowed to cool to room temperature for 3 h. Then, the reaction solution was diluted with EtOAc (5mL), and the resulting suspension was filtered through a plug of celite anchored by a cotton plug. The plug of celite was washed with an additional EtOAc (4mL) and Dichloromethane (CH_2Cl_2) (4mL). The combined filtrates were concentrated under reduced pressure with the aid of a rotary evaporator and the crude residue was purified by

automated column chromatography using SiliCycle™ prepacked Flash Cartridges. Purification yielded the C–N coupled product as a purple oil in 47% isolated yield.

Chromatography Conditions: SiO_2 supported columns with a gradient of 15% to 90% EtOAc in Hexanes.

^{1}H NMR: (500 MHz, $CDCl_3$): δ 6.79 – 6.71 (m, 2H), 6.65 (d, J = 8.7 Hz, 1H), 3.79 (s, 3H), 3.33 – 3.20 (m, 1H), 3.10 (s, 1H), 2.17 (d, J = 12.2 Hz, 3H), 2.16 – 2.09 (m, 2H), 1.88 – 1.77 (m, 2H), 1.77 – 1.67 (m, 1H), 1.44 (tt, J = 15.4, 3.3 Hz, 2H), 1.37 – 1.16 (m, 3H).

^{13}C NMR: (126 MHz, $CDCl_3$): δ 151.39, 139.67, 123.81, 117.02, 112.09, 111.87, 55.80, 52.54, 33.84, 26.15, 25.17, 17.89.

FT-IR (Diamond-ATR, neat, cm^{-1}): 3414, 2928, 2853, 1504, 1217, 1049, 794.

HRMS (ES+): calc: $C_{14}H_{21}NO$ $[M+H]^+$: 220.3280.; found: 220.1706.

Synthesis of *N*-(cyclopentylmethyl)-2,6-dimethylaniline (P204)

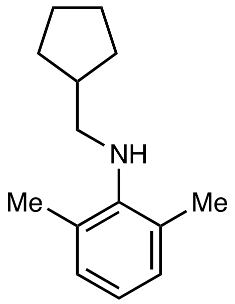

To an oven-dried and torched scintillation vial the reaction tube was equipped with a Teflon-coated magnetic stir bar. 2-Bromo-1,3-dimethylbenzene was then added under a positive flow of nitrogen (2.0 mmol, 1.00 equiv). Cyclopentylmethanamine was then added under a positive flow of nitrogen (2.4 mmol, 1.2 equiv). Under the same positive flow of nitrogen, the addition of NaO*t*-Bu (2.8 mmol, 1.40 equiv), and BrettPhos Pd Gen3 catalyst (0.06 mmol, 3 mol%) were added to the reaction vessel. The reaction tube was loosely capped with a screw-thread caps fitted with Teflon/SIL septa. The assembled reaction vessel was brought into a nitrogen-filled glovebox, after which the cap was removed, and 4 mL of anhydrous toluene (PhMe) (>30ppm water by Karl Fisher Titrator™) was added *via* syringe. The reaction mixture was stirred open to the nitrogen-filled glovebox atmosphere for 30 min. After complete homogeneity occurred within the reaction vessel, the scintillation vial was tightly capped and brought out of the glovebox, and placed into an oil bath preheated to 100 °C. After stirring for 24 h at 100 °C, the reaction vessel was removed from the oil bath and allowed to cool to room temperature for 3 h. Then, the reaction solution was diluted with EtOAc (5mL), and the resulting suspension was filtered through a plug of celite anchored by a cotton plug. The plug of celite was washed with an additional EtOAc (4mL) and Dichloromethane (CH_2Cl_2) (4mL). The combined filtrates were

concentrated under reduced pressure with the aid of a rotary evaporator and the crude residue was purified by automated column chromatography using SiliCycle™ prepacked Flash Cartridges. Purification yielded the C–N coupled product as a yellow oil in 39% isolated yield.

Chromatography Conditions: SiO_2 supported columns with a gradient of 5% to 40% EtOAc in Hexanes.

^{1}H NMR: (500 MHz, $CDCl_3$): δ 7.05 (d, J = 7.5 Hz, 2H), 6.86 (t, J = 7.5 Hz, 1H), 3.20 – 3.03 (m, 1H), 2.98 (d, J = 7.2 Hz, 2H), 2.36 (s, 6H), 2.16 (dq, J = 15.1, 7.6 Hz, 1H), 1.90 (tt, J = 11.8, 5.8 Hz, 2H), 1.75 – 1.59 (m, 4H), 1.40 – 1.27 (m, 2H).

^{13}C NMR: (126 MHz, $CDCl_3$): δ 146.53, 129.01, 128.89, 121.54, 54.26, 41.09, 30.82, 25.47, 18.65.

FT-IR (Diamond-ATR, neat, cm^{-1}): 3427, 2948, 2864, 1474, 1219, 1098, 760.

HRMS (ES+): calc: $C_{14}H_{21}N$ $[M+H]^+$: 204.3290.; found: 204.1750.

Synthesis of *N*-(2,6-dimethylphenyl)naphthalen-1-amine (P205)

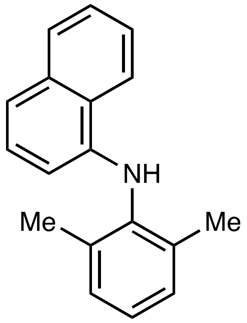

To an oven-dried and torched scintillation vial the reaction tube was equipped with a Teflon-coated magnetic stir bar. 2-Bromo-1,3-dimethylbenzene was then added under a positive flow of nitrogen (2.0 mmol, 1.00 equiv). Naphthalen-1-amine was then added under a positive flow of nitrogen (2.4 mmol, 1.2 equiv). Under the same positive flow of nitrogen, the addition of NaO*t*-Bu (2.8 mmol, 1.40 equiv), and BrettPhos Pd Gen3 catalyst (0.06 mmol, 3 mol%) were added to the reaction vessel. The reaction tube was loosely capped with a screw-thread caps fitted with Teflon/SIL septa. The assembled reaction vessel was brought into a nitrogen-filled glovebox, after which the cap was removed, and 4 mL of anhydrous toluene (PhMe) (>30ppm water by Karl Fisher Titrator™) was added *via* syringe. The reaction mixture was stirred open to the nitrogen-filled glovebox atmosphere for 30 min. After complete homogeneity occurred within the reaction vessel, the scintillation vial was tightly capped and brought out of the glovebox, and placed into an oil bath preheated to 100 °C. After stirring for 24 h at 100 °C, the reaction vessel was removed from the oil bath and allowed to cool to room temperature for 3 h. Then, the reaction solution was diluted with EtOAc (5mL), and the resulting suspension was filtered through a plug of celite anchored by a cotton plug. The plug of

celite was washed with an additional EtOAc (4mL) and Dichloromethane (CH_2Cl_2) (4mL). The combined filtrates were concentrated under reduced pressure with the aid of a rotary evaporator and the crude residue was purified by automated column chromatography using SiliCycle™ prepacked Flash Cartridges. Purification yielded the C–N coupled product as a maroon-colored solid in 76% isolated yield.

Chromatography Conditions: SiO_2 supported columns with a gradient of 0% to 30% EtOAc in Hexanes.

^{1}H NMR: (500 MHz, $CDCl_3$): δ 7.94 (dt, J = 11.0, 4.9 Hz, 1H), 7.72 (td, J = 6.7, 3.8 Hz, 1H), 7.42 – 7.35 (m, 2H), 7.19 (d, J = 8.1 Hz, 1H), 7.09 (t, J = 7.8 Hz, 1H), 7.01 (dt, J = 8.5, 6.6 Hz, 3H), 6.12 (d, J = 7.5 Hz, 1H), 5.57 (s, 1H), 2.09 (s, 6H).

^{13}C NMR: (126 MHz, $CDCl_3$): δ 141.35, 138.84, 135.28, 134.68, 128.84, 128.80, 126.59, 125.92, 125.66, 125.13, 124.14, 120.48, 118.92, 107.39, 18.26.

FT-IR (Diamond-ATR, neat, cm^{-1}): 3412, 2853, 1517, 1334, 1278, 1161, 1019, 764.

HRMS (ES+): calc: $C_{18}H_{17}N$ $[M+H]^+$: 248.3410.; found: 248.1441.

Synthesis of *N*-cyclohexyl-2,6-dimethylaniline (P206)

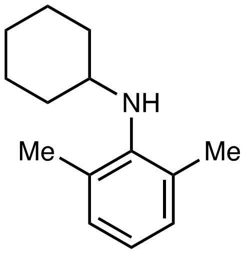

To an oven-dried and torched scintillation vial the reaction tube was equipped with a Teflon-coated magnetic stir bar. 2-Bromo-1,3-dimethylbenzene was then added under a positive flow of nitrogen (2.0 mmol, 1.00 equiv). Cyclohexanamine was then added under a positive flow of nitrogen (2.4 mmol, 1.2 equiv). Under the same positive flow of nitrogen, the addition of NaO*t*-Bu (2.8 mmol, 1.40 equiv), and BrettPhos Pd Gen3 catalyst (0.06 mmol, 3 mol%) were added to the reaction vessel. The reaction tube was loosely capped with a screw-thread caps fitted with Teflon/SIL septa. The assembled reaction vessel was brought into a nitrogen-filled glovebox, after which the cap was removed, and 4 mL of anhydrous toluene (PhMe) (>30ppm water by Karl Fisher Titrator™) was added *via* syringe. The reaction mixture was stirred open to the nitrogen-filled glovebox atmosphere for 30 min. After complete homogeneity occurred within the reaction vessel, the scintillation vial was tightly capped and brought out of the glovebox, and placed into an oil bath preheated to 100 °C. After stirring for 24 h at 100 °C, the reaction vessel was removed from the oil bath and allowed to cool to room temperature for 3 h.

Then, the reaction solution was diluted with EtOAc (5mL), and the resulting suspension was filtered through a plug of celite anchored by a cotton plug. The plug of celite was washed with an additional EtOAc (4mL) and Dichloromethane (CH_2Cl_2) (4mL). The combined filtrates were concentrated under reduced pressure with the aid of a rotary evaporator and the crude residue was purified by automated column chromatography using SiliCycle™ prepacked Flash Cartridges. Purification yielded the C–N coupled product as a yellow oil in 59% isolated yield.

Chromatography Conditions: SiO_2 supported columns with a gradient of 0% to 80% EtOAc in Hexanes.

^{1}H NMR: (500 MHz, $CDCl_3$) δ 7.06 (d, J = 7.5 Hz, 2H), 6.87 (t, J = 7.5 Hz, 1H), 3.09 – 3.01 (m, 1H), 2.97 (s, 1H), 2.35 (s, 6H), 2.11 – 2.00 (m, 2H), 1.88 – 1.79 (m, 2H), 1.77 – 1.66 (m, 1H), 1.39 – 1.18 (m, 5H).

^{13}C NMR: (126 MHz, $CDCl_3$): δ 145.23, 129.07, 128.84, 121.21, 56.29, 35.10, 26.11, 25.71, 19.12.

FT-IR (Diamond-ATR, neat, cm^{-1}): 2928, 2853, 1474, 1450, 1101, 1029, 889.

HRMS (ES+): calc: $C_{14}H_{21}N$ $[M+H]^+$: 204.3290.; found: 204.1754.

Synthesis of *N*-cyclohexyl-2,3-dimethylaniline (P207)

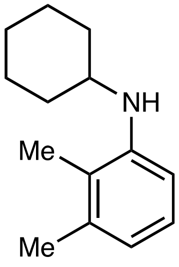

To an oven-dried and torched scintillation vial the reaction tube was equipped with a Teflon-coated magnetic stir bar. 1-Bromo-2,3-dimethylbenzene was then added under a positive flow of nitrogen (2.0 mmol, 1.00 equiv). Cyclohexanamine was then added under a positive flow of nitrogen (2.4 mmol, 1.2 equiv). Under the same positive flow of nitrogen, the addition of NaO*t*-Bu (2.8 mmol, 1.40 equiv), and BrettPhos Pd Gen3 catalyst (0.06 mmol, 3 mol%) were added to the reaction vessel. The reaction tube was loosely capped with a screw-thread caps fitted with Teflon/SIL septa. The assembled reaction vessel was brought into a nitrogen-filled glovebox, after which the cap was removed, and 4 mL of anhydrous toluene (PhMe) (>30ppm water by Karl Fisher Titrator™) was added *via* syringe. The reaction mixture was stirred open to the nitrogen-filled glovebox atmosphere for 30 min. After complete homogeneity occurred within the reaction vessel, the scintillation vial was tightly capped and brought out of the glovebox, and placed into an oil bath preheated to 100 °C. After stirring for 24 h at 100 °C, the reaction vessel was removed from the

oil bath and allowed to cool to room temperature for 3 h. Then, the reaction solution was diluted with EtOAc (5mL), and the resulting suspension was filtered through a plug of celite anchored by a cotton plug. The plug of celite was washed with an additional EtOAc (4mL) and Dichloromethane (CH_2Cl_2) (4mL). The combined filtrates were concentrated under reduced pressure with the aid of a rotary evaporator and the crude residue was purified by automated column chromatography using SiliCycle™ prepacked Flash Cartridges. Purification yielded the C–N coupled product as a brown solid in 62% isolated yield.

Chromatography Conditions: SiO_2 supported columns with a gradient of 0% to 60% EtOAc in Hexanes.

^{1}H NMR: (500 MHz, $CDCl_3$): δ 7.10 – 7.05 (m, 1H), 6.63 (dd, J = 7.5, 4.8 Hz, 2H), 3.44 (d, J = 29.7 Hz, 1H), 3.38 (ddd, J = 13.6, 9.9, 3.6 Hz, 1H), 2.35 (d, J = 7.5 Hz, 3H), 2.16 (dt, J = 15.4, 7.9 Hz, 2H), 2.11 (s, 3H), 1.91 – 1.80 (m, 2H), 1.80 – 1.67 (m, 1H), 1.53 – 1.40 (m, 2H), 1.40 – 1.23 (m, 3H).

^{13}C NMR: (126 MHz, $CDCl_3$): δ 145.29, 136.72, 126.19, 120.01, 118.76, 108.65, 51.81, 33.77, 26.14, 25.14, 20.91, 12.61.

FT-IR (Diamond-ATR, neat, cm^{-1}): 3429, 2928, 2853, 1588, 1476, 1450, 1314.

HRMS (ES+): calc: $C_{14}H_{21}N$ $[M+H]^+$: 204.3290.; found: 204.1756.

Synthesis of *N*-(cyclopentylmethyl)-5-fluoro-2-methylaniline (P208)

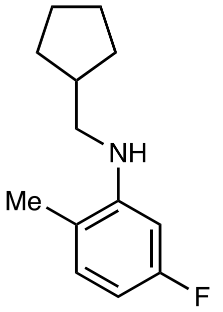

To an oven-dried and torched scintillation vial the reaction tube was equipped with a Teflon-coated magnetic stir bar. 2-Bromo-4-fluoro-1-methylbenzene was then added under a positive flow of nitrogen (2.0 mmol, 1.00 equiv). Cyclopentylmethanamine was then added under a positive flow of nitrogen (2.4 mmol, 1.2 equiv). Under the same positive flow of nitrogen, the addition of NaO*t*-Bu (2.8 mmol, 1.40 equiv), and BrettPhos Pd Gen3 catalyst (0.06 mmol, 3 mol%) were added to the reaction vessel. The reaction tube was loosely capped with a screw-thread caps fitted with Teflon/SIL septa. The assembled reaction vessel was brought into a nitrogen-filled glovebox, after which the cap was removed, and 4 mL of anhydrous toluene (PhMe) (>30ppm water by Karl Fisher Titrator™) was added *via* syringe. The reaction mixture was stirred open to the nitrogen-filled glovebox atmosphere for 30 min. After complete homogeneity

occurred within the reaction vessel, the scintillation vial was tightly capped and brought out of the glovebox, and placed into an oil bath preheated to 100 °C. After stirring for 24 h at 100 °C, the reaction vessel was removed from the oil bath and allowed to cool to room temperature for 3 h. Then, the reaction solution was diluted with EtOAc (5mL), and the resulting suspension was filtered through a plug of celite anchored by a cotton plug. The plug of celite was washed with an additional EtOAc (4mL) and Dichloromethane (CH_2Cl_2) (4mL). The combined filtrates were concentrated under reduced pressure with the aid of a rotary evaporator and the crude residue was purified by automated column chromatography using SiliCycle™ prepacked Flash Cartridges. Purification yielded the C–N coupled product as a yellow oil in 24% isolated yield.

Chromatography Conditions: SiO_2 supported columns with a gradient of 5% to 50% EtOAc in Hexanes.

^{1}H NMR: (500 MHz, $CDCl_3$): δ 7.01 – 6.93 (m, 1H), 6.39 – 6.29 (m, 2H), 3.68 (d, J = 29.0 Hz, 1H), 3.06 (d, J = 7.2 Hz, 2H), 2.30 – 2.18 (m, 1H), 2.11 (s, 3H), 1.88 (td, J = 11.7, 6.9 Hz, 2H), 1.74 – 1.58 (m, 4H), 1.37 – 1.27 (m, 2H).

^{13}C NMR: (126 MHz, $CDCl_3$): δ 146.53, 129.01, 128.89, 121.54, 54.26, 41.09, 30.82, 25.47, 18.65.

^{19}F NMR: ($CDCl_3$, 471 MHz): δ -115.80.

FT-IR (Diamond-ATR, neat, cm^{-1}): 3442, 2948, 2864, 1618, 1517, 1165, 824.

HRMS (ES+): calc: $C_{13}H_{18}FN$ $[M+H]^+$: 208.2924.; found: 208.1502.

Synthesis of *N*-(5-fluoro-2-methylphenyl)naphthalen-1-amine (P209)

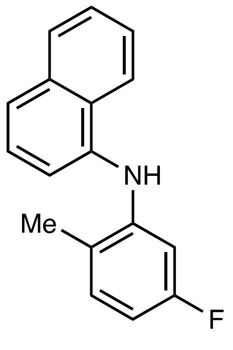

To an oven-dried and torched scintillation vial the reaction tube was equipped with a Teflon-coated magnetic stir bar. 2-Bromo-4-fluoro-1-methylbenzene was then added under a positive flow of nitrogen (2.0 mmol, 1.00 equiv). Naphthalen-1-amine was then added under a positive flow of nitrogen (2.4 mmol, 1.2 equiv). Under the same positive flow of nitrogen, the addition of NaO*t*-Bu (2.8 mmol, 1.40 equiv), and BrettPhos Pd Gen3 catalyst (0.06 mmol, 3 mol%) were added to the reaction vessel. The reaction tube was loosely capped with a screw-thread caps fitted with Teflon/SIL septa. The assembled reaction vessel was brought into a

nitrogen-filled glovebox, after which the cap was removed, and 4 mL of anhydrous toluene (PhMe) (>30ppm water by Karl Fisher Titrator™) was added *via* syringe. The reaction mixture was stirred open to the nitrogen-filled glovebox atmosphere for 30 min. After complete homogeneity occurred within the reaction vessel, the scintillation vial was tightly capped and brought out of the glovebox, and placed into an oil bath preheated to 100 °C. After stirring for 24 h at 100 °C, the reaction vessel was removed from the oil bath and allowed to cool to room temperature for 3 h. Then, the reaction solution was diluted with EtOAc (5mL), and the resulting suspension was filtered through a plug of celite anchored by a cotton plug. The plug of celite was washed with an additional EtOAc (4mL) and Dichloromethane (CH_2Cl_2) (4mL). The combined filtrates were concentrated under reduced pressure with the aid of a rotary evaporator and the crude residue was purified by automated column chromatography using SiliCycle™ prepacked Flash Cartridges. Purification yielded the C–N coupled product as a red oil in 93% isolated yield.

Chromatography Conditions: SiO_2 supported columns with a gradient of 0% to 90% EtOAc in Hexanes.

^{1}H NMR: (500 MHz, $CDCl_3$): δ 8.08 (d, J = 8.3 Hz, 1H), 7.99 (d, J = 7.9 Hz, 1H), 7.74 (d, J = 8.2 Hz, 1H), 7.64 – 7.55 (m, 2H), 7.52 (t, J = 7.8 Hz, 1H), 7.36 (d, J = 7.4 Hz, 1H), 7.26 – 7.19 (m, 1H), 6.72 – 6.62 (m, 2H), 5.81 (s, 1H), 2.40 (s, 3H).

^{13}C NMR: (126 MHz, $CDCl_3$): δ 163.32, 161.40, 145.06, 144.98, 138.13, 134.92, 131.57, 131.49, 128.77, 128.44, 126.39, 126.18, 126.14, 124.22, 122.09, 120.75, 120.73, 118.32, 106.70, 106.53, 103.40, 103.20, 17.26.

^{19}F NMR: ($CDCl_3$, 471 MHz): δ -115.33.

FT-IR (Diamond-ATR, neat, cm^{-1}): 3405, 3055, 1504, 1154, 785.

HRMS (ES+): calc: $C_{17}H_{14}FN$ [M+H]$^{+}$: 252.3044.; found: 252.1185.

Synthesis of 4-((cyclopentylmethyl)amino)-2-fluorobenzonitrile (P210)

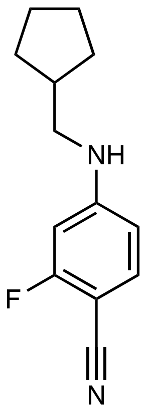

To an oven-dried and torched scintillation vial the reaction tube was equipped with a Teflon-coated magnetic stir bar. 4-Bromo-2-fluorobenzonitrile was then added under a positive flow of nitrogen (2.0 mmol, 1.00 equiv). Cyclopentylmethanamine was then added under a positive flow of nitrogen (2.4 mmol, 1.2 equiv). Under the same positive flow of nitrogen, the addition of NaO*t*-Bu (2.8 mmol, 1.40 equiv), and BrettPhos Pd Gen3 catalyst (0.06 mmol, 3 mol%) were added to the reaction vessel. The reaction tube was loosely capped with a screw-thread caps fitted with Teflon/SIL septa. The assembled reaction vessel was brought into a nitrogen-filled glovebox, after which the cap was removed, and 4 mL of anhydrous toluene (PhMe) (>30ppm water by Karl Fisher Titrator^{TM}) was added *via* syringe. The reaction mixture was stirred open to the nitrogen-filled glovebox atmosphere for 30 min. After complete homogeneity occurred within the reaction vessel, the scintillation vial was tightly capped and brought out of the glovebox, and placed into an oil bath preheated to 100 °C. After stirring for 24 h at 100 °C, the reaction vessel was removed from the oil bath and allowed to cool to room temperature for 3 h. Then, the reaction solution was diluted with EtOAc (5mL), and the resulting suspension was filtered through a plug of celite anchored by a cotton plug. The plug of celite was washed with an additional EtOAc (4mL) and Dichloromethane (CH_2Cl_2) (4mL). The combined filtrates were concentrated under reduced pressure with the aid of a rotary evaporator and the crude residue was purified by automated column chromatography using SiliCycle^{TM} prepacked Flash Cartridges. Purification yielded the C–N coupled product as an off-white solid in 73% isolated yield.

Chromatography Conditions: SiO_2 supported columns with a gradient of 0% to 90% CH_2Cl_2 in Hexanes.

^{1}H NMR: (500 MHz, $CDCl_3$): δ 7.29 (dd, J = 14.8, 6.4 Hz, 1H), 6.33 (dt, J = 11.7, 5.8 Hz, 1H), 6.27 (dd, J = 12.0, 2.1 Hz, 1H), 4.54 – 4.27 (m, 1H), 3.04 (dd, J = 7.2, 5.4 Hz, 2H), 2.24 – 2.07 (m, 1H), 1.90 – 1.76 (m, 2H), 1.72 – 1.56 (m, 4H), 1.25 (td, J = 14.2, 7.1 Hz, 2H).

^{13}C NMR: (126 MHz, $CDCl_3$): δ 166.16, 164.14, 153.75, 153.66, 133.95, 133.93, 115.77, 108.89, 98.35, 98.16, 87.24, 87.11, 48.66, 39.10, 30.55, 25.23, 25.20.

^{19}F NMR: ($CDCl_3$, 471 MHz) δ -106.22.

FT-IR (Diamond-ATR, neat, cm^{-1}): 3349, 2945, 2863, 2216, 1105, 807.

HRMS (ES+): calc: $C_{13}H_{15}FN_2$ [M+H] $^+$: 219.2754.; found: 219.1297.

Synthesis of (4-((cyclopentylmethyl)amino)phenyl)(phenyl)methanone (P211)

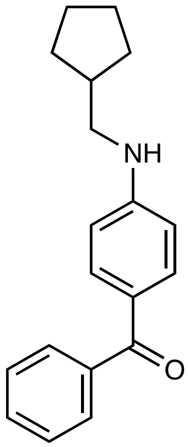

To an oven-dried and torched scintillation vial the reaction tube was equipped with a Teflon-coated magnetic stir bar. (4-Bromophenyl)(phenyl)methanone was then added under a positive flow of nitrogen (2.0 mmol, 1.00 equiv). Cyclopentylmethanamine was then added under a positive flow of nitrogen (2.4 mmol, 1.2 equiv). Under the same positive flow of nitrogen, the addition of NaO*t*-Bu (2.8 mmol, 1.40 equiv), and BrettPhos Pd Gen3 catalyst (0.06 mmol, 3 mol%) were added to the reaction vessel. The reaction tube was loosely capped with a screw-thread caps fitted with Teflon/SIL septa. The assembled reaction vessel was brought into a nitrogen-filled glovebox, after which the cap was removed, and 4 mL of anhydrous toluene (PhMe) (>30ppm water by Karl Fisher Titrator™) was added *via* syringe. The reaction mixture was stirred open to the nitrogen-filled glovebox atmosphere for 30 min. After complete homogeneity occurred within the reaction vessel, the scintillation vial was tightly capped and brought out of the glovebox, and placed into an oil bath preheated to 100 °C. After stirring for 24 h at 100 °C, the reaction vessel was removed from the oil bath and allowed to cool to room temperature for 3 h. Then, the reaction solution was diluted with EtOAc (5mL), and the resulting suspension was filtered through a plug of celite anchored by a cotton plug. The plug of celite was washed with an additional EtOAc (4mL) and Dichloromethane (CH_2Cl_2) (4mL). The combined filtrates were concentrated under reduced pressure with the aid of a rotary evaporator and the crude residue was purified by automated column chromatography using SiliCycle™ prepacked Flash Cartridges. Purification yielded the C–N coupled product as a brown solid in 53% isolated yield.

Chromatography Conditions: SiO_2 supported columns with a gradient of 5% to 50% CH_2Cl_2 in Hexanes.

^{1}H NMR: (500 MHz, $CDCl_3$): δ 7.62 (dd, J = 12.5, 8.0 Hz, 4H), 7.39 (q, J = 7.5 Hz, 1H), 7.35 – 7.28 (m, 2H), 6.47 (d, J = 8.7 Hz, 2H), 2.98 (d, J = 7.3 Hz, 2H), 2.11 – 1.98 (m, 1H), 1.69 (qd, J = 11.9, 5.4 Hz, 2H), 1.54 – 1.41 (m, 4H), 1.23 – 1.09 (m, 3H).

^{13}C NMR: (126 MHz, $CDCl_3$): δ 195.21, 152.55, 139.29, 133.09, 131.17, 129.45, 128.05, 111.22, 48.64, 39.35, 30.62, 25.27.

FT-IR (Diamond-ATR, neat, cm^{-1}): 3345, 2943, 1584, 1280, 1146, 937.

HRMS (ES+): calc: $C_{19}H_{21}NO$ $[M+H]^+$: 280.3830.; found: 280.1703.

Synthesis of phenyl(4-(pyridin-4-ylamino)phenyl)methanone (P212)

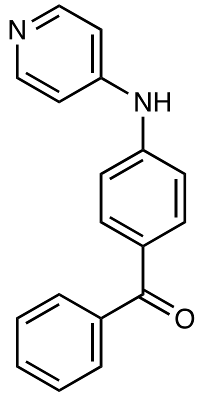

To an oven-dried and torched scintillation vial the reaction tube was equipped with a Teflon-coated magnetic stir bar. (4-Bromophenyl)(phenyl)methanone was then added under a positive flow of nitrogen (2.0 mmol, 1.00 equiv). Pyridin-4-amine was then added under a positive flow of nitrogen (2.4 mmol, 1.2 equiv). Under the same positive flow of nitrogen, the addition of NaO*t*-Bu (2.8 mmol, 1.40 equiv), and BrettPhos Pd Gen3 catalyst (0.06 mmol, 3 mol%) were added to the reaction vessel. The reaction tube was loosely capped with a screw-thread caps fitted with Teflon/SIL septa. The assembled reaction vessel was brought into a nitrogen-filled glovebox, after which the cap was removed, and 4 mL of anhydrous toluene (PhMe) (>30ppm water by Karl Fisher Titrator™) was added *via* syringe. The reaction mixture was stirred open to the nitrogen-filled glovebox atmosphere for 30 min. After complete homogeneity occurred within the reaction vessel, the scintillation vial was tightly capped and brought out of the glovebox, and placed into an oil bath preheated to 100 °C. After stirring for 24 h at 100 °C, the reaction vessel was removed from the oil bath and allowed to cool to room temperature for 3 h. Then, the reaction solution was diluted with EtOAc (5mL), and the resulting suspension was filtered through a plug of celite anchored by a cotton plug. The plug of celite was washed with an additional EtOAc (4mL) and Dichloromethane (CH_2Cl_2) (4mL). The combined filtrates were concentrated under reduced pressure with the aid of a rotary evaporator and the crude residue was purified by automated column chromatography using SiliCycle™ prepacked Flash Cartridges. Purification yielded the C–N coupled product as an off-white solid in 83% isolated yield.

Chromatography Conditions: SiO_2 supported columns with a gradient of 0% to 15% MeOH in CH_2Cl_2.

^{1}H NMR: (500 MHz, $CDCl_3$): δ 8.29 (d, J = 4.6 Hz, 1H), 7.75 (d, J = 8.6 Hz, 1H), 7.71 – 7.67 (m, 1H), 7.58 – 7.47 (m, 1H), 7.41 (q, J = 7.7 Hz, 1H), 7.18 (t, J = 6.6 Hz, 1H), 6.98 (dd, J = 24.7, 3.9 Hz, 1H).

^{13}C NMR: (126 MHz, $CDCl_3$): δ 195.43, 150.36, 144.73, 138.03, 132.32, 132.14, 131.52, 129.76, 128.30, 118.20, 111.17.

FT-IR (Diamond-ATR, neat, cm^{-1}): 3269, 3163, 2950, 1642, 1347, 1280, 997.

HRMS (ES+): calc: $C_{18}H_{14}N_2O$ $[M+H]^+$: 275.3230.; found: 275.1186.

Synthesis of 3,5-di-tert-butyl-N-(4-(trifluoromethyl)benzyl)aniline (P213)

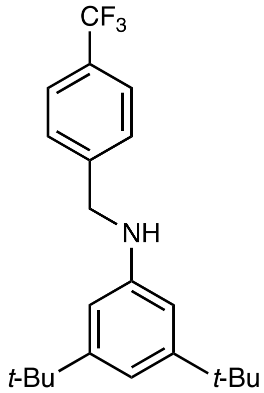

To an oven-dried and torched scintillation vial the reaction tube was equipped with a Teflon-coated magnetic stir bar. 1-Bromo-3,5-di-tert-butylbenzene was then added under a positive flow of nitrogen (2.0 mmol, 1.00 equiv). (4-(trifluoromethyl)phenyl)Methanamine was then added under a positive flow of nitrogen (2.4 mmol, 1.2 equiv). Under the same positive flow of nitrogen, the addition of NaO*t*-Bu (2.8 mmol, 1.40 equiv), and BrettPhos Pd Gen3 catalyst (0.06 mmol, 3 mol%) were added to the reaction vessel. The reaction tube was loosely capped with a screw-thread caps fitted with Teflon/SIL septa. The assembled reaction vessel was brought into a nitrogen-filled glovebox, after which the cap was removed, and 4 mL of anhydrous toluene (PhMe) (>30ppm water by Karl Fisher Titrator™) was added *via* syringe. The reaction mixture was stirred open to the nitrogen-filled glovebox atmosphere for 30 min. After complete homogeneity occurred within the reaction vessel, the scintillation vial was tightly capped and brought out of the glovebox, and placed into an oil bath preheated to 100 °C. After stirring for 24 h at 100 °C, the reaction vessel was removed from the oil bath and allowed to cool to room temperature for 3 h. Then, the reaction solution was diluted with EtOAc (5mL), and the resulting suspension was filtered through a plug of celite anchored by a cotton plug. The plug of celite was washed with an additional EtOAc (4mL) and Dichloromethane (CH_2Cl_2) (4mL). The combined filtrates were concentrated under reduced pressure with the aid of a rotary evaporator and the crude residue was purified by automated column chromatography using SiliCycle™ prepacked Flash Cartridges. Purification yielded the C–N coupled product as an off-white solid in 56% isolated yield.

Chromatography Conditions: SiO_2 supported columns with a gradient of 5% to 50% CH_2Cl_2 in Hexanes.

^{1}H NMR: (500 MHz, $CDCl_3$): δ 7.64 – 7.52 (m, 4H), 6.86 (s, 1H), 6.51 (d, J = 1.2 Hz, 2H), 4.42 (s, 2H), 1.29 (s, 18H).

^{13}C NMR: (126 MHz, $CDCl_3$): δ 151.89, 147.08, 144.06, 127.85, 125.54, 125.51, 112.86, 107.75, 48.44, 34.85, 31.42.

^{19}F NMR: ($CDCl_3$, 471 MHz): δ -62.40.

FT-IR (Diamond-ATR, neat, cm^{-1}): 3403, 2950, 2898, 1597, 1321, 1118, 1064, 848.

HRMS (ES+): calc: $C_{22}H_{28}F_3N$ $[M+H]^+$: 364.4682.; found: 364.2251.

Synthesis of *N*-(3,5-di-tert-butylphenyl)pyridin-4-amine (P214)

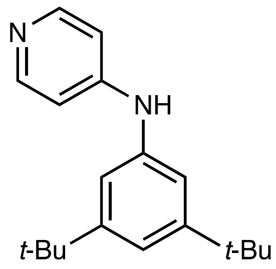

To an oven-dried and torched scintillation vial the reaction tube was equipped with a Teflon-coated magnetic stir bar. 1-Bromo-3,5-di-tert-butylbenzene was then added under a positive flow of nitrogen (2.0 mmol, 1.00 equiv). Pyridin-4-amine was then added under a positive flow of nitrogen (2.4 mmol, 1.2 equiv). Under the same positive flow of nitrogen, the addition of NaO*t*-Bu (2.8 mmol, 1.40 equiv), and BrettPhos Pd Gen3 catalyst (0.06 mmol, 3 mol%) were added to the reaction vessel. The reaction tube was loosely capped with a screw-thread caps fitted with Teflon/SIL septa. The assembled reaction vessel was brought into a nitrogen-filled glovebox, after which the cap was removed, and 4 mL of anhydrous toluene (PhMe) (>30ppm water by Karl Fisher Titrator^{TM}) was added *via* syringe. The reaction mixture was stirred open to the nitrogen-filled glovebox atmosphere for 30 min. After complete homogeneity occurred within the reaction vessel, the scintillation vial was tightly capped and brought out of the glovebox, and placed into an oil bath preheated to 100 °C. After stirring for 24 h at 100 °C, the reaction vessel was removed from the oil bath and allowed to cool to room temperature for 3 h. Then, the reaction solution was diluted with EtOAc (5mL), and the resulting suspension was filtered through a plug of celite anchored by a cotton plug. The plug of celite was washed with an additional EtOAc (4mL) and Dichloromethane (CH_2Cl_2) (4mL). The combined filtrates were concentrated under reduced pressure with the aid of a rotary evaporator and the crude residue was purified by automated column chromatography using SiliCycle^{TM} prepacked Flash Cartridges. Purification yielded the C–N coupled product as a white solid in 76% isolated yield.

Chromatography Conditions: SiO_2 supported columns with a gradient of 0% to 90% CH_2Cl_2 in Hexanes.

^{1}H NMR: (500 MHz, $CDCl_3$): δ 8.29 (d, J = 5.7 Hz, 2H), 7.23 (d, J = 1.5 Hz, 1H), 7.07 (d, J = 1.5 Hz, 2H), 6.83 (d, J = 6.1 Hz, 2H), 6.65 (s, 1H), 1.36 (s, 18H).

^{13}C NMR: (126 MHz, $CDCl_3$): δ 152.29, 150.12, 138.83, 118.41, 116.41, 109.27, 34.96, 31.41.

FT-IR (Diamond-ATR, neat, cm^{-1}): 3271, 2954, 2863, 1582, 1353, 997, 977.

HRMS (ES+): calc: $C_{19}H_{26}N_2$ $[M+H]^+$: 283.4310.; found: 283.2174.

Synthesis of *N*-cyclohexyl-4'-methyl-[1,1'-biphenyl]-4-amine (P215)

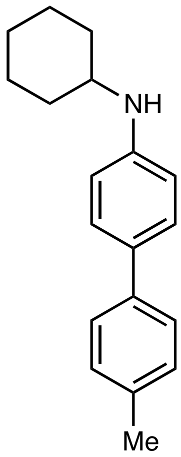

To an oven-dried and torched scintillation vial the reaction tube was equipped with a Teflon-coated magnetic stir bar. 4-Bromo-4'-methyl-1,1'-biphenyl was then added under a positive flow of nitrogen (2.0 mmol, 1.00 equiv). Cyclohexanamine was then added under a positive flow of nitrogen (2.4 mmol, 1.2 equiv). Under the same positive flow of nitrogen, the addition of NaO*t*-Bu (2.8 mmol, 1.40 equiv), and BrettPhos Pd Gen3 catalyst (0.06 mmol, 3 mol%) were added to the reaction vessel. The reaction tube was loosely capped with a screw-thread caps fitted with Teflon/SIL septa. The assembled reaction vessel was brought into a nitrogen-filled glovebox, after which the cap was removed, and 4 mL of anhydrous toluene (PhMe) (>30ppm water by Karl Fisher Titrator™) was added *via* syringe. The reaction mixture was stirred open to the nitrogen-filled glovebox atmosphere for 30 min. After complete homogeneity occurred within the reaction vessel, the scintillation vial was tightly capped and brought out of the glovebox, and placed into an oil bath preheated to 100 °C. After stirring for 24 h at 100 °C, the reaction vessel was removed from the oil bath and allowed to cool to room temperature for 3 h. Then, the reaction solution was diluted with EtOAc (5mL), and the resulting suspension was filtered through a plug of celite anchored by a cotton plug. The plug of celite was washed with an additional EtOAc (4mL) and Dichloromethane (CH_2Cl_2) (4mL). The combined filtrates were concentrated under reduced pressure with the aid of a rotary evaporator and the crude residue was purified by automated column chromatography using SiliCycle™ prepacked Flash Cartridges. Purification yielded the C–N coupled product as a white solid in 88% isolated yield.

Chromatography Conditions: SiO_2 supported columns with a gradient of 10% to 40% EtOAc in Hexanes.

^{1}H NMR: (500 MHz, $CDCl_3$): δ 7.50 (dd, J = 15.3, 8.1 Hz, 4H), 7.27 (d, J = 7.8 Hz, 2H), 6.72 (d, J = 8.5 Hz, 2H), 3.89 – 3.57 (s, 1H), 3.37 (dd, J = 12.0, 8.3 Hz, 1H), 2.45 (s, 3H), 2.15 (t, J = 12.2 Hz, 2H), 1.95 – 1.78 (m, 2H), 1.74 (dd, J = 9.2, 3.6 Hz, 1H), 1.54 – 1.39 (m, 2H), 1.28 (dtd, J = 24.8, 12.4, 3.3 Hz, 3H).

^{13}C NMR: (126 MHz, $CDCl_3$): δ 146.63, 138.58, 135.57, 129.83, 129.40, 127.82, 126.16, 113.43, 51.83, 33.54, 26.00, 25.08, 21.09.

FT-IR (Diamond-ATR, neat, cm^{-1}): 3394, 2928, 2853, 1606, 1500, 1103, 803.

HRMS (ES+): calc: $C_{19}H_{23}N$ $[M+H]^+$: 266.4000.; found: 266.1907.

Synthesis of 4'-methyl-*N*-(4-(trifluoromethyl)phenyl)-[1,1'-biphenyl]-4-amine (P216)

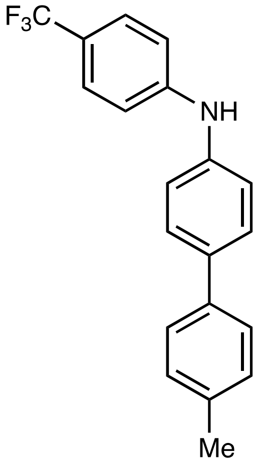

To an oven-dried and torched scintillation vial the reaction tube was equipped with a Teflon-coated magnetic stir bar. 4-Bromo-4'-methyl-1,1'-biphenyl was then added under a positive flow of nitrogen (2.0 mmol, 1.00 equiv). 4-(trifluoromethyl)Aniline was then added under a positive flow of nitrogen (2.4 mmol, 1.2 equiv). Under the same positive flow of nitrogen, the addition of NaO*t*-Bu (2.8 mmol, 1.40 equiv), and BrettPhos Pd Gen3 catalyst (0.06 mmol, 3 mol%) were added to the reaction vessel. The reaction tube was loosely capped with a screw-thread caps fitted with Teflon/SIL septa. The assembled reaction vessel was brought into a nitrogen-filled glovebox, after which the cap was removed, and 4 mL of anhydrous toluene (PhMe) (>30ppm water by Karl Fisher Titrator^{TM}) was added *via* syringe. The reaction mixture was stirred open to the nitrogen-filled glovebox atmosphere for 30 min. After complete homogeneity occurred within the reaction vessel, the scintillation vial was tightly capped and brought out of the glovebox, and placed into an oil bath preheated to 100 °C. After stirring for 24 h at 100 °C, the reaction vessel was removed from the oil bath and allowed to cool to room temperature for 3 h. Then, the reaction solution was diluted with EtOAc (5mL), and the resulting suspension was filtered through a plug of celite anchored by a cotton plug. The plug of celite was washed with an additional EtOAc (4mL) and Dichloromethane (CH_2Cl_2) (4mL). The combined filtrates were concentrated under reduced pressure with the aid of a rotary evaporator and the crude residue was purified by automated

column chromatography using SiliCycle™ prepacked Flash Cartridges. Purification yielded the C–N coupled product as a white solid in 68% isolated yield.

Chromatography Conditions: SiO_2 supported columns with a gradient of 10% to 40% EtOAc in Hexanes.

^{1}H NMR: (500 MHz, $CDCl_3$): δ 7.55 (d, J = 8.5 Hz, 2H), 7.52 – 7.45 (m, 4H), 7.23 (d, J = 11.1 Hz, 2H), 7.19 (t, J = 10.1 Hz, 2H), 7.07 (t, J = 10.5 Hz, 2H), 6.04 – 5.85 (m, 1H), 2.40 (s, 3H).

^{13}C NMR: (126 MHz, $CDCl_3$): δ 146.67, 140.20, 137.66, 136.77, 135.80, 129.54, 127.95, 126.76, 126.54, 120.19, 115.48, 21.08.

^{19}F NMR: ($CDCl_3$, 471 MHz): δ -61.47.

FT-IR (Diamond-ATR, neat, cm^{-1}): 3410, 1601, 1319, 1161, 1100, 1064, 803.

HRMS (ES+): calc: $C_{20}H_{16}F_3N$ $[M+H]^+$: 328.3502.; found: 328.1315.

Synthesis of 6-butoxy-*N*-(4-(trifluoromethyl)phenyl)naphthalen-2-amine (P217)

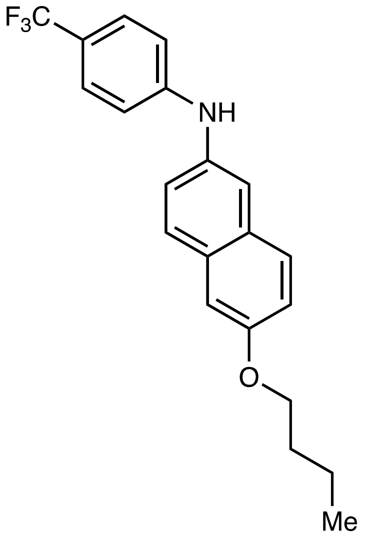

To an oven-dried and torched scintillation vial the reaction tube was equipped with a Teflon-coated magnetic stir bar. 2-Bromo-6-butoxynaphthalene was then added under a positive flow of nitrogen (2.0 mmol, 1.00 equiv). 4-(trifluoromethyl)Aniline was then added under a positive flow of nitrogen (2.4 mmol, 1.2 equiv). Under the same positive flow of nitrogen, the addition of NaO*t*-Bu (2.8 mmol, 1.40 equiv), and BrettPhos Pd Gen3 catalyst (0.06 mmol, 3 mol%) were added to the reaction vessel. The reaction tube was loosely capped with a screw-thread caps fitted with Teflon/SIL septa. The assembled reaction vessel was brought into a nitrogen-filled glovebox, after which the cap was removed, and 4 mL of anhydrous toluene (PhMe) (>30ppm water by Karl Fisher Titrator™) was added *via* syringe. The reaction mixture was stirred open to the nitrogen-filled glovebox atmosphere for 30 min. After complete homogeneity occurred within the reaction vessel, the scintillation vial was tightly capped and brought out of the glovebox, and placed into an oil bath preheated to 100 °C. After stirring for 24 h at 100 °C, the reaction vessel was removed from the oil bath and allowed to cool to room temperature for 3 h.

Then, the reaction solution was diluted with EtOAc (5mL), and the resulting suspension was filtered through a plug of celite anchored by a cotton plug. The plug of celite was washed with an additional EtOAc (4mL) and Dichloromethane (CH_2Cl_2) (4mL). The combined filtrates were concentrated under reduced pressure with the aid of a rotary evaporator and the crude residue was purified by automated column chromatography using SiliCycle™ prepacked Flash Cartridges. Purification yielded the C–N coupled product as a brown solid in 31% isolated yield.

Chromatography Conditions: SiO_2 supported columns with a gradient of 5% to 50% EtOAc in Hexanes.

^{1}H NMR: (500 MHz, $CDCl_3$): δ 7.74 (d, J = 8.7 Hz, 1H), 7.66 (d, J = 8.9 Hz, 1H), 7.56 – 7.48 (m, 3H), 7.29 (dd, J = 8.7, 2.2 Hz, 1H), 7.22 (dt, J = 11.2, 5.6 Hz, 1H), 7.18 (d, J = 2.1 Hz, 1H), 7.06 (d, J = 8.5 Hz, 2H), 5.97 (s, 1H), 4.13 (t, J = 6.5 Hz, 2H), 1.98 – 1.83 (m, 2H), 1.75 – 1.56 (m, 2H), 1.09 (t, J = 7.4 Hz, 3H).

^{13}C NMR: (126 MHz, $CDCl_3$): δ 156.55, 147.42, 136.58, 131.44, 129.58, 128.37, 128.15, 126.80, 126.77, 126.74, 126.71, 122.19, 119.80, 116.85, 114.98, 106.87, 67.87, 31.39, 19.38, 13.93.

^{19}F NMR: ($CDCl_3$, 471 MHz): δ -61.22.

FT-IR (Diamond-ATR, neat, cm^{-1}): 3424, 2939, 2878, 1605, 1528, 1329, 1312, 1161, 1070.

HRMS (ES+): calc: $C_{21}H_{20}F_3NO$ $[M+H]^+$: 360.3922.; found: 360.1568.

Synthesis of *N*-cyclohexyl-9H-fluoren-2-amine (P218)

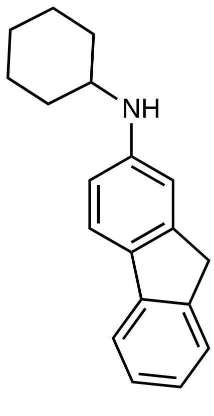

To an oven-dried and torched scintillation vial the reaction tube was equipped with a Teflon-coated magnetic stir bar. 2-Bromo-9H-fluorene was then added under a positive flow of nitrogen (2.0 mmol, 1.00 equiv). Cyclohexanamine was then added under a positive flow of nitrogen (2.4 mmol, 1.2 equiv). Under the same positive flow of nitrogen, the addition of NaO*t*-Bu (2.8 mmol, 1.40 equiv), and BrettPhos Pd Gen3 catalyst (0.06 mmol, 3 mol%) were added to the reaction vessel. The reaction tube was loosely capped with a screw-thread caps fitted with Teflon/SIL septa. The assembled reaction vessel was brought into a nitrogen-filled glovebox, after which the cap was removed, and 4 mL of anhydrous toluene (PhMe) (>30ppm water by Karl Fisher Titrator™) was added *via* syringe. The reaction mixture was stirred open to

the nitrogen-filled glovebox atmosphere for 30 min. After complete homogeneity occurred within the reaction vessel, the scintillation vial was tightly capped and brought out of the glovebox, and placed into an oil bath preheated to 100 °C. After stirring for 24 h at 100 °C, the reaction vessel was removed from the oil bath and allowed to cool to room temperature for 3 h. Then, the reaction solution was diluted with EtOAc (5mL), and the resulting suspension was filtered through a plug of celite anchored by a cotton plug. The plug of celite was washed with an additional EtOAc (4mL) and Dichloromethane (CH_2Cl_2) (4mL). The combined filtrates were concentrated under reduced pressure with the aid of a rotary evaporator and the crude residue was purified by automated column chromatography using SiliCycle™ prepacked Flash Cartridges. Purification yielded the C–N coupled product as a pink solid in 32% isolated yield.

Chromatography Conditions: SiO_2 supported columns with a gradient of 5% to 50% EtOAc in Hexanes.

^{1}H NMR: (500 MHz, $CDCl_3$): δ 7.62 (dd, J = 13.9, 6.0 Hz, 1H), 7.59 (d, J = 8.2 Hz, 1H), 7.47 (t, J = 12.9 Hz, 1H), 7.32 (q, J = 7.6 Hz, 1H), 7.19 (t, J = 7.3 Hz, 1H), 6.83 (s, 1H), 6.65 (d, J = 7.8 Hz, 1H), 3.84 (s, 2H), 3.42 – 3.28 (m, 1H), 2.21 – 2.09 (m, 2H), 1.89 – 1.76 (m, 2H), 1.77 – 1.64 (m, 1H), 1.52 – 1.36 (m, 2H), 1.36 – 1.16 (m, 3H).

^{13}C NMR: (126 MHz, $CDCl_3$): δ 146.80, 145.24, 142.46, 142.15, 131.59, 126.59, 124.66, 120.68, 118.33, 112.52, 109.62, 52.14, 36.96, 33.52, 25.96, 25.07.

FT-IR (Diamond-ATR, neat, cm^{-1}): 3386, 2922, 2853, 1618, 1454, 1403, 1318, 1135.

HRMS (ES+): calc: $C_{19}H_{21}N$ $[M+H]^+$: 264.3840.; found: 264.1751.

Synthesis of *N*-(4-(trifluoromethyl)phenyl)-9H-fluoren-2-amine (P219)

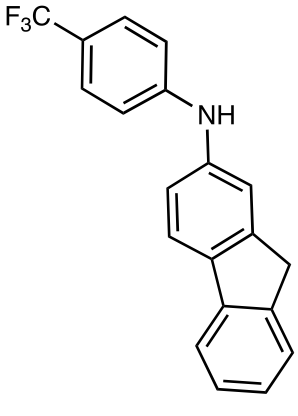

To an oven-dried and torched scintillation vial the reaction tube was equipped with a Teflon-coated magnetic stir bar. 2-Bromo-9H-fluorene was then added under a positive flow of nitrogen (2.0 mmol, 1.00 equiv). 4-(trifluoromethyl)Aniline was then added under a positive flow of nitrogen (2.4 mmol, 1.2 equiv). Under the same positive flow of nitrogen, the addition of NaO*t*-Bu (2.8 mmol, 1.40 equiv), and BrettPhos Pd Gen3 catalyst (0.06 mmol, 3 mol%) were added to the reaction vessel. The reaction tube was loosely capped with a screw-thread caps fitted with Teflon/SIL septa. The

assembled reaction vessel was brought into a nitrogen-filled glovebox, after which the cap was removed, and 4 mL of anhydrous toluene (PhMe) (>30ppm water by Karl Fisher Titrator™) was added *via* syringe. The reaction mixture was stirred open to the nitrogen-filled glovebox atmosphere for 30 min. After complete homogeneity occurred within the reaction vessel, the scintillation vial was tightly capped and brought out of the glovebox, and placed into an oil bath preheated to 100 °C. After stirring for 24 h at 100 °C, the reaction vessel was removed from the oil bath and allowed to cool to room temperature for 3 h. Then, the reaction solution was diluted with EtOAc (5mL), and the resulting suspension was filtered through a plug of celite anchored by a cotton plug. The plug of celite was washed with an additional EtOAc (4mL) and Dichloromethane (CH_2Cl_2) (4mL). The combined filtrates were concentrated under reduced pressure with the aid of a rotary evaporator and the crude residue was purified by automated column chromatography using SiliCycle™ prepacked Flash Cartridges. Purification yielded the C–N coupled product as a brown solid in 32% isolated yield.

Chromatography Conditions: SiO_2 supported columns with a gradient of 5% to 50% EtOAc in Hexanes.

^{1}H NMR: (500 MHz, $CDCl_3$): δ 7.81 – 7.71 (m, 2H), 7.58 (t, J = 11.4 Hz, 1H), 7.53 (d, J = 8.5 Hz, 2H), 7.42 (t, J = 7.4 Hz, 1H), 7.36 (d, J = 9.8 Hz, 1H), 7.32 (dd, J = 13.4, 6.0 Hz, 1H), 7.17 (t, J = 15.2 Hz, 1H), 7.08 (d, J = 8.4 Hz, 2H), 6.21 – 5.84 (m, 1H), 3.91 (s, 2H).

^{13}C NMR: (126 MHz, $CDCl_3$): δ 147.12, 144.96, 142.89, 141.43, 140.04, 126.89, 126.77, 126.74, 126.19, 125.00, 120.73, 119.37, 117.05, 115.30, 36.96.

^{19}F NMR: ($CDCl_3$, 471 MHz): δ -61.34.

FT-IR (Diamond-ATR, neat, cm^{-1}): 3403, 1608, 1526, 1318, 1094, 1066, 826.

HRMS (ES+): calc: $C_{20}H_{14}F_3N$ $[M+H]^+$: 326.3342.; found: 326.1153.

Synthesis of *N*-(4-(1,3-dioxolan-2-yl)phenyl)-1-benzylpiperidin-4-amine (P300)

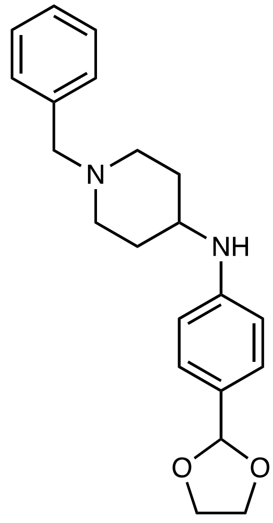

To an oven-dried and torched scintillation vial the reaction tube was equipped with a Teflon-coated magnetic stir bar. 2-(4-Bromophenyl)-1,3-dioxolane then was added under a positive flow of nitrogen (2.0 mmol, 1.00 equiv). 1-Benzylpiperidin-4-amine was then added under a positive flow of nitrogen (2.4 mmol, 1.2 equiv). Under the same positive flow of nitrogen, the addition of NaO*t*-Bu (2.8 mmol, 1.40 equiv), and BrettPhos Pd Gen3 catalyst (0.06 mmol, 3 mol%) were added to the reaction vessel. The reaction tube was loosely capped with a screw-thread caps fitted with Teflon/SIL septa. The assembled reaction vessel was brought into a nitrogen-filled glovebox, after which the cap was removed, and 4 mL of anhydrous toluene (PhMe) (>30ppm water by Karl Fisher Titrator™) was added *via* syringe. The reaction mixture was stirred open to the nitrogen-filled glovebox atmosphere for 30 min. After complete homogeneity occurred within the reaction vessel, the scintillation vial was tightly capped and brought out of the glovebox, and placed into an oil bath preheated to 100 °C. After stirring for 24 h at 100 °C, the reaction vessel was removed from the oil bath and allowed to cool to room temperature for 3 h. Then, the reaction solution was diluted with EtOAc (5mL), and the resulting suspension was filtered through a plug of celite anchored by a cotton plug. The plug of celite was washed with an additional EtOAc (4mL) and Dichloromethane (CH_2Cl_2) (4mL). The combined filtrates were concentrated under reduced pressure with the aid of a rotary evaporator and the crude residue was purified by automated column chromatography using SiliCycle™ prepacked Flash Cartridges. Purification yielded the C–N coupled product as a yellow solid in 67% isolated yield.

Chromatography Conditions: SiO_2 supported columns with a gradient of 0% to 70% EtOAc in Hexanes.

^{1}H NMR: (500 MHz, $CDCl_3$): δ 7.21 (t, J = 6.4 Hz, 4H), 7.19 – 7.13 (m, 3H), 6.46 (d, J = 8.5 Hz, 2H), 5.59 (s, 1H), 4.04 – 3.93 (m, 2H), 3.91 – 3.82 (m, 2H), 3.54 (d, J = 7.9 Hz, 1H), 3.42 (s, 2H), 3.18 (dd, J = 19.9, 16.3 Hz, 1H), 2.73 (d, J = 11.6 Hz, 2H), 2.04 (t, J = 10.7 Hz, 2H), 1.96 – 1.85 (m, 2H), 1.43 – 1.27 (m, 2H).

^{13}C NMR: (126 MHz, $CDCl_3$): δ 148.12, 138.48, 129.16, 128.25, 127.84, 127.06, 126.00, 112.85, 104.26, 65.17, 63.18, 52.35, 49.89, 32.48.

FT-IR (Diamond-ATR, neat, cm^{-1}): 3381, 3342, 2879, 1614, 1523, 1424, 1310, 1267, 1070.

HRMS (ES+): calc: $C_{21}H_{26}N_2O_2$ [M+H]$^+$: 339.4510.; found: 339.2074.

Synthesis of *N*-(benzo[d][1,3]dioxol-5-yl)pyrimidin-2-amine (P301)

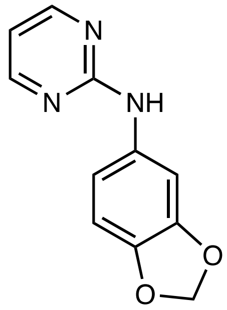

To an oven-dried and torched scintillation vial the reaction tube was equipped with a Teflon-coated magnetic stir bar. 5-Bromobenzo[d][1,3]dioxole was then added under a positive flow of nitrogen (2.0 mmol, 1.00 equiv). 1- Pyrimidin-2-amine was then added under a positive flow of nitrogen (2.4 mmol, 1.2 equiv). Under the same positive flow of nitrogen, the addition of NaO*t*-Bu (2.8 mmol, 1.40 equiv), and BrettPhos Pd Gen3 catalyst (0.06 mmol, 3 mol%) were added to the reaction vessel. The reaction tube was loosely capped with a screw-thread caps fitted with Teflon/SIL septa. The assembled reaction vessel was brought into a nitrogen-filled glovebox, after which the cap was removed, and 4 mL of anhydrous toluene (PhMe) (>30ppm water by Karl Fisher Titrator^{TM}) was added *via* syringe. The reaction mixture was stirred open to the nitrogen-filled glovebox atmosphere for 30 min. After complete homogeneity occurred within the reaction vessel, the scintillation vial was tightly capped and brought out of the glovebox, and placed into an oil bath preheated to 100 °C. After stirring for 24 h at 100 °C, the reaction vessel was removed from the oil bath and allowed to cool to room temperature for 3 h. Then, the reaction solution was diluted with EtOAc (5mL), and the resulting suspension was filtered through a plug of celite anchored by a cotton plug. The plug of celite was washed with an additional EtOAc (4mL) and Dichloromethane (CH_2Cl_2) (4mL). The combined filtrates were concentrated under reduced pressure with the aid of a rotary evaporator and the crude residue was purified by automated column chromatography using SiliCycle^{TM} prepacked Flash Cartridges. Purification yielded the C–N coupled product as an off-white solid in 47% isolated yield.

Chromatography Conditions: SiO_2 supported columns with a gradient of 0% to 30% CH_2Cl_2 in Hexanes.

^{1}H NMR: (500 MHz, $CDCl_3$): δ 8.38 (d, J = 4.8 Hz, 2H), 7.31 (d, J = 2.0 Hz, 1H), 7.19 (s, 1H), 6.86 (dd, J = 8.3, 2.0 Hz, 1H), 6.77 (d, J = 8.3 Hz, 1H), 6.67 (t, J = 4.8 Hz, 1H), 5.95 (s, 2H).

^{13}C NMR: (126 MHz, $CDCl_3$): δ 160.47, 158.04, 147.82, 143.53, 133.56, 113.33, 112.25, 108.14, 103.18, 101.15.

FT-IR (Diamond-ATR, neat, cm^{-1}): 3261, 3014, 1413, 1239, 1187, 1038, 936.

HRMS (ES+): calc: $C_{11}H_9N_3O_2$ $[M+H]^+$: 216.2120.; found: 216.0775.

NMR Spectra of C–N Coupling Products

^{1}H NMR ($CDCl_3$, 500 MHz) of P100

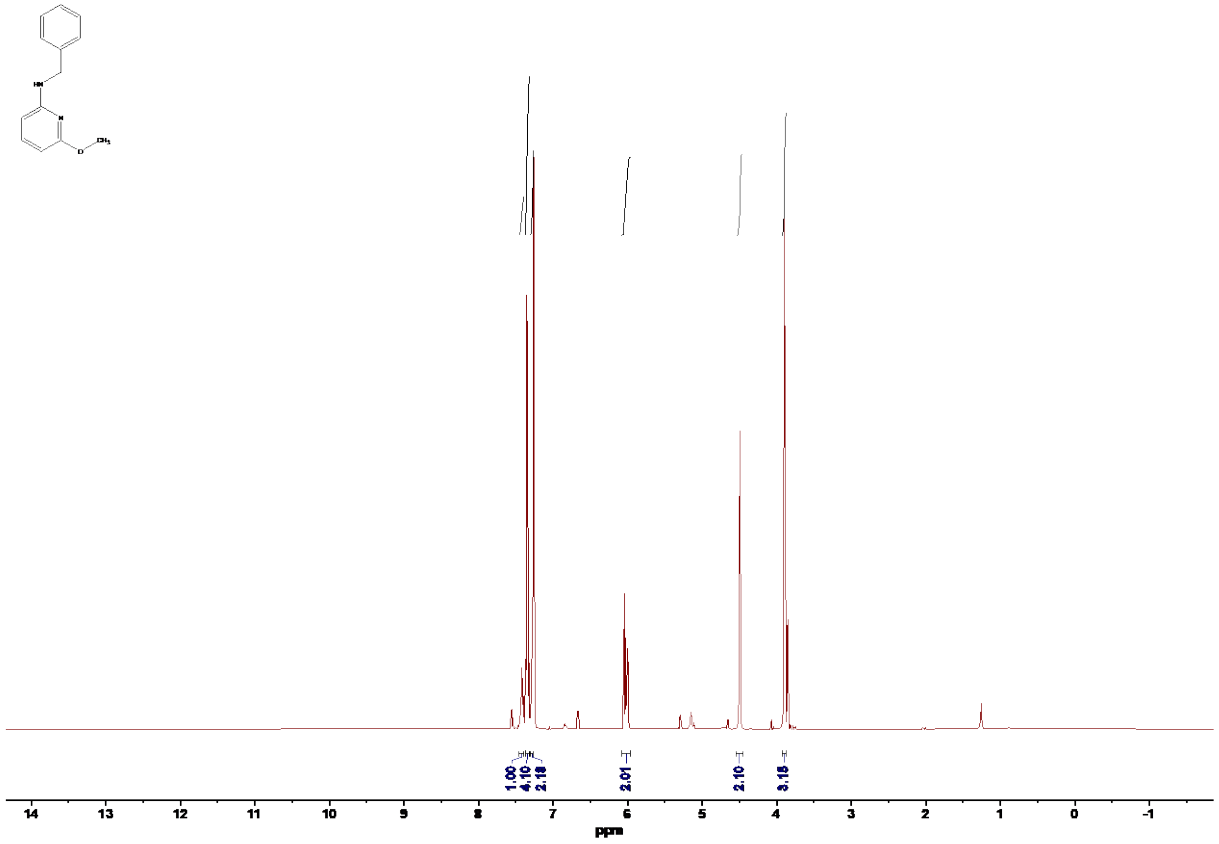

^{1}H NMR ($CDCl_3$, 500 MHz) of P101

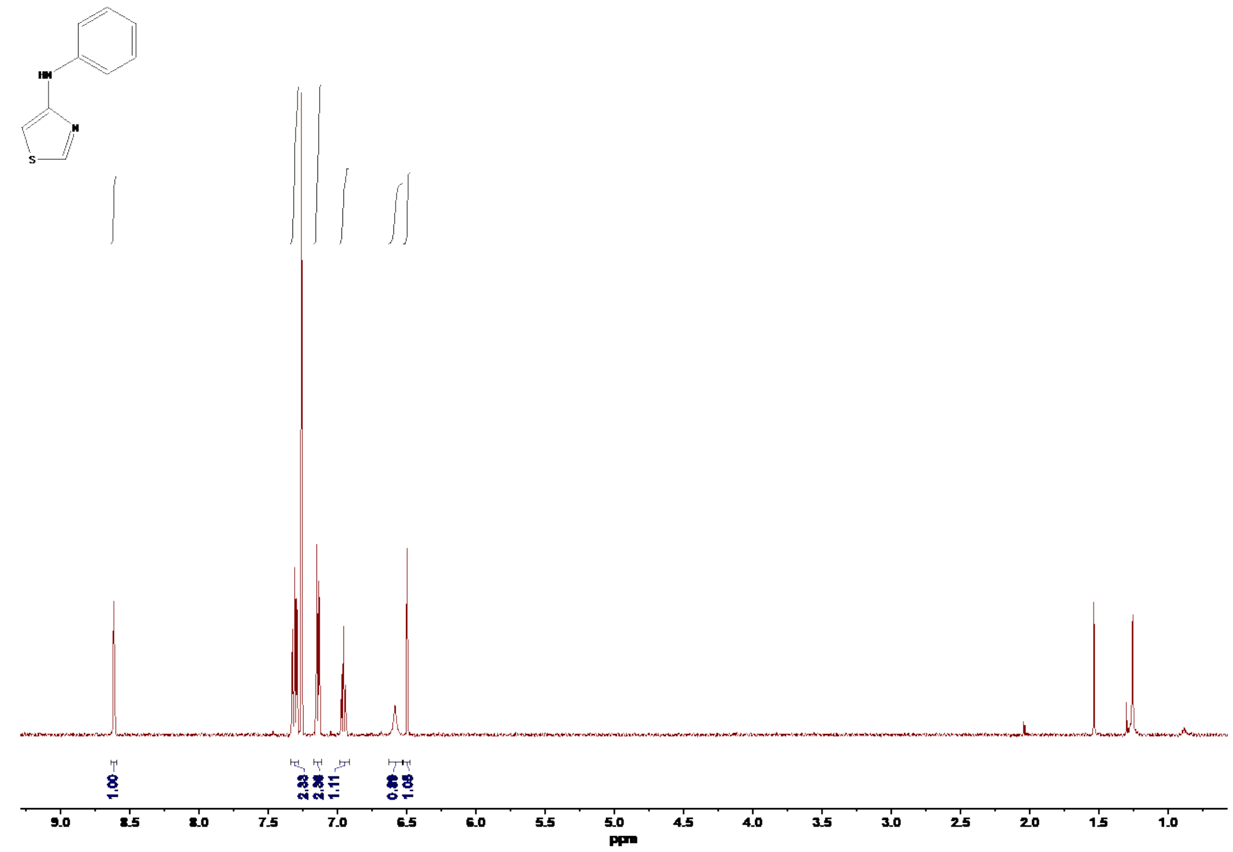

^{1}H NMR ($CDCl_3$, 500 MHz) of P102

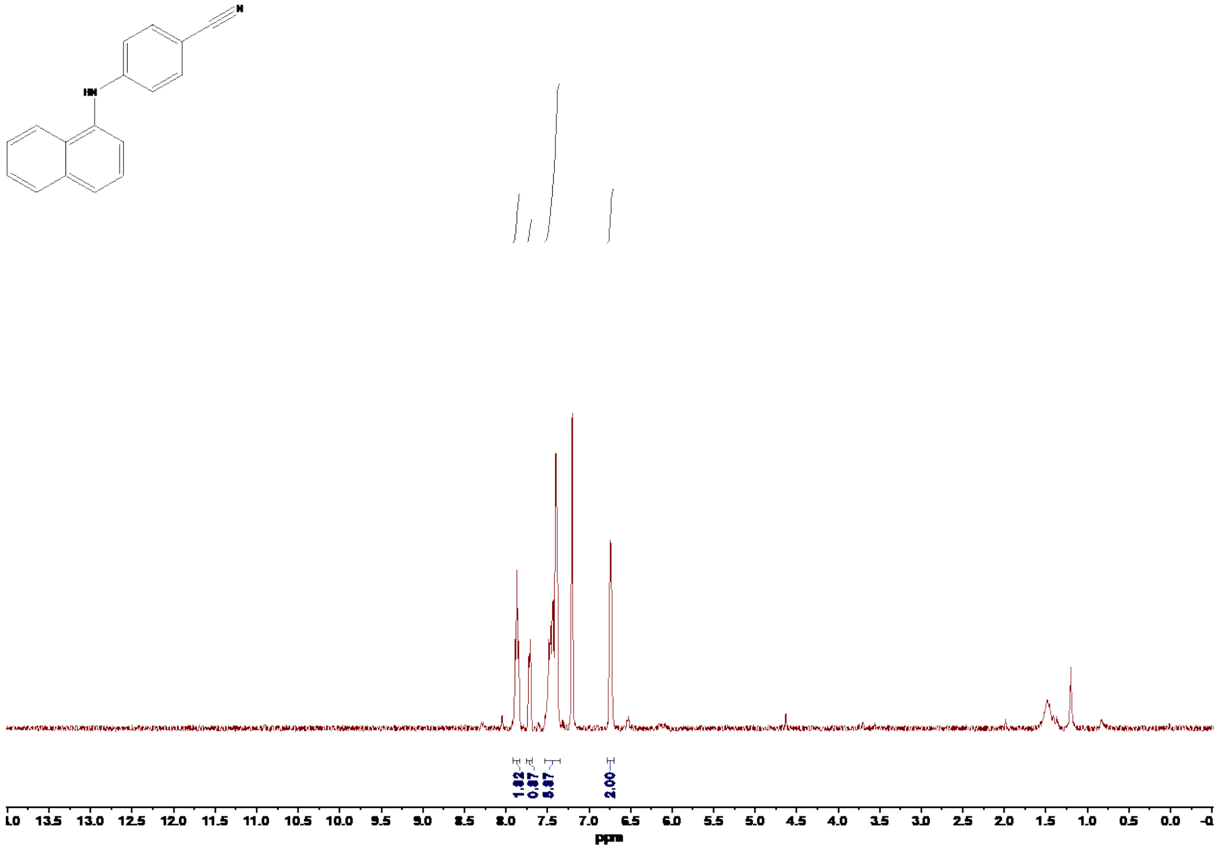

^{1}H NMR ($CDCl_3$, 500 MHz) of P106

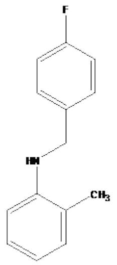
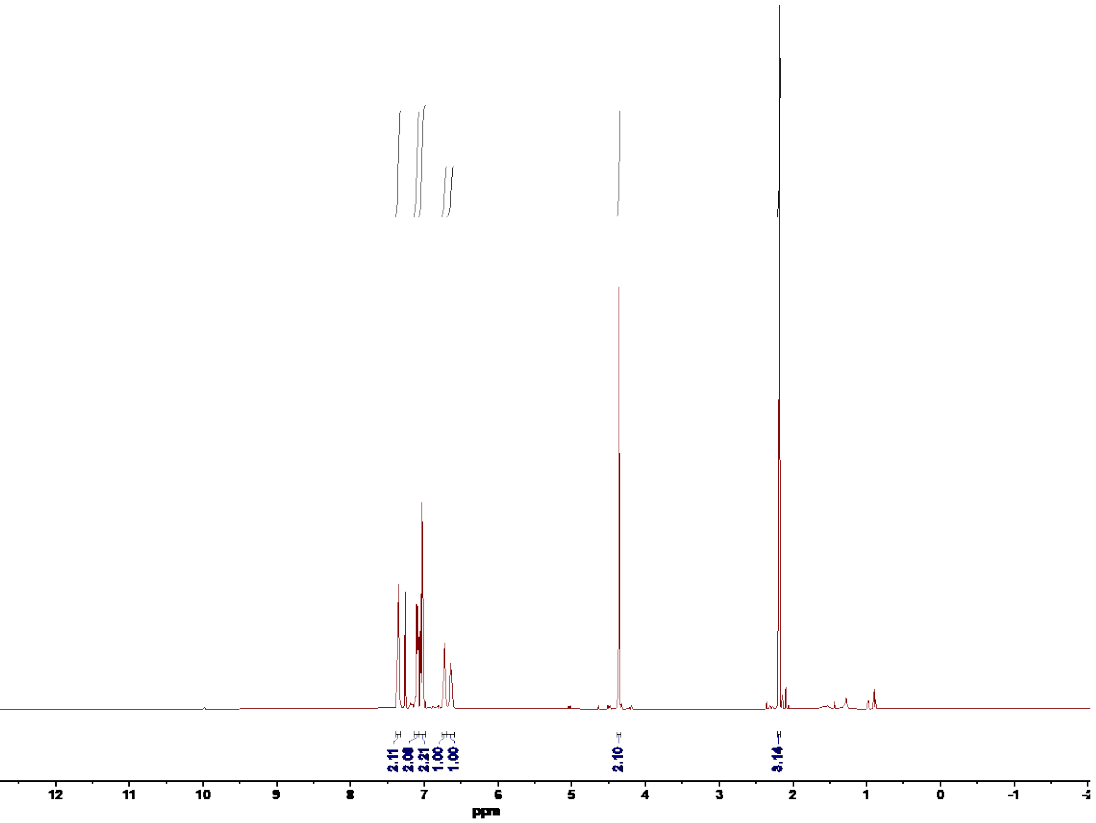

^{1}H NMR ($CDCl_3$, 500 MHz) of P107

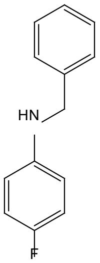

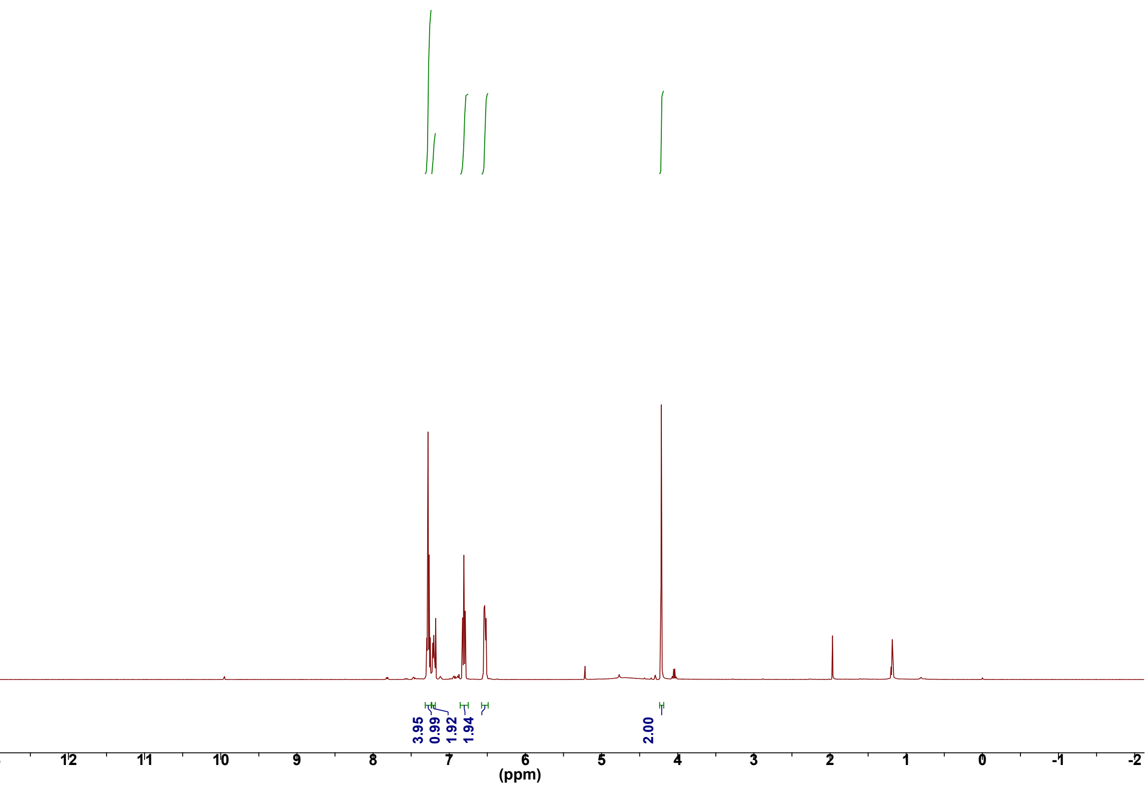

^{1}H NMR ($CDCl_3$, 500 MHz) of P112

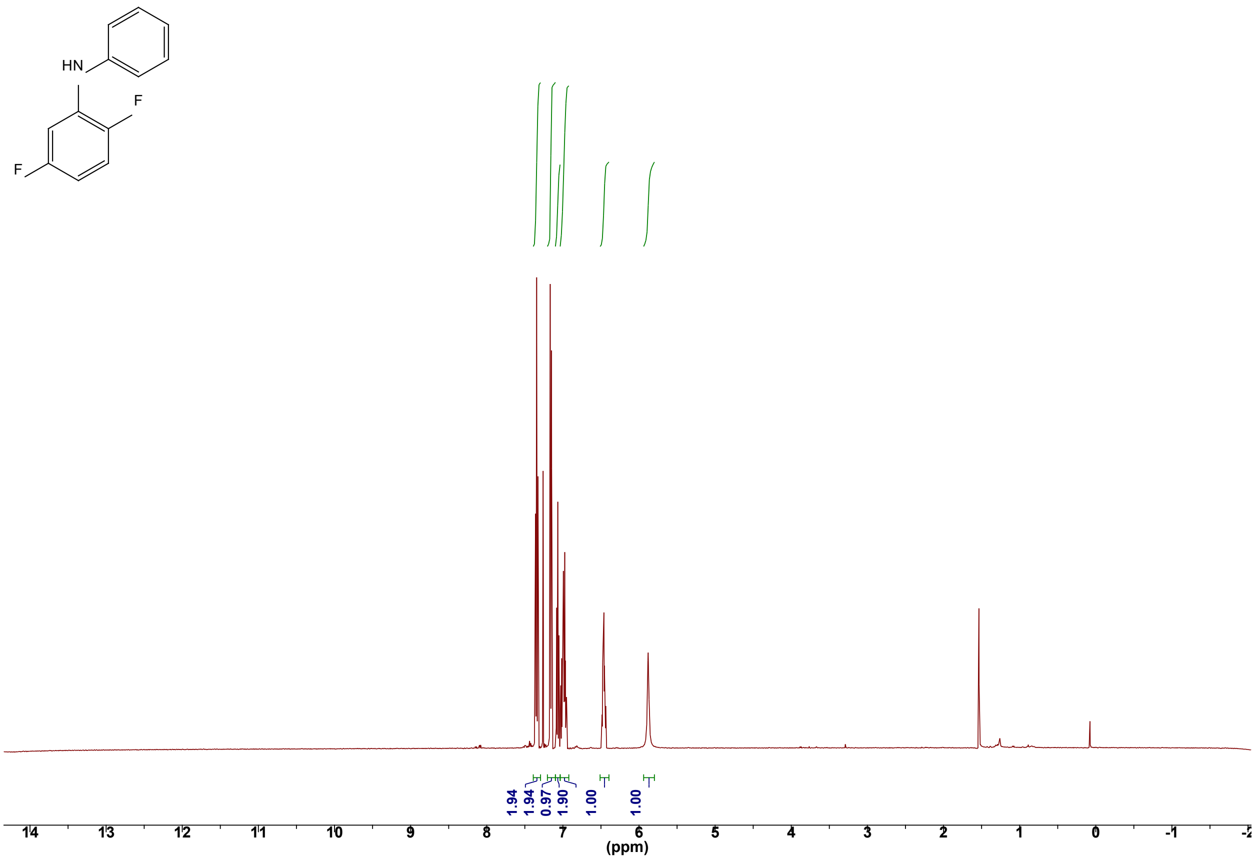

^{13}C NMR ($CDCl_3$, 126 MHz) of P112

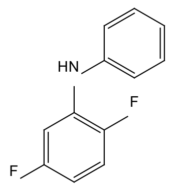

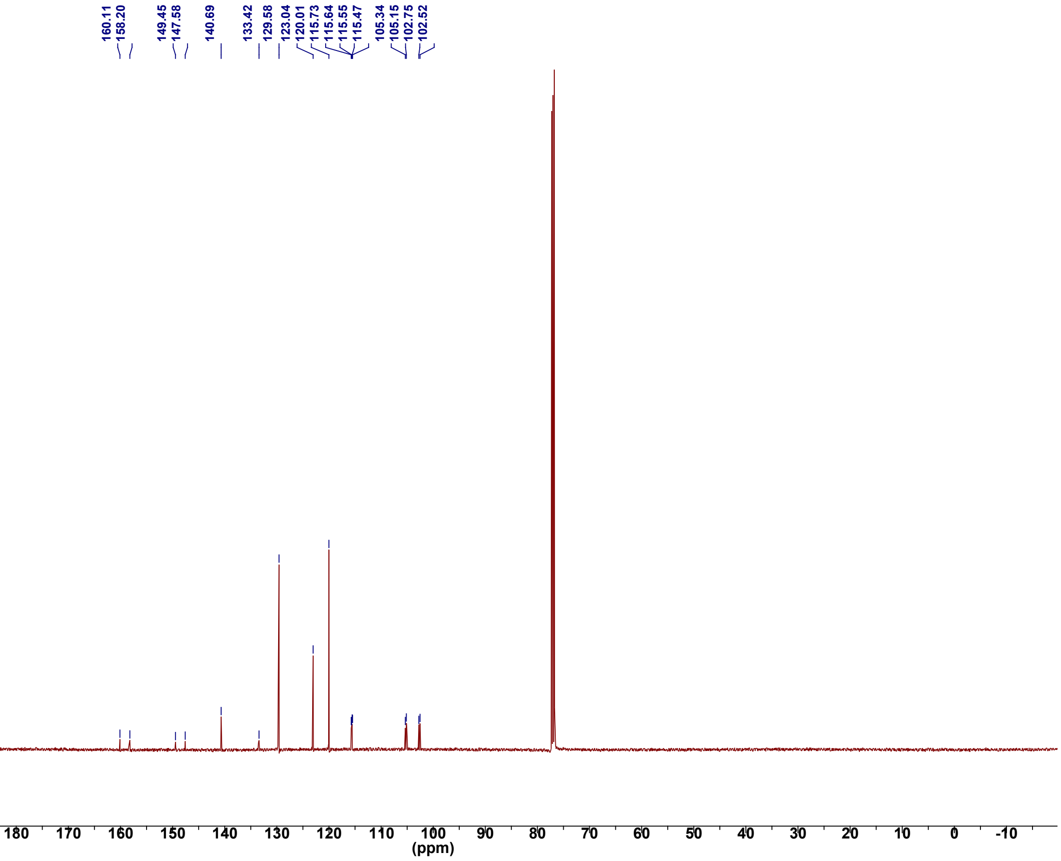

^{19}F NMR ($CDCl_3$, 471 MHz) of P112

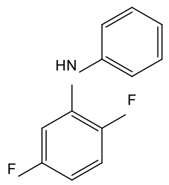

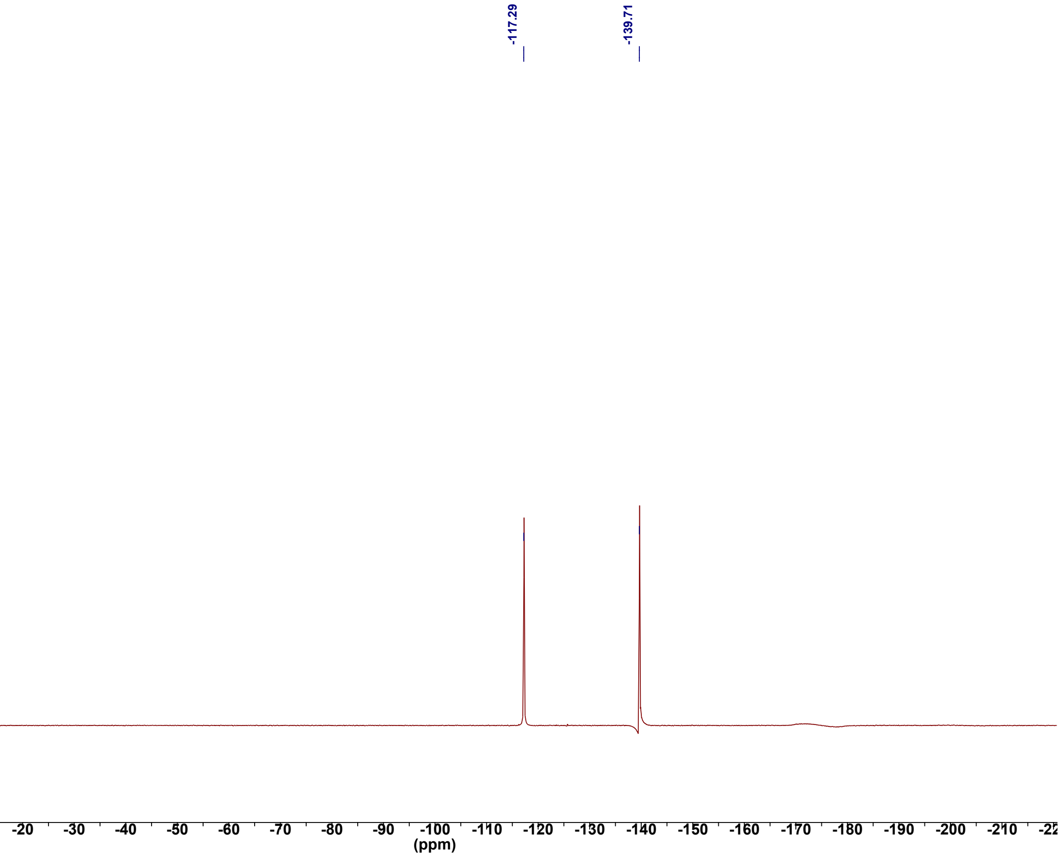

^{1}H NMR (CDCl$_3$, 500 MHz) of P113

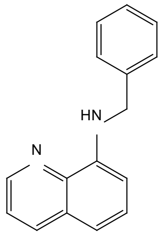

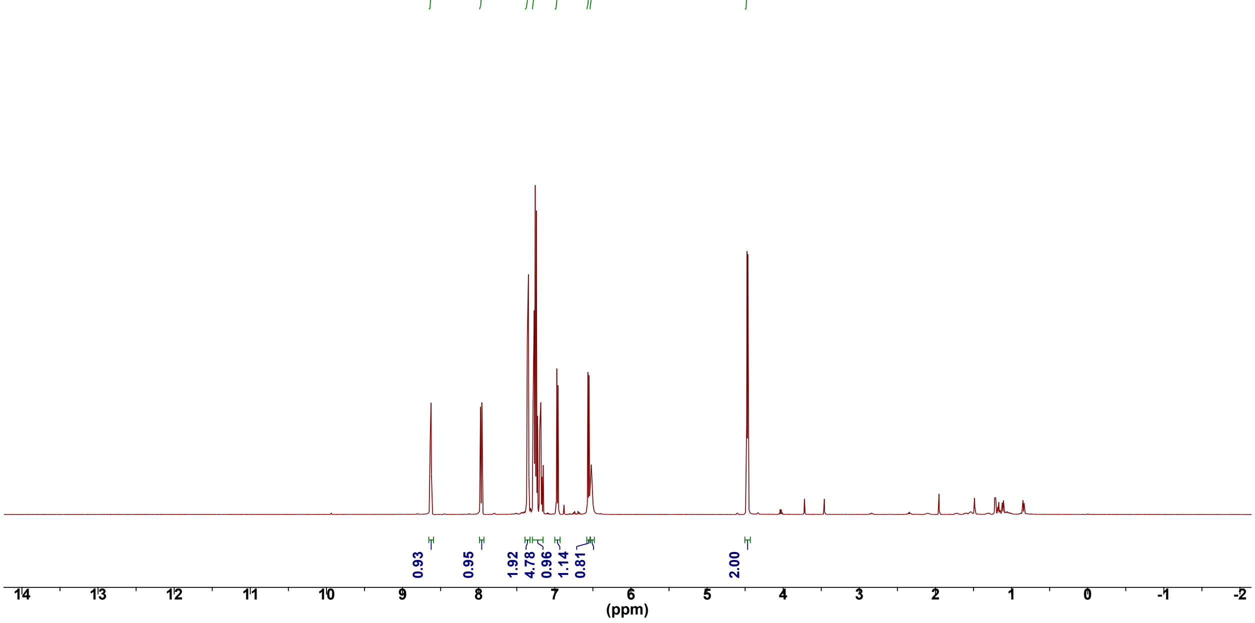

^{13}C NMR ($CDCl_3$, 126 MHz) of P113

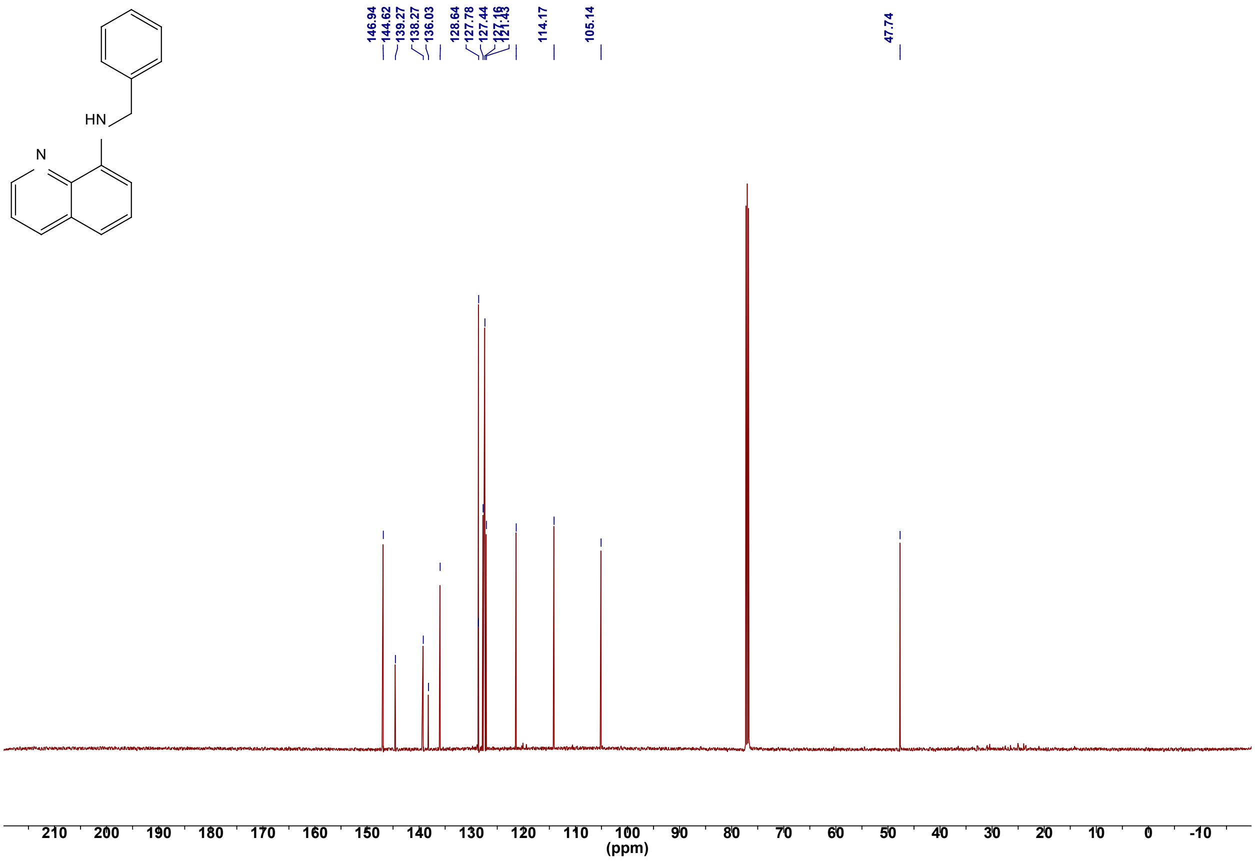

^1H NMR ($CDCl_3$, 500 MHz) of P114

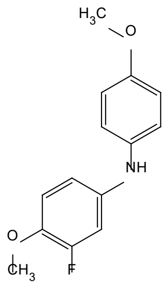

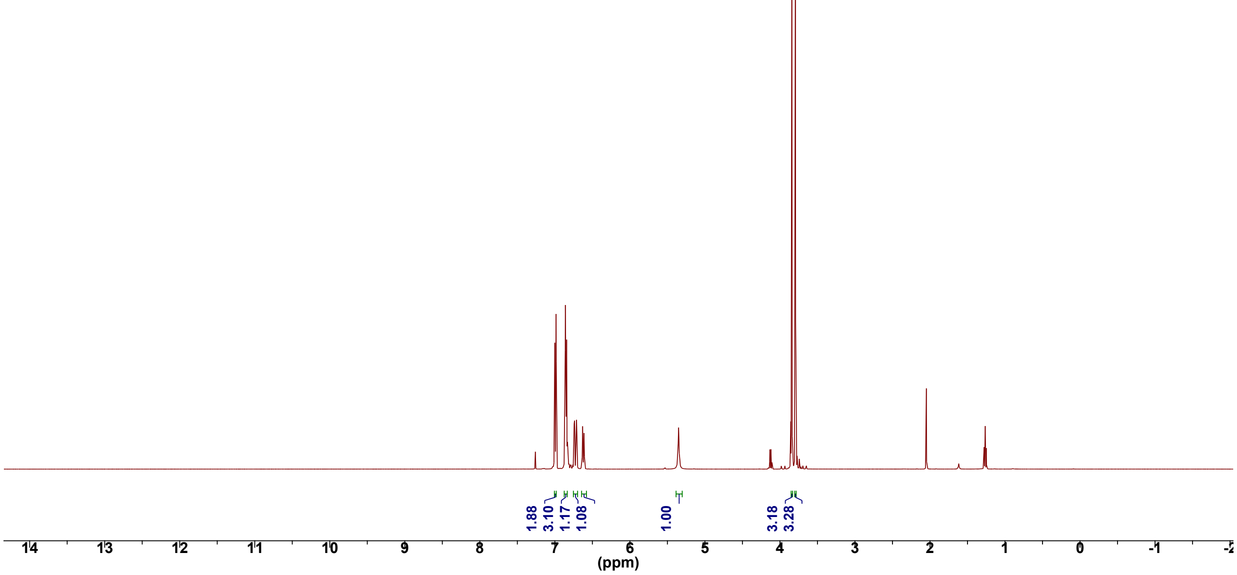

^{13}C NMR ($CDCl_3$, 126 MHz) of P114

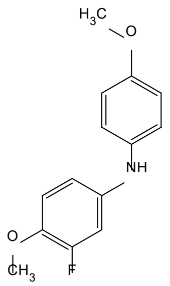

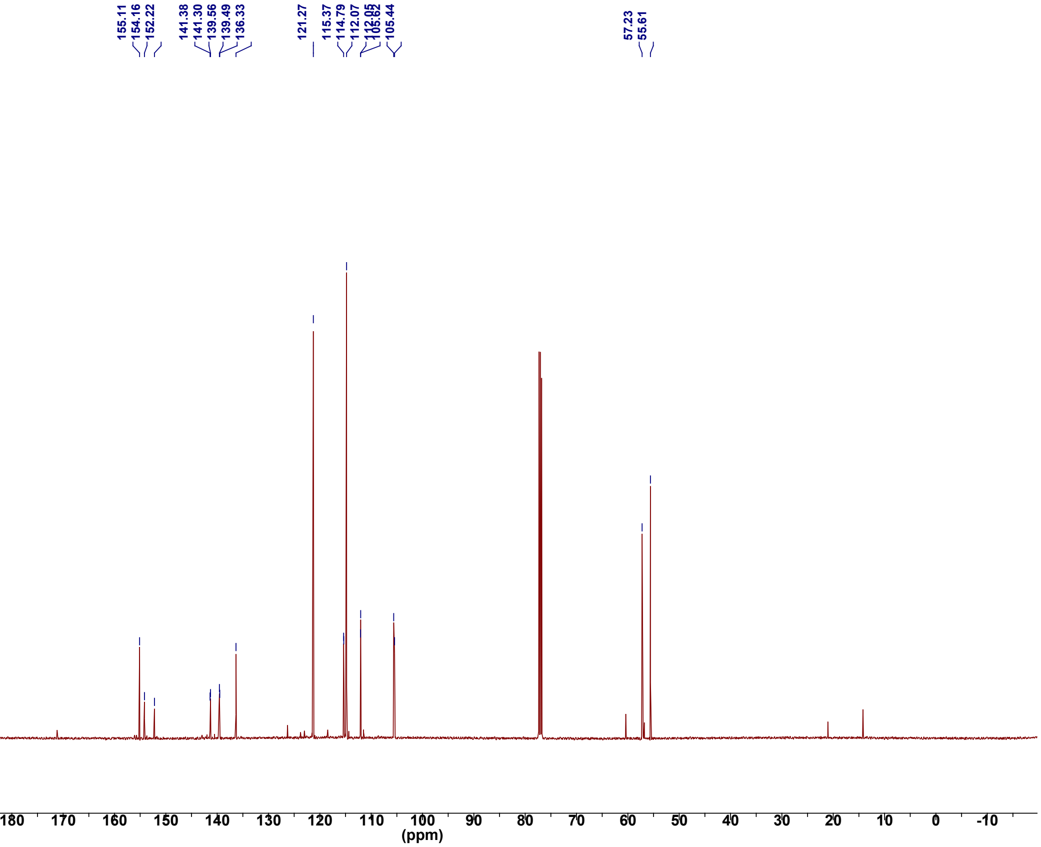

^{19}F NMR ($CDCl_3$, 471 MHz) of P114

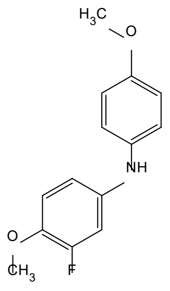

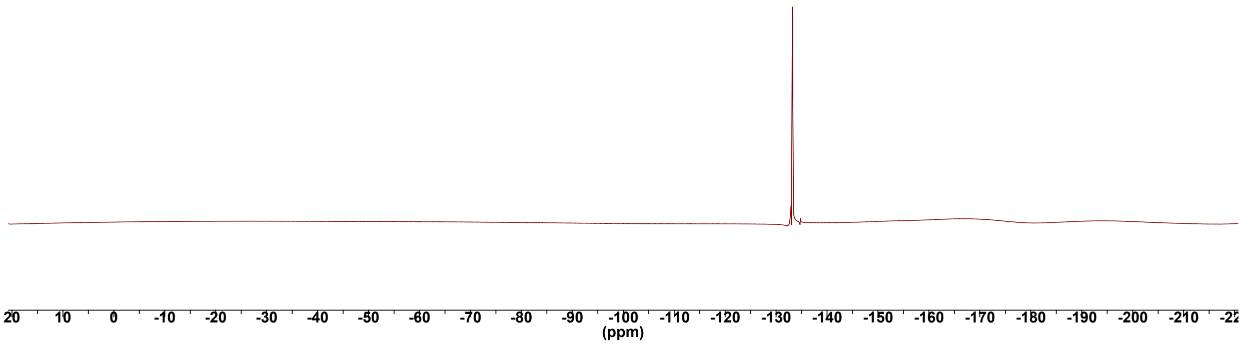

^{1}H NMR ($CDCl_3$, 500 MHz) of P115

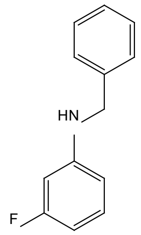

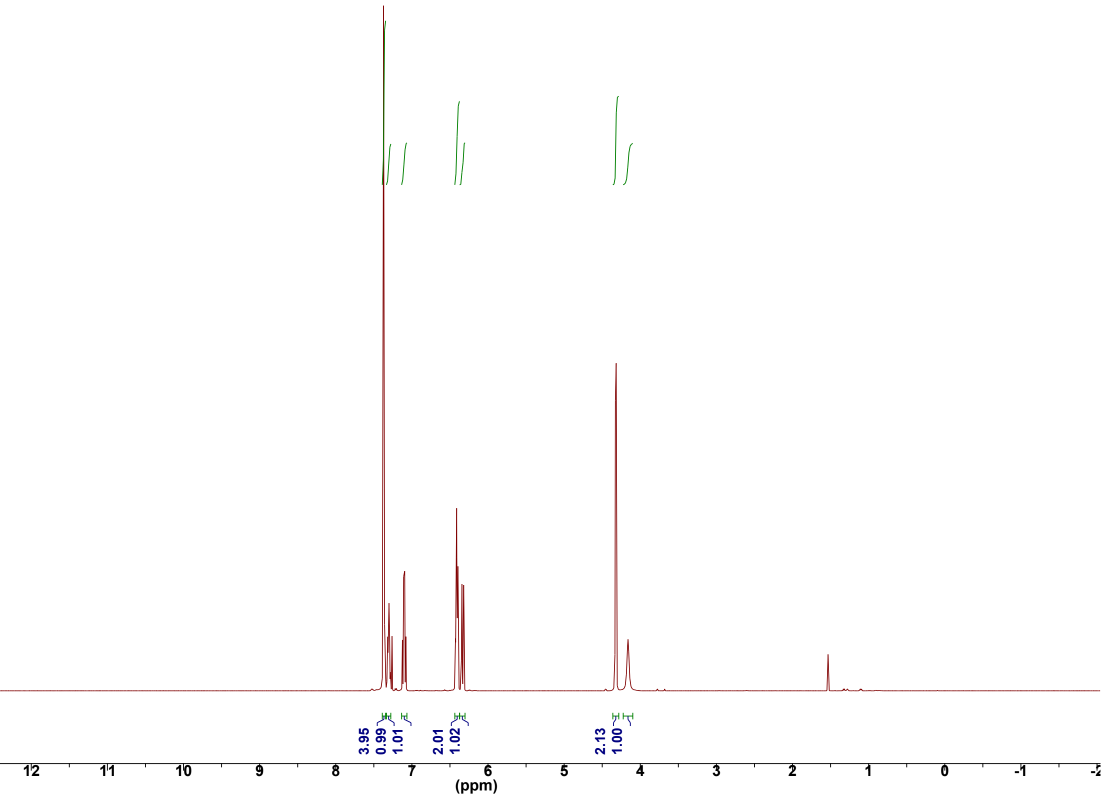

^{13}C NMR ($CDCl_3$, 126 MHz) of P115

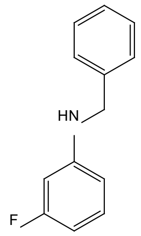

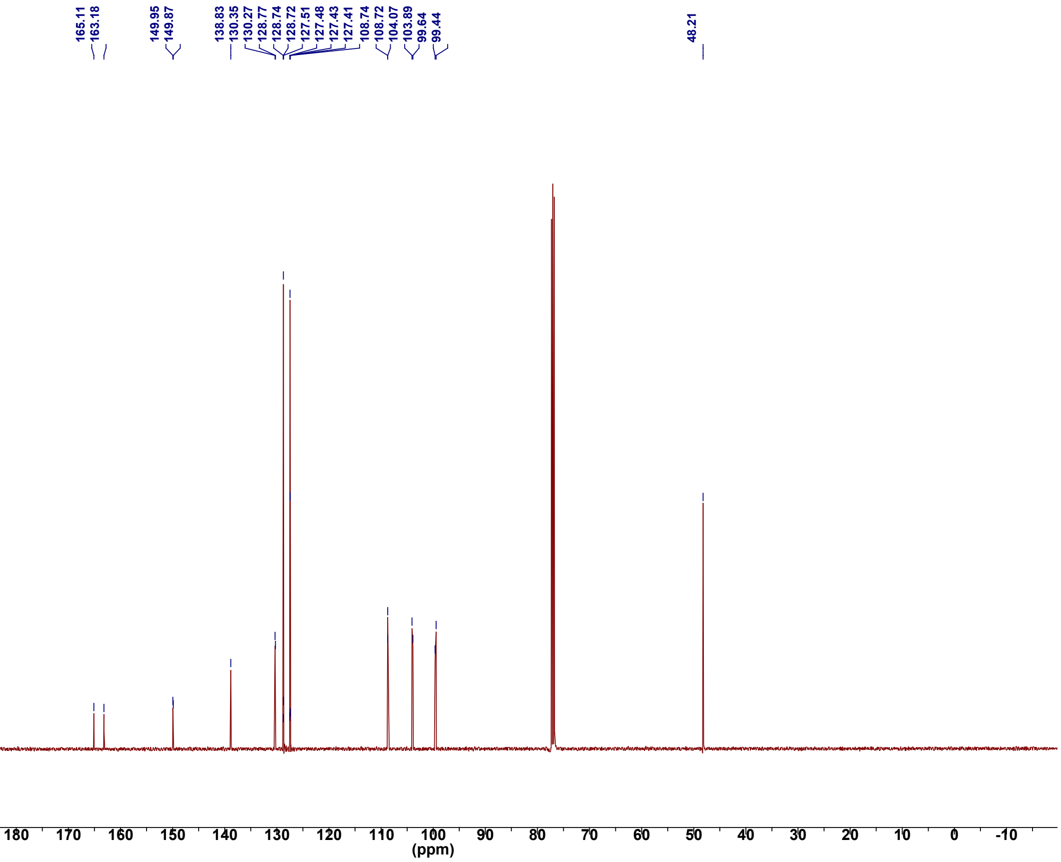

^{19}F NMR ($CDCl_3$, 471 MHz) of P115

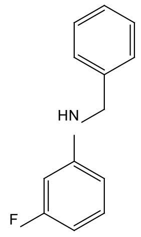

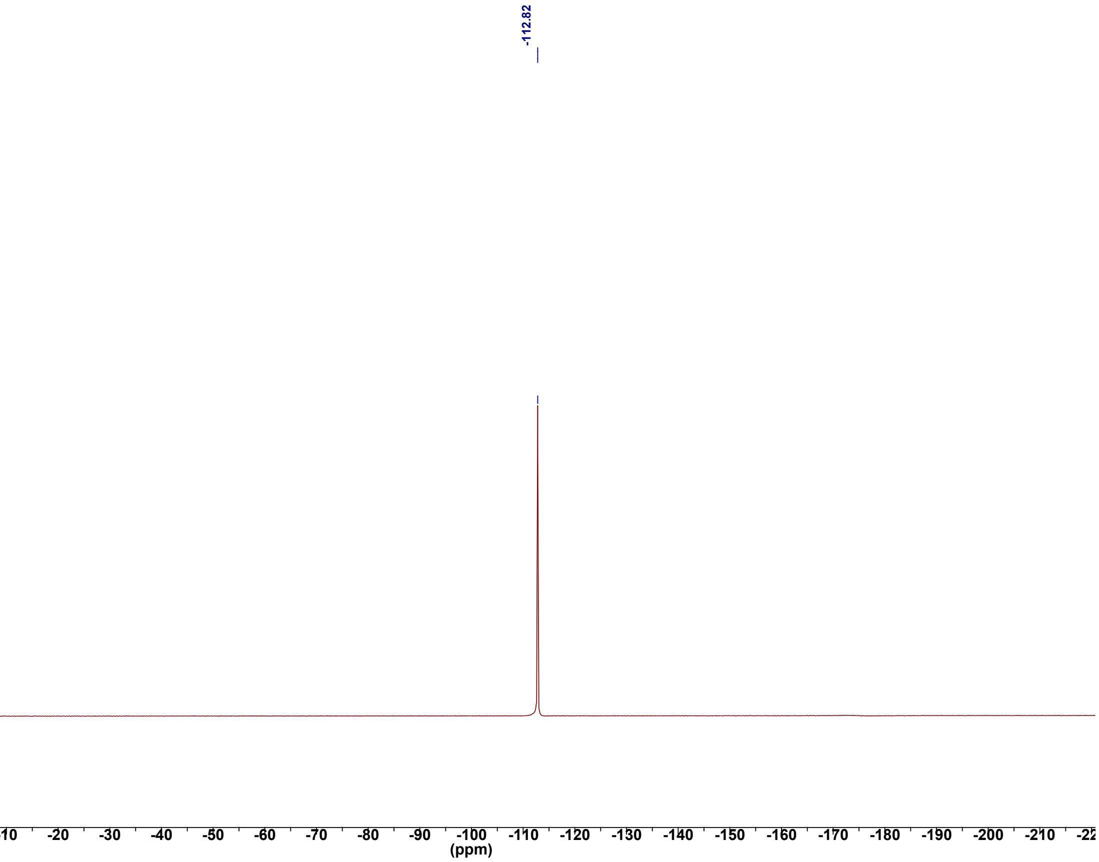

^1H NMR ($CDCl_3$, 500 MHz) of P116

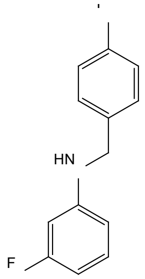

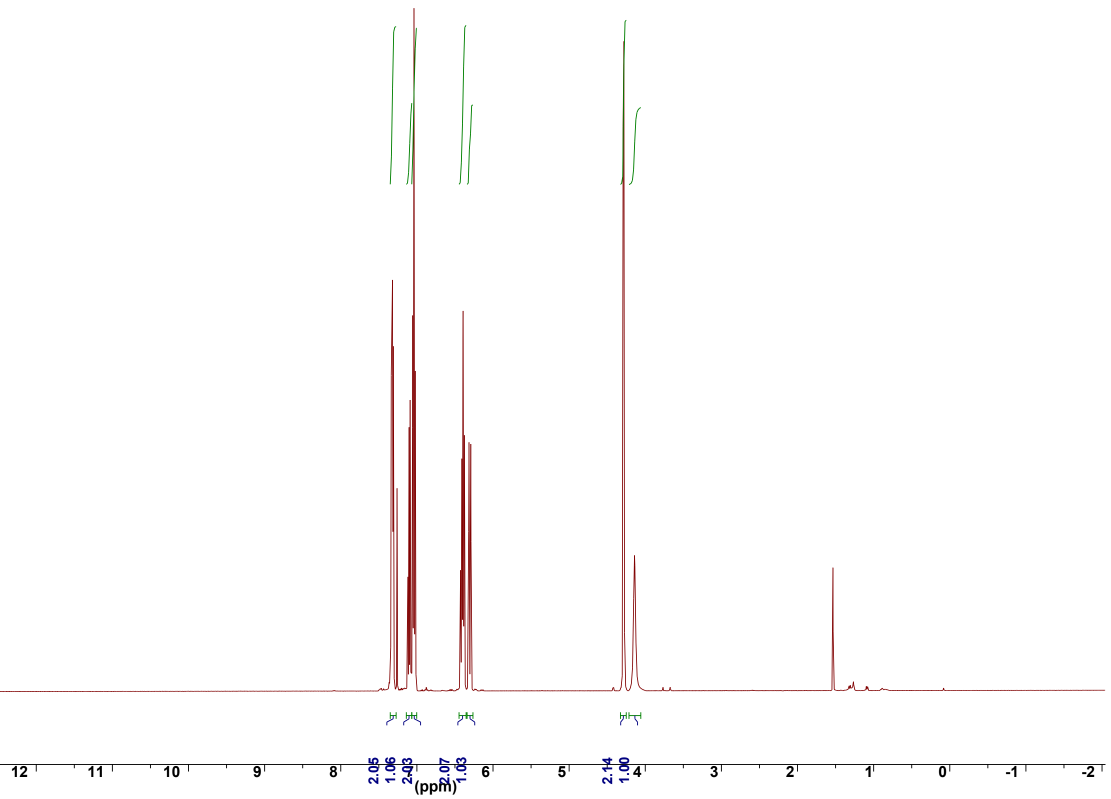

^{13}C NMR ($CDCl_3$, 126 MHz) of P116

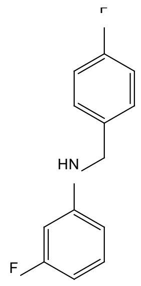

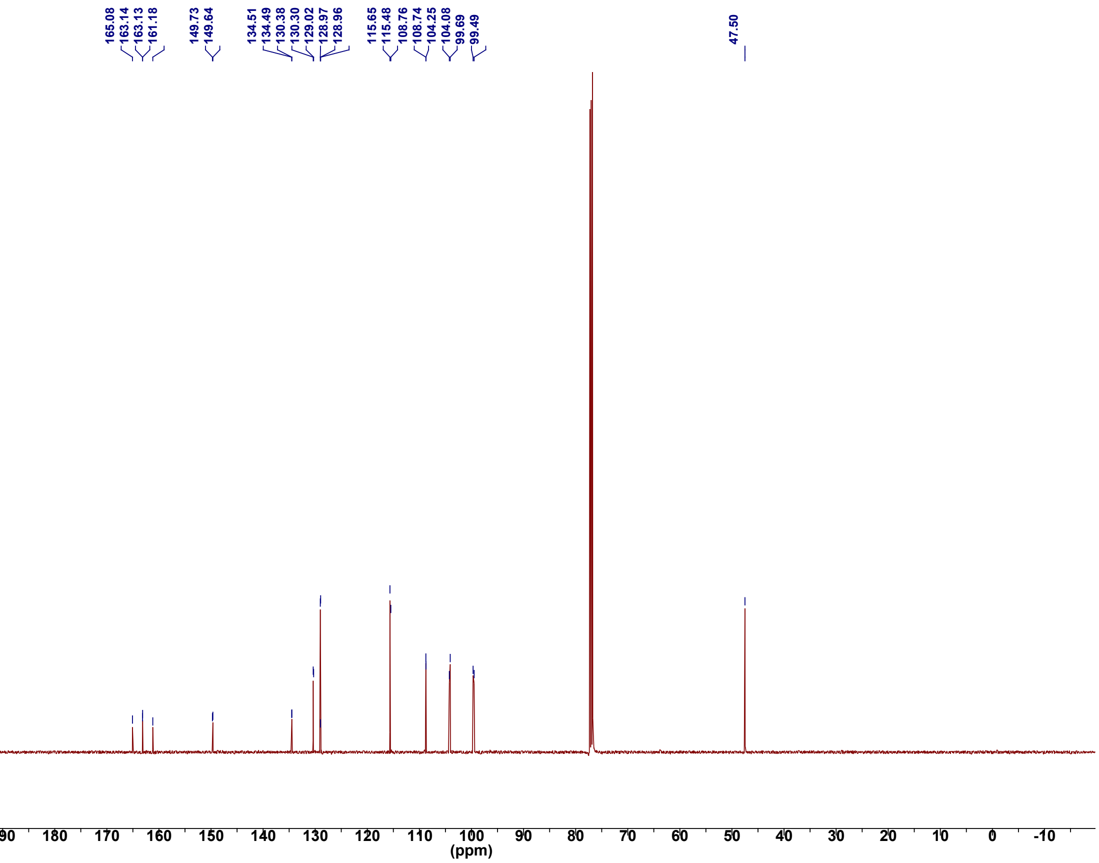

^{19}F NMR ($CDCl_3$, 471 MHz) of P116

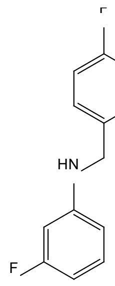

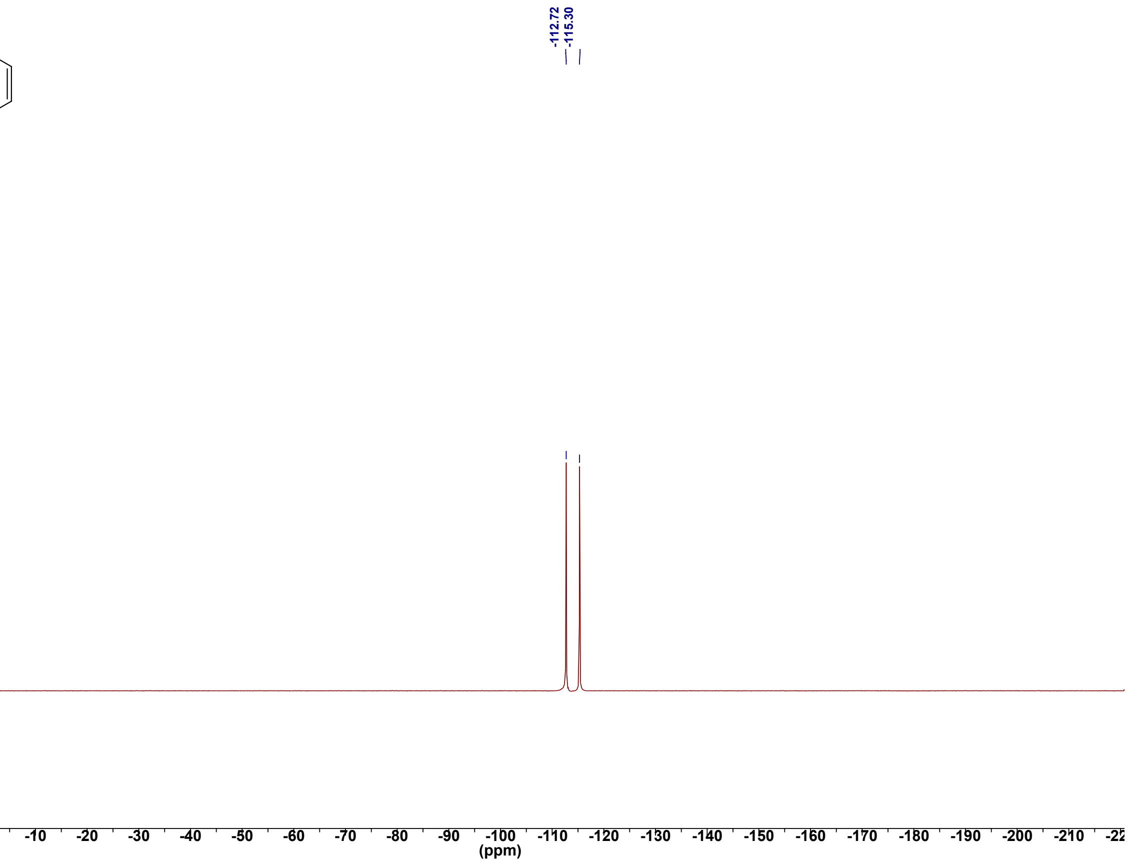

^{1}H NMR ($CDCl_3$, 500 MHz) of P117

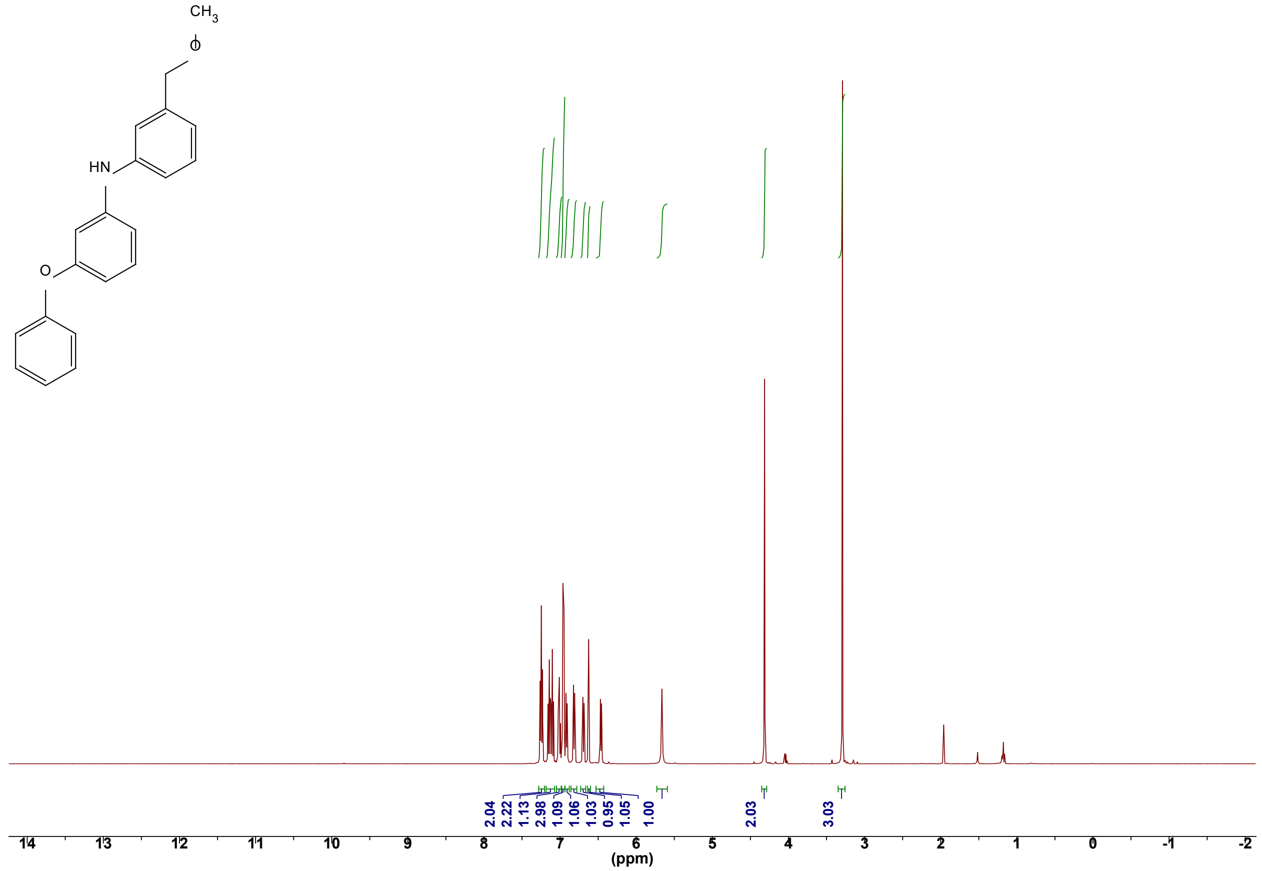

^{13}C NMR ($CDCl_3$, 126 MHz) of P117

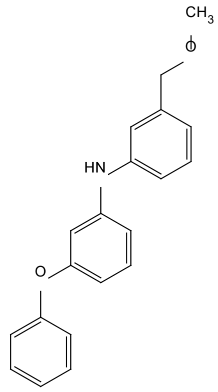

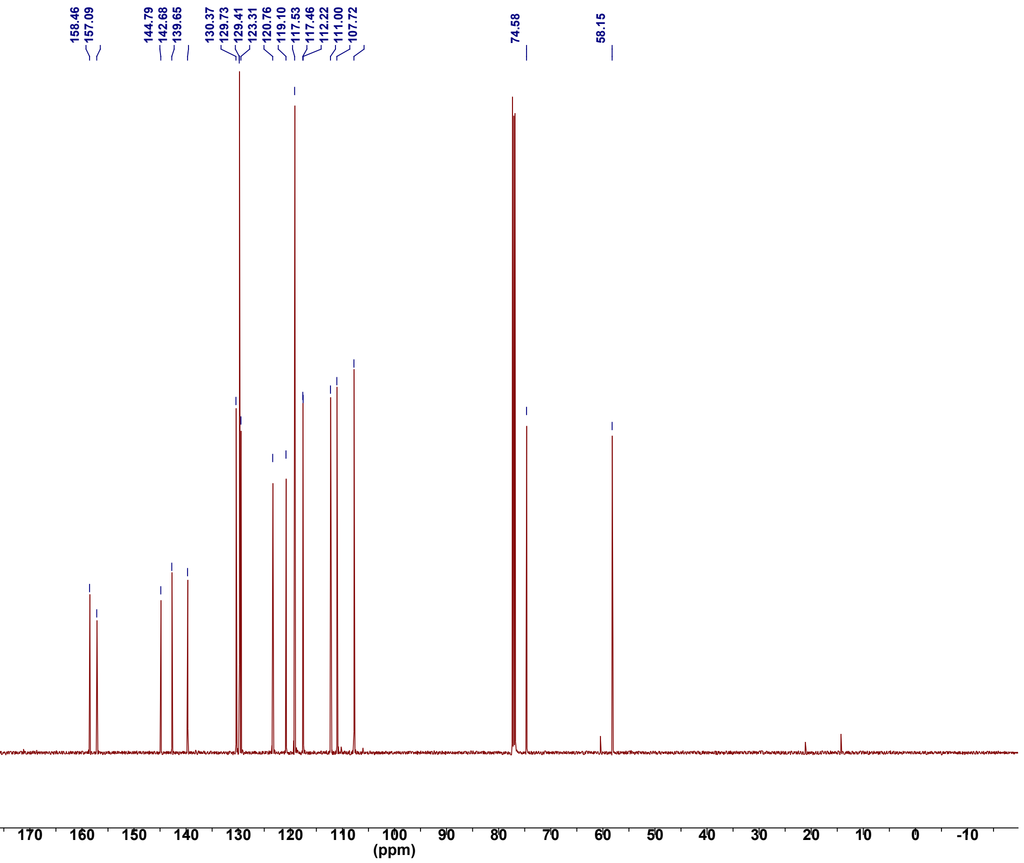

^{1}H NMR ($CDCl_3$, 500 MHz) of P118

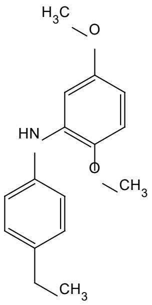

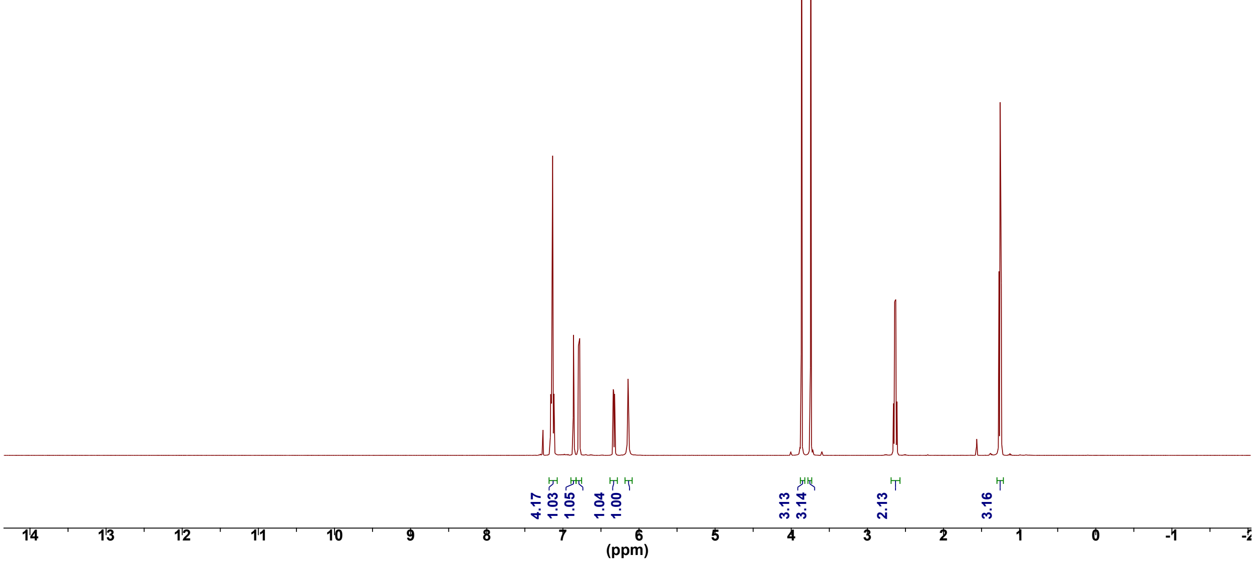

^{13}C NMR ($CDCl_3$, 126 MHz) of P118

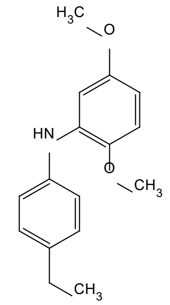

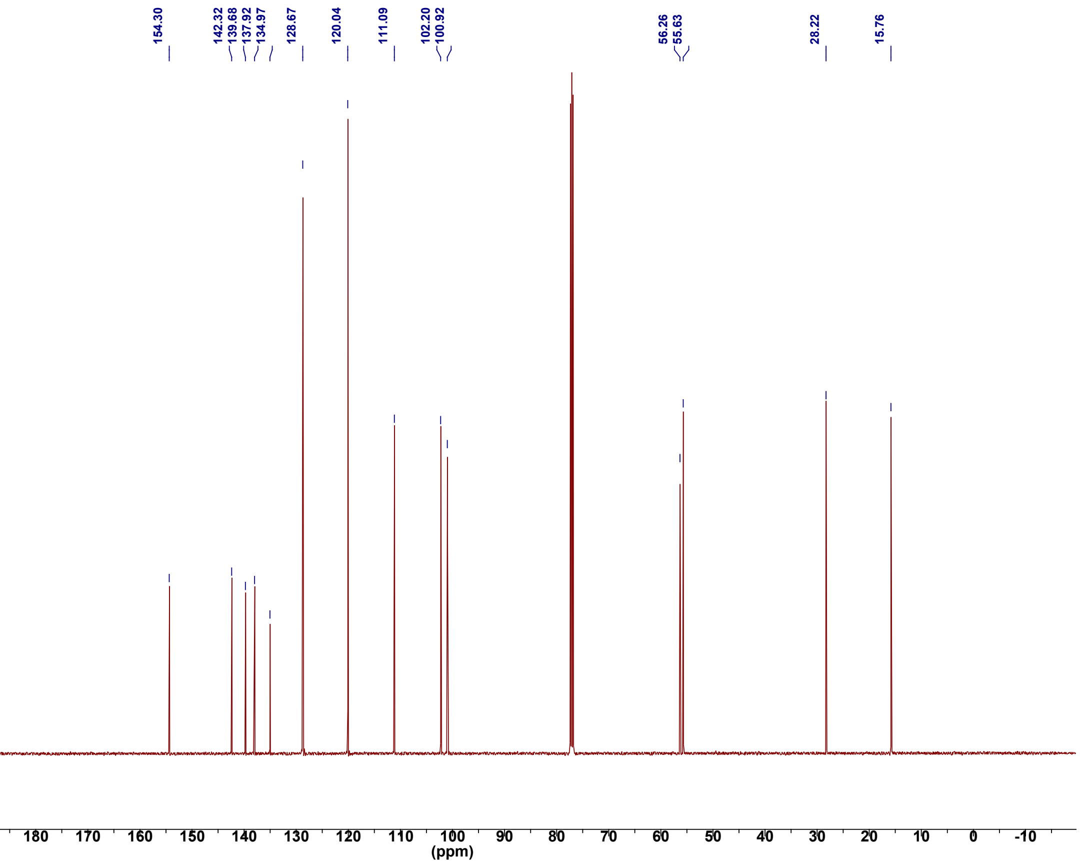

^{1}H NMR ($CDCl_3$, 500 MHz) of P119

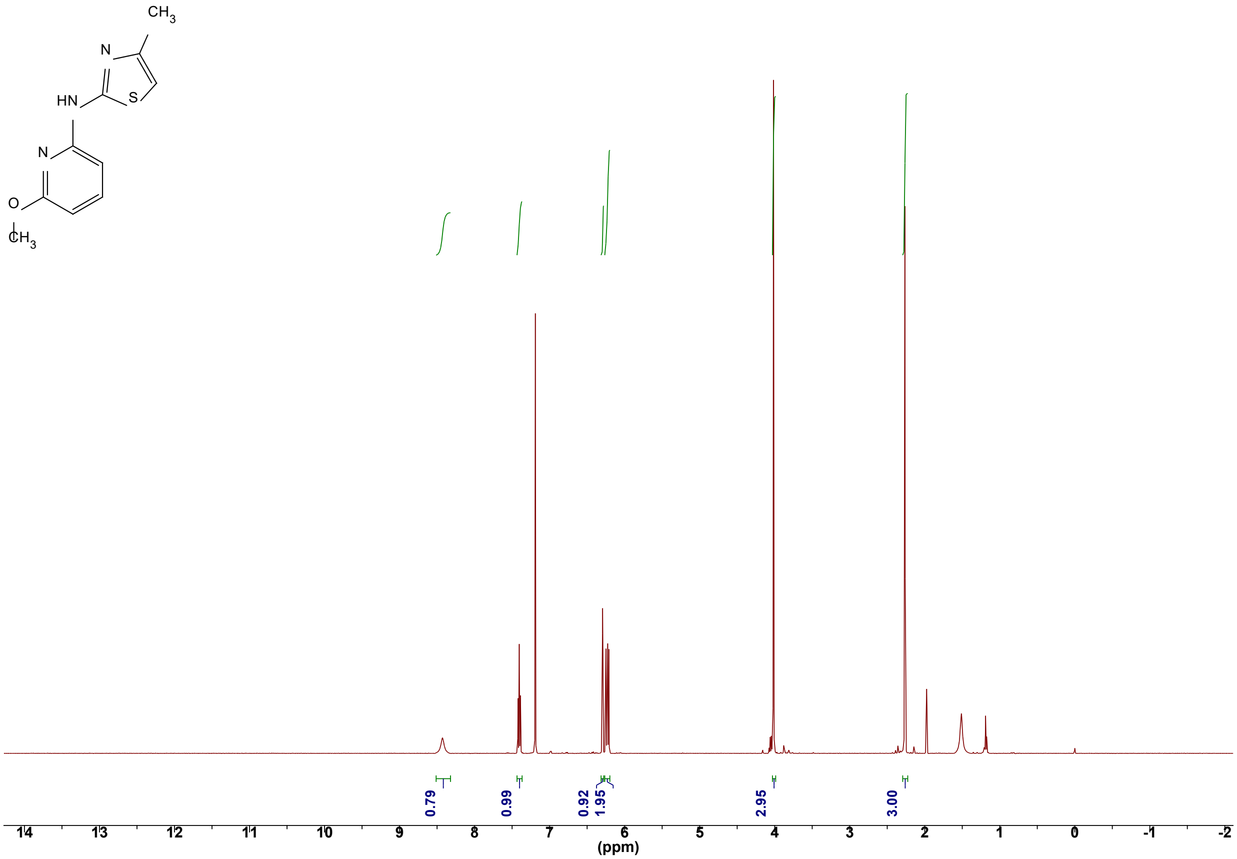

^{13}C NMR ($CDCl_3$, 126 MHz) of P119

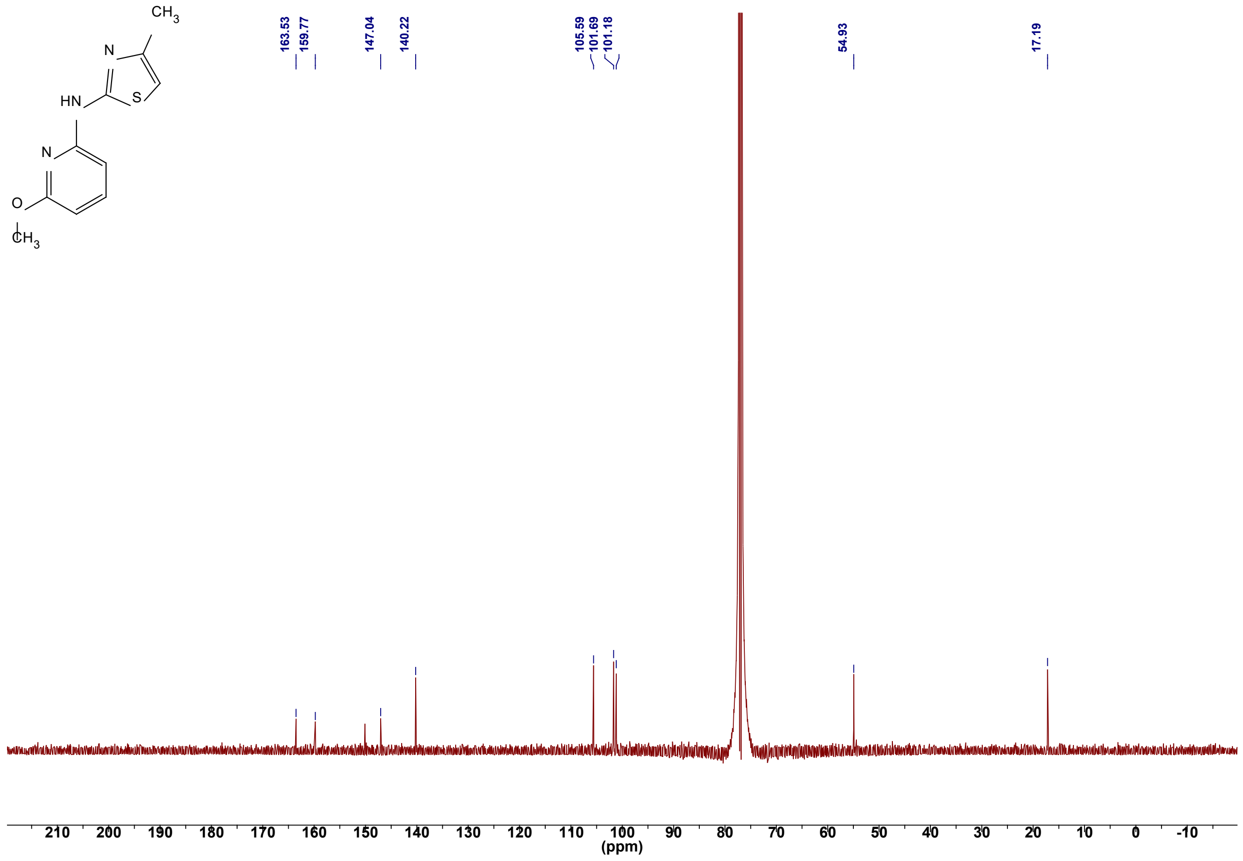

^1H NMR ($CDCl_3$, 500 MHz) of P120

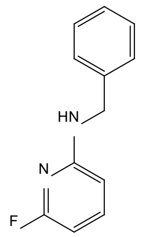

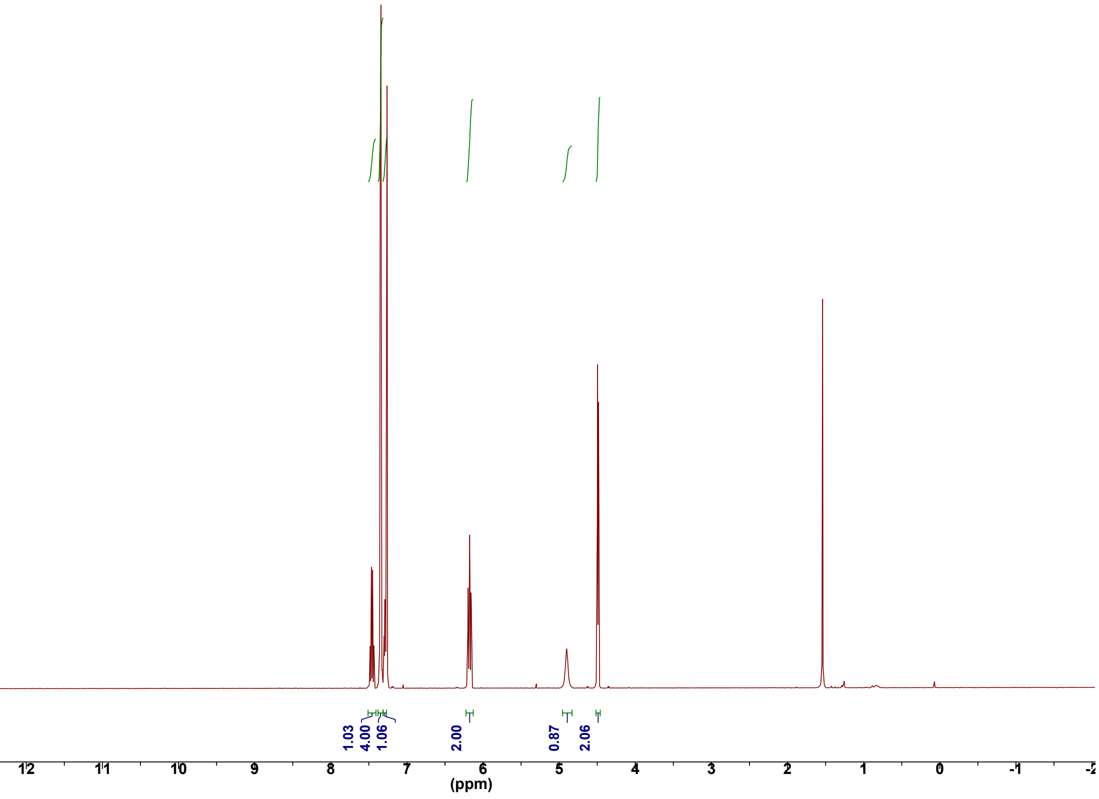

^{13}C NMR ($CDCl_3$, 126 MHz) of P120

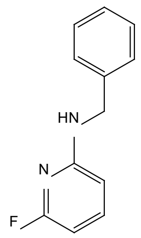

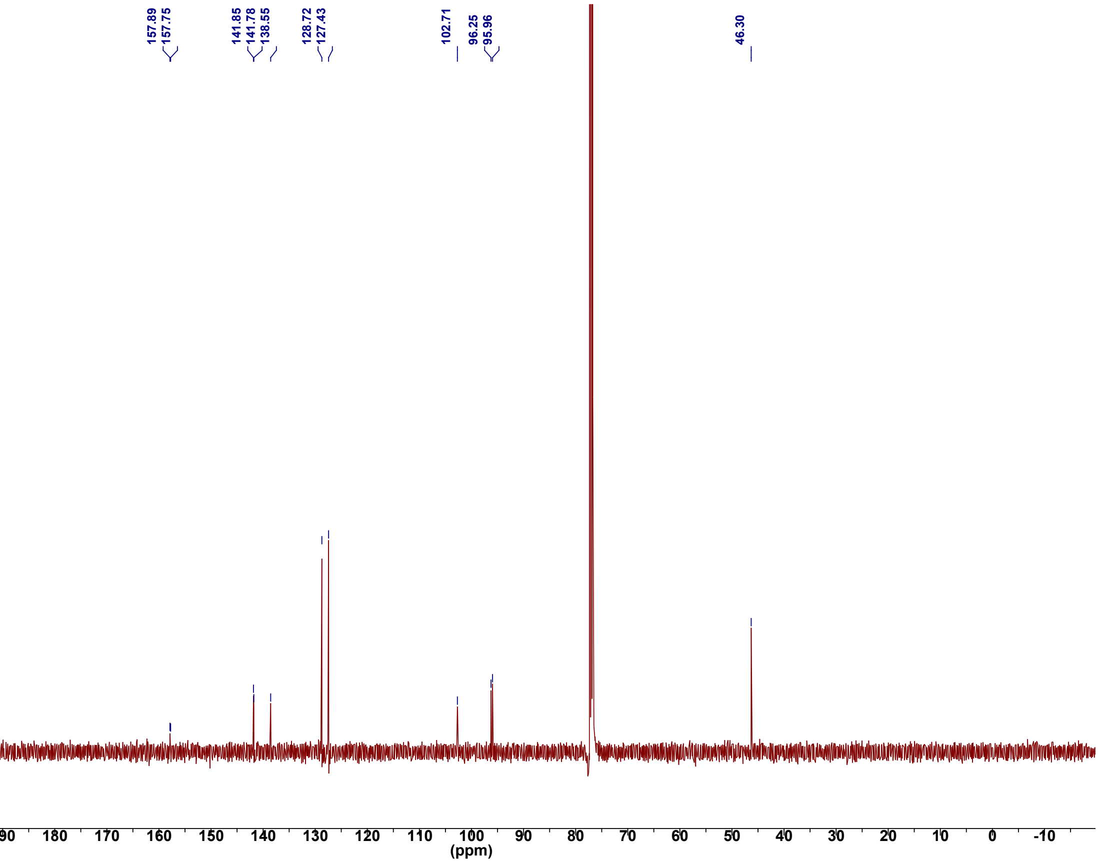

^{19}F NMR ($CDCl_3$, 471 MHz) of P120

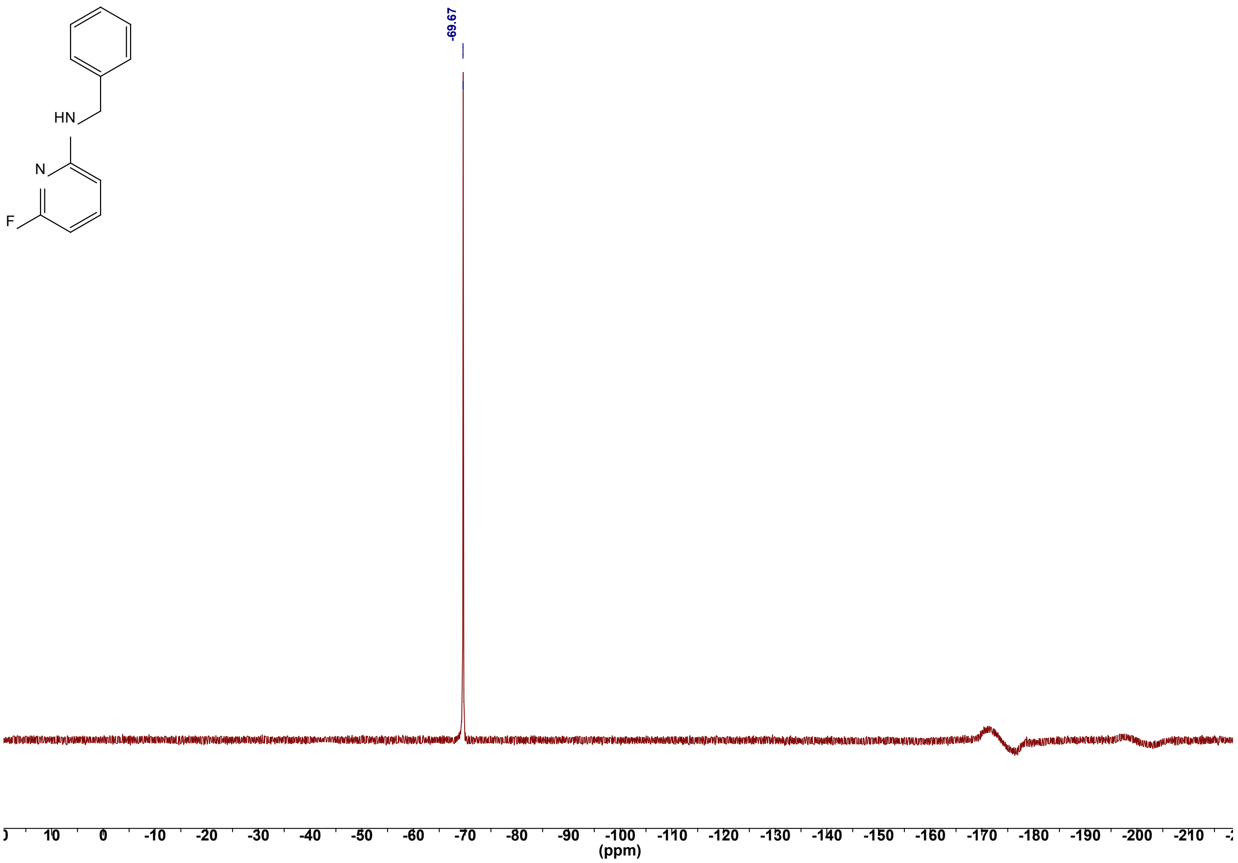

^{1}H NMR ($CDCl_3$, 500 MHz) of P121

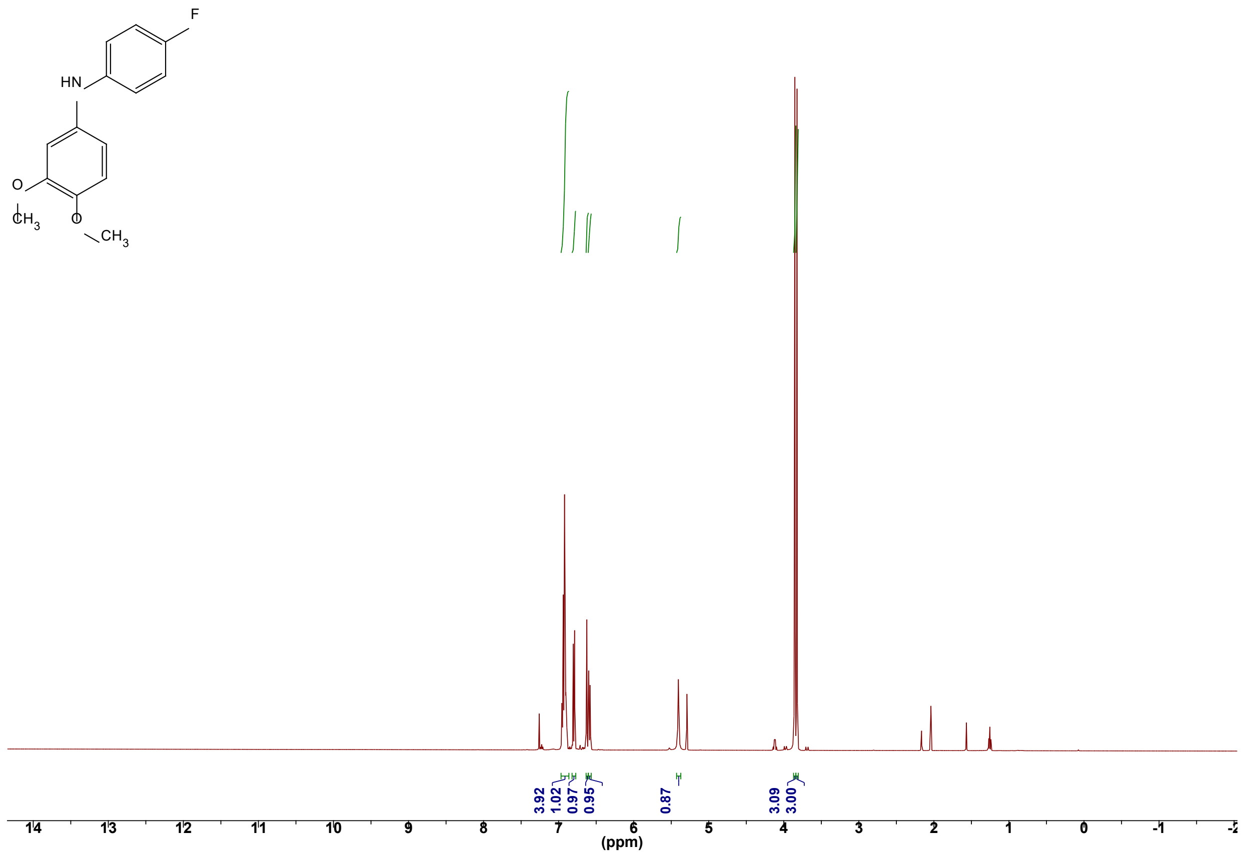

^{13}C NMR ($CDCl_3$, 126 MHz) of P121

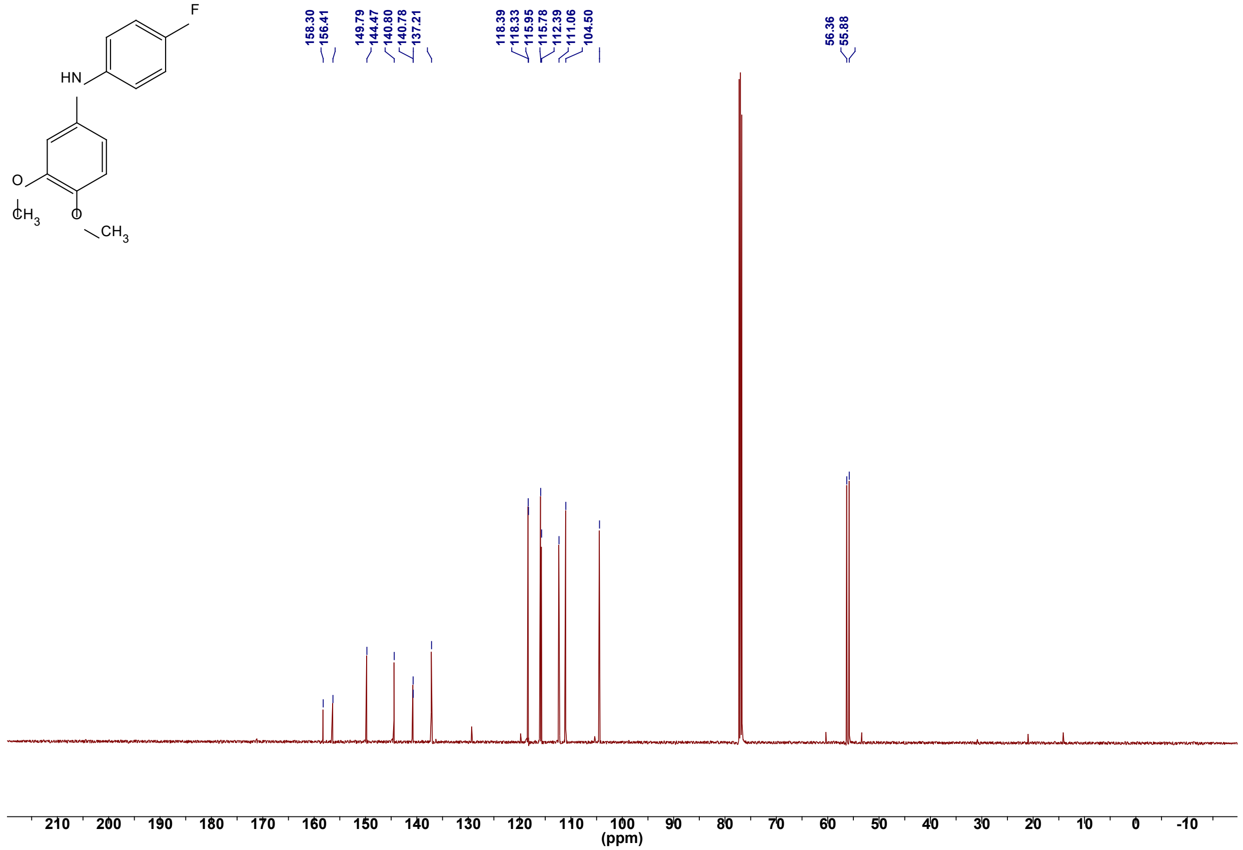

^{19}F NMR ($CDCl_3$, 471 MHz) of P121

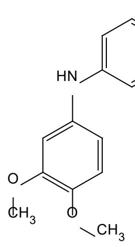

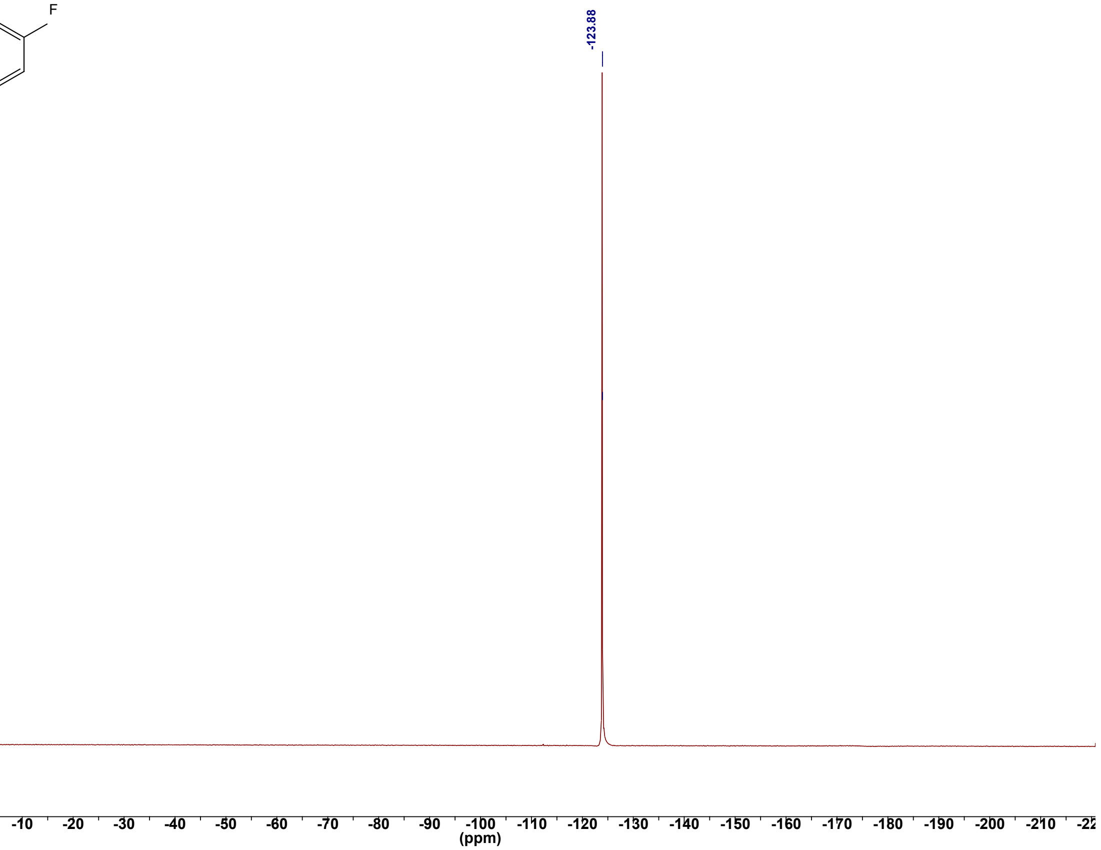

^1H NMR ($CDCl_3$, 500 MHz) of P122

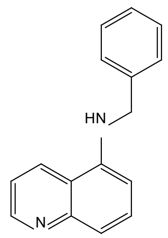

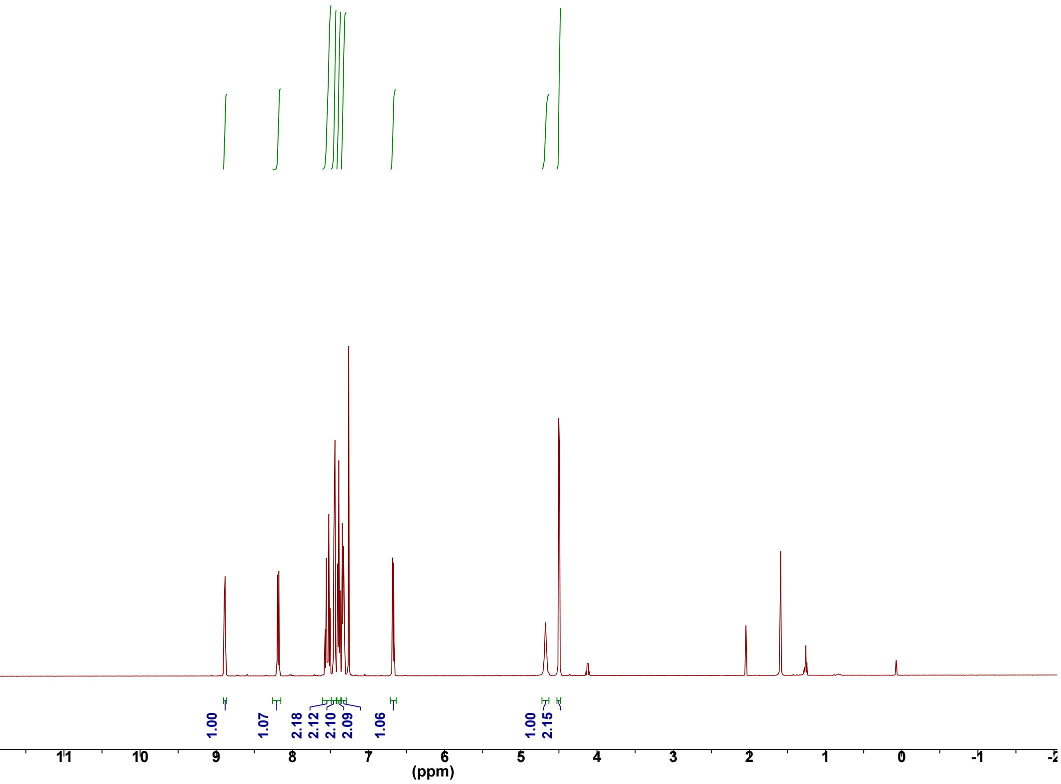

^{13}C NMR ($CDCl_3$, 126 MHz) of P122

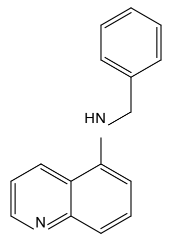

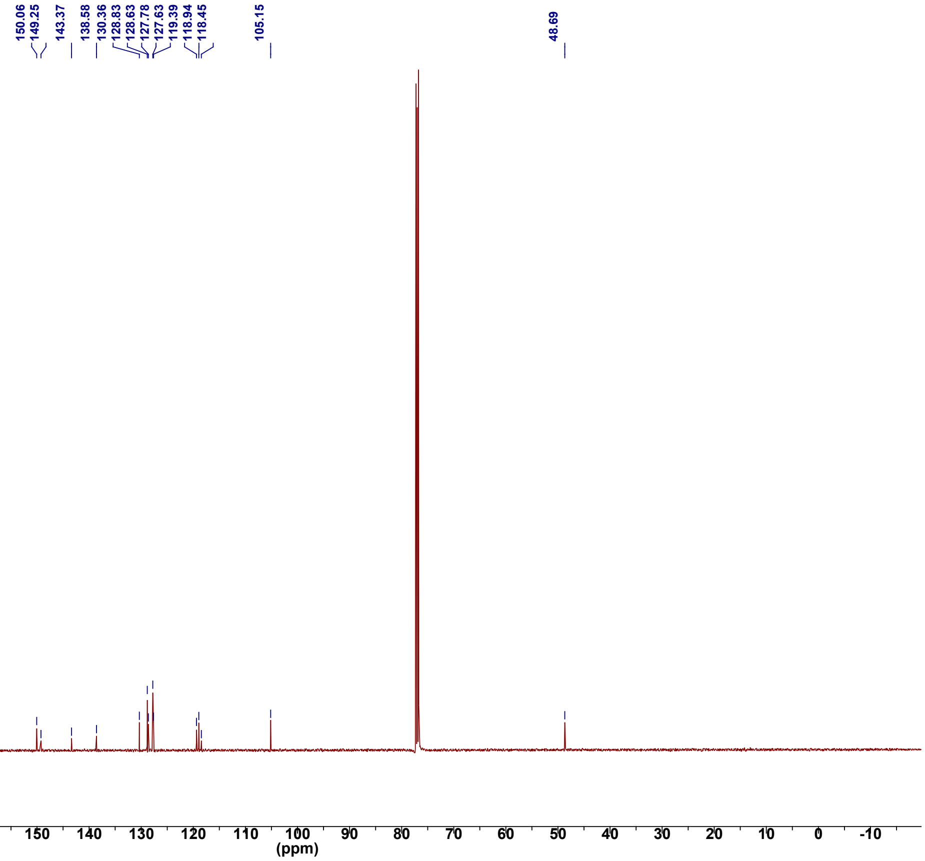

^{1}H NMR ($CDCl_3$, 500 MHz) of P123

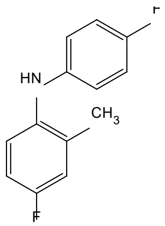

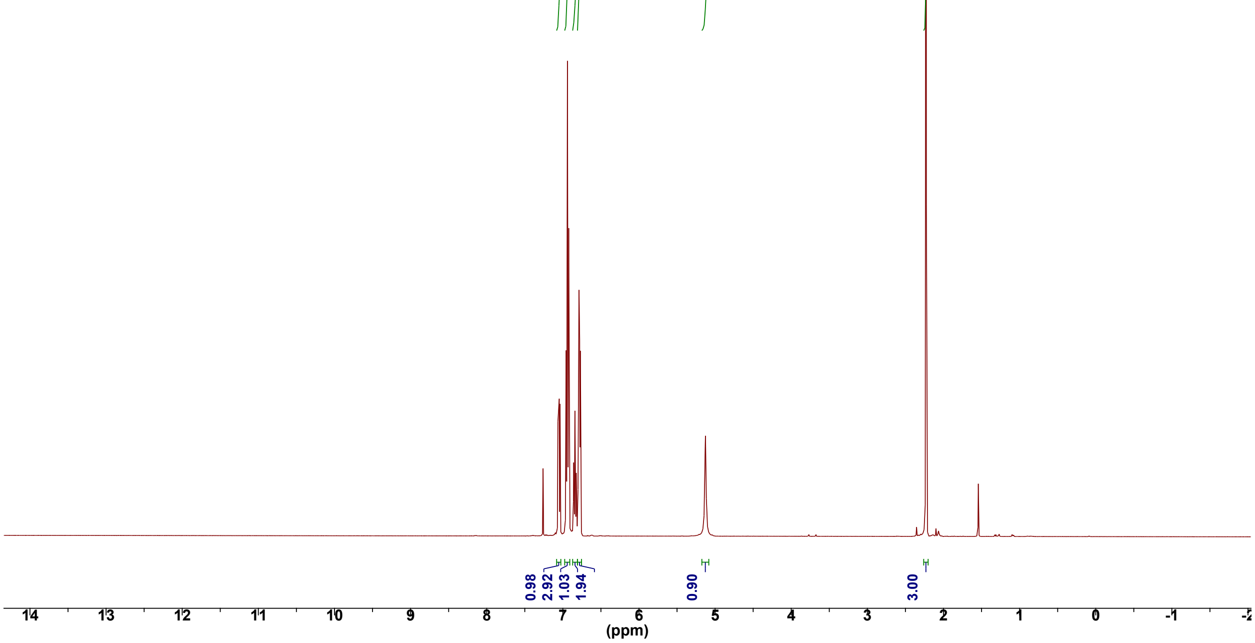

^{13}C NMR ($CDCl_3$, 126 MHz) of P123

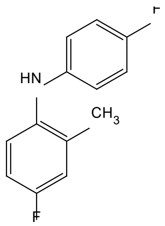

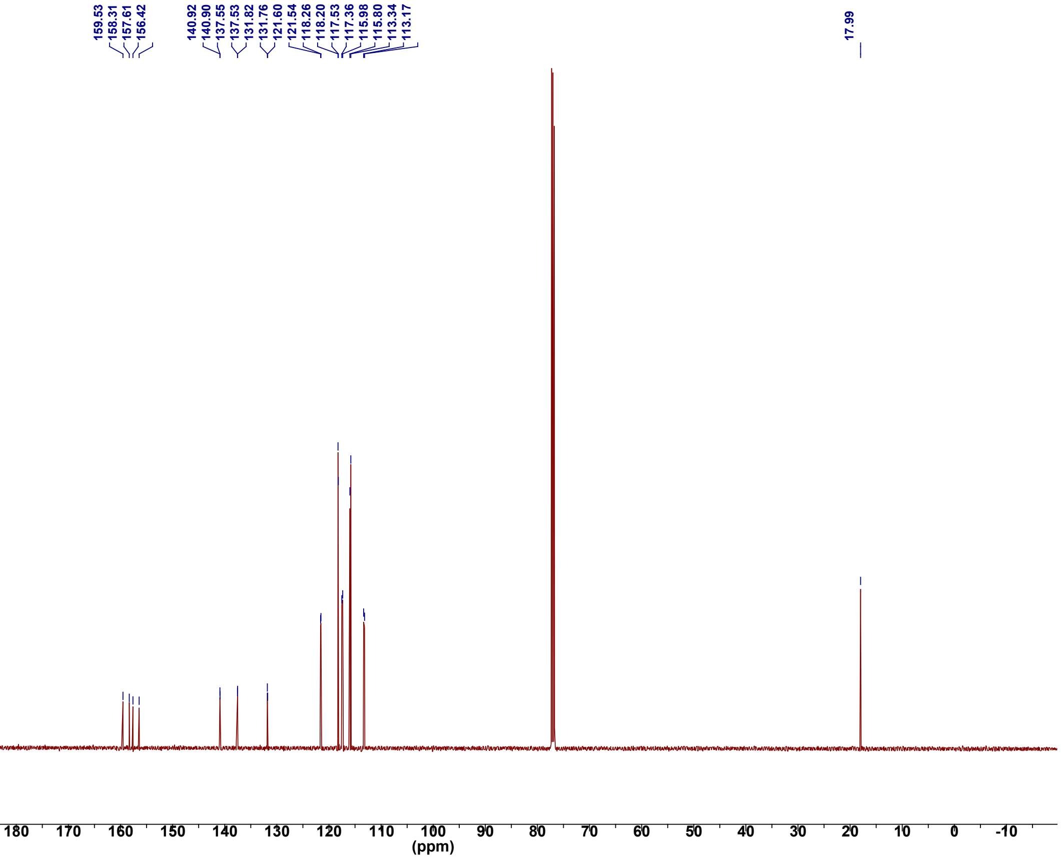

^{19}F NMR ($CDCl_3$, 471 MHz) of P123

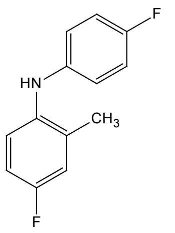

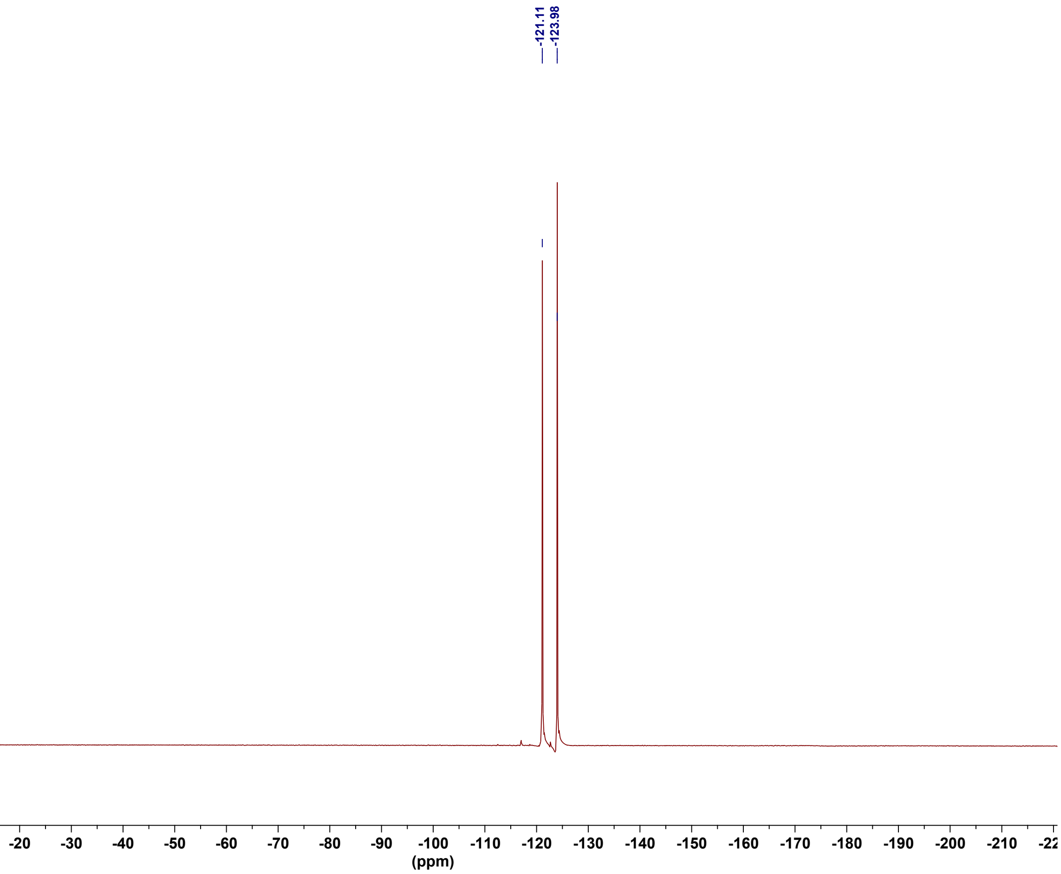

^{1}H NMR ($CDCl_3$, 500 MHz) of P124

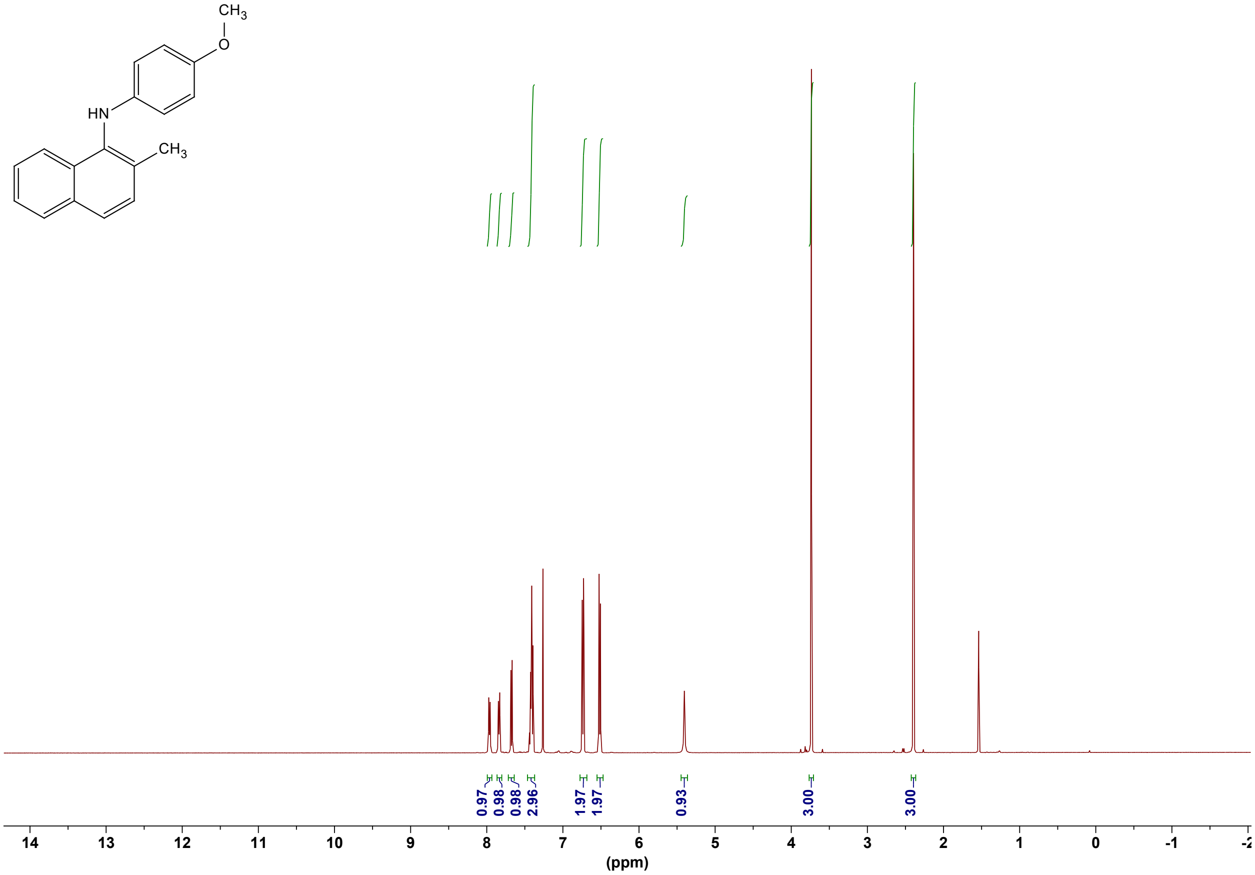

^{13}C NMR ($CDCl_3$, 126 MHz) of P124

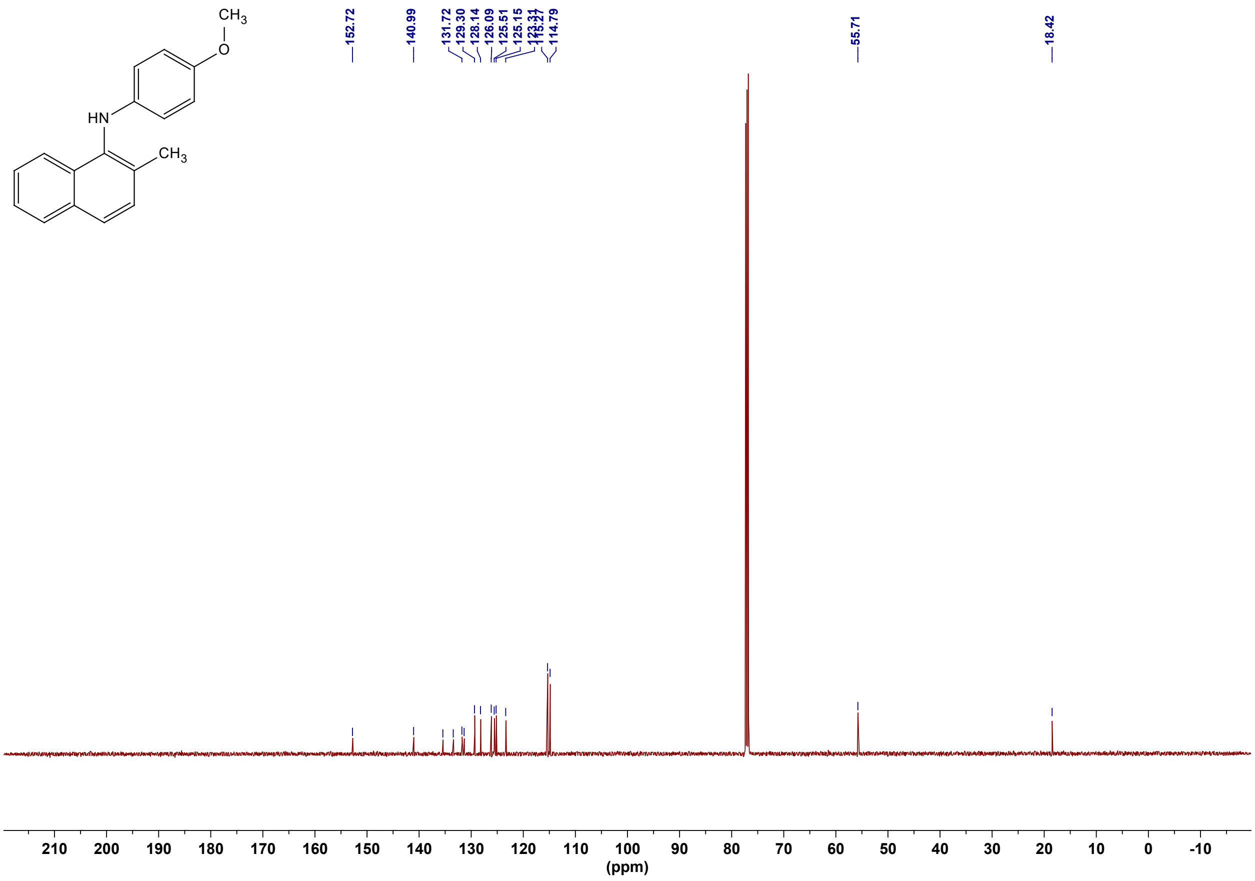

^{1}H NMR ($CDCl_3$, 500 MHz) of P125

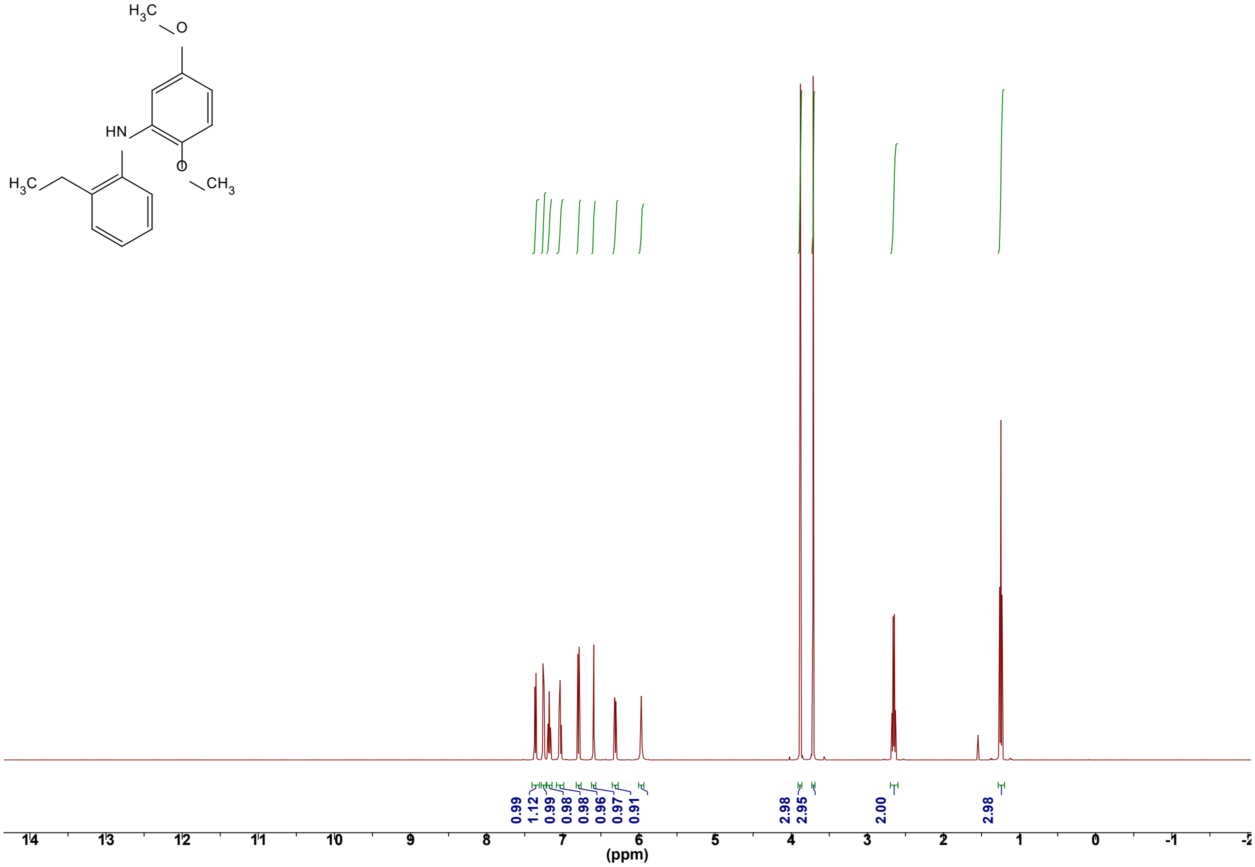

^{13}C NMR ($CDCl_3$, 126 MHz) of P125

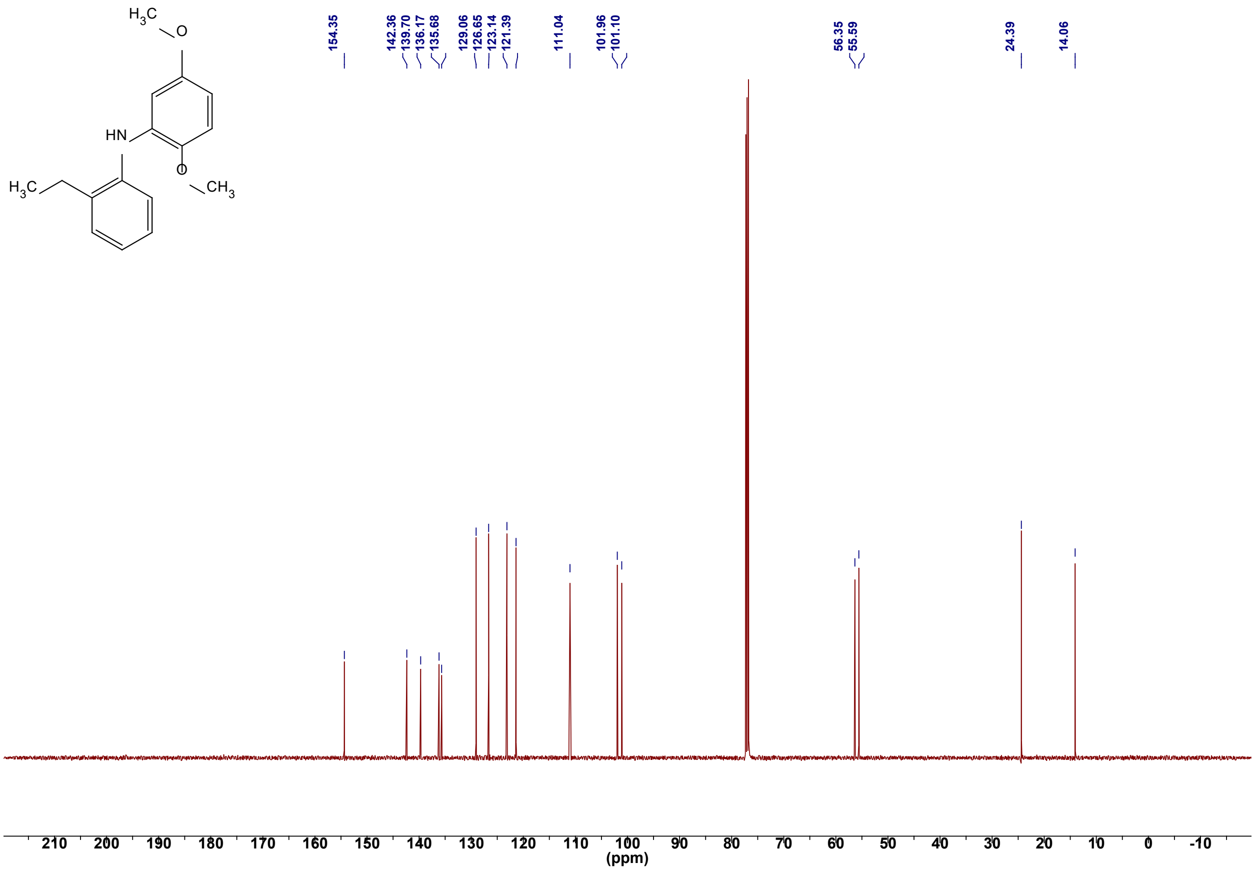

^{1}H NMR ($CDCl_3$, 500 MHz) of P126

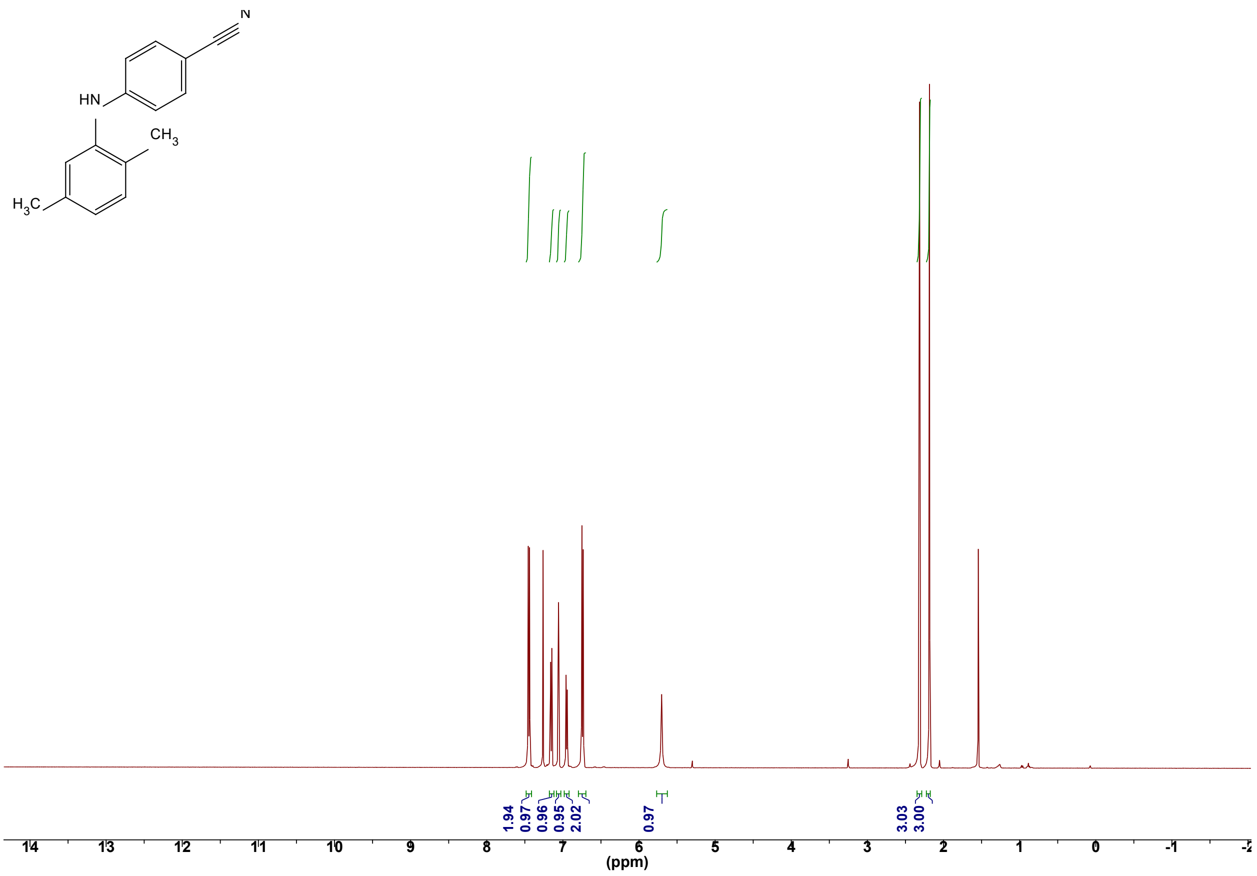

^{13}C NMR ($CDCl_3$, 126 MHz) of P126

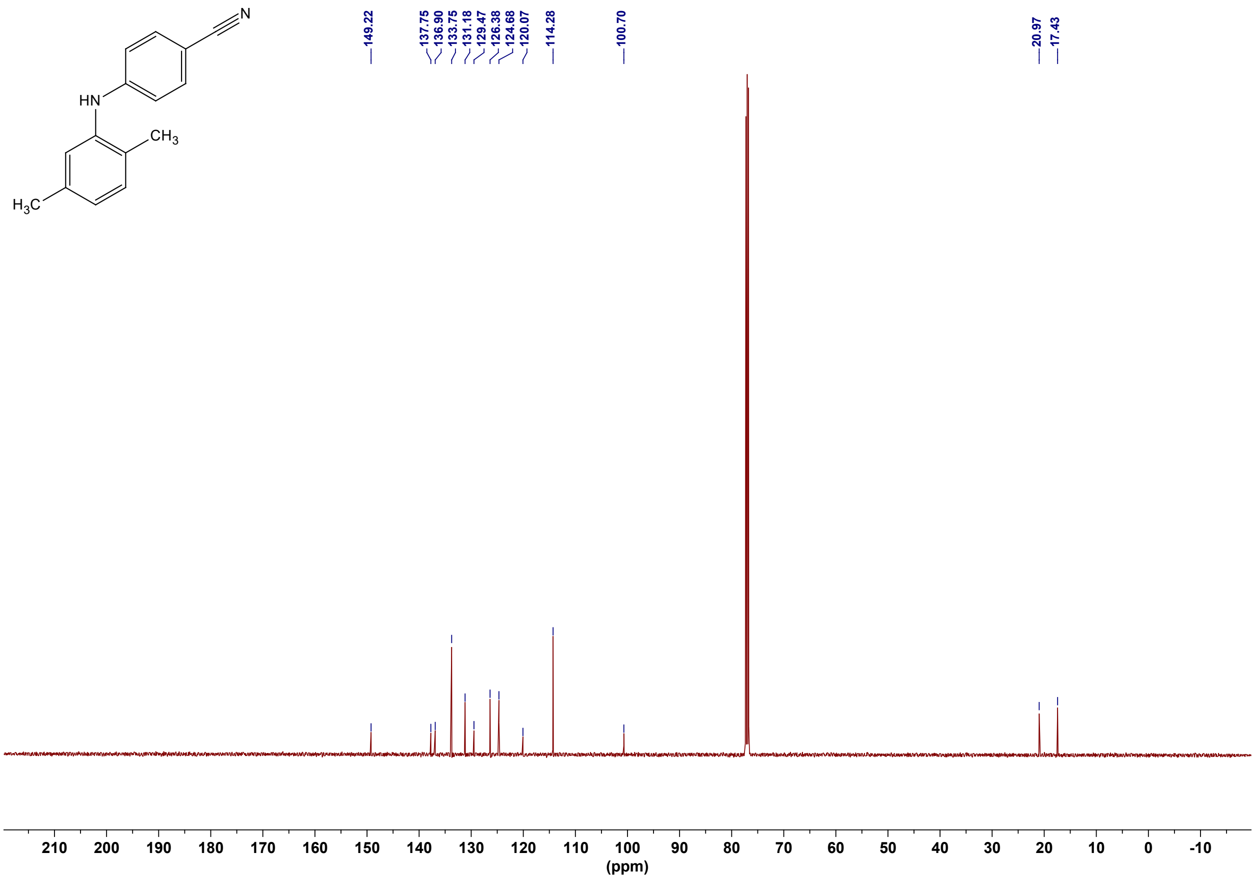

^1H NMR ($CDCl_3$, 500 MHz) of P127

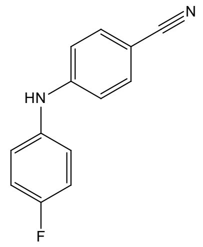

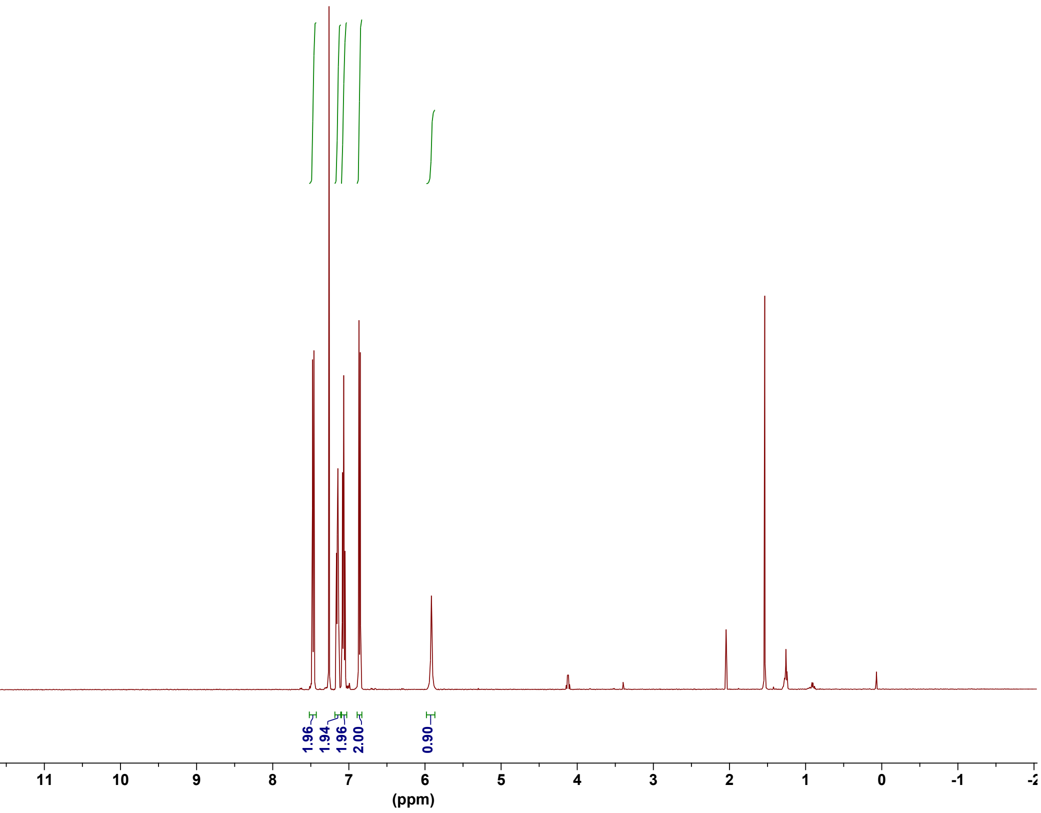

^{13}C NMR ($CDCl_3$, 126 MHz) of P127

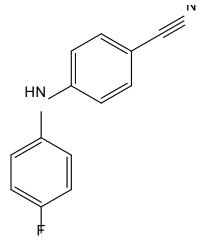

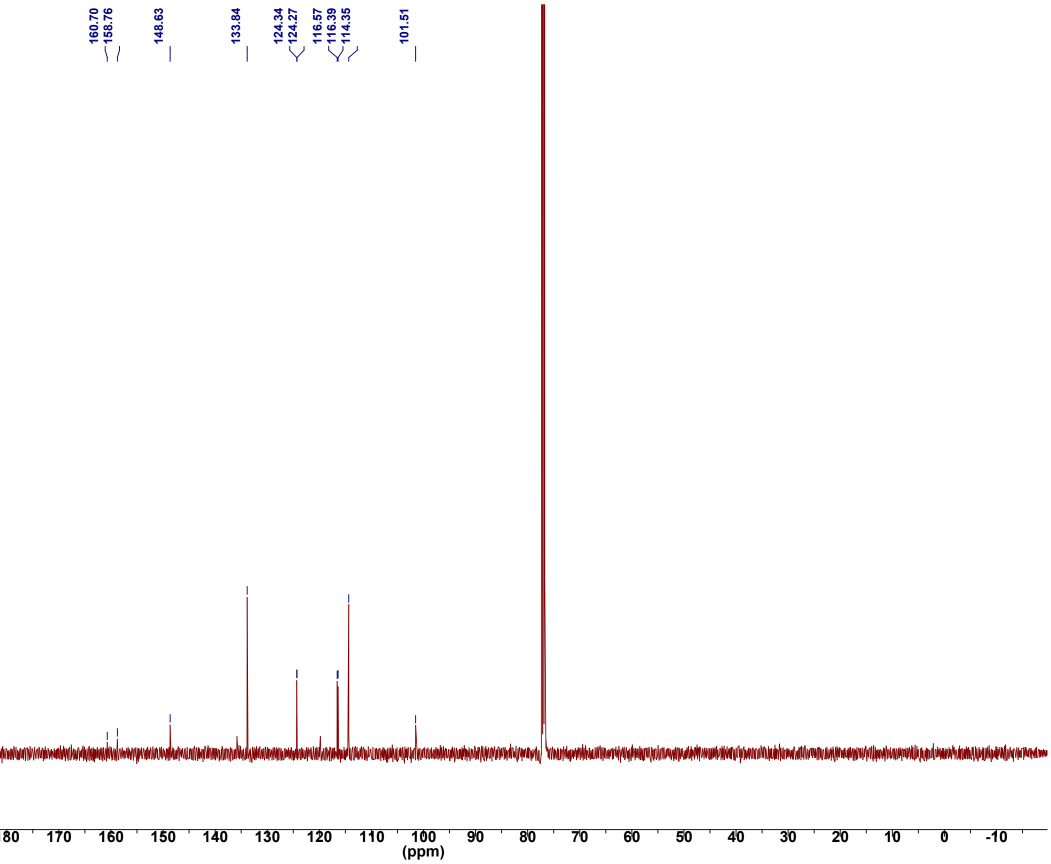

^{19}F NMR ($CDCl_3$, 471 MHz) of P127

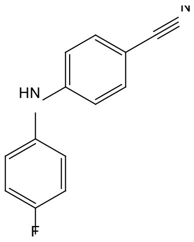

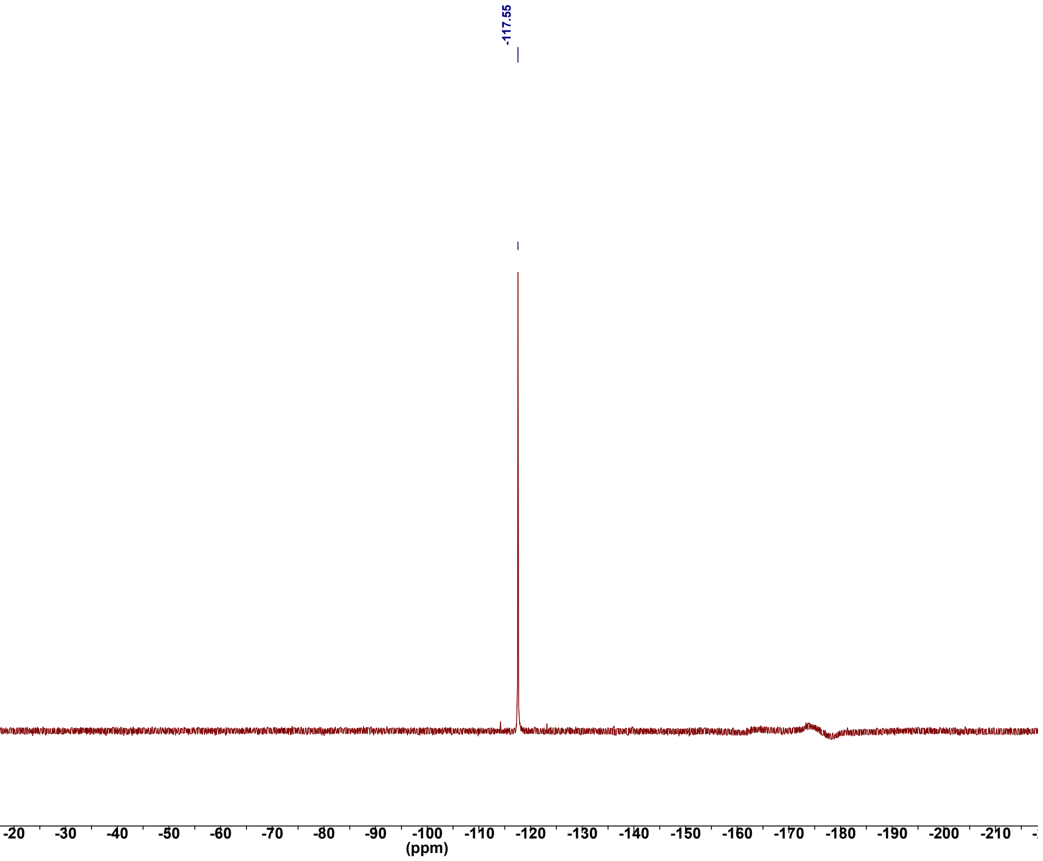

^1H NMR ($CDCl_3$, 500 MHz) of P200

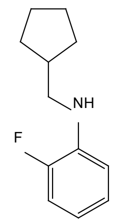

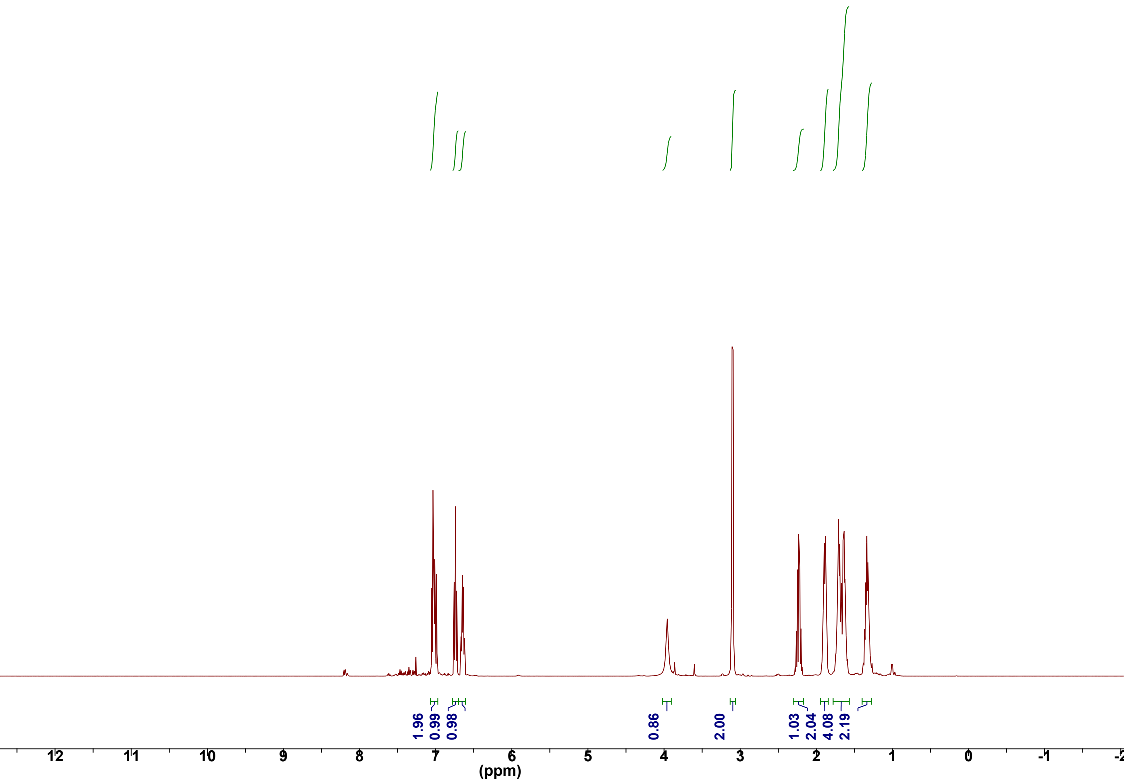

^{13}C NMR ($CDCl_3$, 126 MHz) of P200

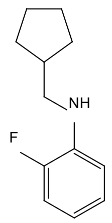

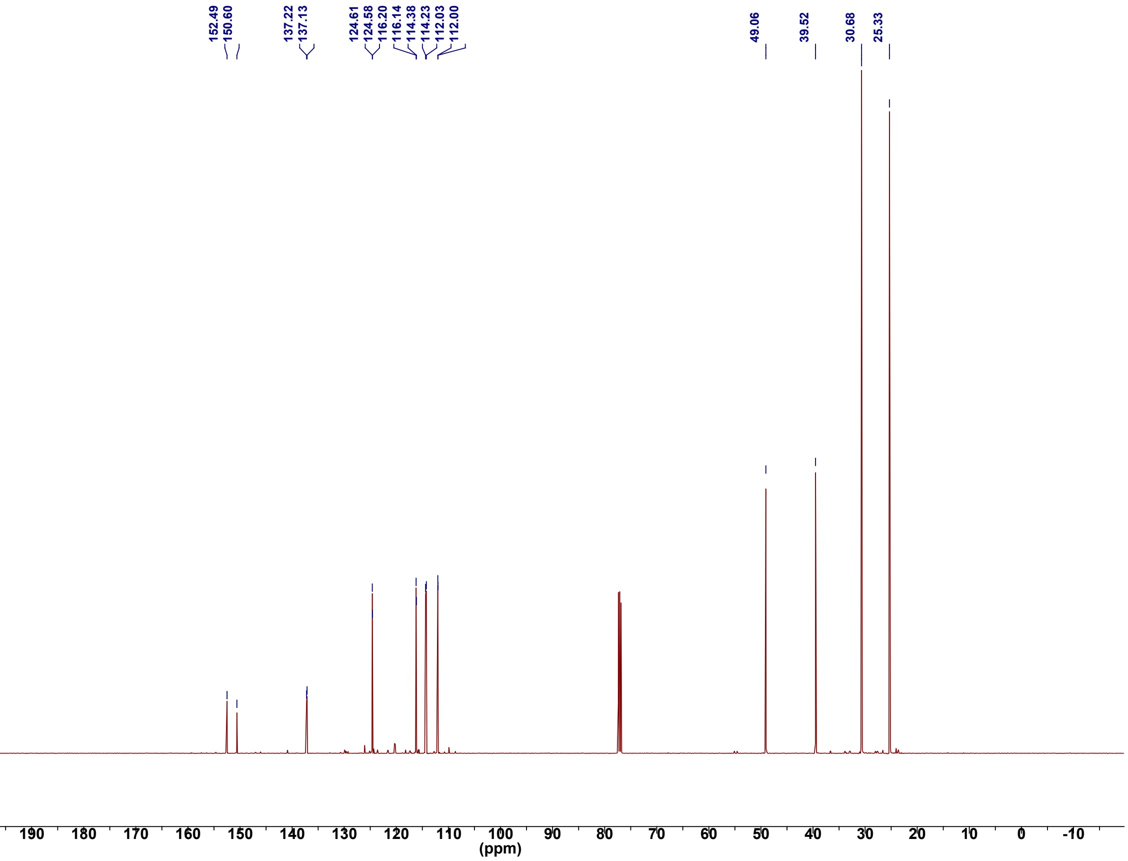

^{19}F NMR ($CDCl_3$, 471 MHz) of P200

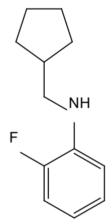

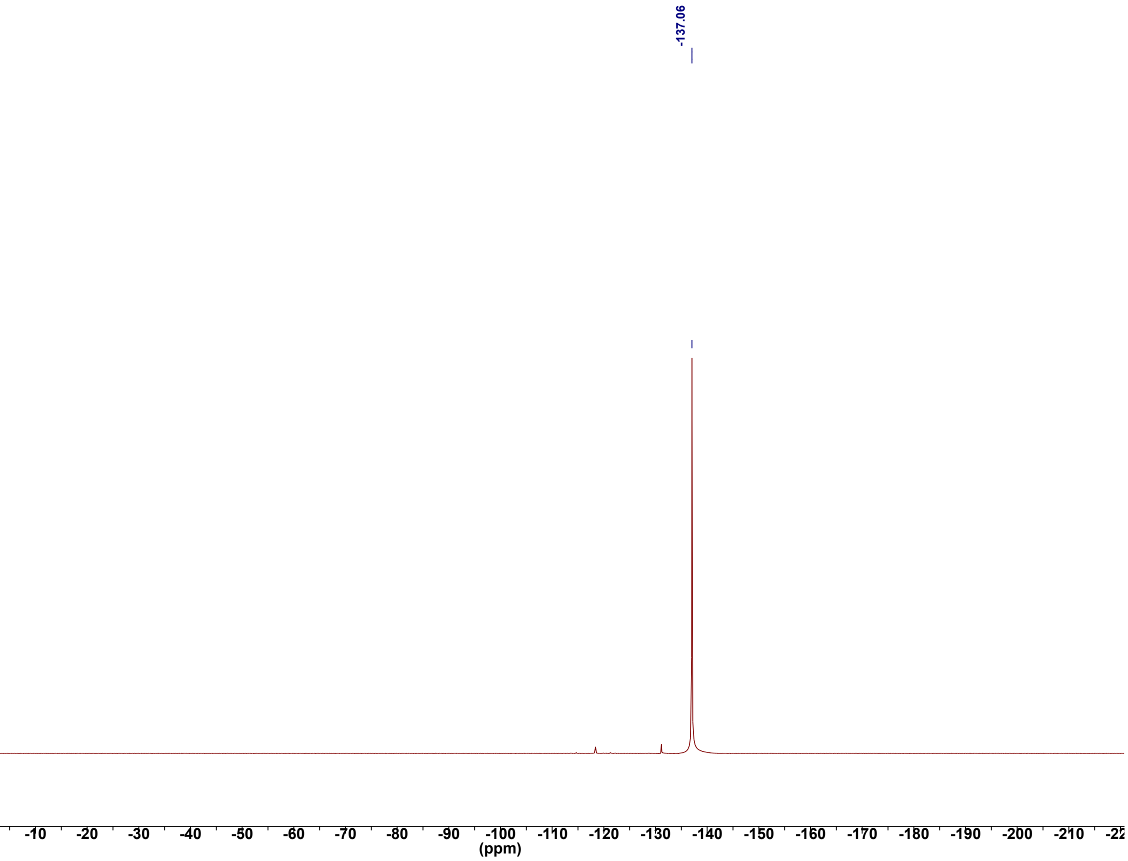

^{1}H NMR ($CDCl_3$, 500 MHz) of P201

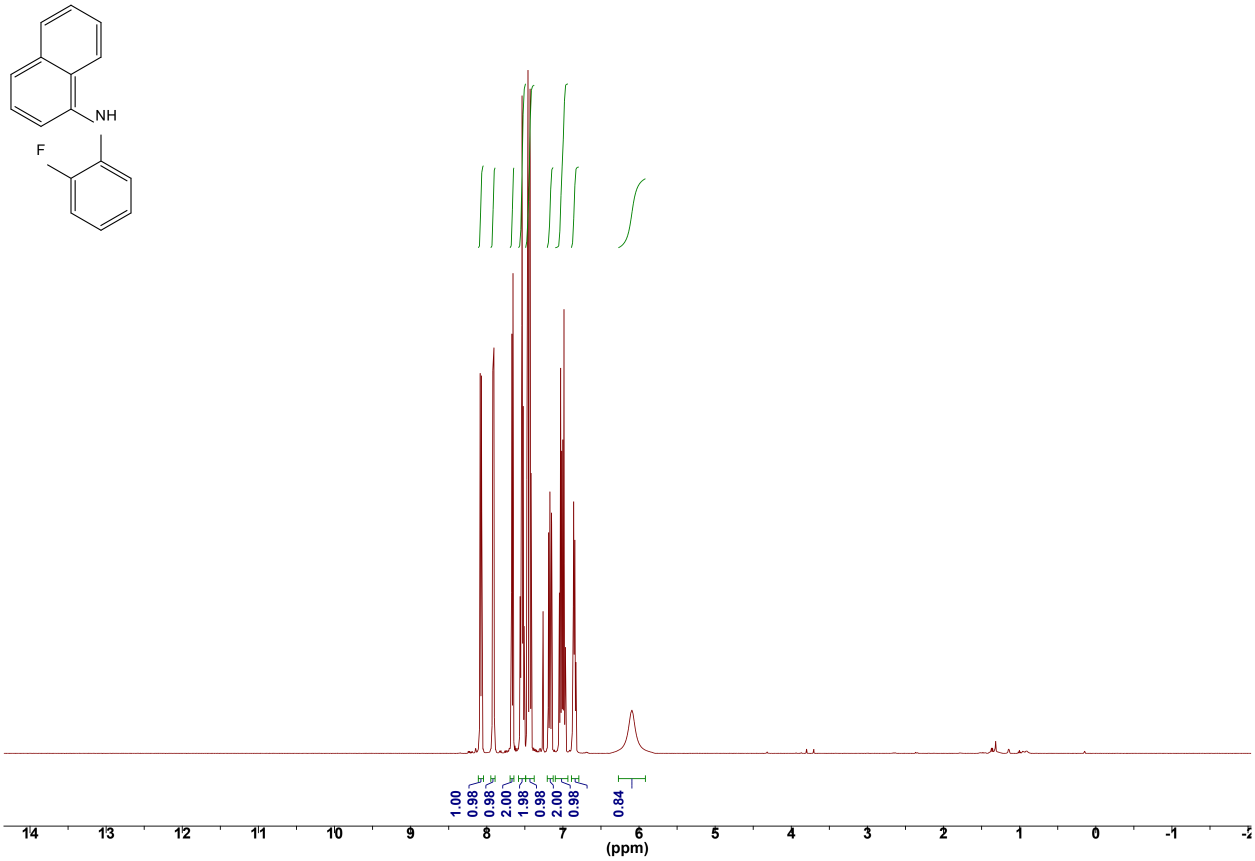

^{13}C NMR ($CDCl_3$, 126 MHz) of P201

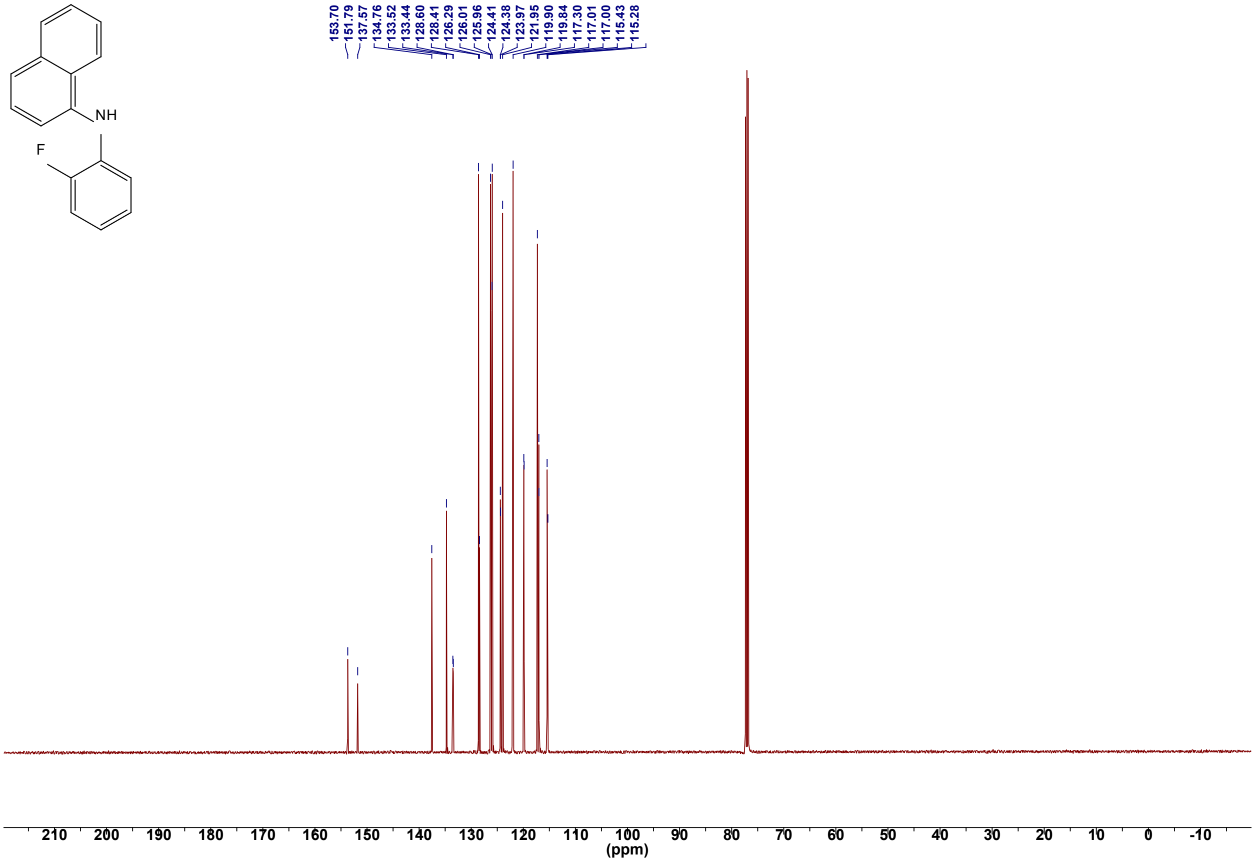

^{19}F NMR ($CDCl_3$, 471 MHz) of P201

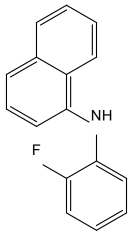

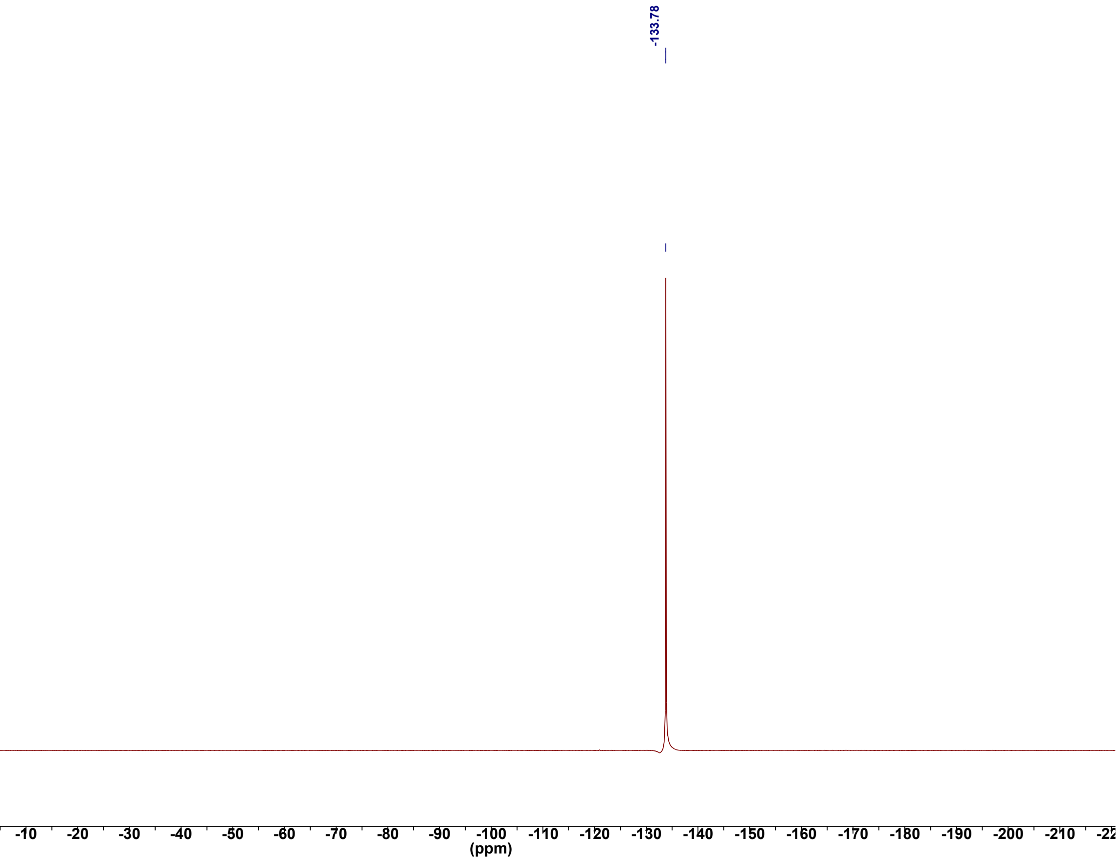

^{1}H NMR ($CDCl_3$, 500 MHz) of P202

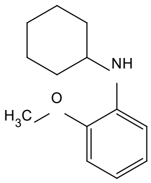

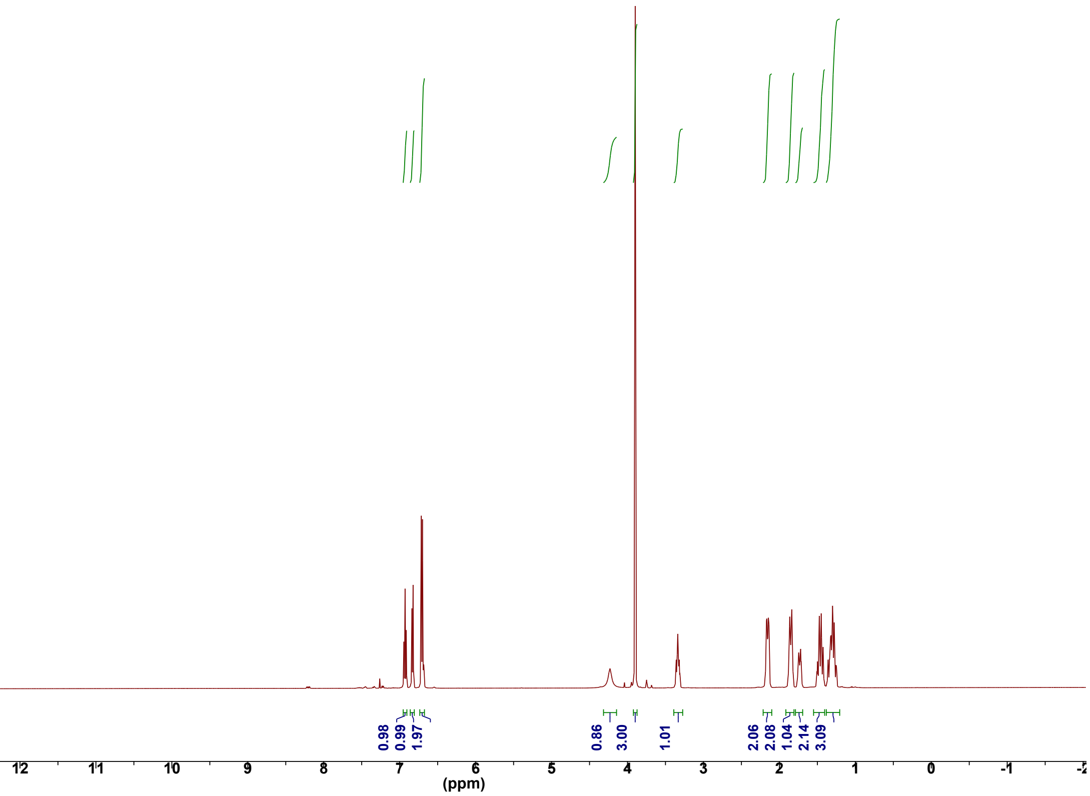

^{13}C NMR ($CDCl_3$, 126 MHz) of P202

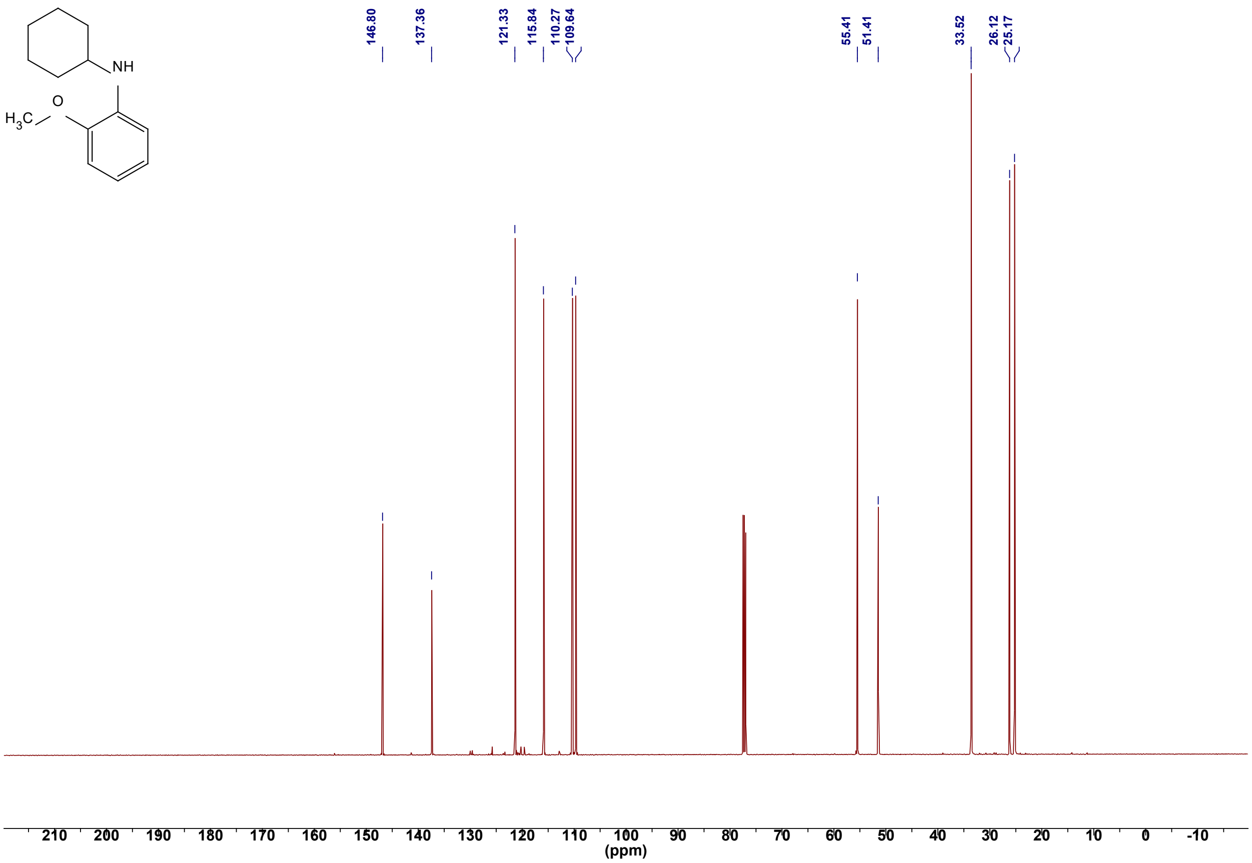

^{1}H NMR ($CDCl_3$, 500 MHz) of P203

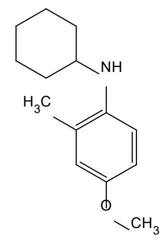

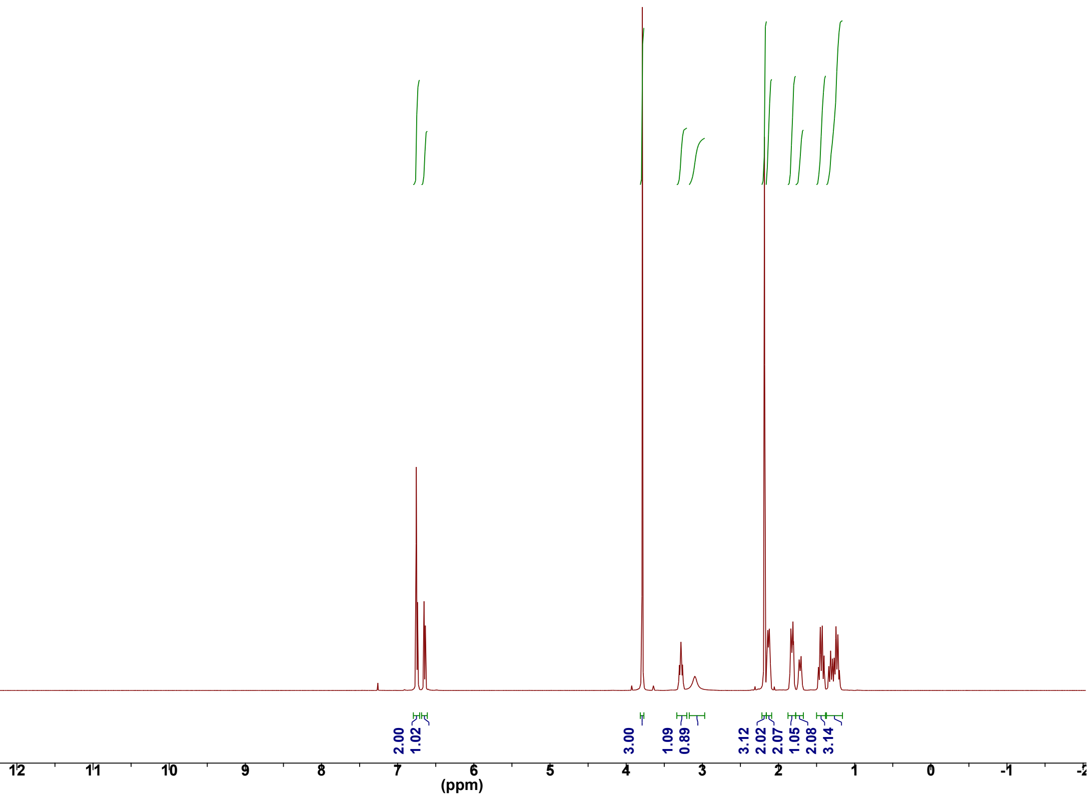

^{13}C NMR ($CDCl_3$, 126 MHz) of P203

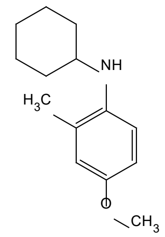

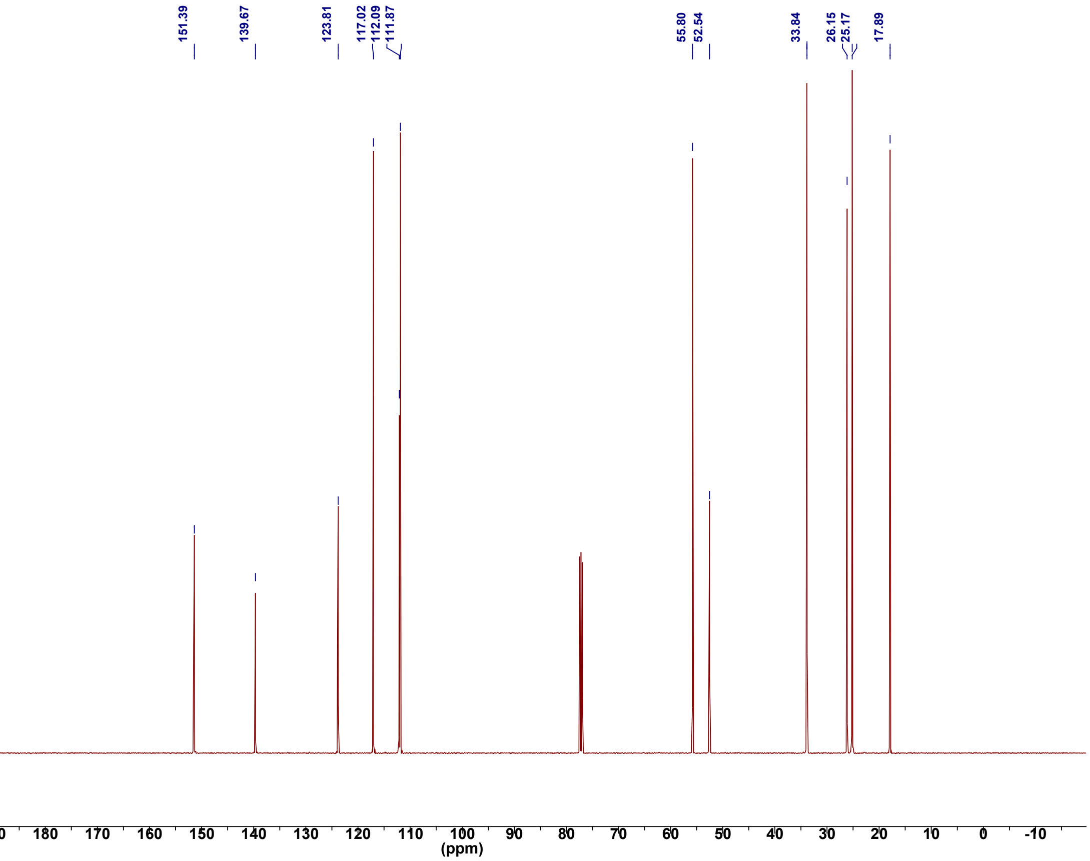

^{1}H NMR ($CDCl_3$, 500 MHz) of P204

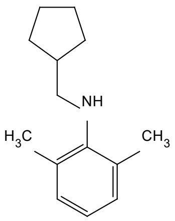

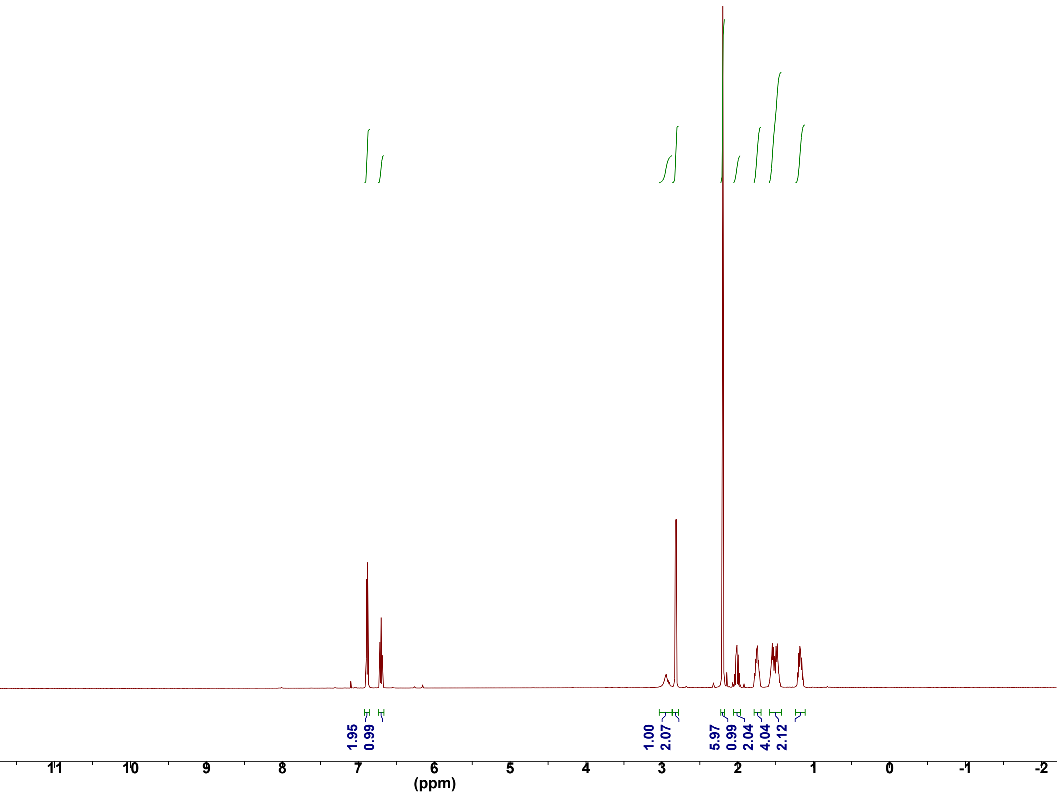

^{13}C NMR ($CDCl_3$, 126 MHz) of P204

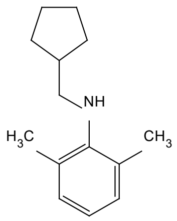

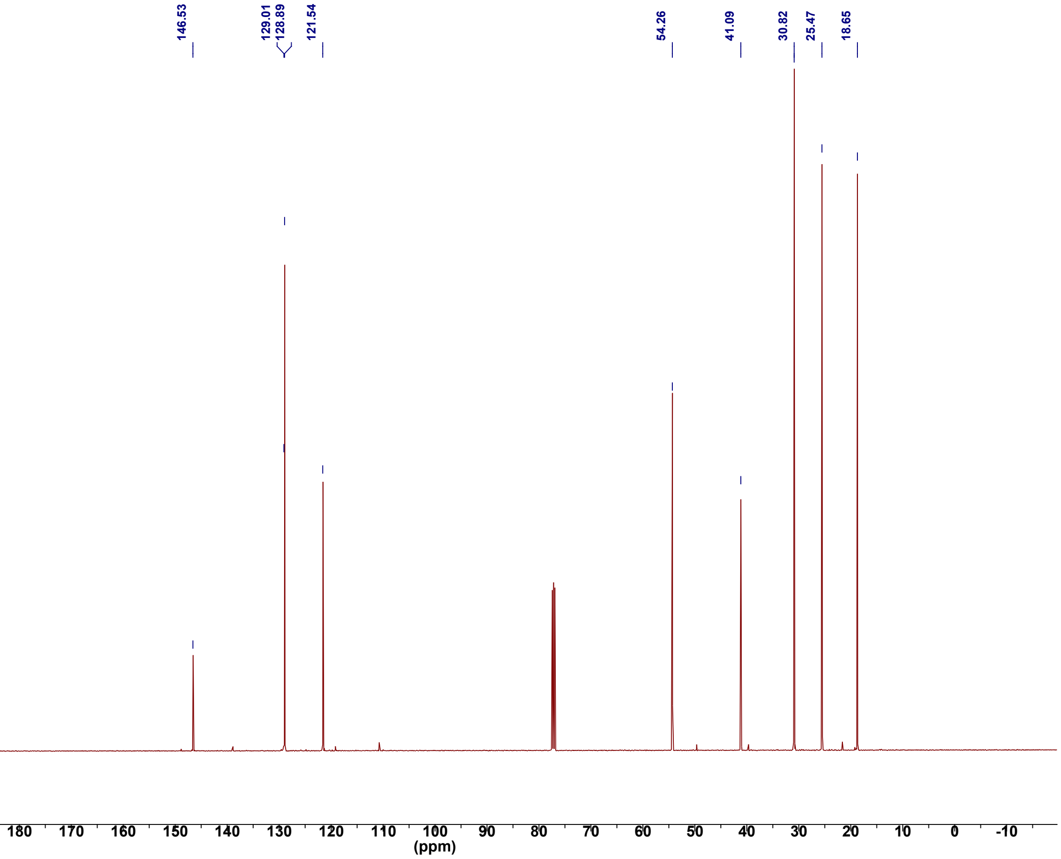

^{1}H NMR ($CDCl_3$, 500 MHz) of P205

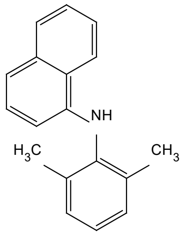

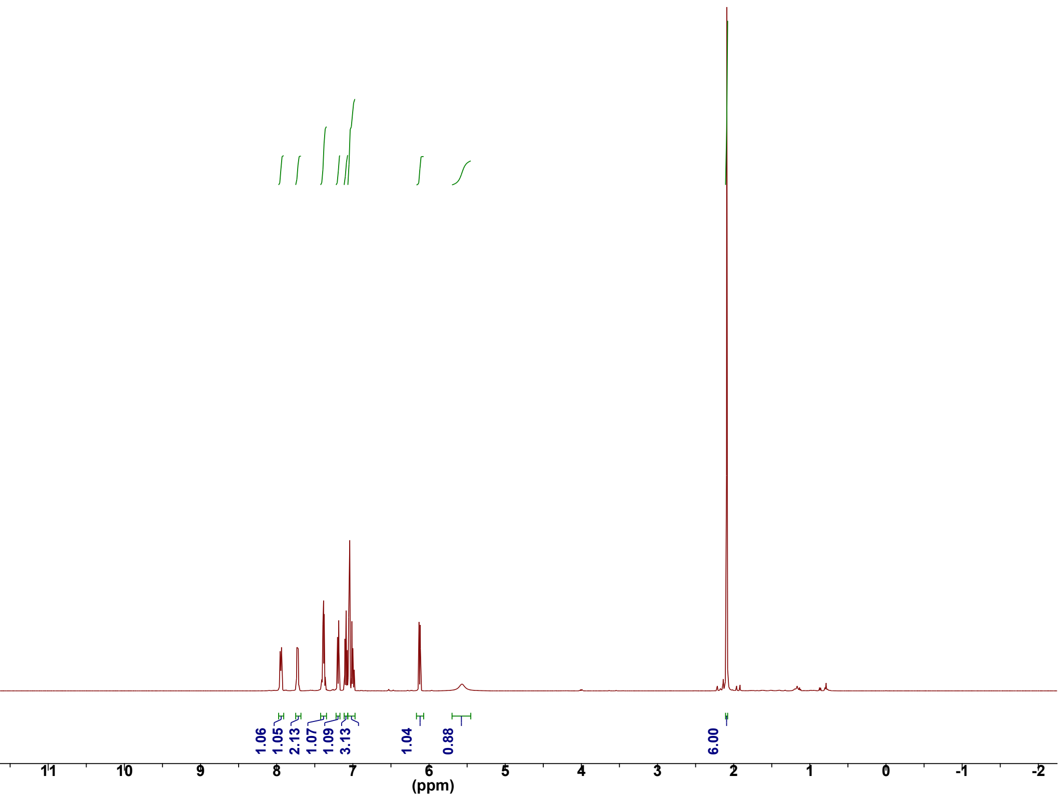

^{13}C NMR ($CDCl_3$, 126 MHz) of P205

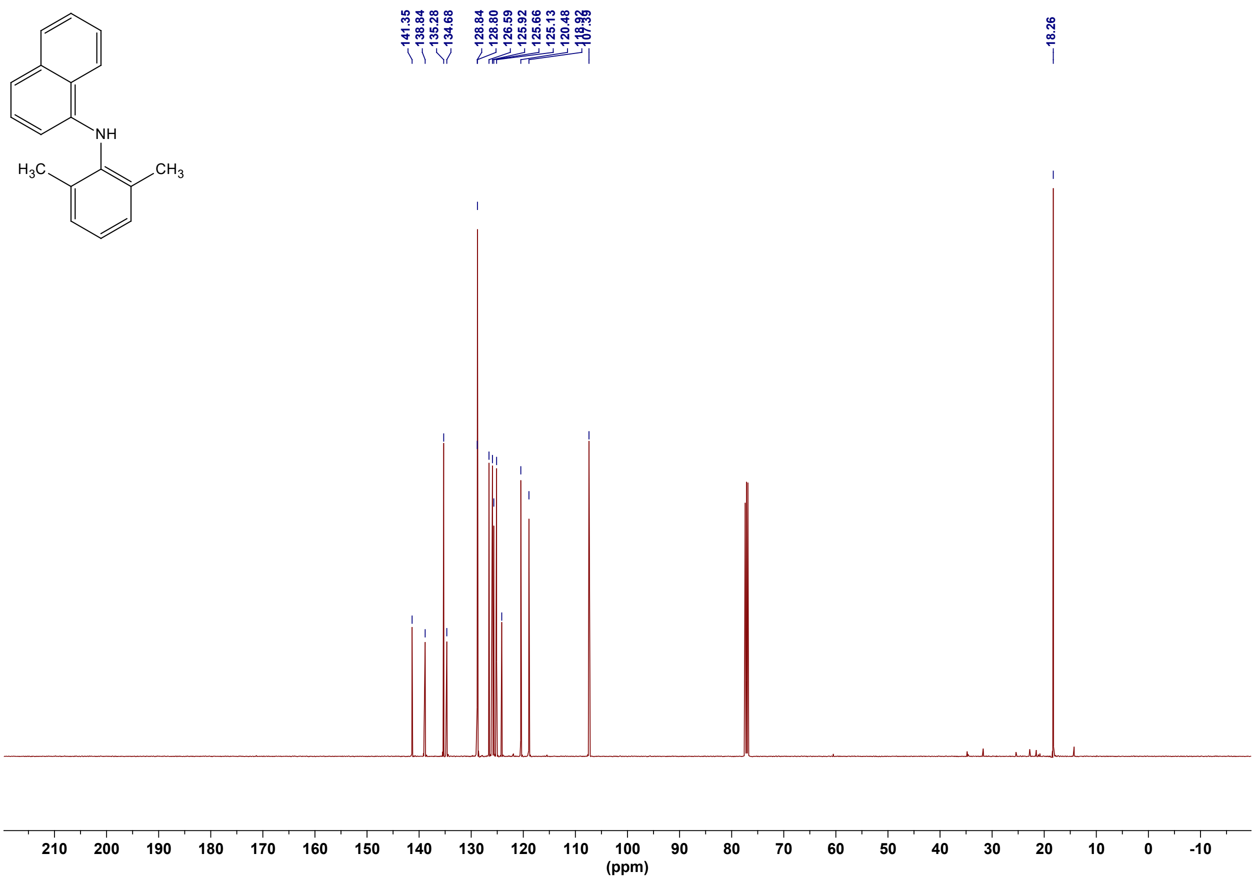

^{1}H NMR ($CDCl_3$, 500 MHz) of P206

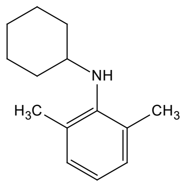

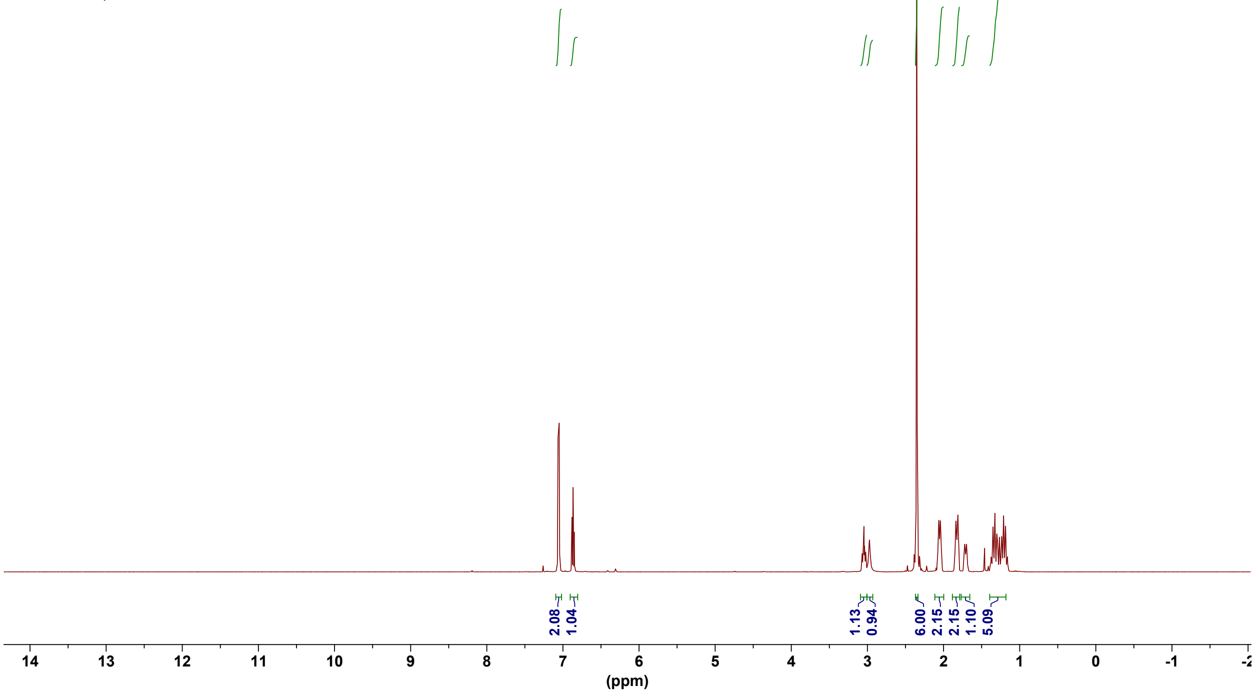

^{13}C NMR ($CDCl_3$, 126 MHz) of P206

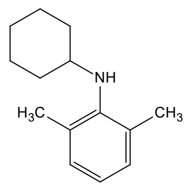

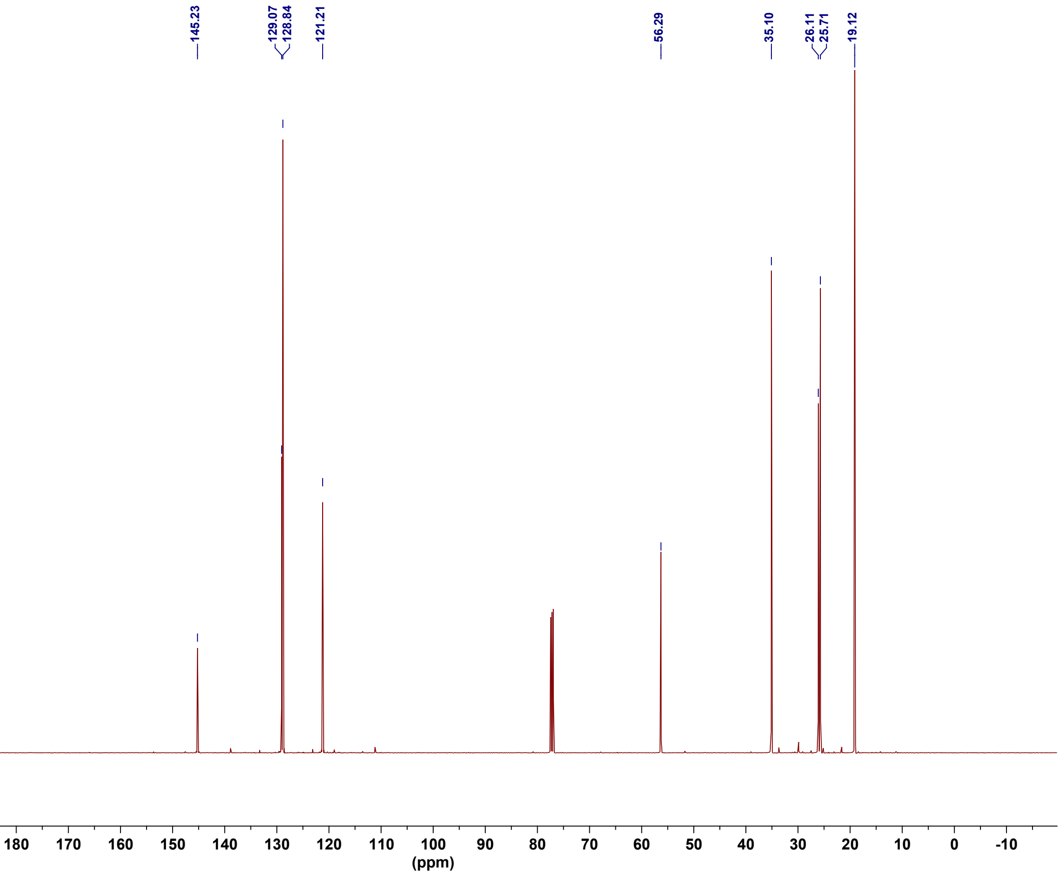

^{1}H NMR ($CDCl_3$, 500 MHz) of P207

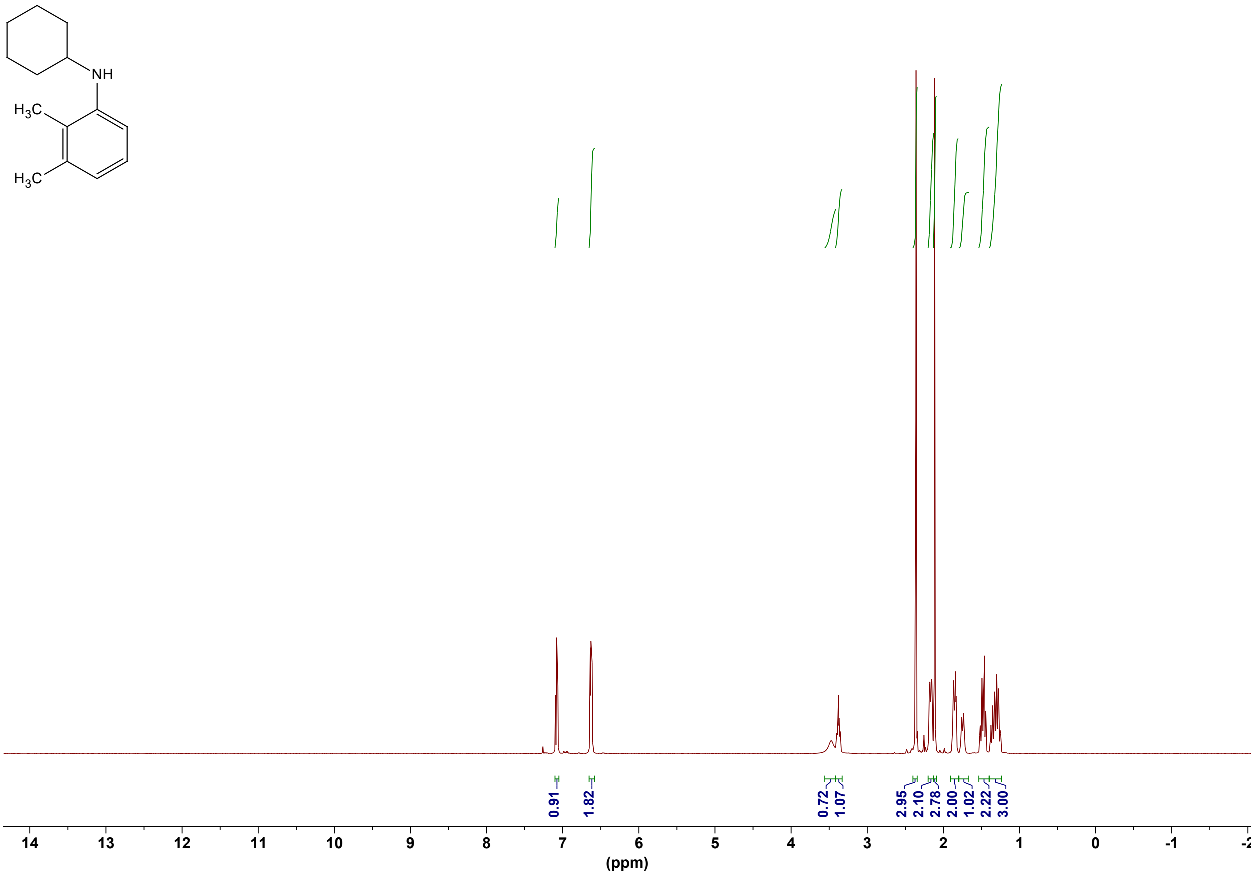

^{13}C NMR ($CDCl_3$, 126 MHz) of P207

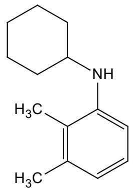

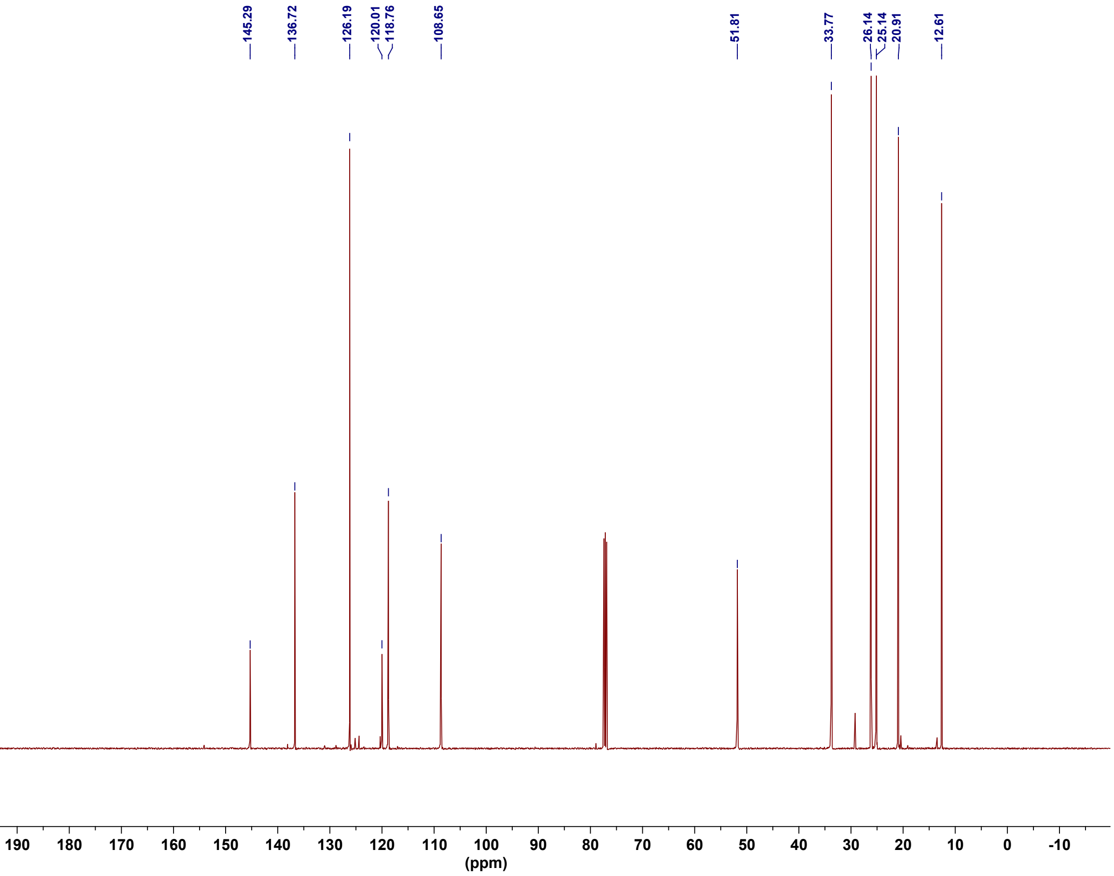

^{1}H NMR ($CDCl_3$, 500 MHz) of P208

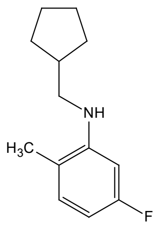

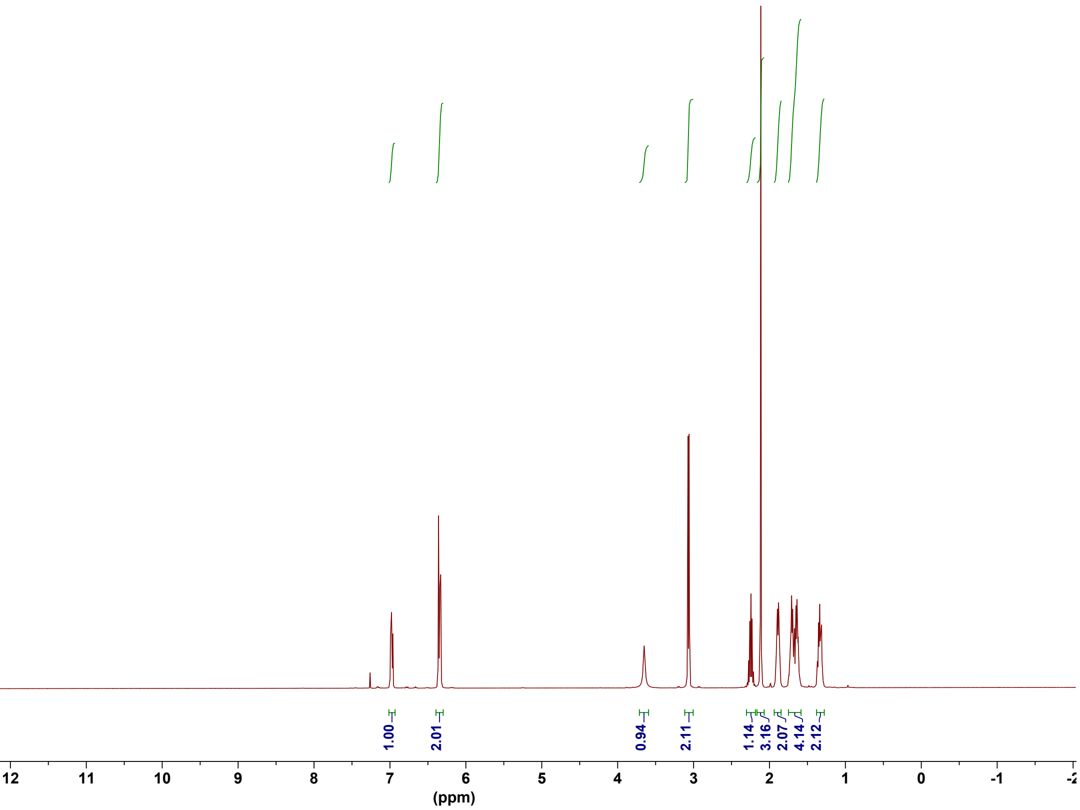

^{13}C NMR ($CDCl_3$, 126 MHz) of P208

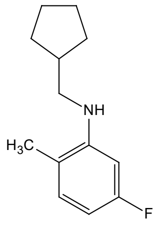

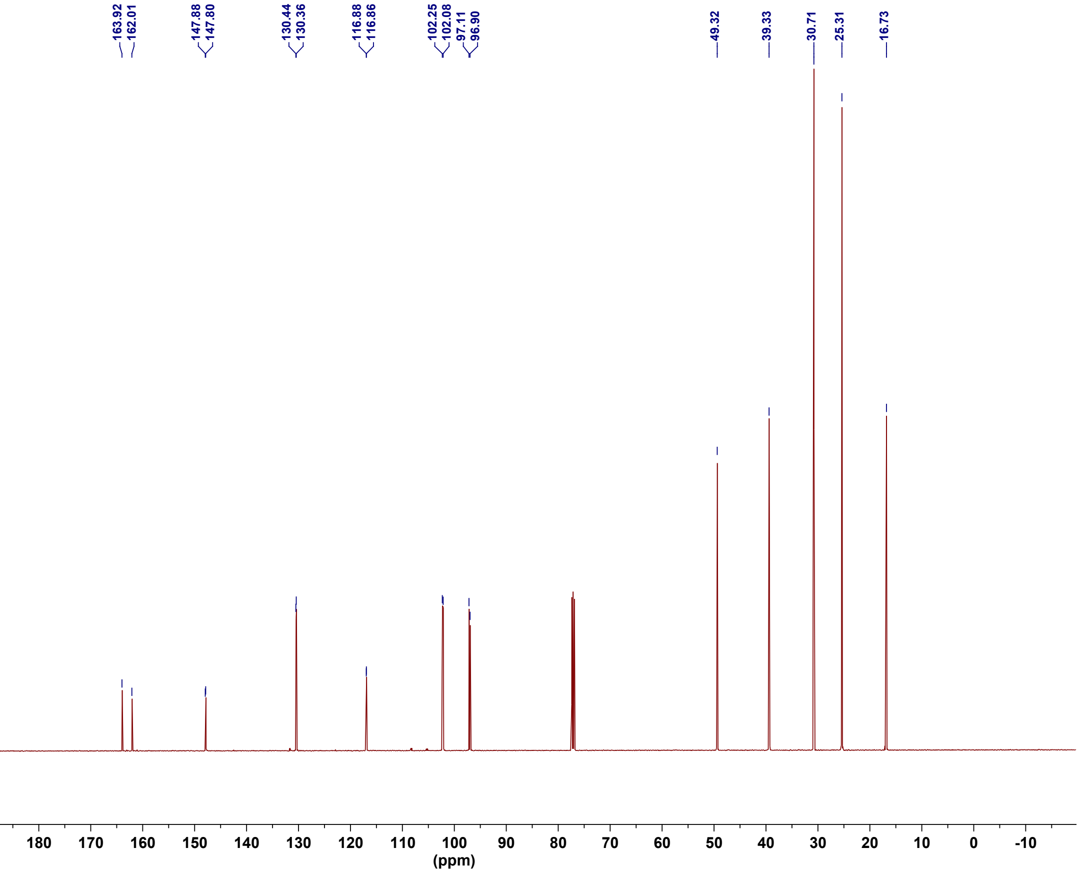

^{19}F NMR (CDCl$_3$, 471 MHz) of P208

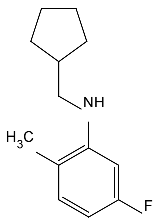

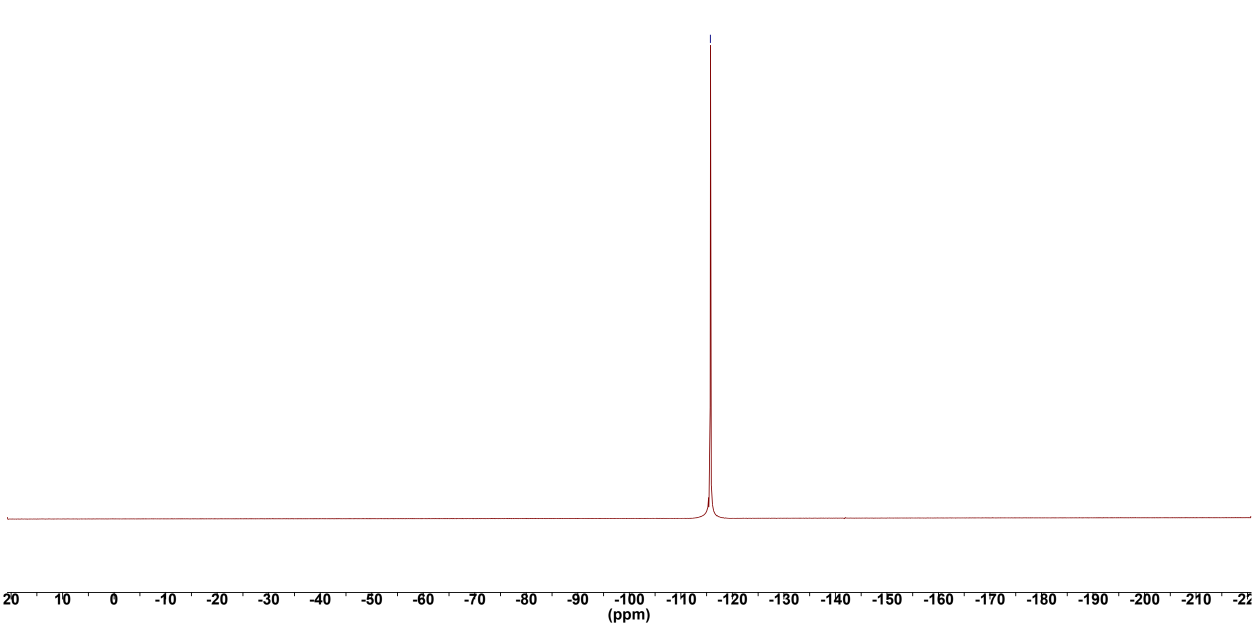

^{1}H NMR ($CDCl_3$, 500 MHz) of P209

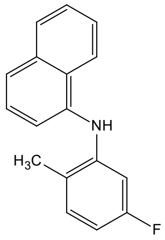

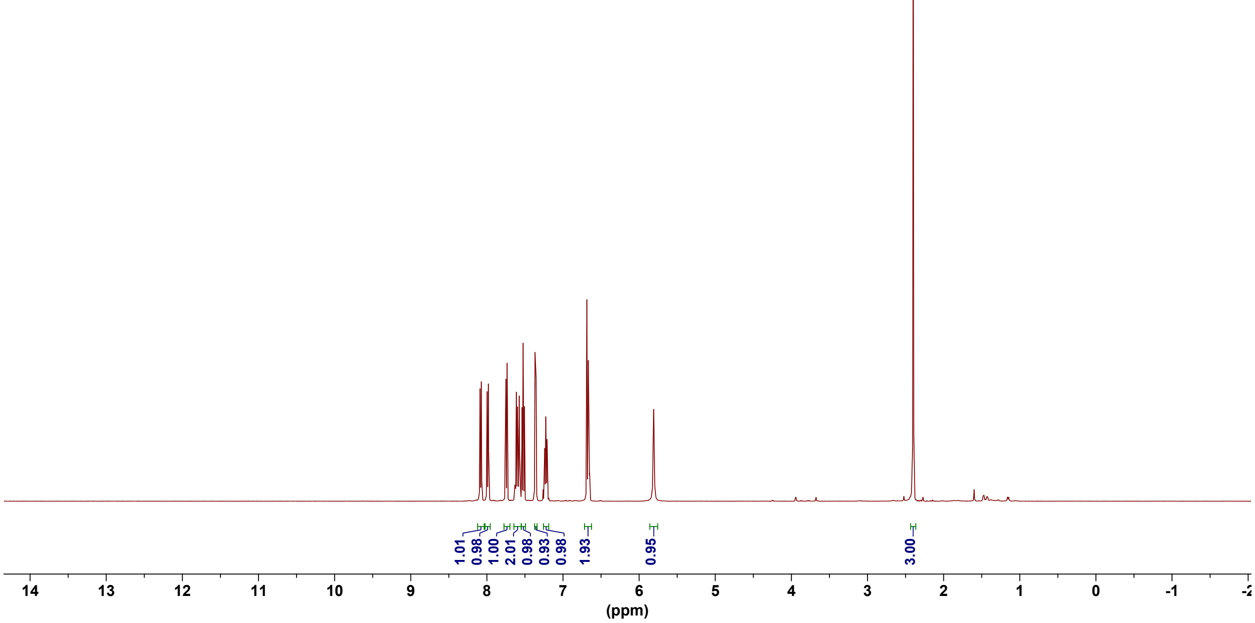

^{13}C NMR ($CDCl_3$, 126 MHz) of P209

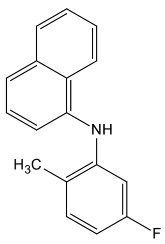

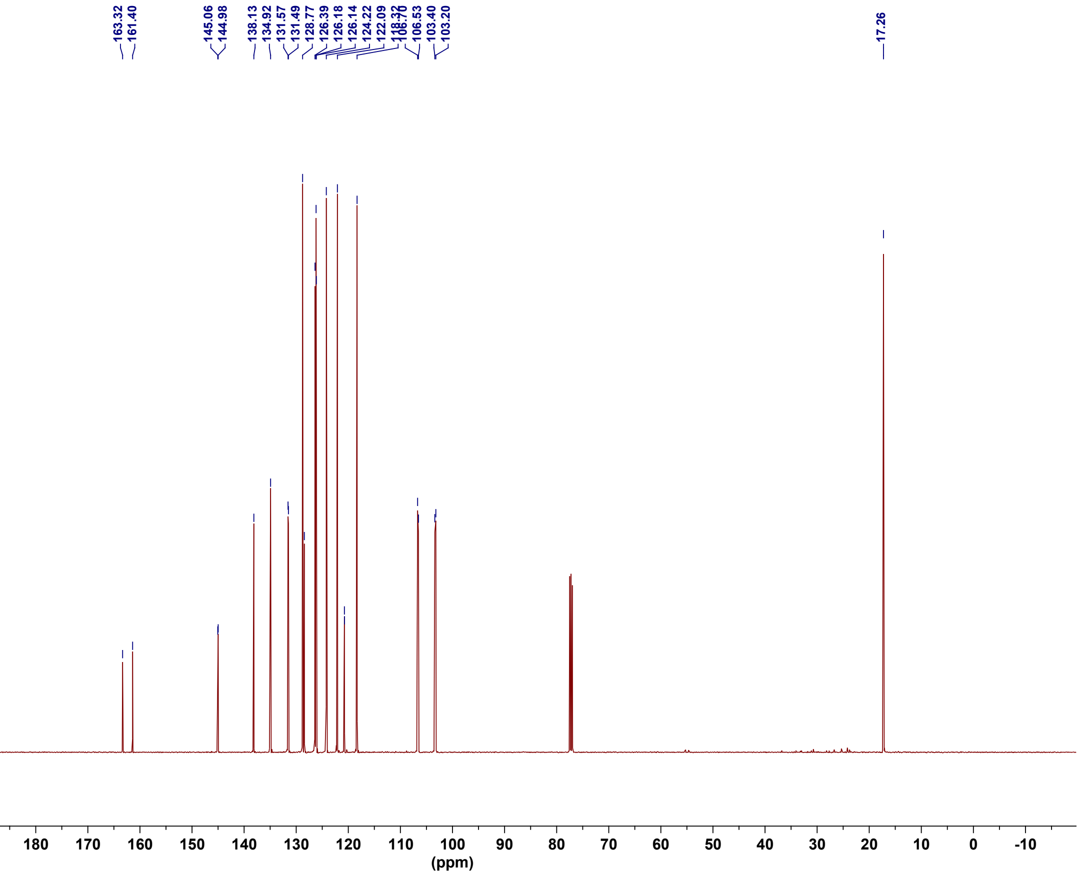

^{19}F NMR ($CDCl_3$, 471 MHz) of P209

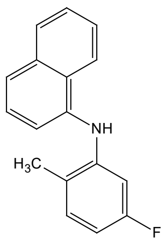

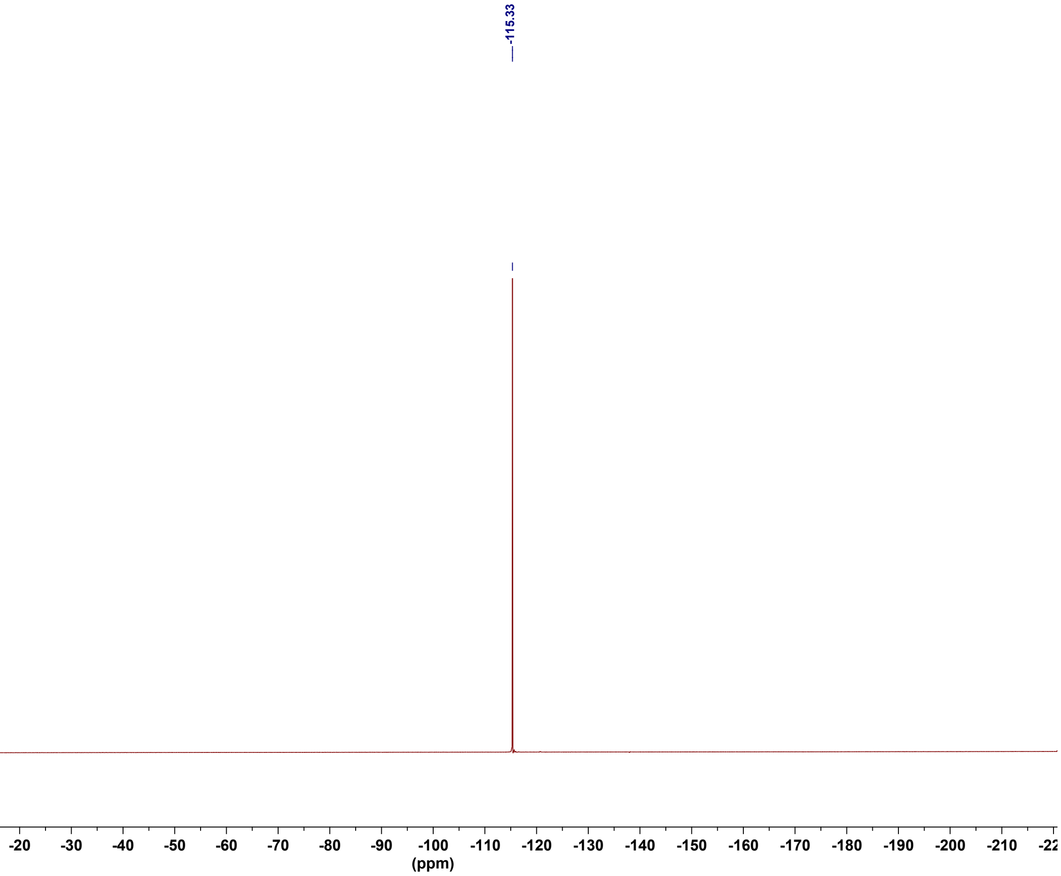

^{1}H NMR ($CDCl_3$, 500 MHz) of P210

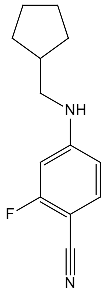

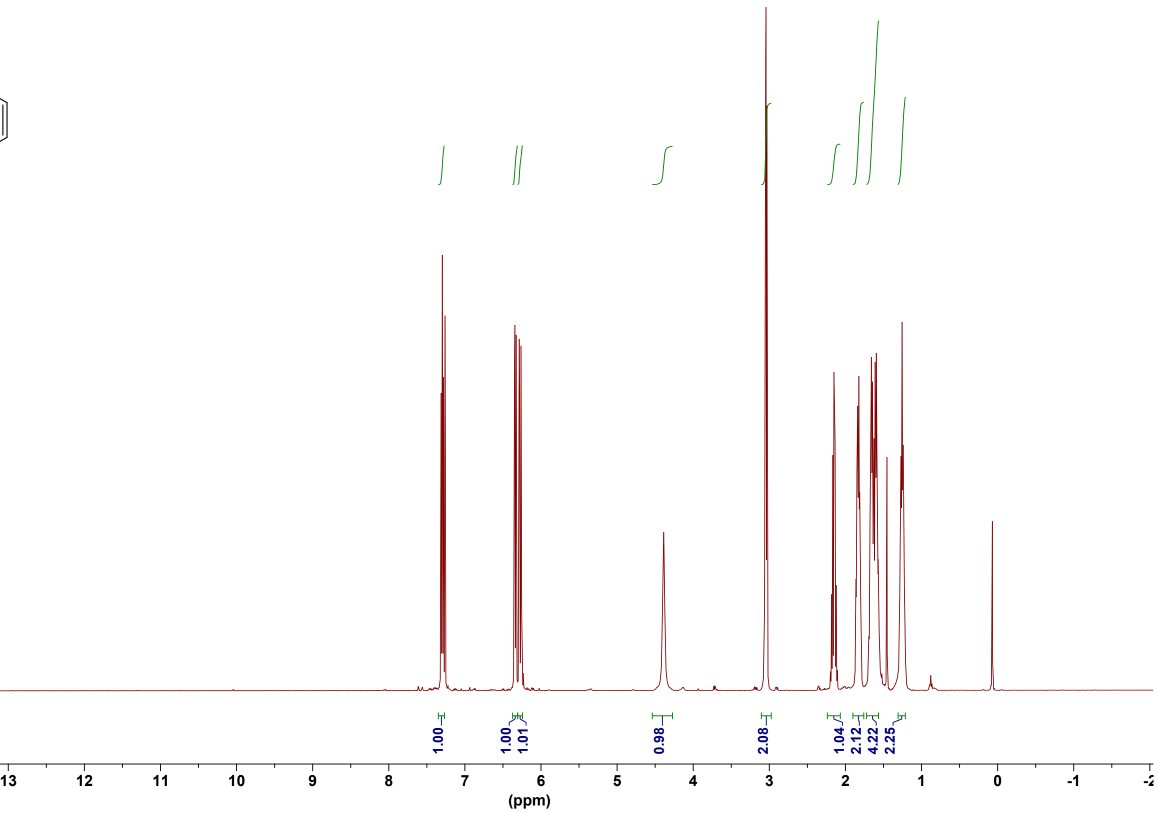

^{13}C NMR ($CDCl_3$, 126 MHz) of P210

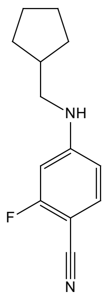

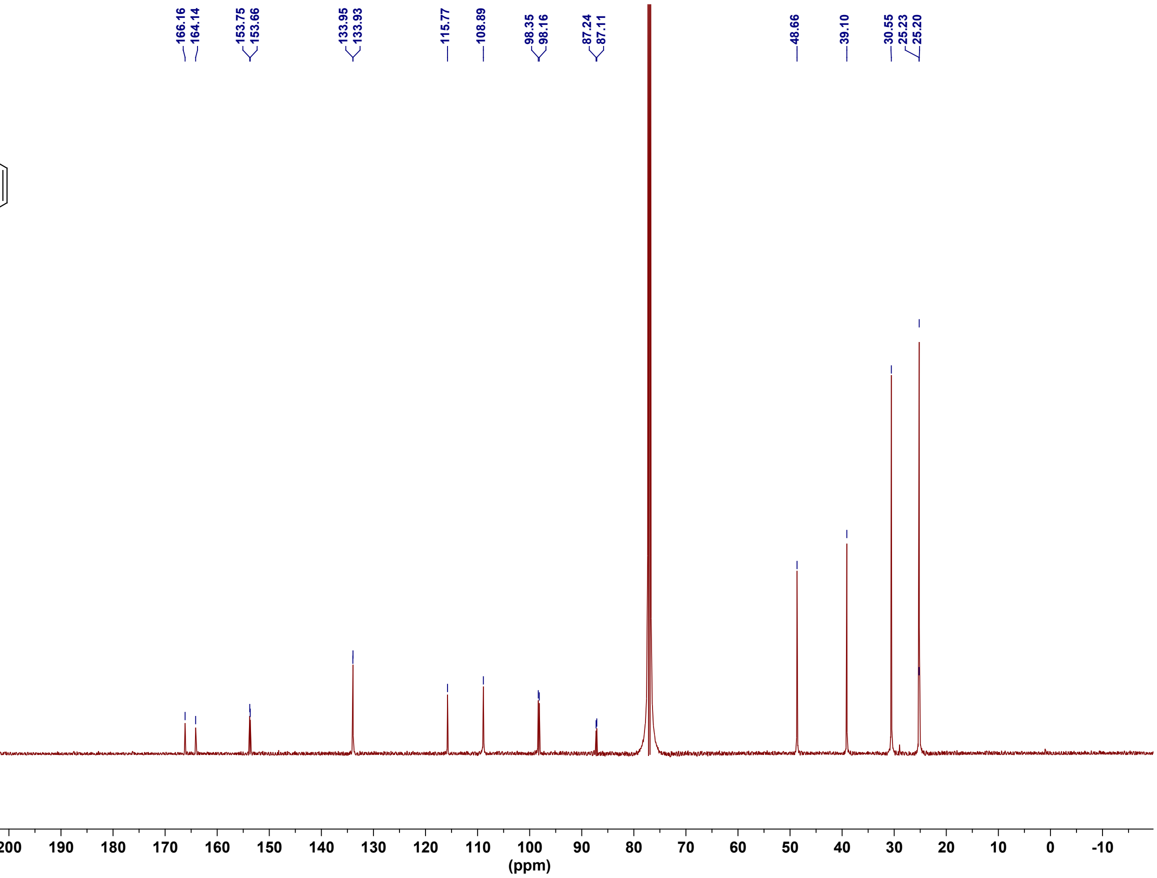

^{19}F NMR ($CDCl_3$, 471 MHz) of P210

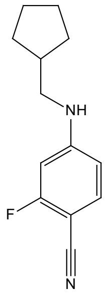

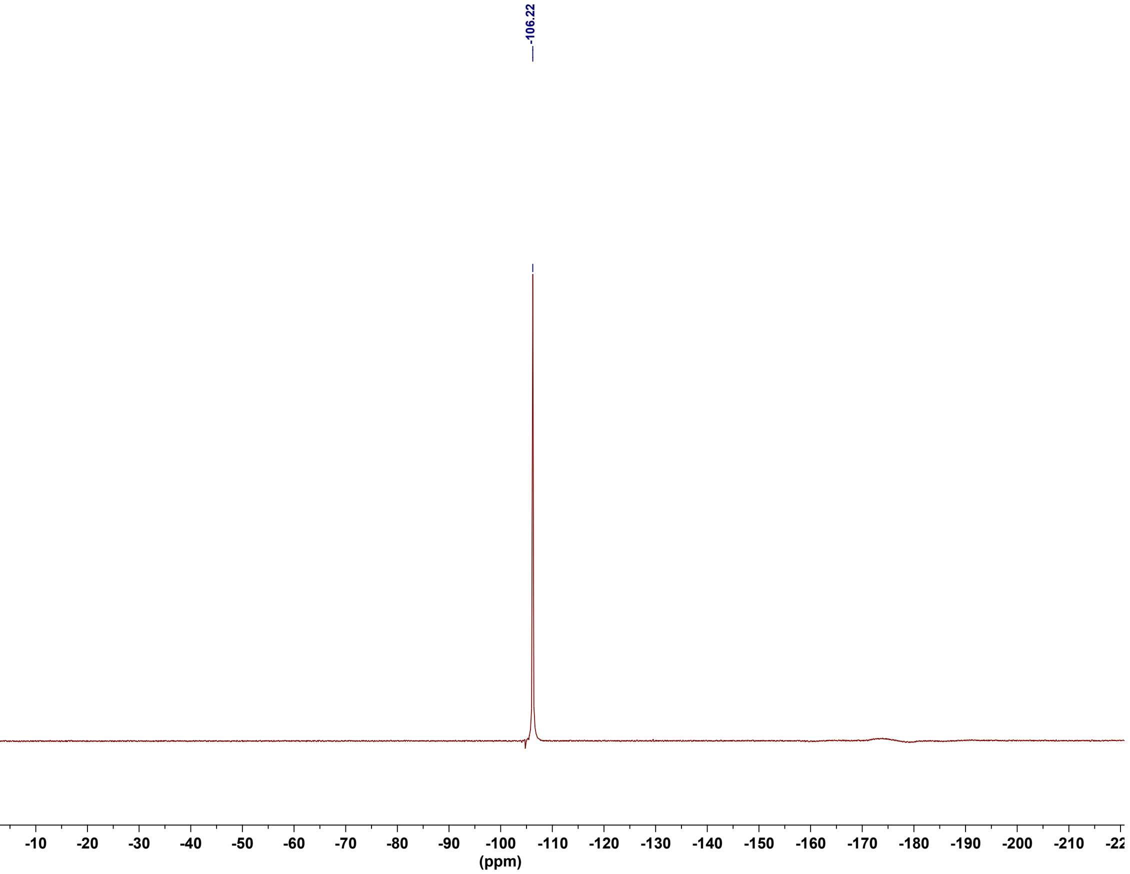

^1H NMR ($CDCl_3$, 500 MHz) of P211

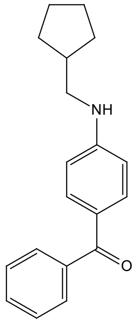

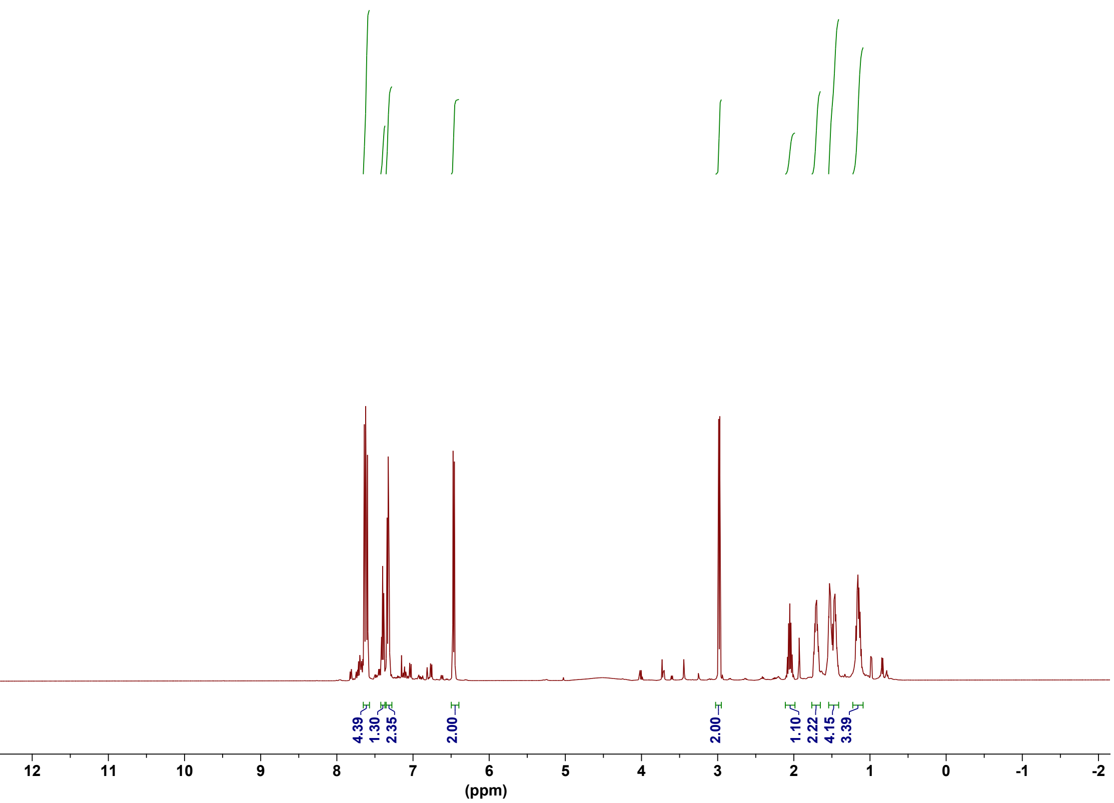

^{13}C NMR ($CDCl_3$, 126 MHz) of P211

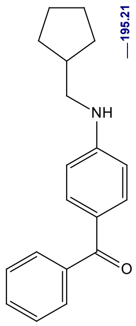

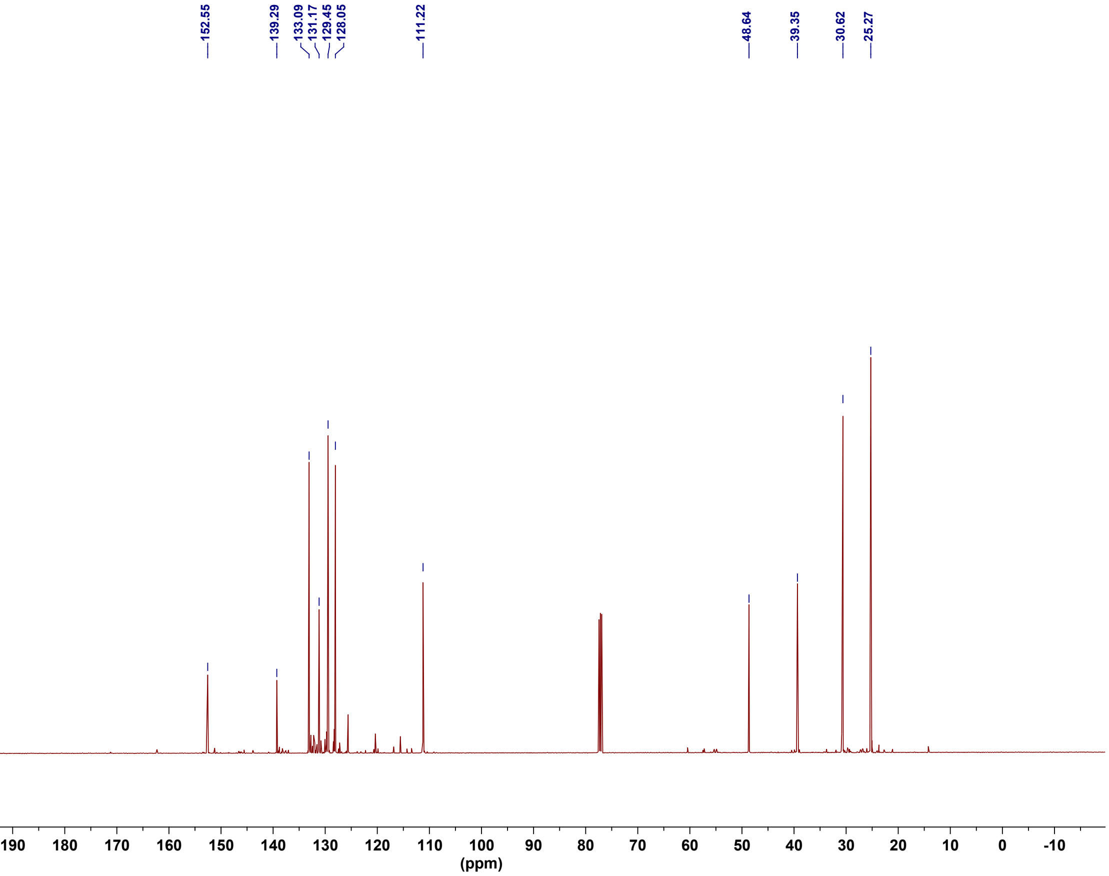

^{1}H NMR ($CDCl_3$, 500 MHz) of P212

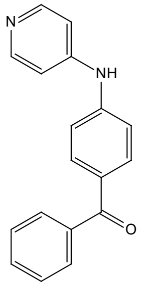

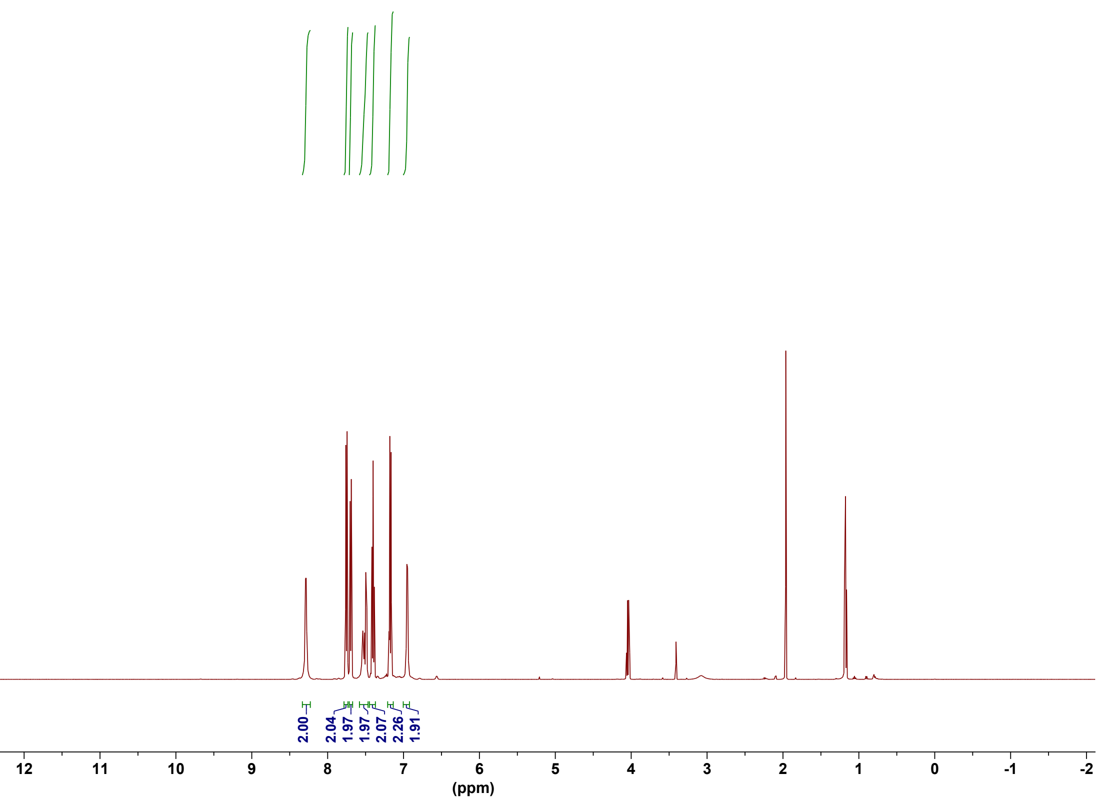

^{13}C NMR ($CDCl_3$, 126 MHz) of P212

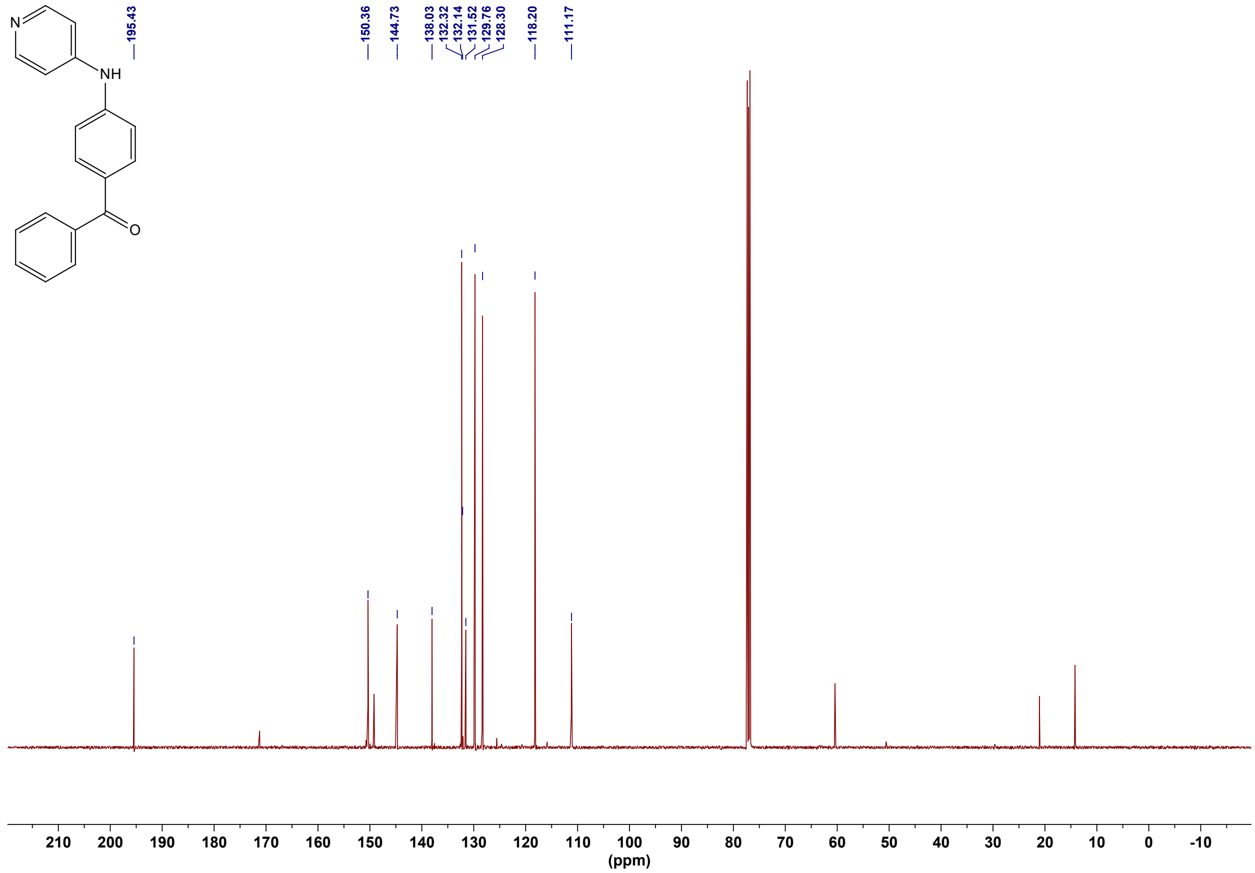

^{1}H NMR ($CDCl_3$, 500 MHz) of P213

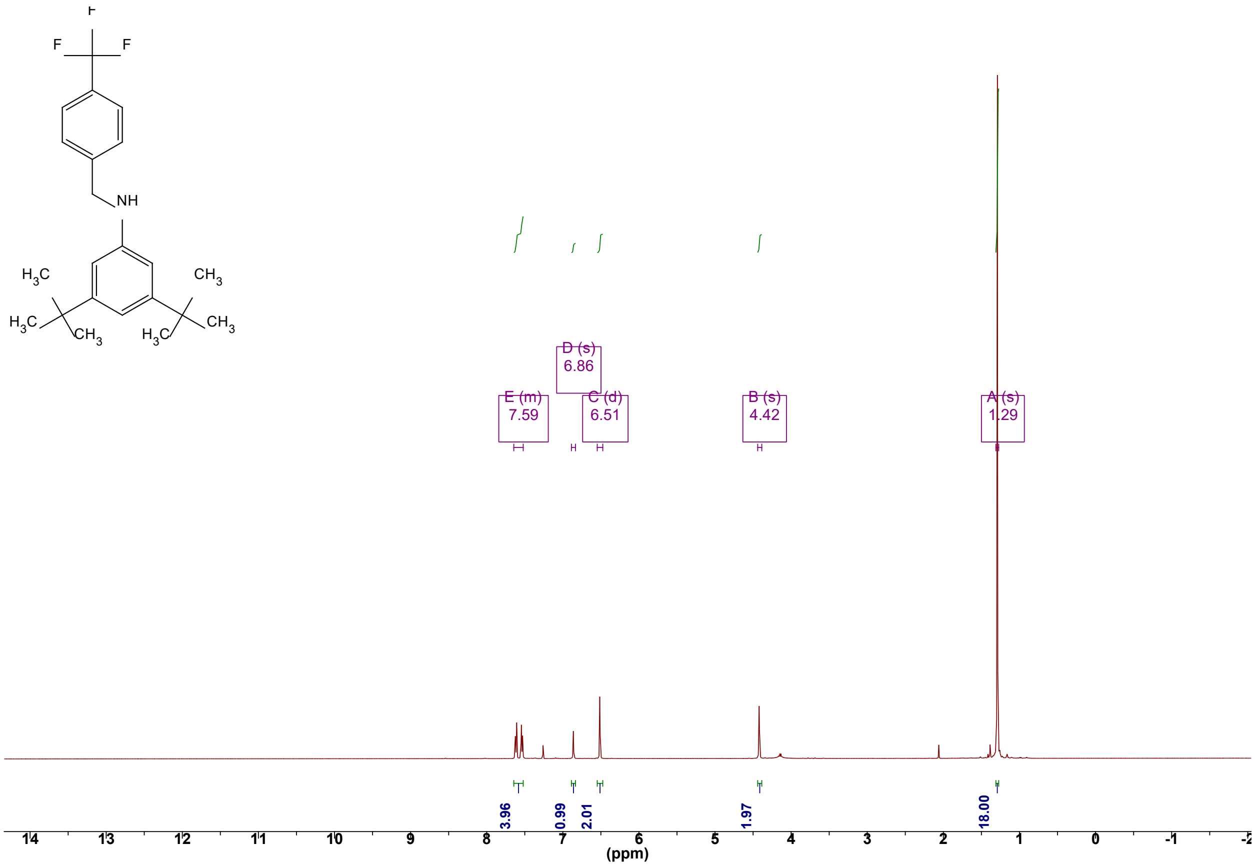

^{13}C NMR ($CDCl_3$, 126 MHz) of P213

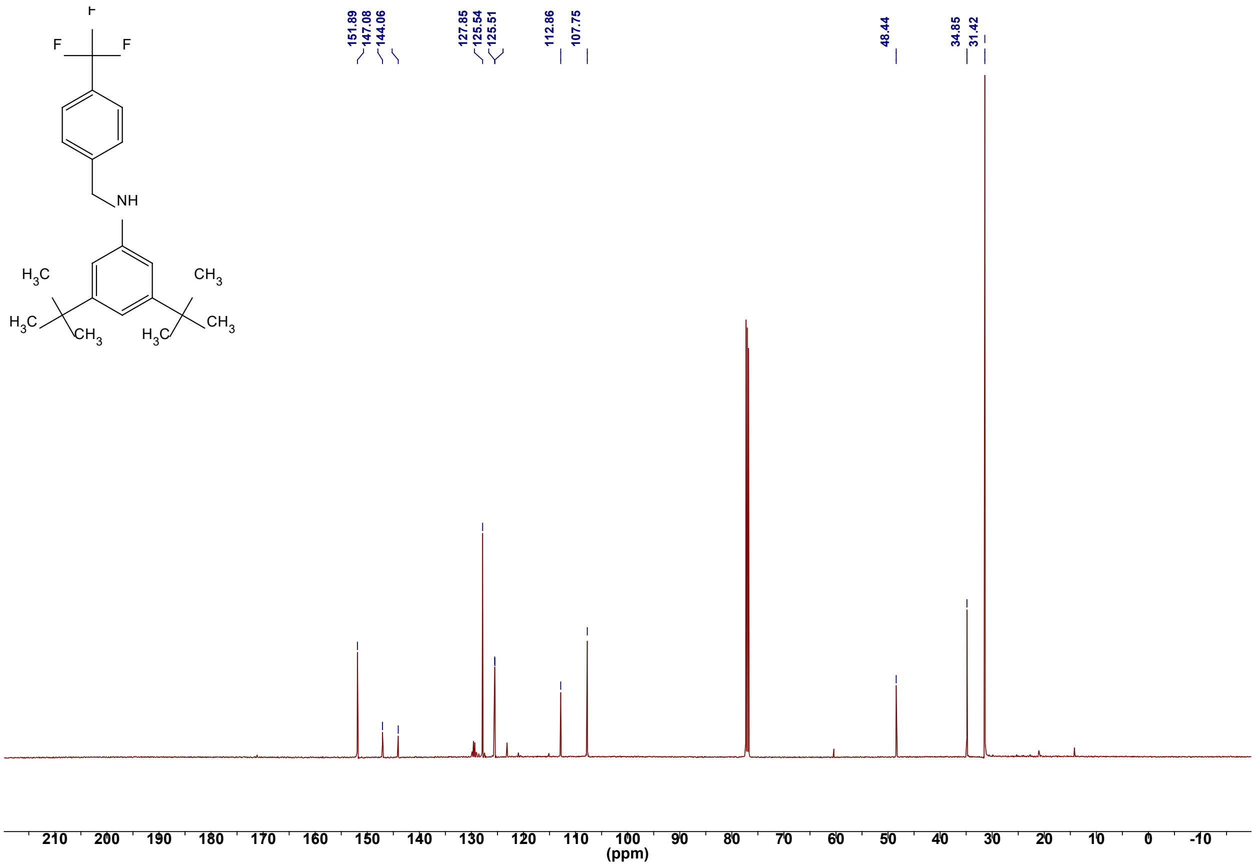

^{19}F NMR ($CDCl_3$, 471 MHz) of P213

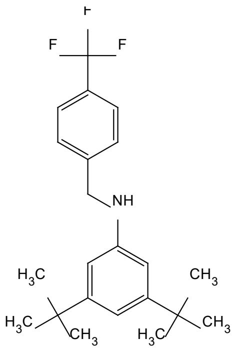

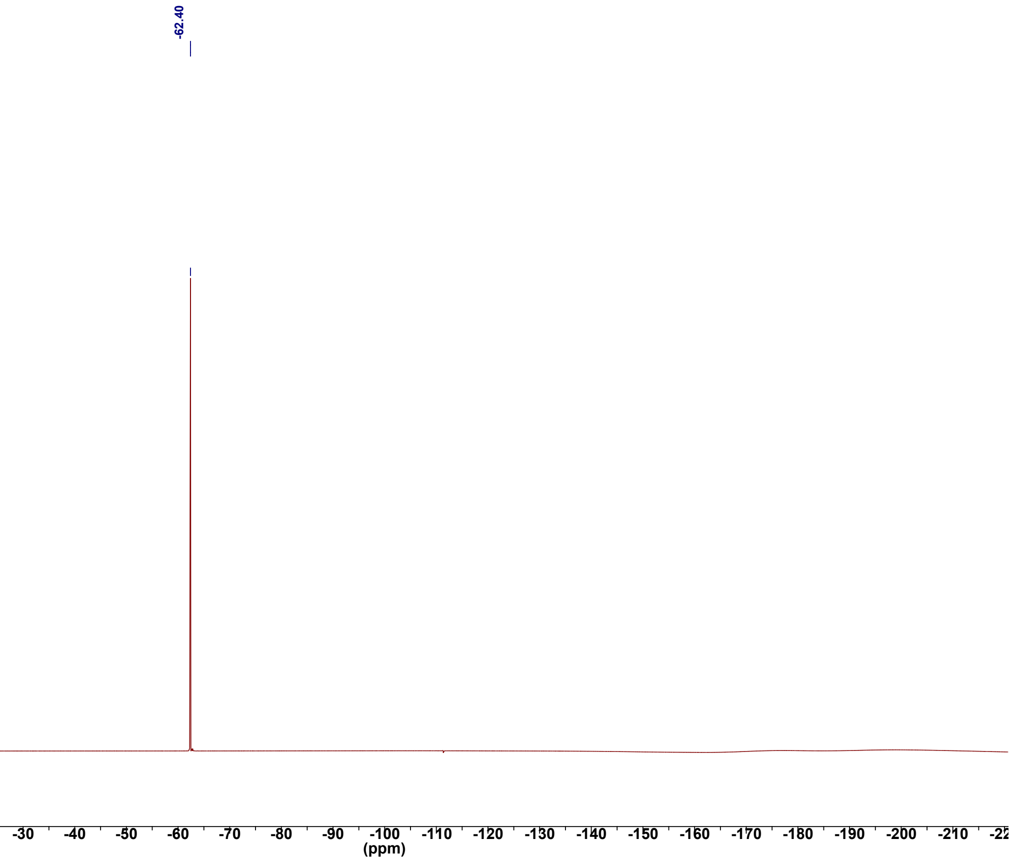

^{1}H NMR ($CDCl_3$, 500 MHz) of P214

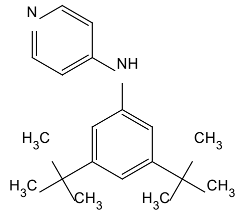

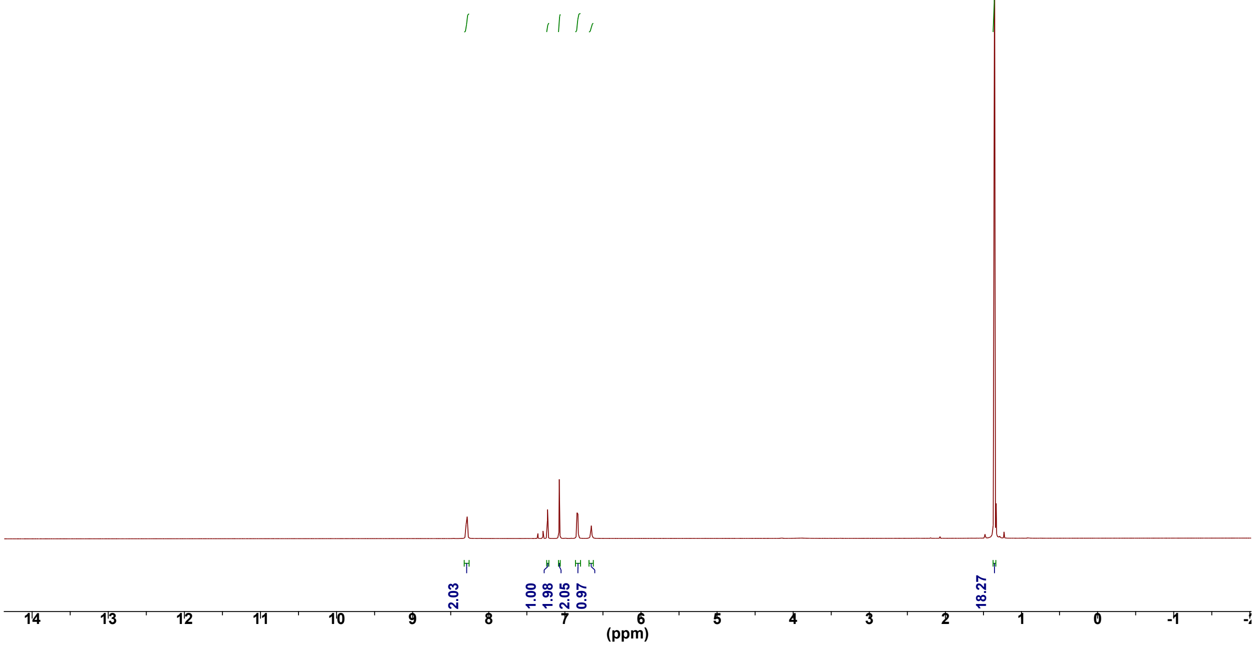

^{13}C NMR ($CDCl_3$, 126 MHz) of P214

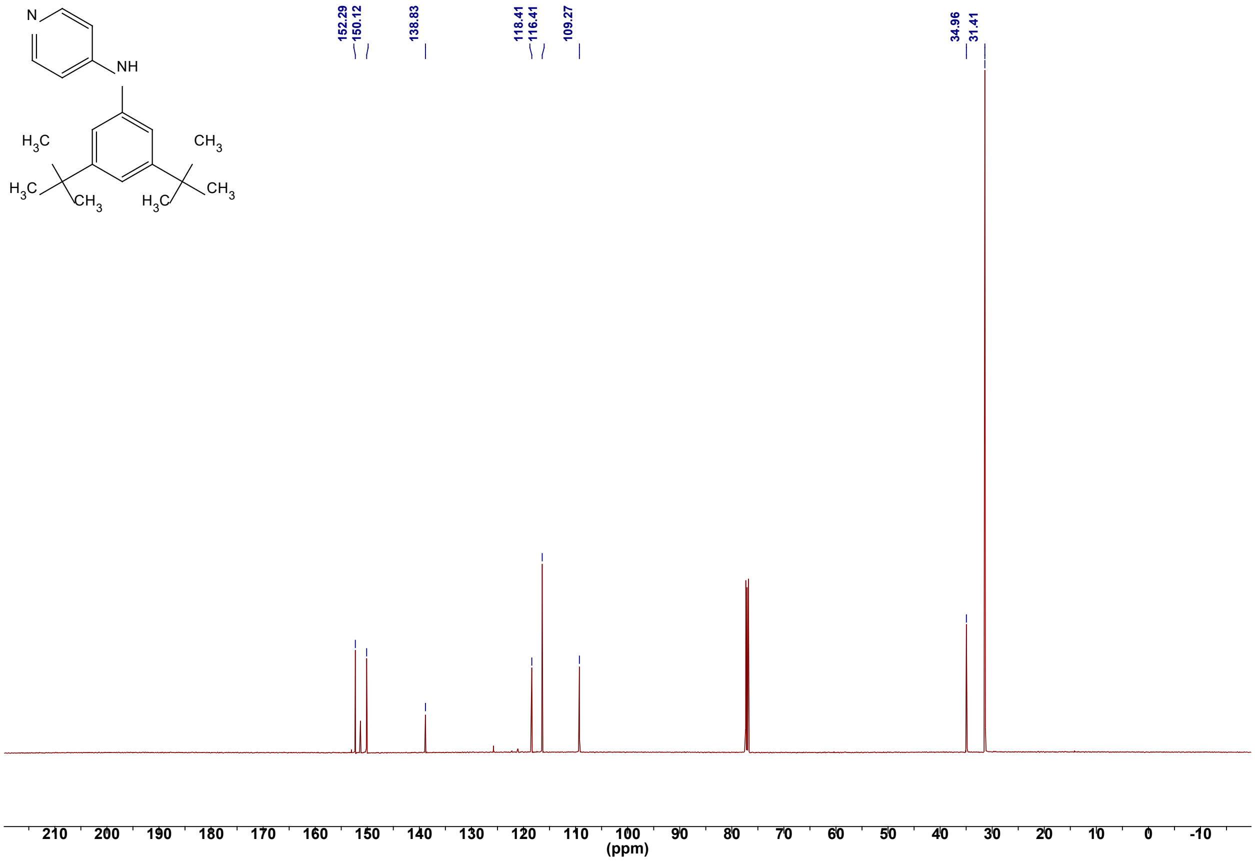

^{1}H NMR ($CDCl_3$, 500 MHz) of P215

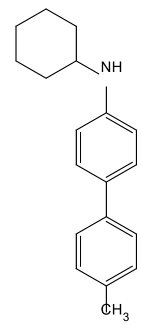

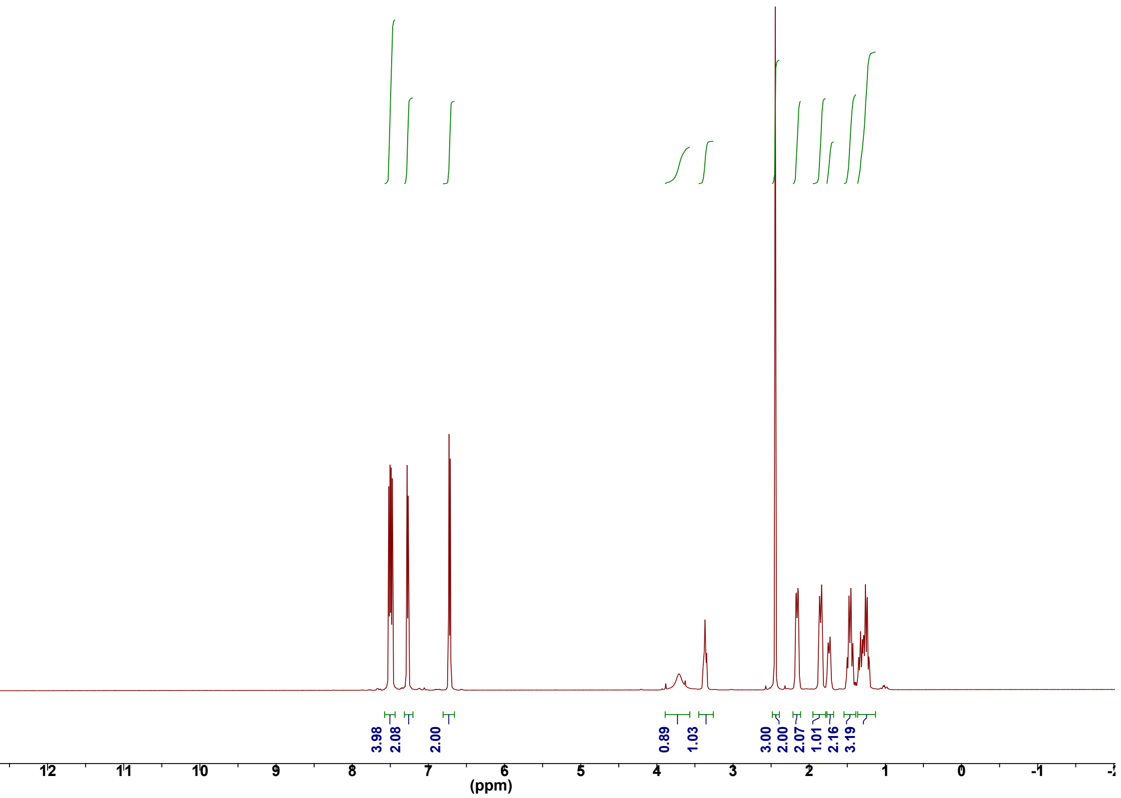

^{13}C NMR ($CDCl_3$, 126 MHz) of P215

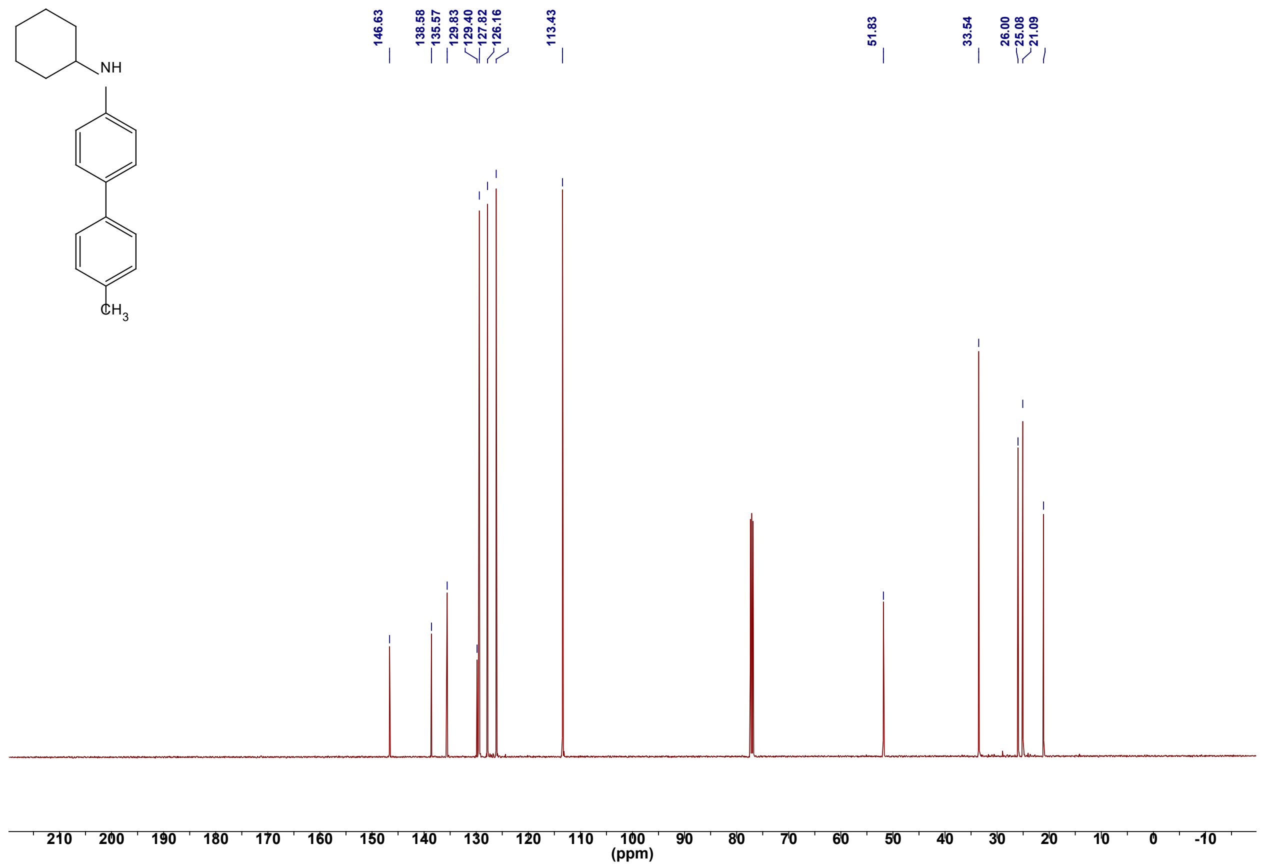

^{1}H NMR ($CDCl_3$, 500 MHz) of P216

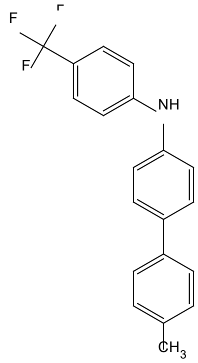

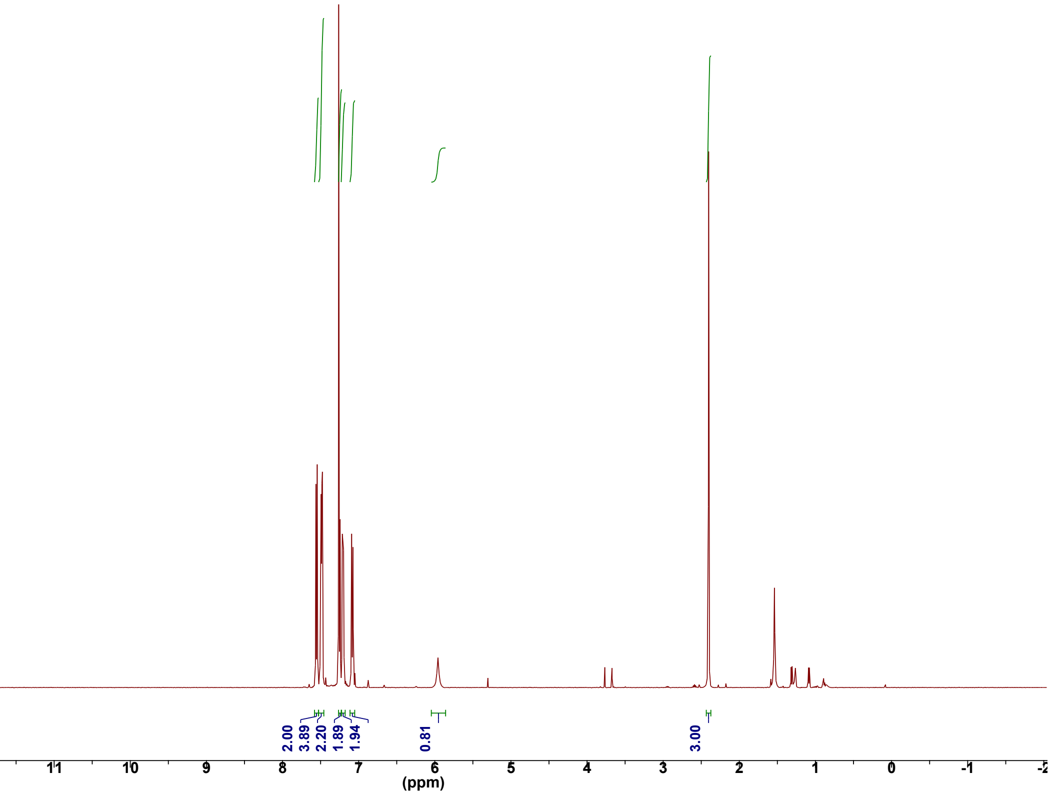

^{13}C NMR ($CDCl_3$, 126 MHz) of P216

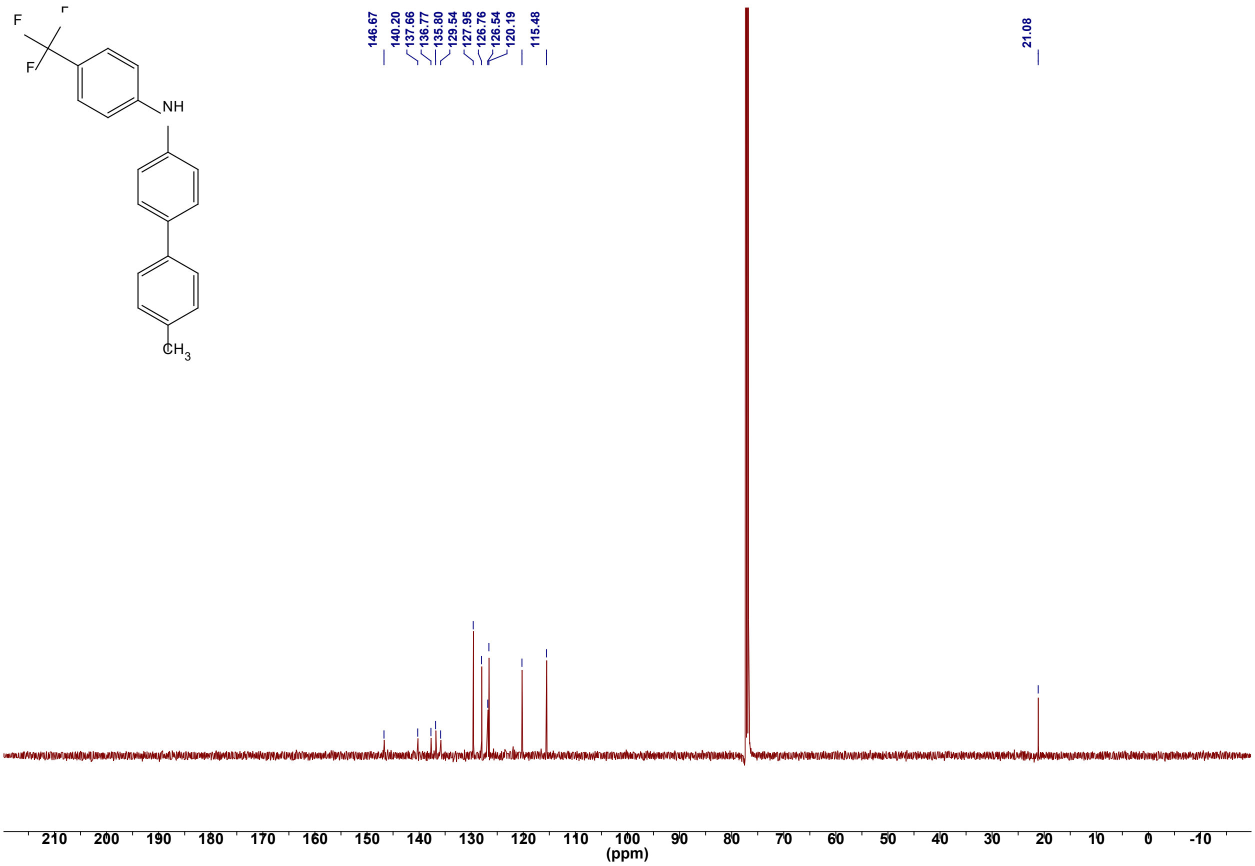

^{19}F NMR ($CDCl_3$, 471 MHz) of P216

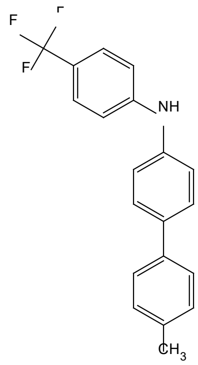

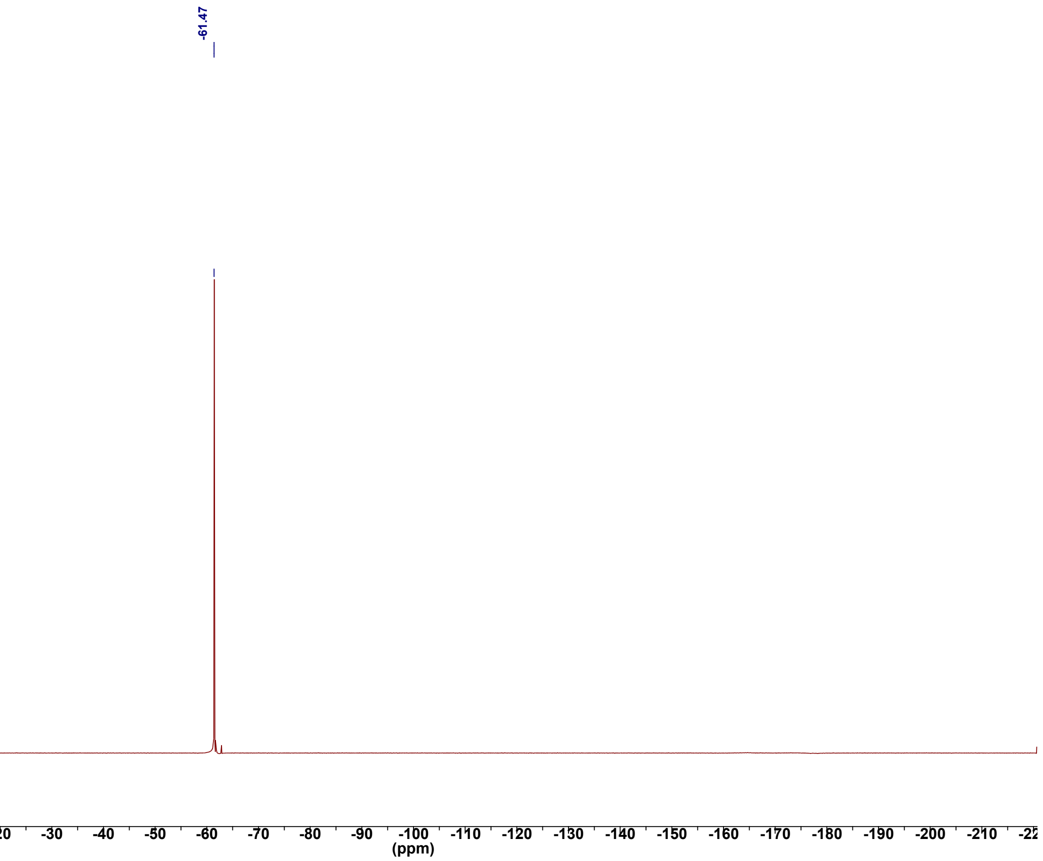

^{1}H NMR ($CDCl_3$, 500 MHz) of P217

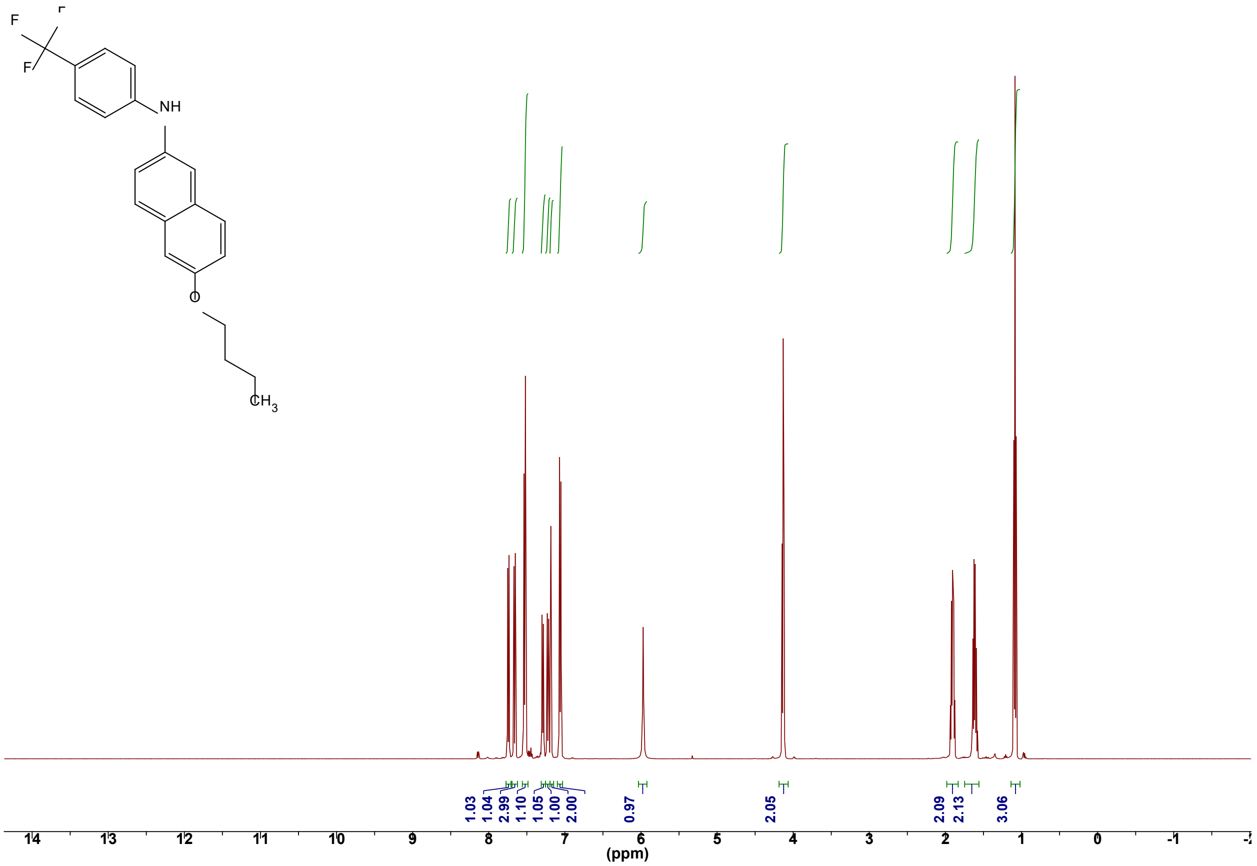

^{13}C NMR ($CDCl_3$, 126 MHz) of P217

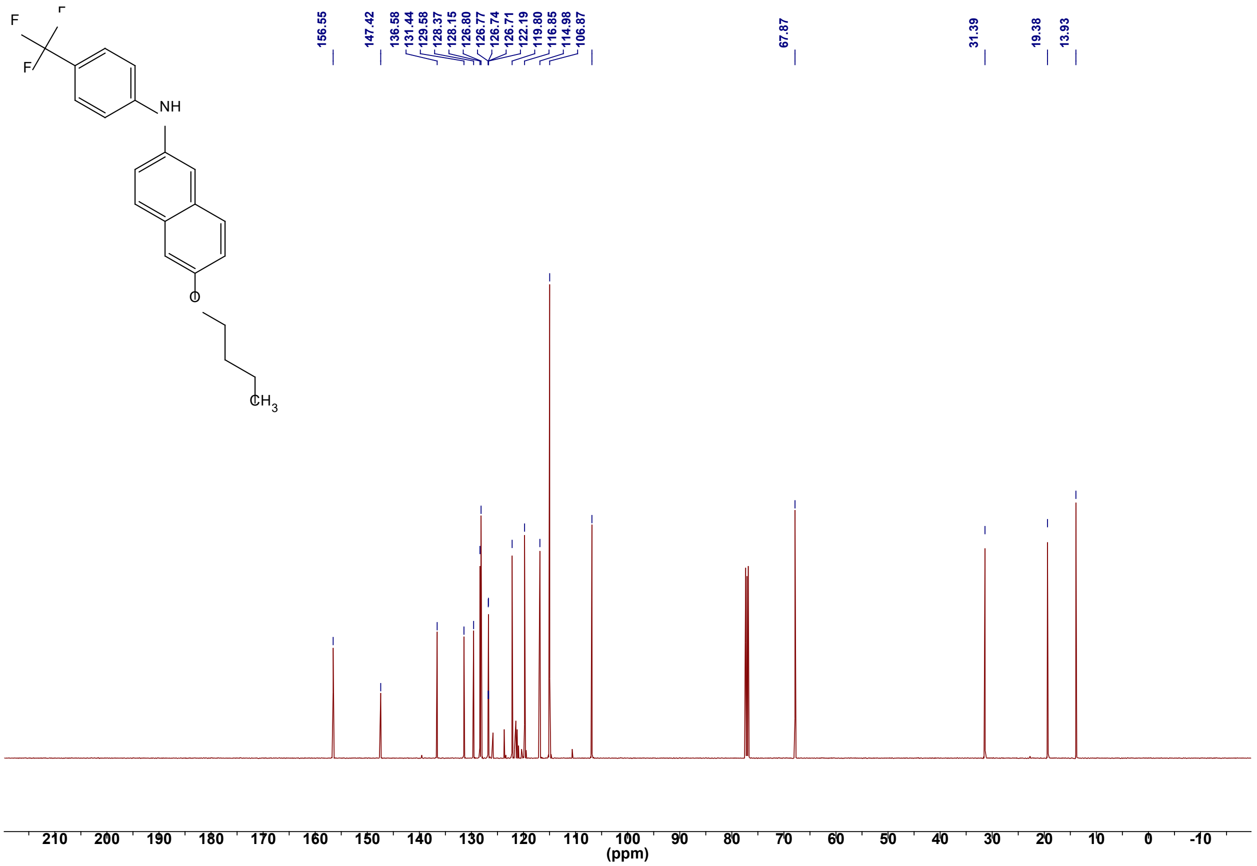

^{19}F NMR ($CDCl_3$, 471 MHz) of P217

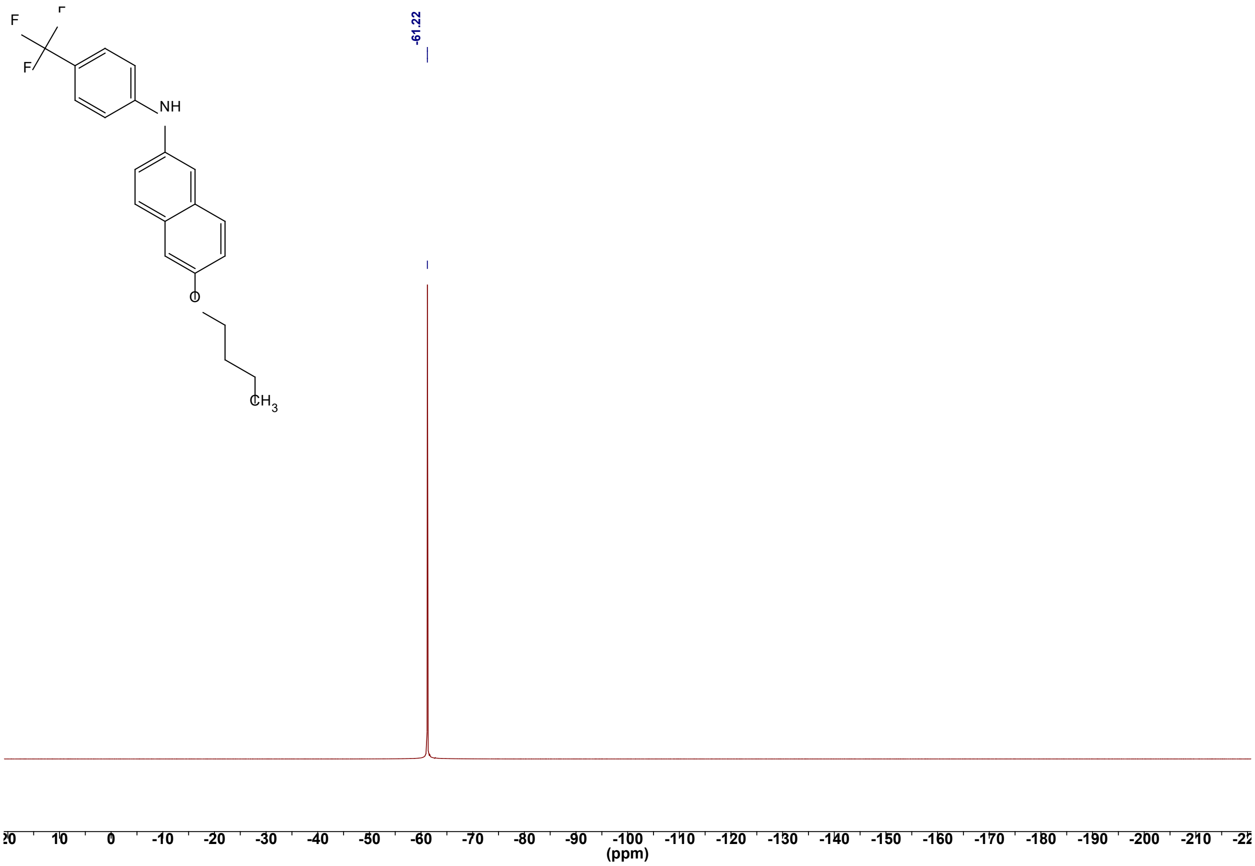

^{1}H NMR ($CDCl_3$, 500 MHz) of P218

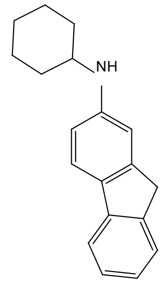

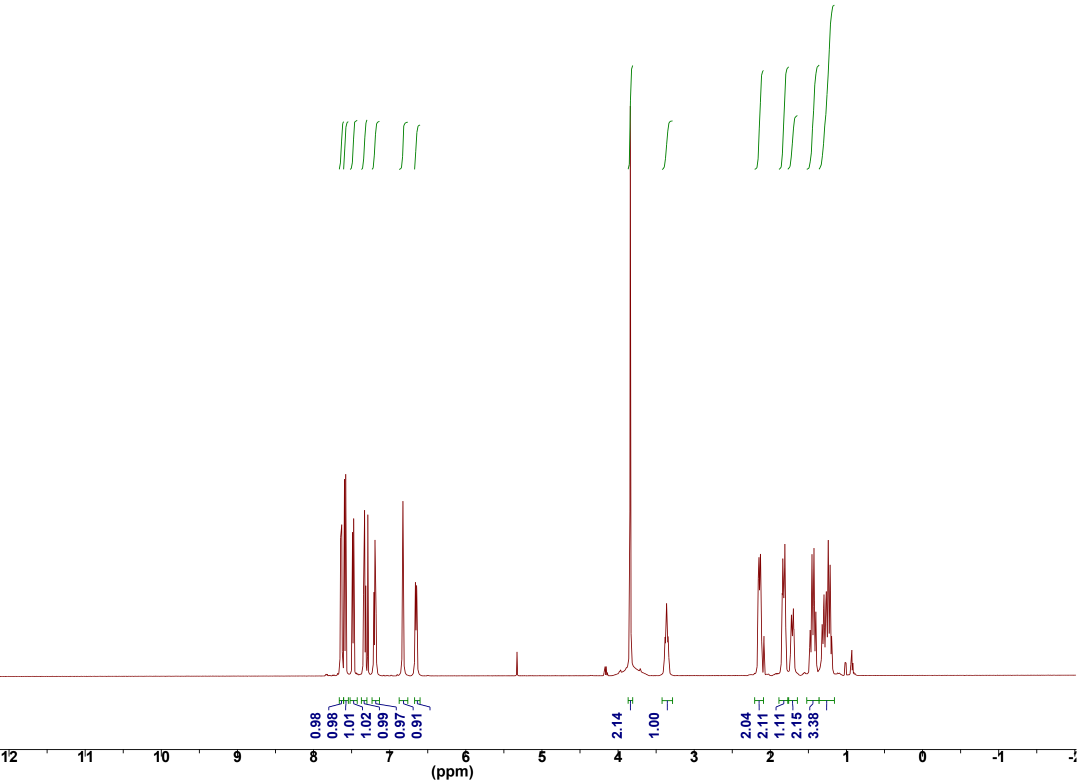

^{13}C NMR ($CDCl_3$, 126 MHz) of P218

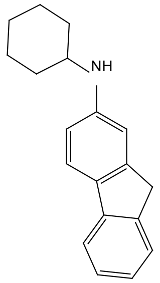

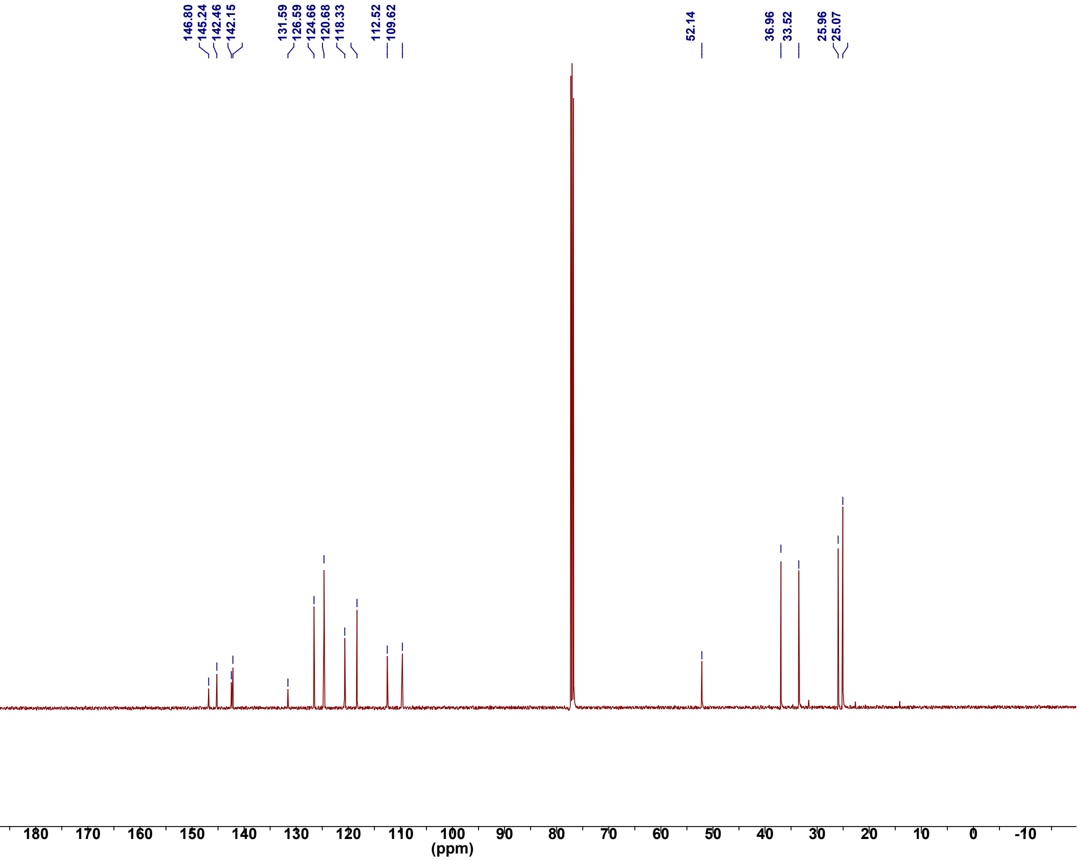

^1H NMR ($CDCl_3$, 500 MHz) of P219

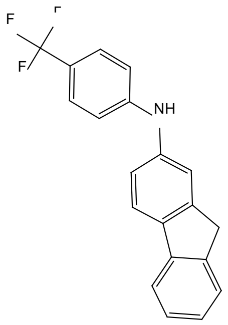

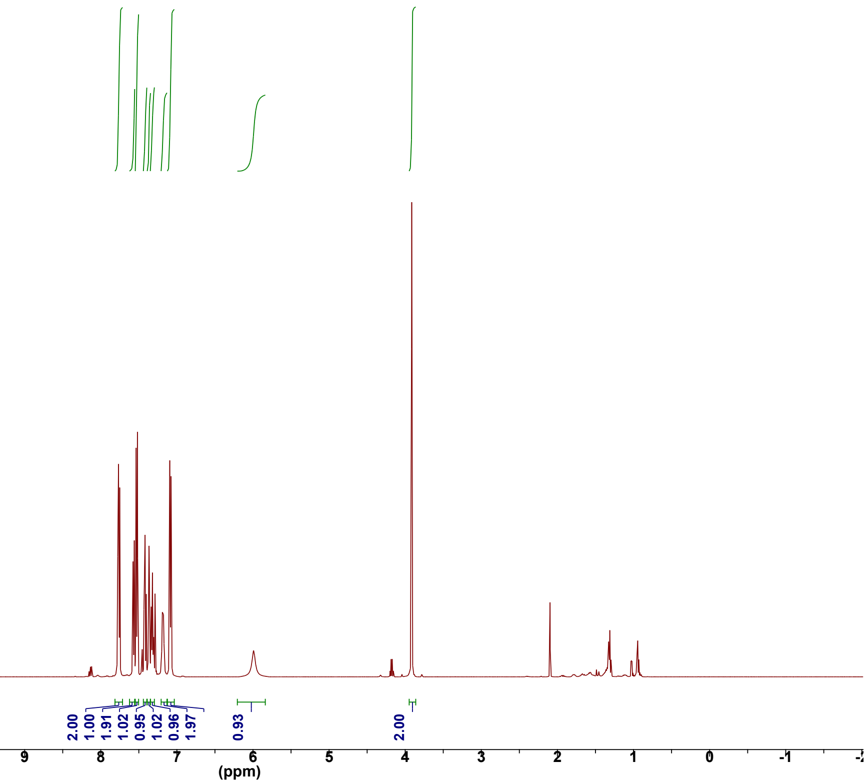

^{13}C NMR ($CDCl_3$, 126 MHz) of P219

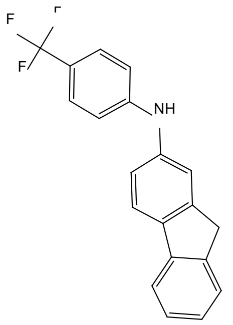

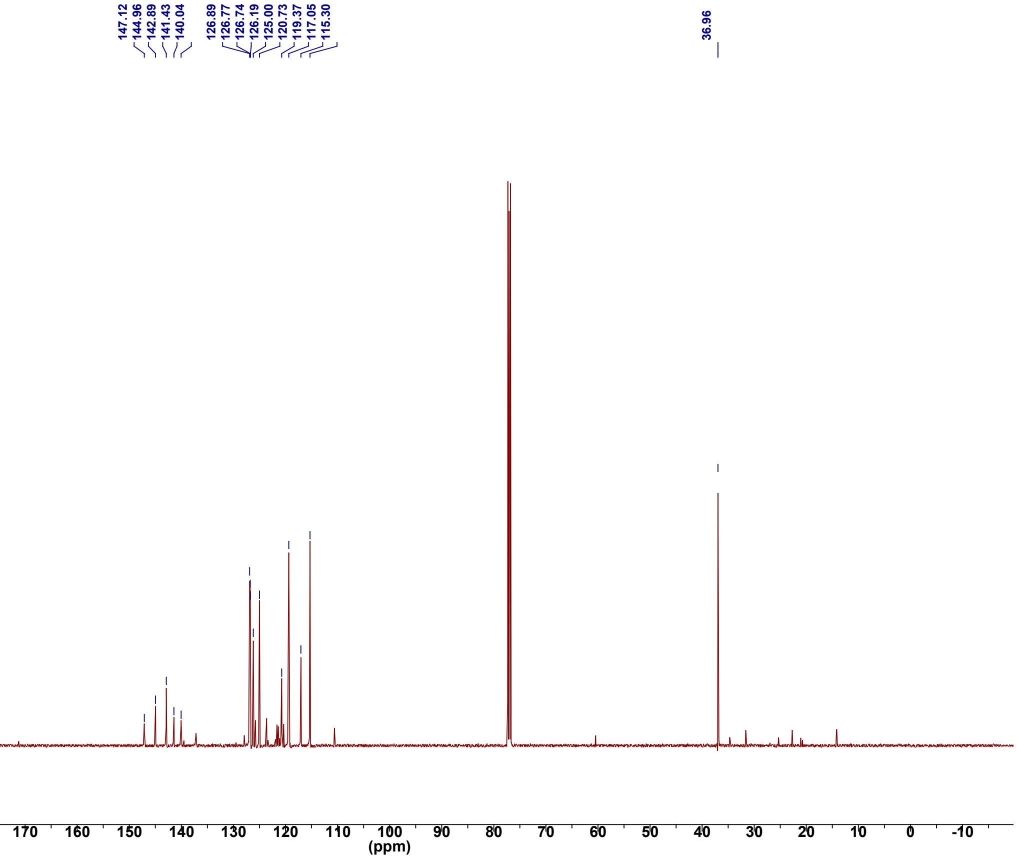

^{19}F NMR ($CDCl_3$, 471 MHz) of P219

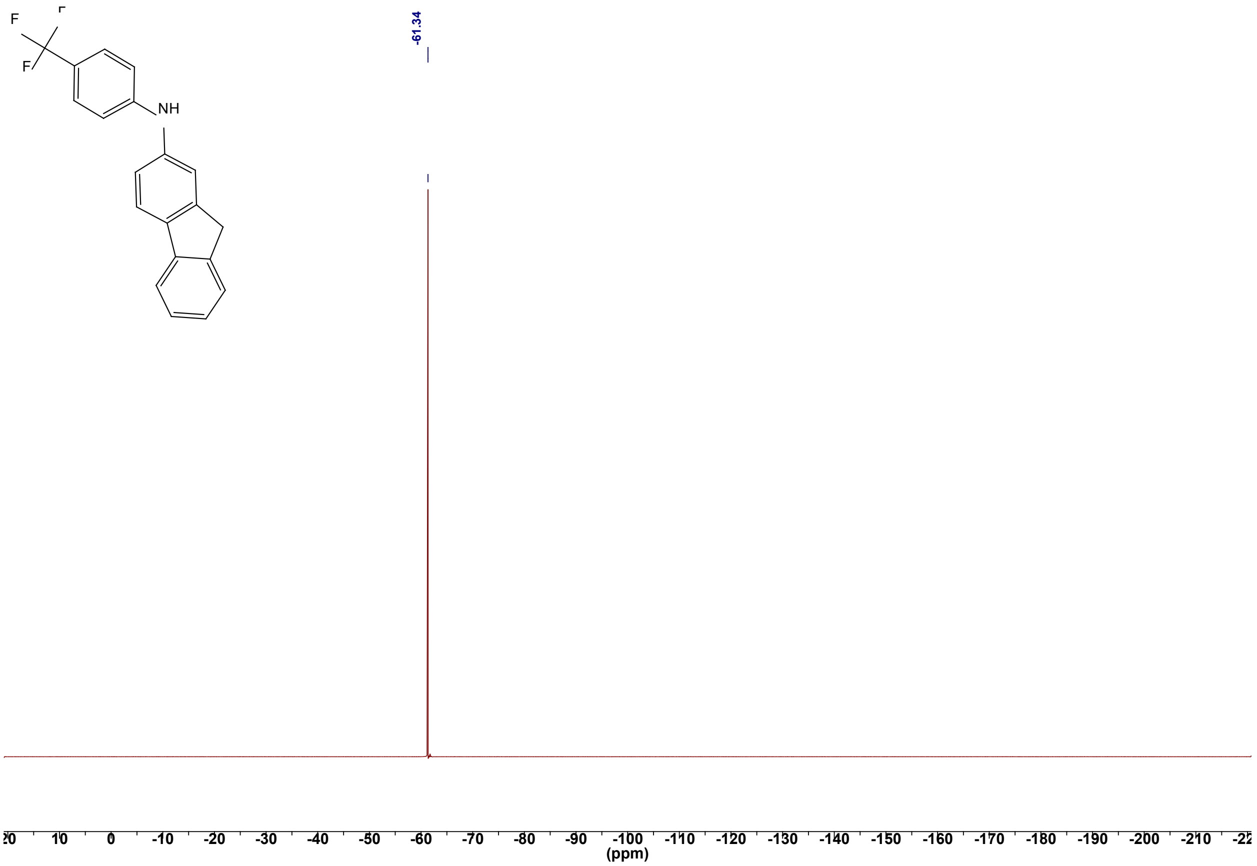

^{1}H NMR ($CDCl_3$, 500 MHz) of P300

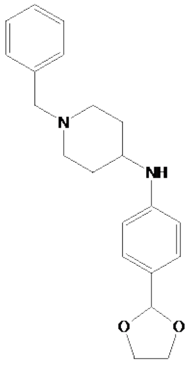

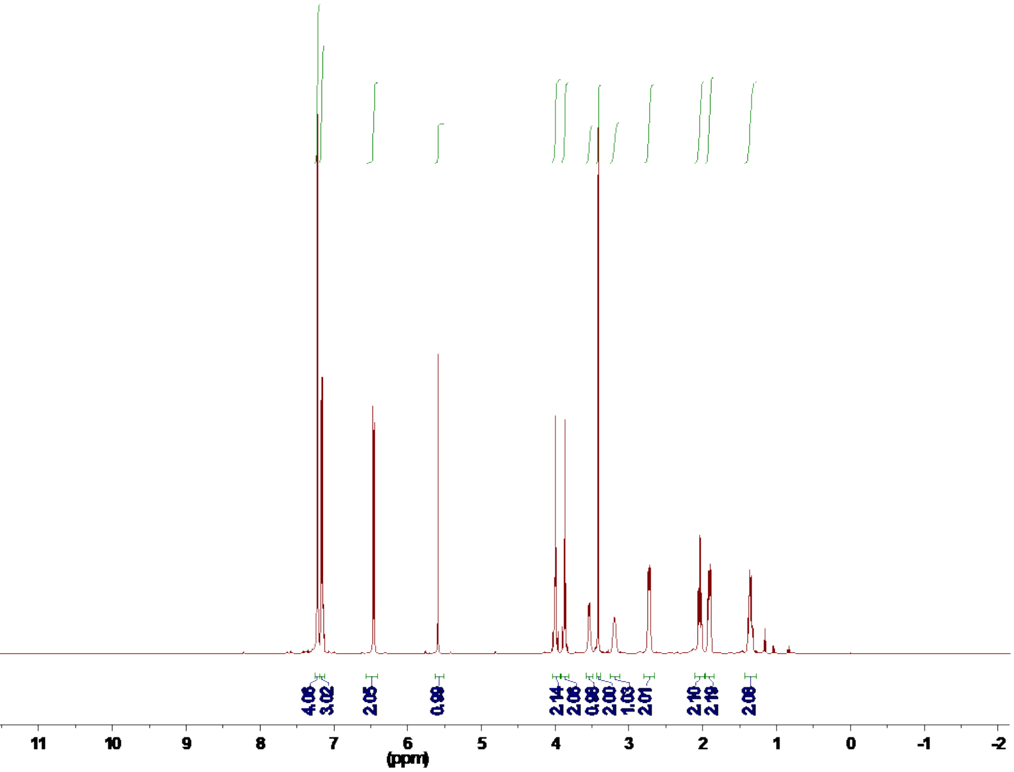

^{13}C NMR ($CDCl_3$, 126 MHz) of P300

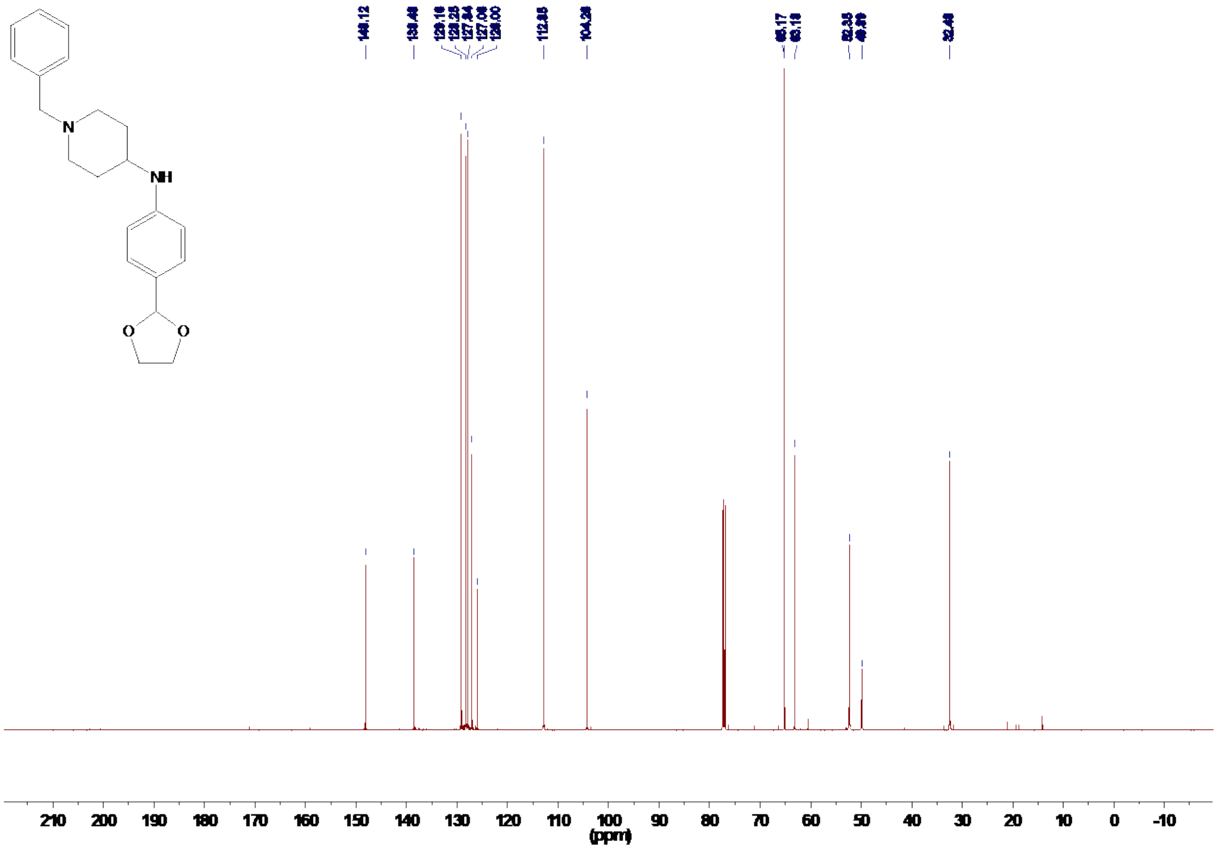

^{1}H NMR ($CDCl_3$, 500 MHz) of P301

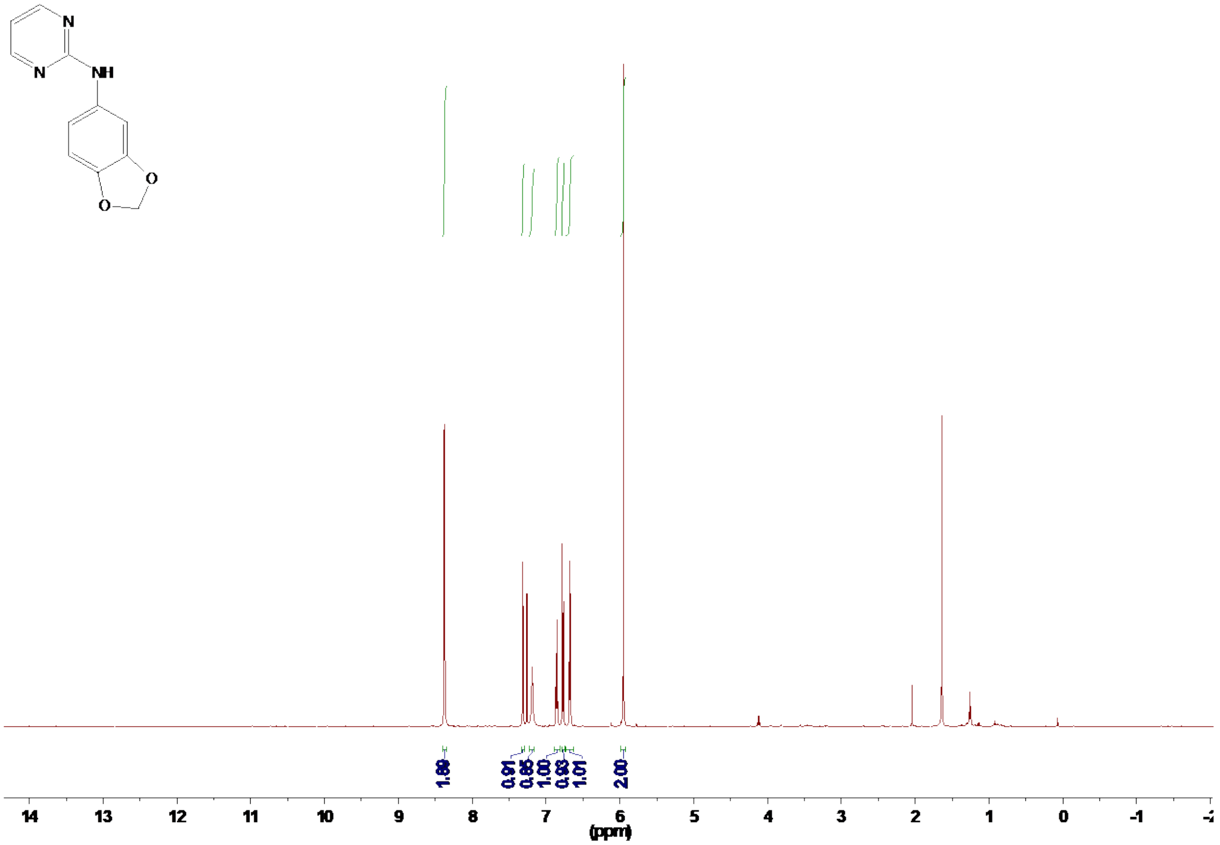

^{13}C NMR ($CDCl_3$, 126 MHz) of P301

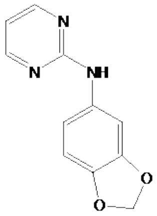

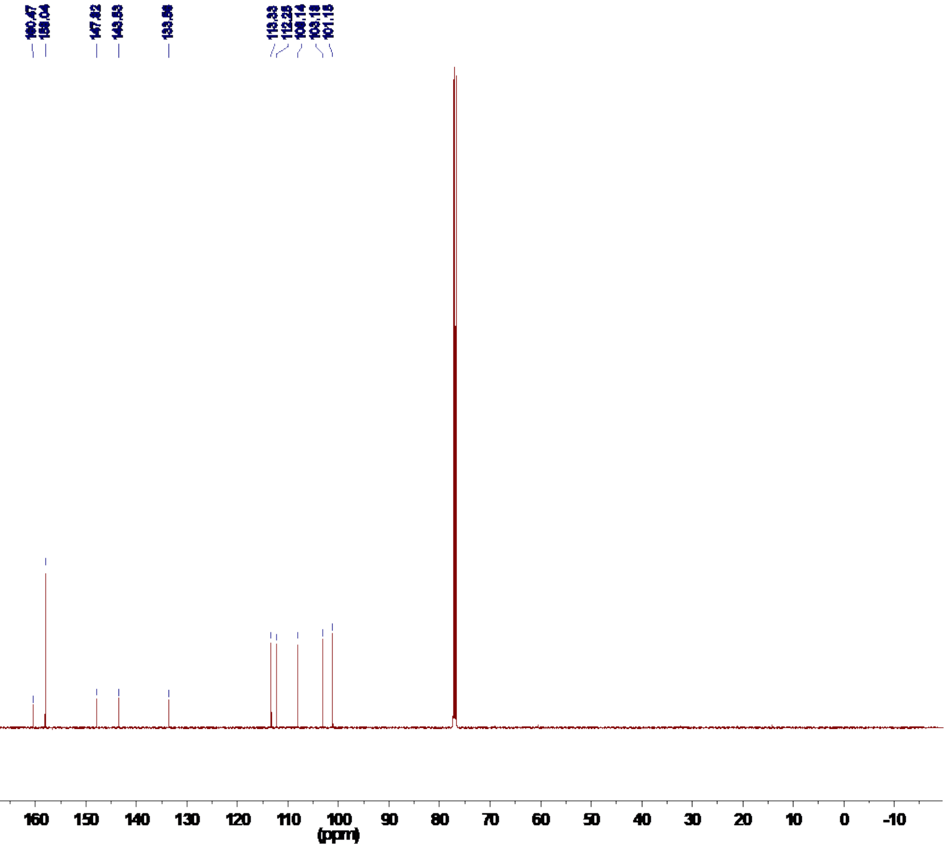

IR Spectra of C–N Coupling Products

FT-IR (Diamond-ATR, neat, cm^{-1}) of P100

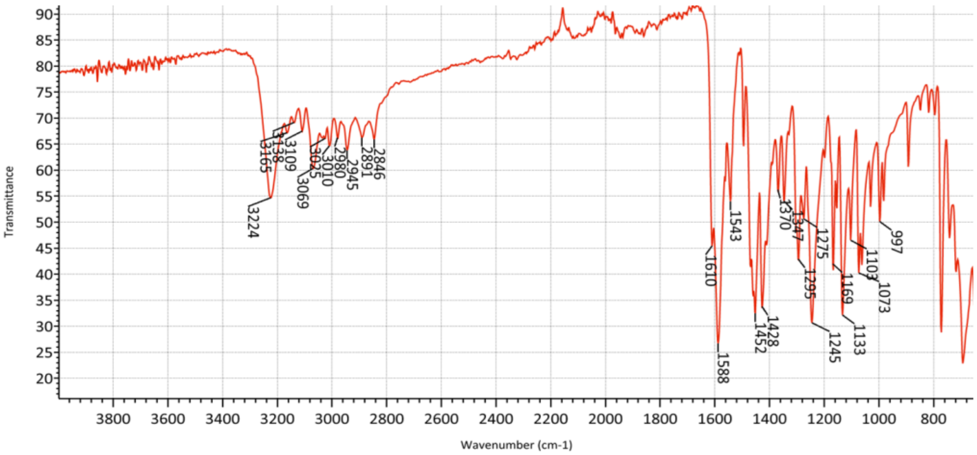

FT-IR (Diamond-ATR, neat, cm^{-1}) of P101

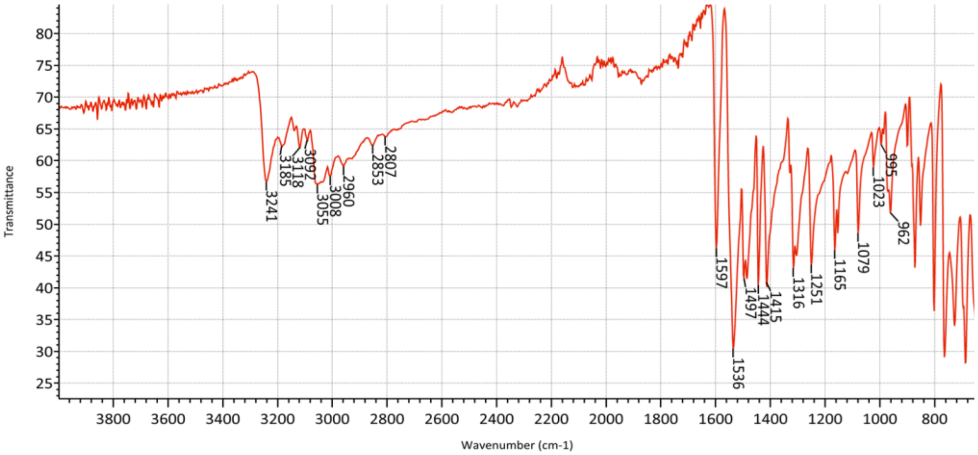

FT-IR (Diamond-ATR, neat, cm^{-1}) of P102

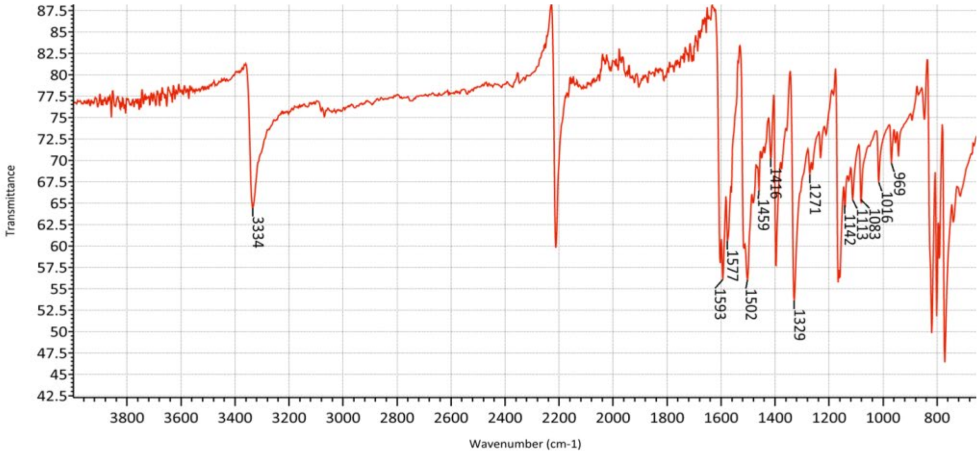

FT-IR (Diamond-ATR, neat, cm^{-1}) of P103

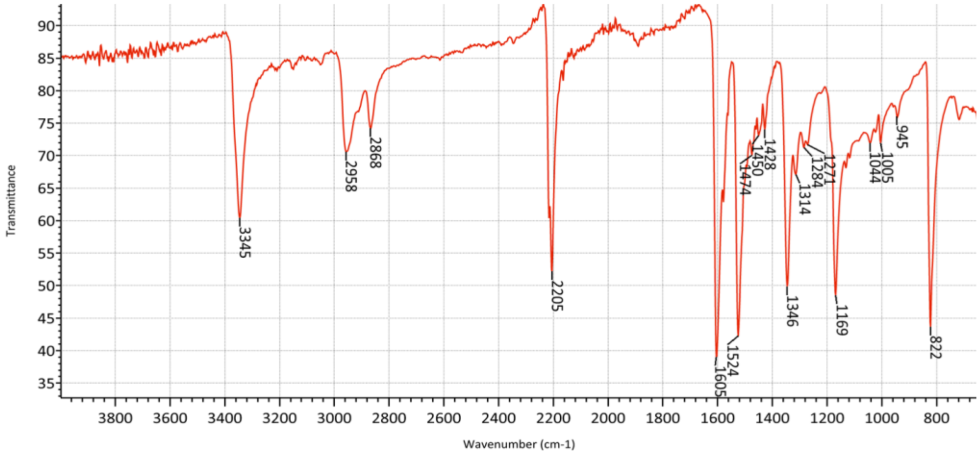

FT-IR (Diamond-ATR, neat, cm^{-1}) of P104

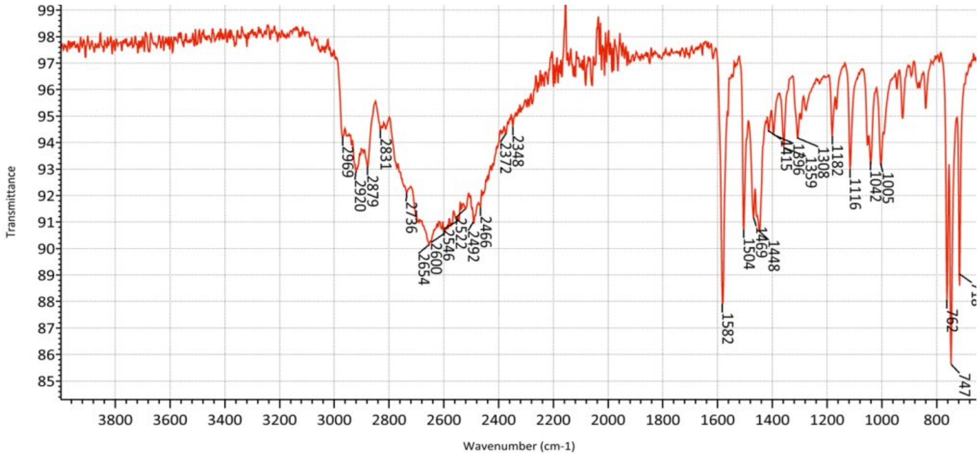

FT-IR (Diamond-ATR, neat, cm^{-1}) of P105

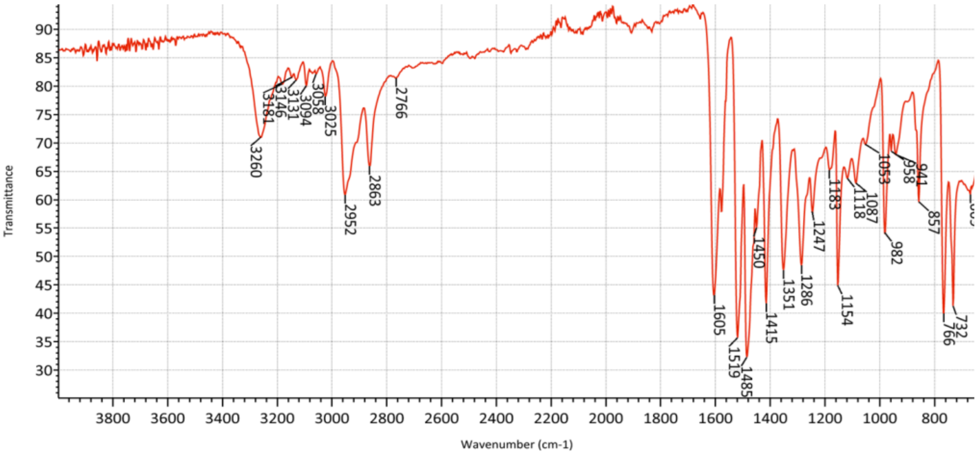

FT-IR (Diamond-ATR, neat, cm^{-1}) of P106

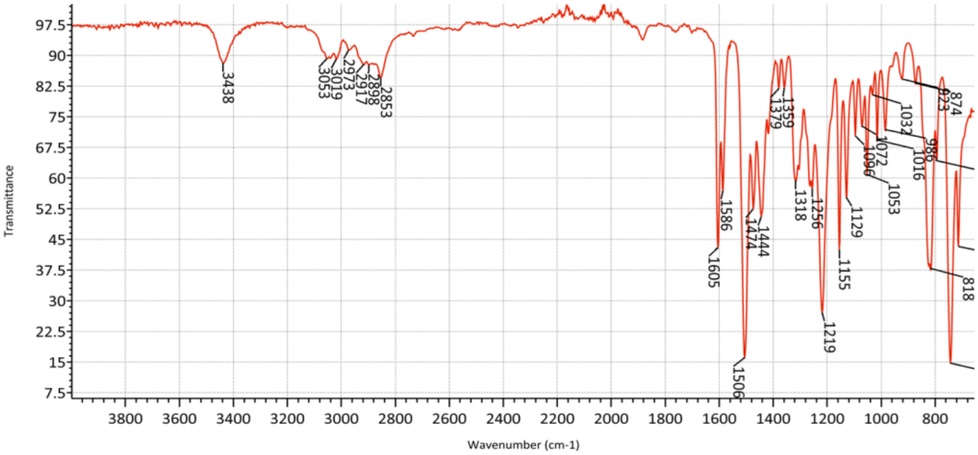

FT-IR (Diamond-ATR, neat, cm^{-1}) of P107

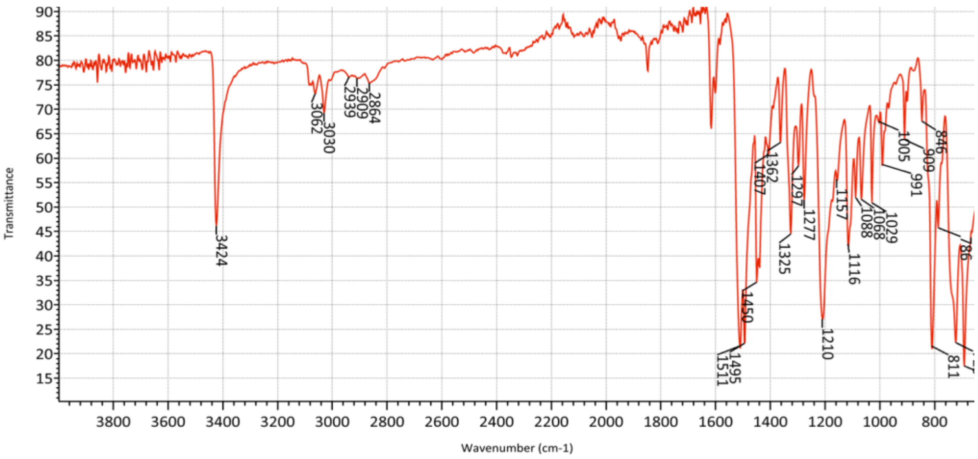

FT-IR (Diamond-ATR, neat, cm^{-1}) of P108

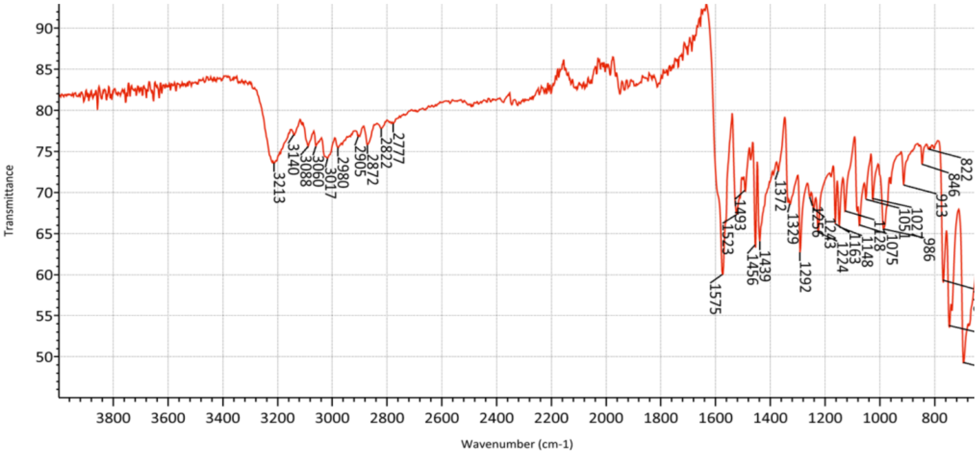

FT-IR (Diamond-ATR, neat, cm^{-1}) of P109

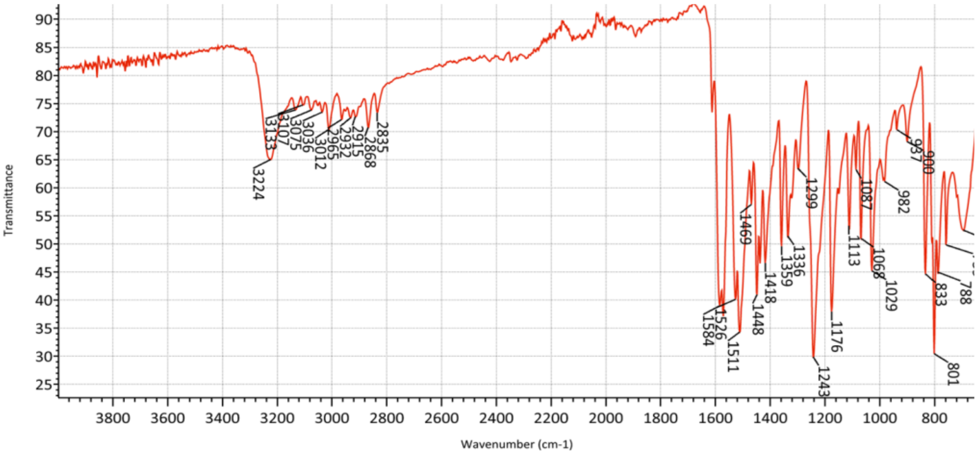

FT-IR (Diamond-ATR, neat, cm^{-1}) of P111

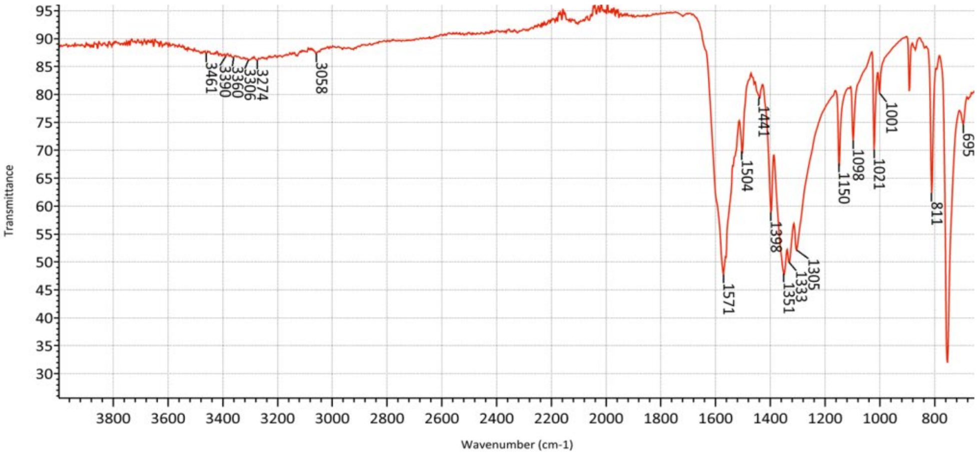

FT-IR (Diamond-ATR, neat, cm^{-1}) of P112

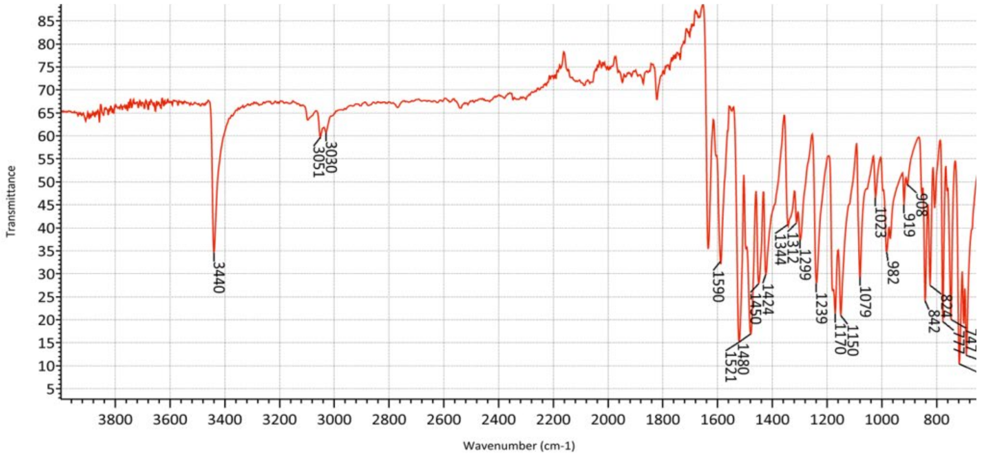

FT-IR (Diamond-ATR, neat, cm^{-1}) of P113

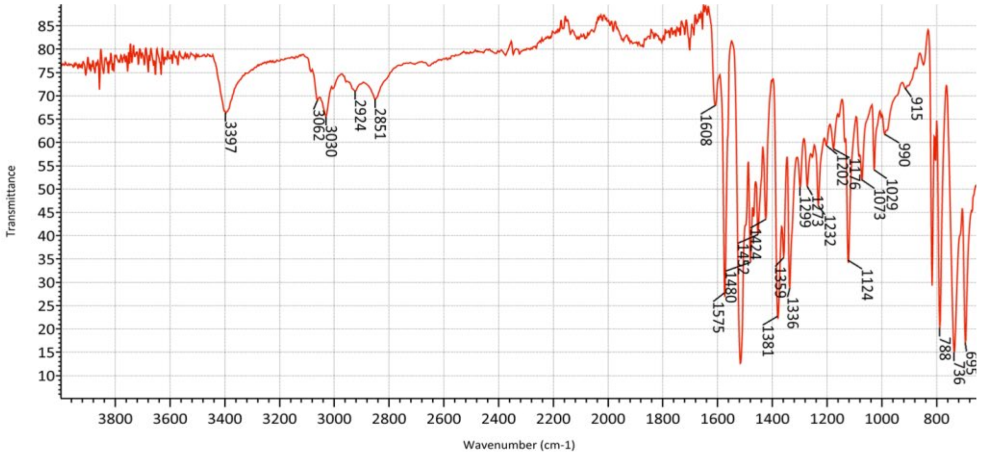

FT-IR (Diamond-ATR, neat, cm^{-1}) of P114

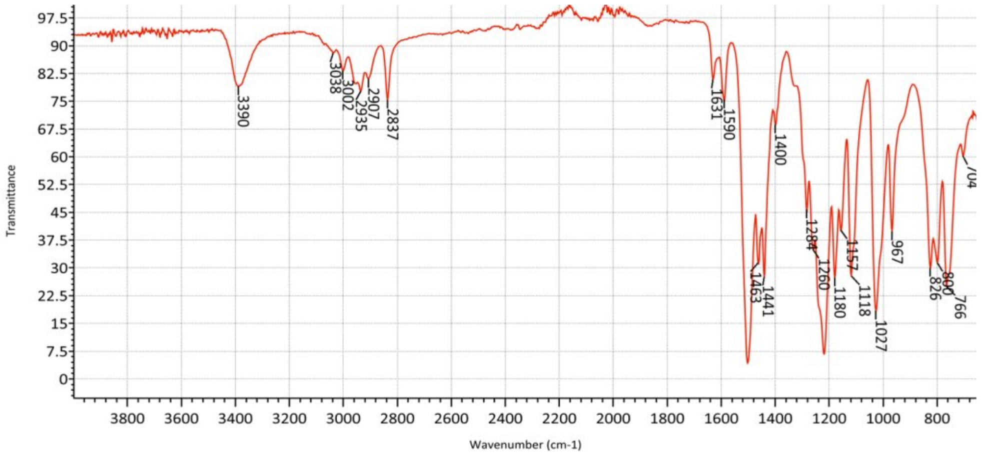

FT-IR (Diamond-ATR, neat, cm^{-1}) of P115

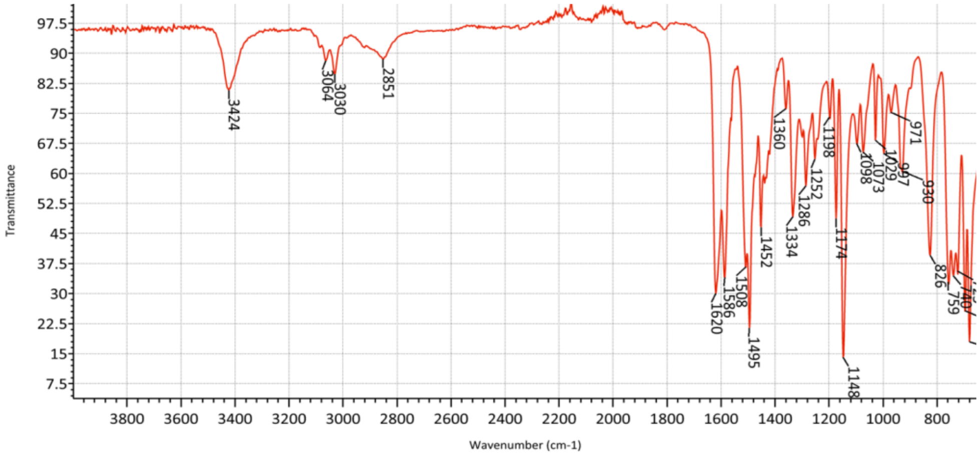

FT-IR (Diamond-ATR, neat, cm^{-1}) of P116

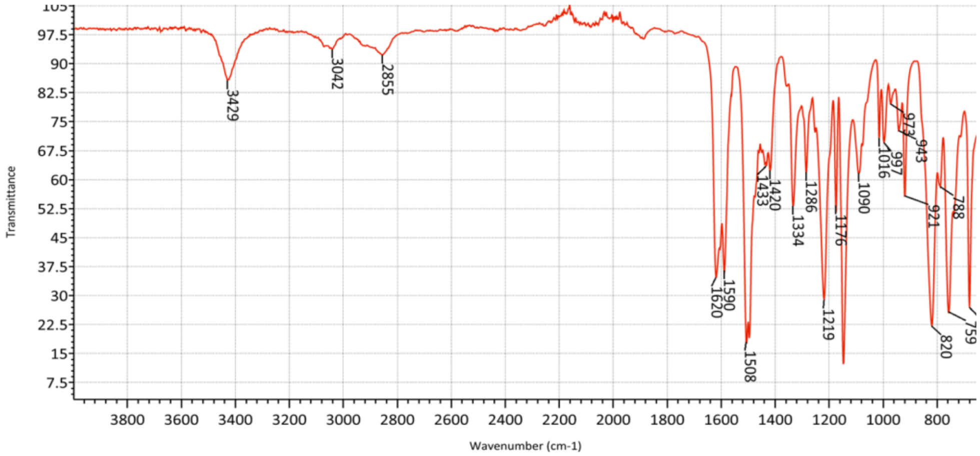

FT-IR (Diamond-ATR, neat, cm^{-1}) of P117

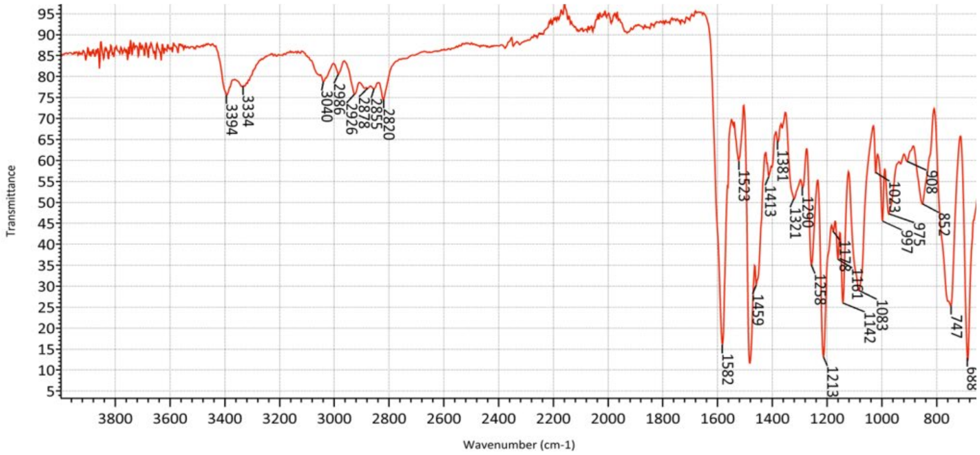

FT-IR (Diamond-ATR, neat, cm^{-1}) of P118

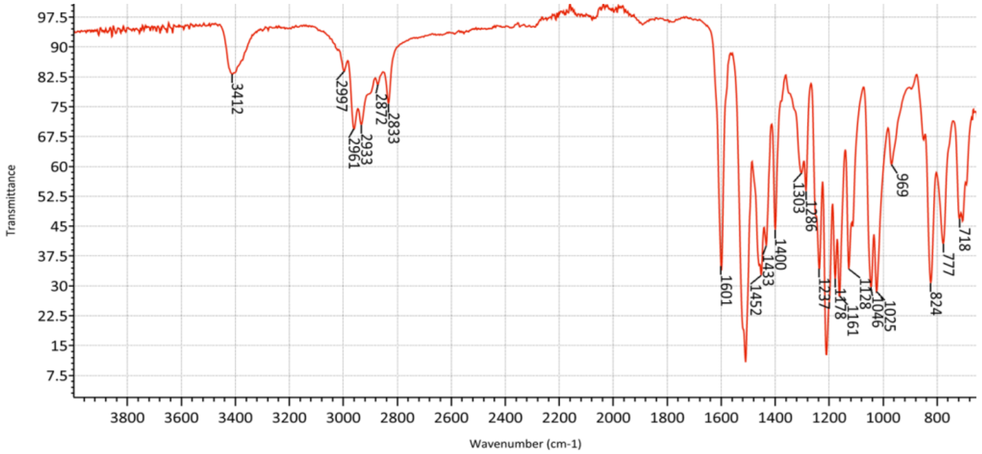

FT-IR (Diamond-ATR, neat, cm^{-1}) of P119

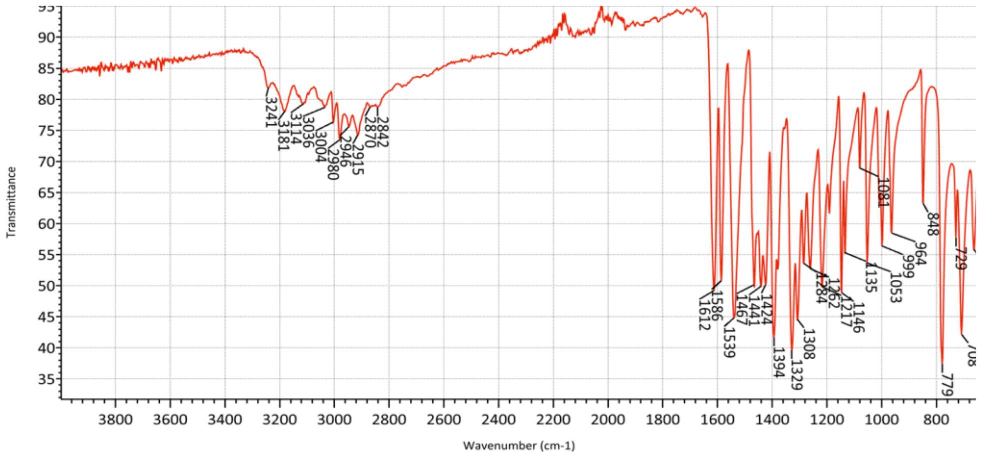

FT-IR (Diamond-ATR, neat, cm^{-1}) of P120

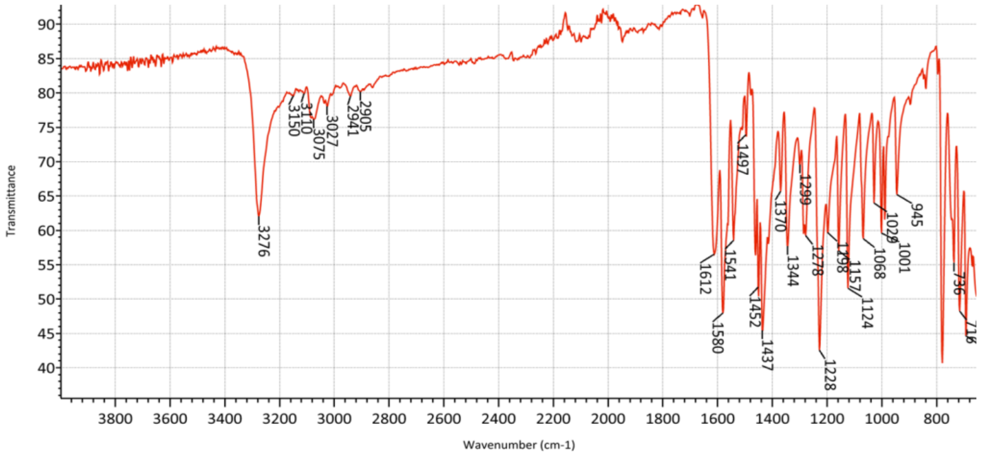

FT-IR (Diamond-ATR, neat, cm^{-1}) of P121

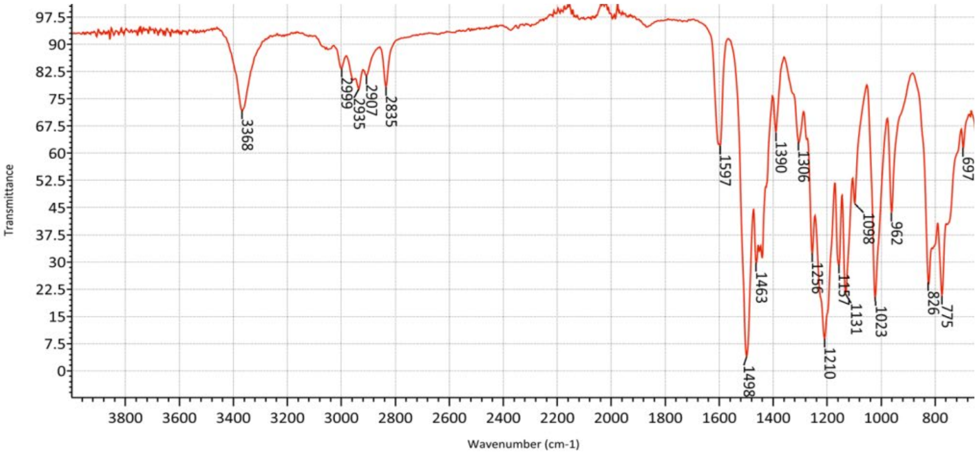

FT-IR (Diamond-ATR, neat, cm^{-1}) of P122

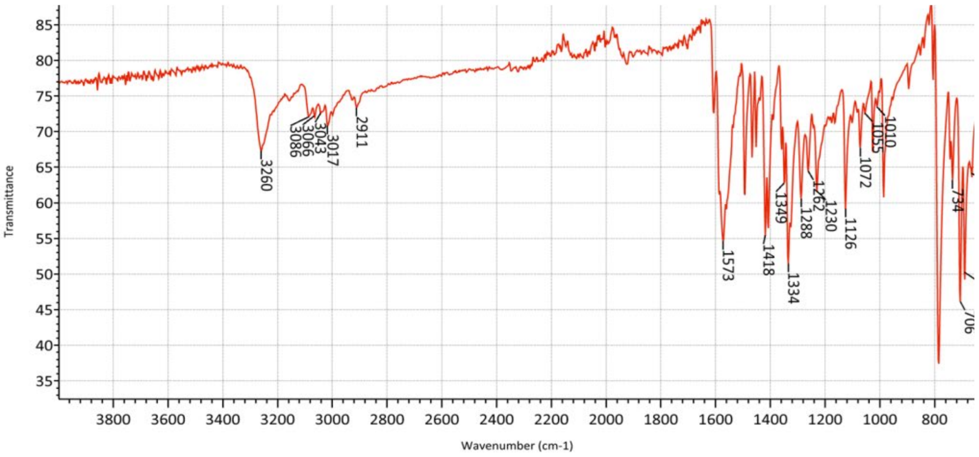

FT-IR (Diamond-ATR, neat, cm^{-1}) of P123

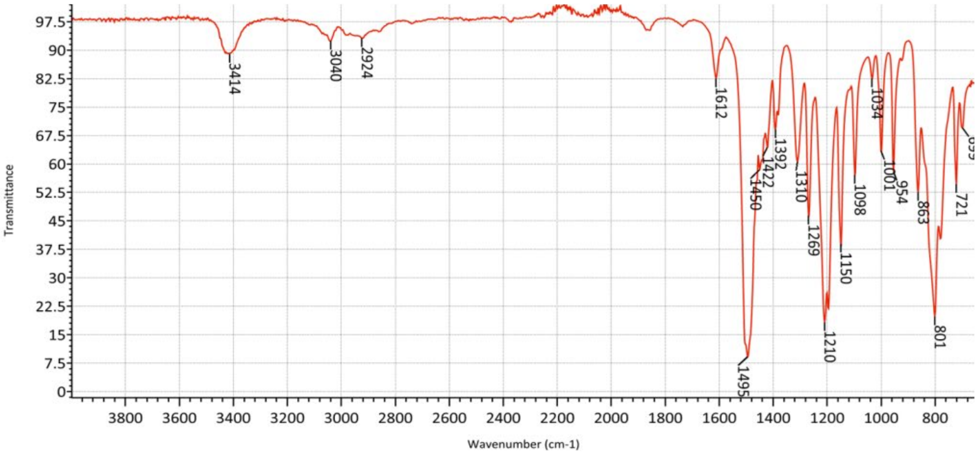

FT-IR (Diamond-ATR, neat, cm^{-1}) of P124

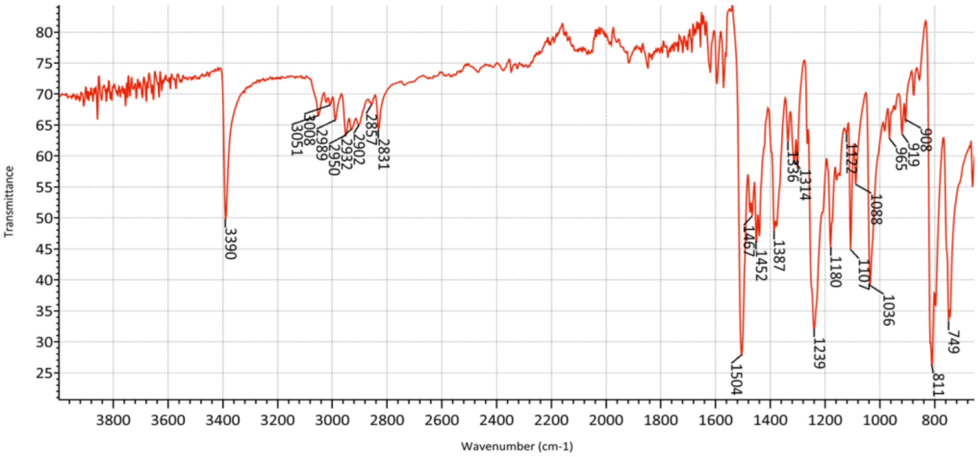

FT-IR (Diamond-ATR, neat, cm^{-1}) of P125

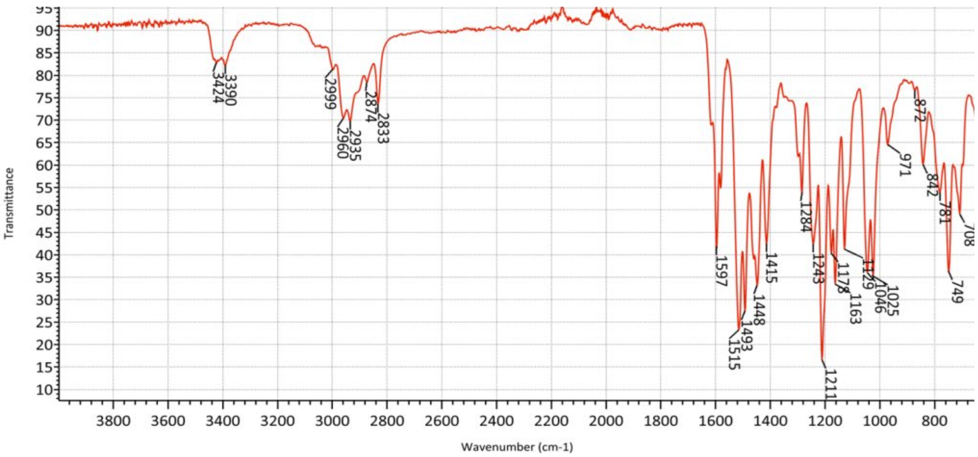

FT-IR (Diamond-ATR, neat, cm^{-1}) of P126

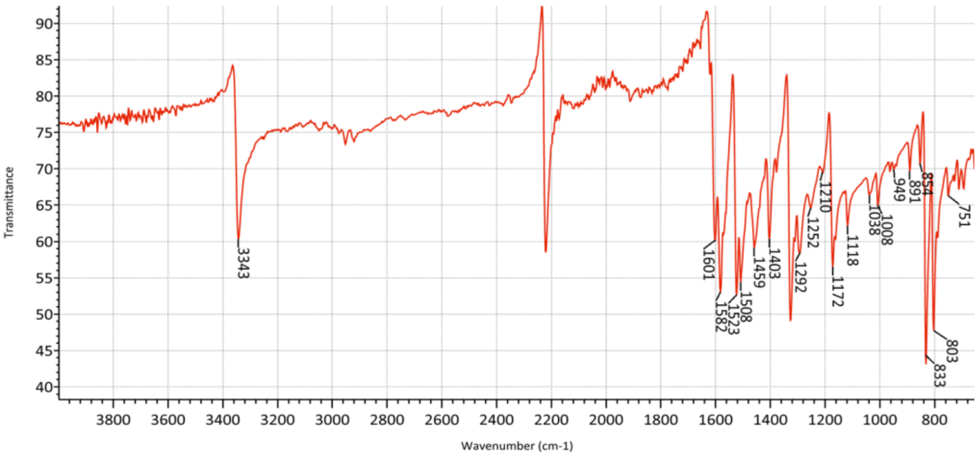

FT-IR (Diamond-ATR, neat, cm^{-1}) of P127

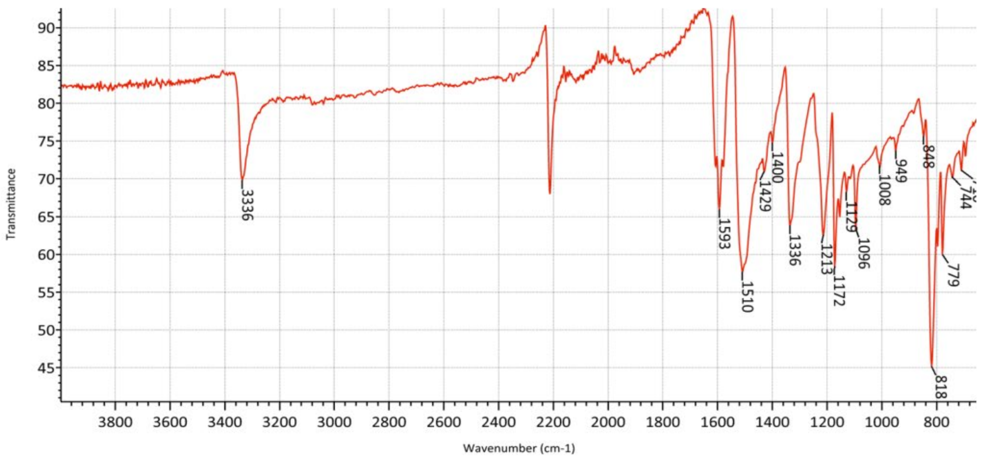

FT-IR (Diamond-ATR, neat, cm^{-1}) of P200

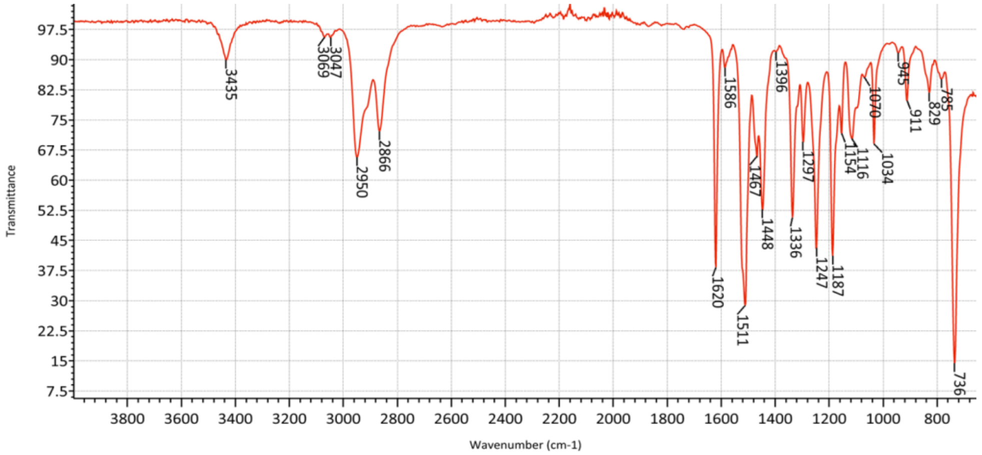

FT-IR (Diamond-ATR, neat, cm^{-1}) of P201

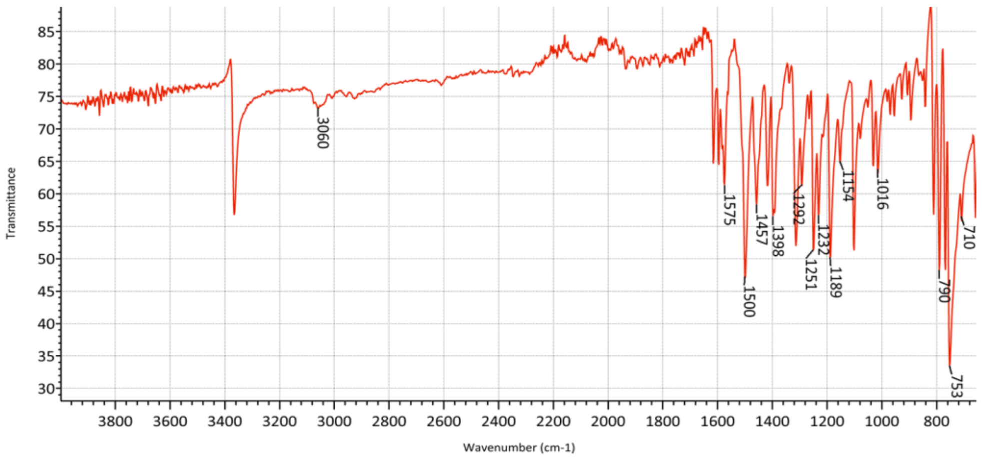

FT-IR (Diamond-ATR, neat, cm^{-1}) of P202

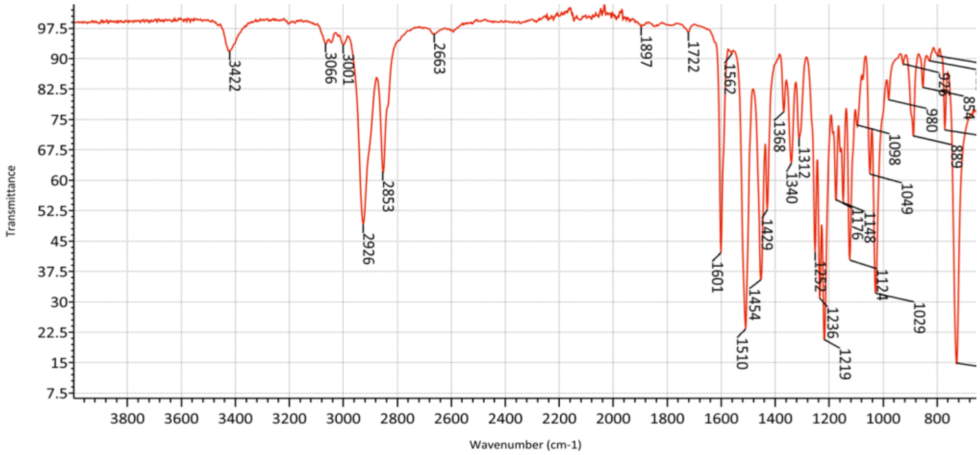

FT-IR (Diamond-ATR, neat, cm^{-1}) of P203

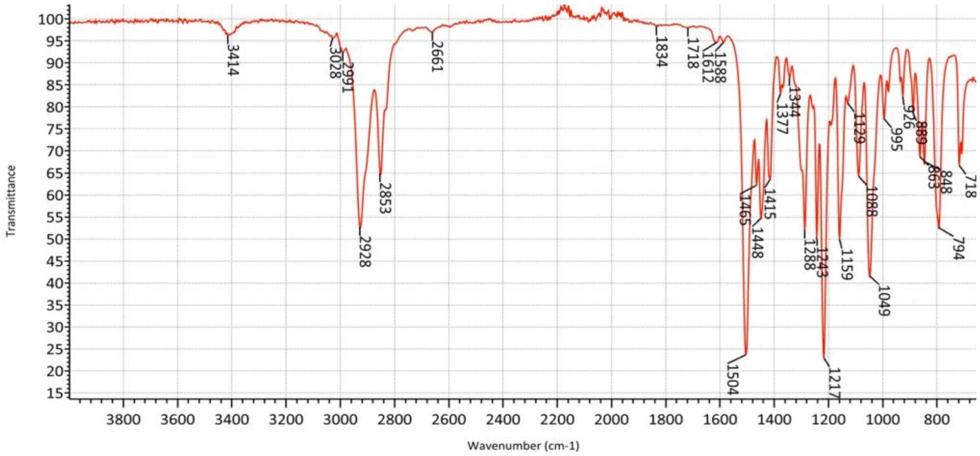

FT-IR (Diamond-ATR, neat, cm^{-1}) of P204

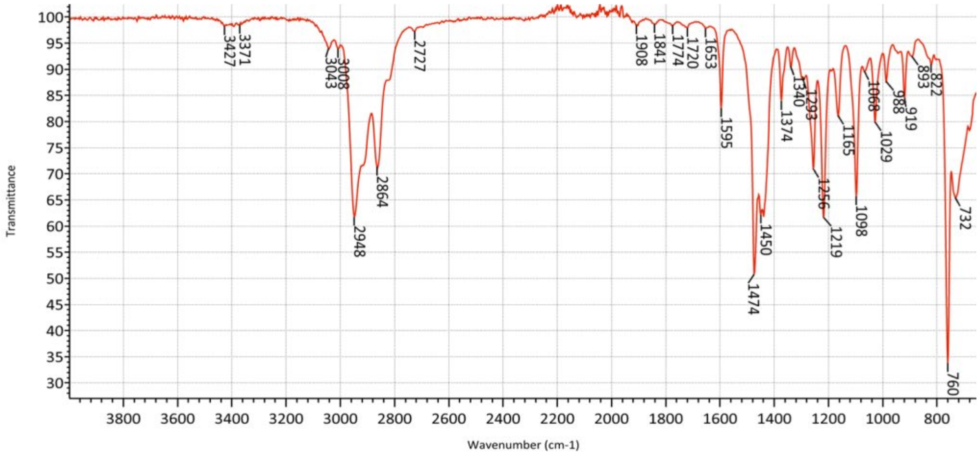

FT-IR (Diamond-ATR, neat, cm^{-1}) of P205

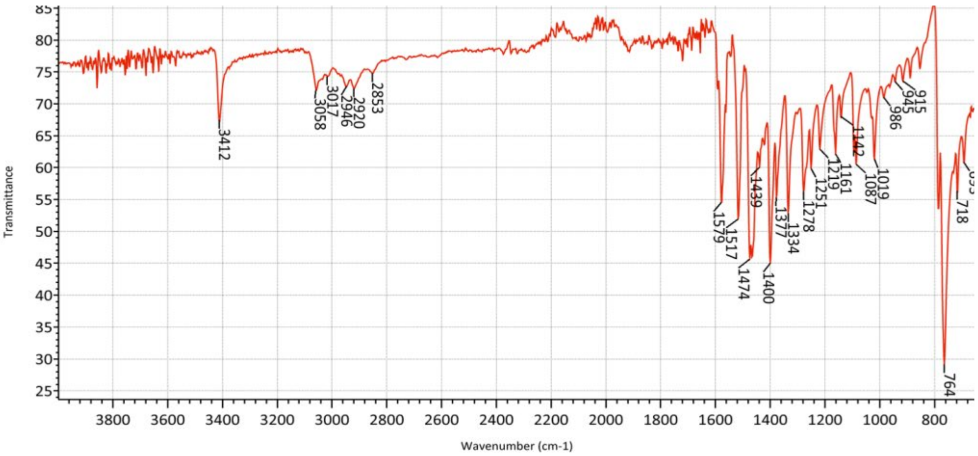

FT-IR (Diamond-ATR, neat, cm^{-1}) of P206

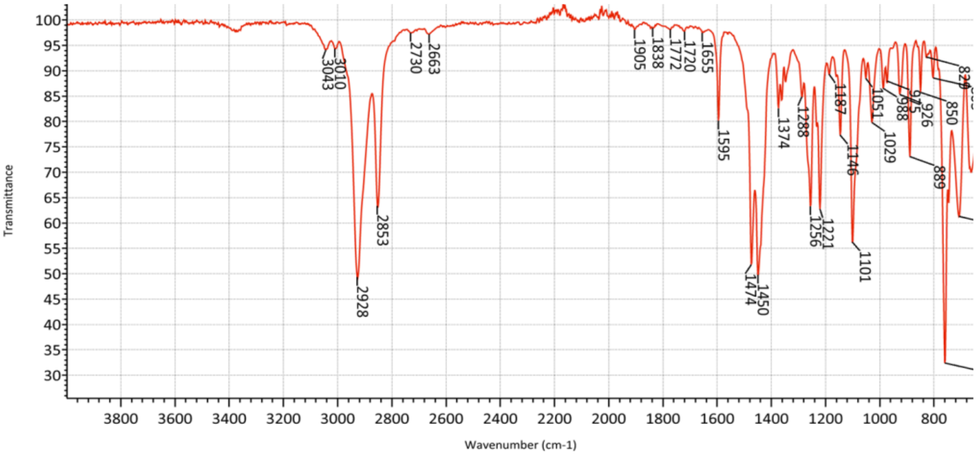

FT-IR (Diamond-ATR, neat, cm^{-1}) of P207

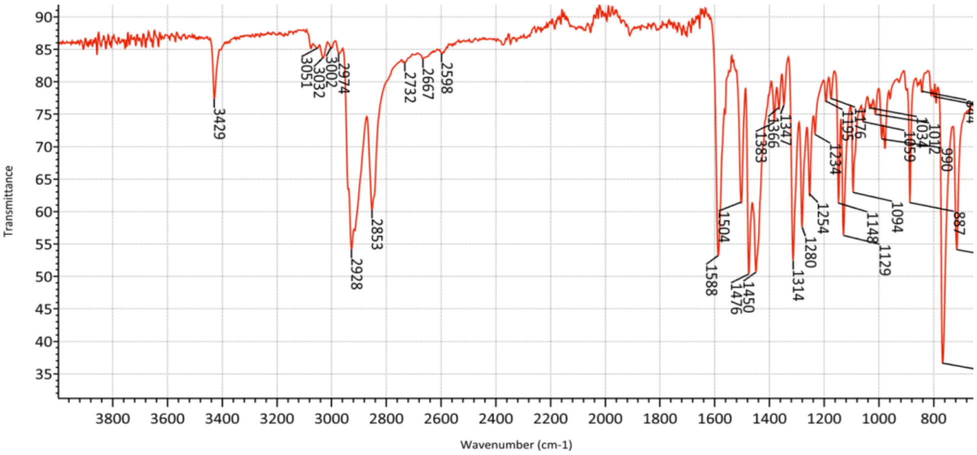

FT-IR (Diamond-ATR, neat, cm^{-1}) of P208

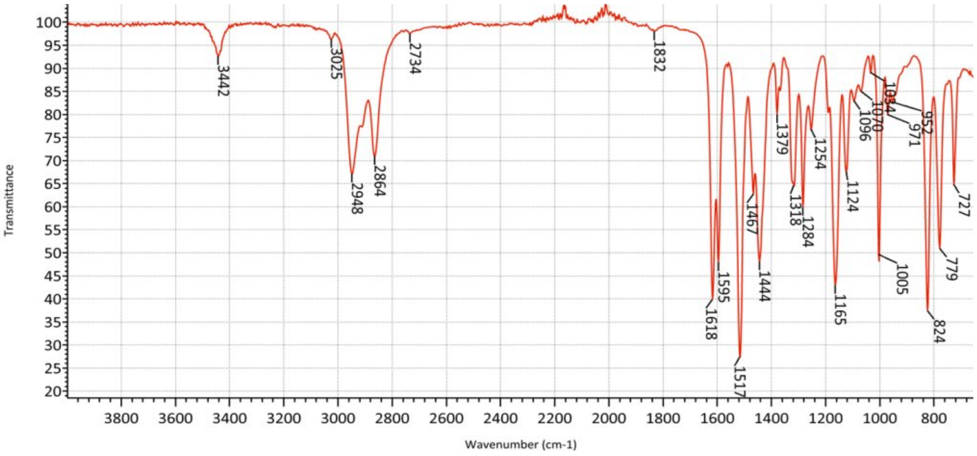

FT-IR (Diamond-ATR, neat, cm^{-1}) of P209

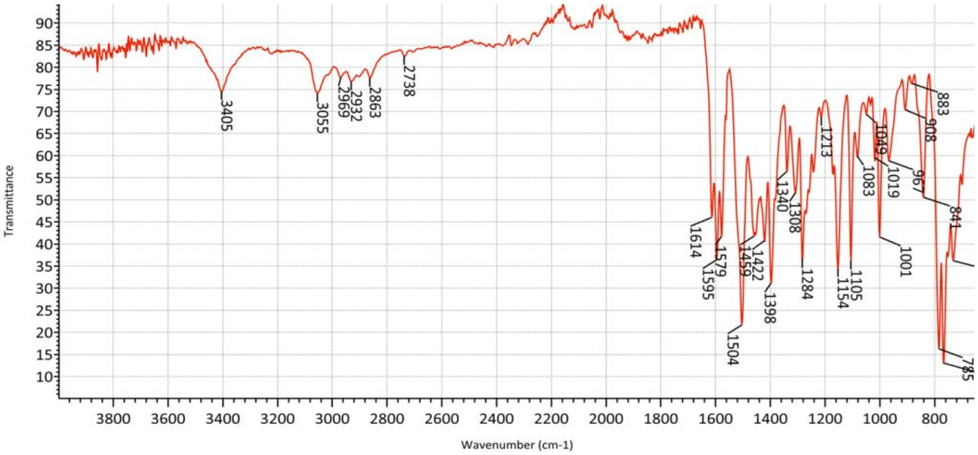

FT-IR (Diamond-ATR, neat, cm^{-1}) of P210

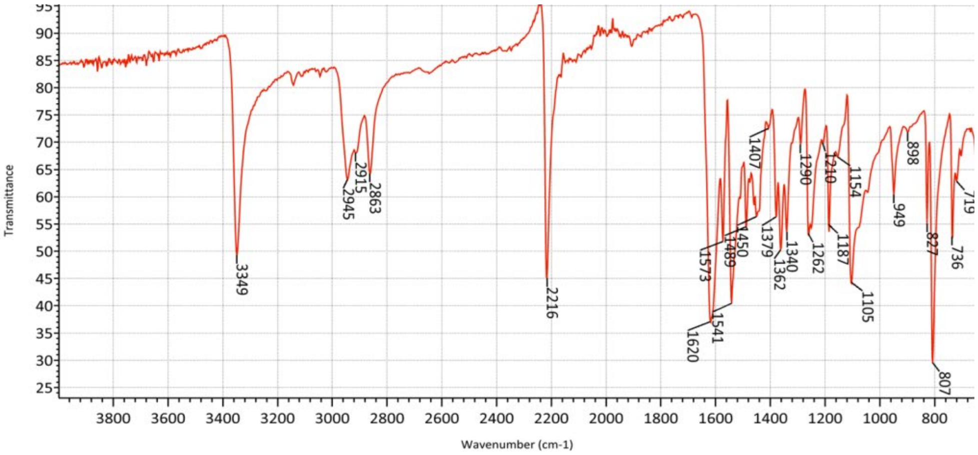

FT-IR (Diamond-ATR, neat, cm^{-1}) of P211

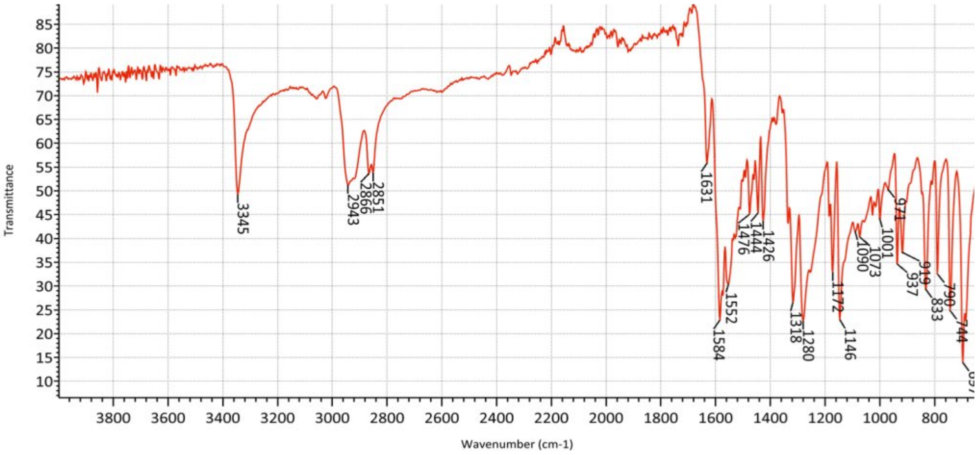

FT-IR (Diamond-ATR, neat, cm^{-1}) of P212

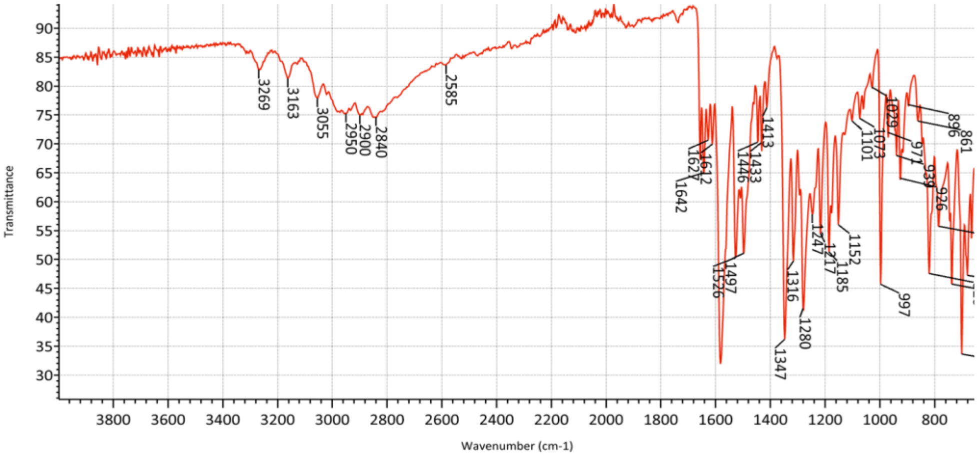

FT-IR (Diamond-ATR, neat, cm^{-1}) of P213

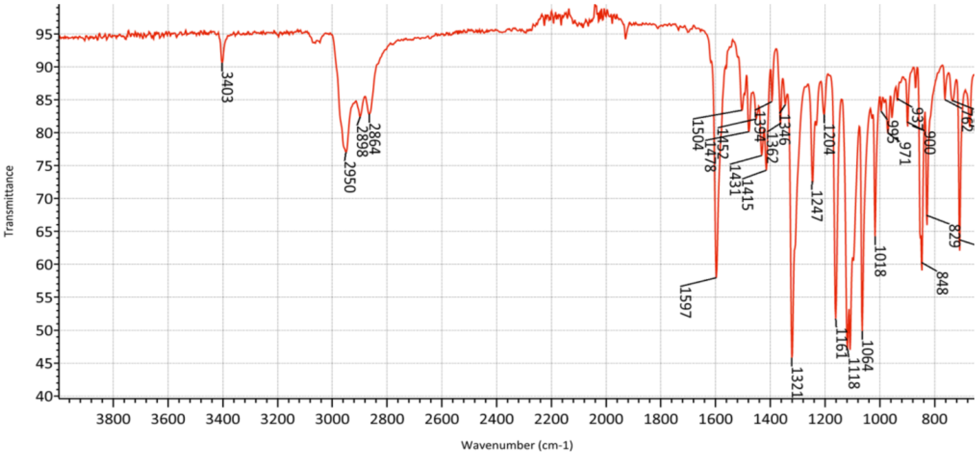

FT-IR (Diamond-ATR, neat, cm^{-1}) of P214

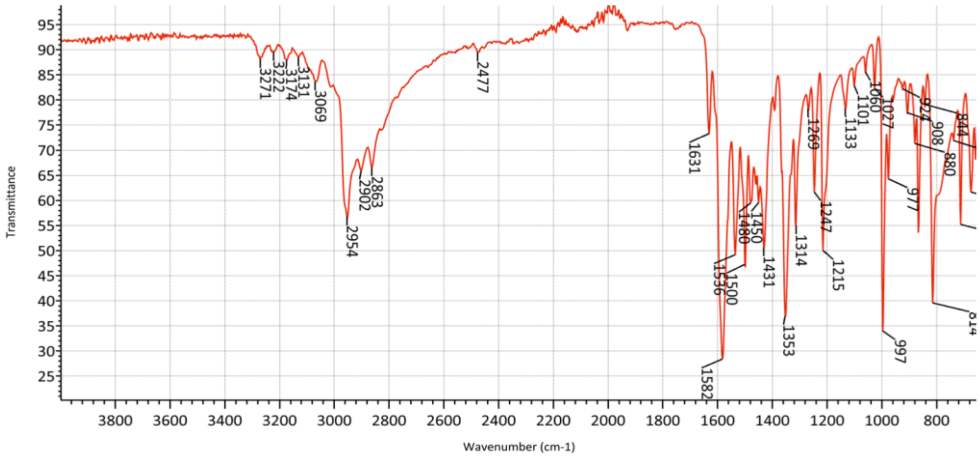

FT-IR (Diamond-ATR, neat, cm^{-1}) of P215

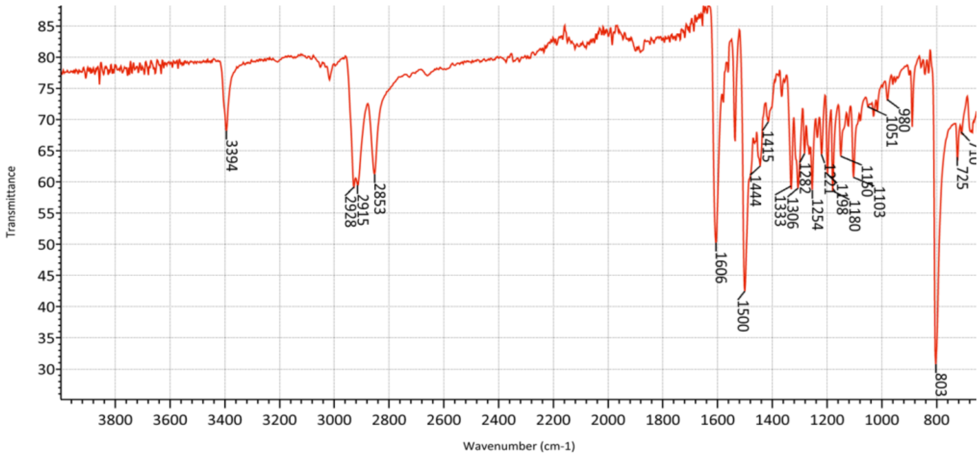

FT-IR (Diamond-ATR, neat, cm^{-1}) of P216

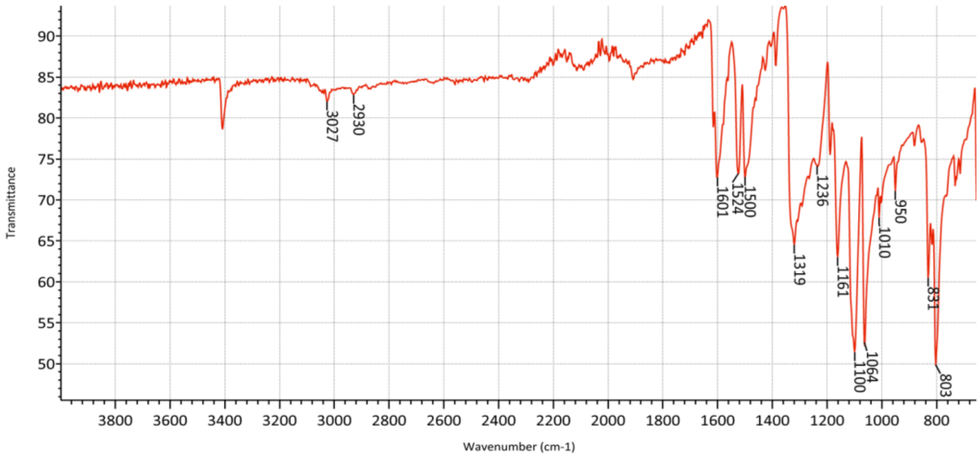

FT-IR (Diamond-ATR, neat, cm^{-1}) of P217

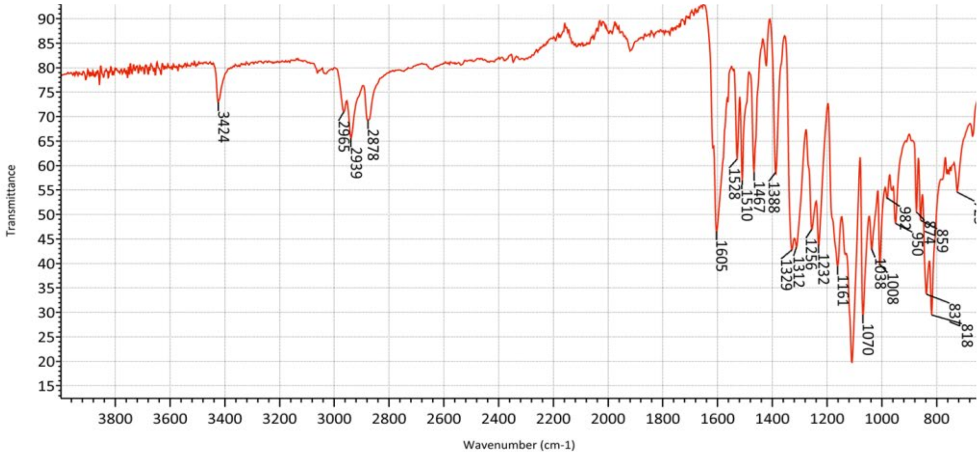

FT-IR (Diamond-ATR, neat, cm^{-1}) of P218

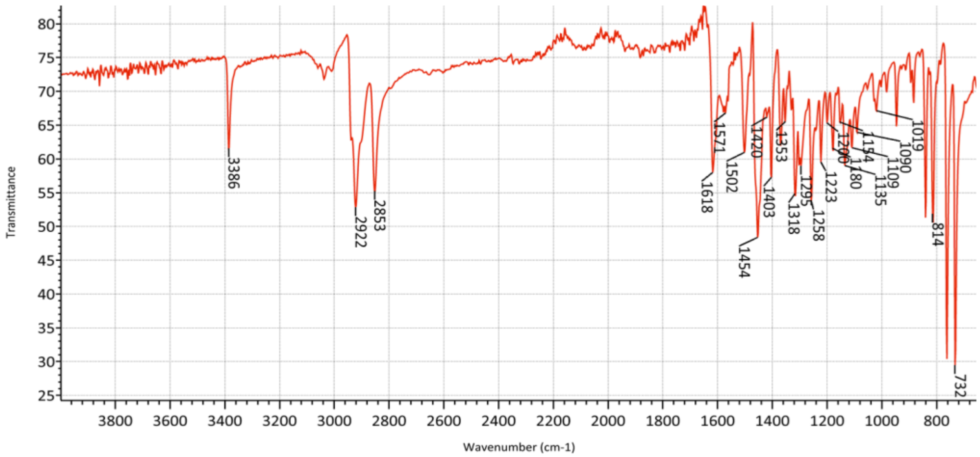

FT-IR (Diamond-ATR, neat, cm^{-1}) of P219

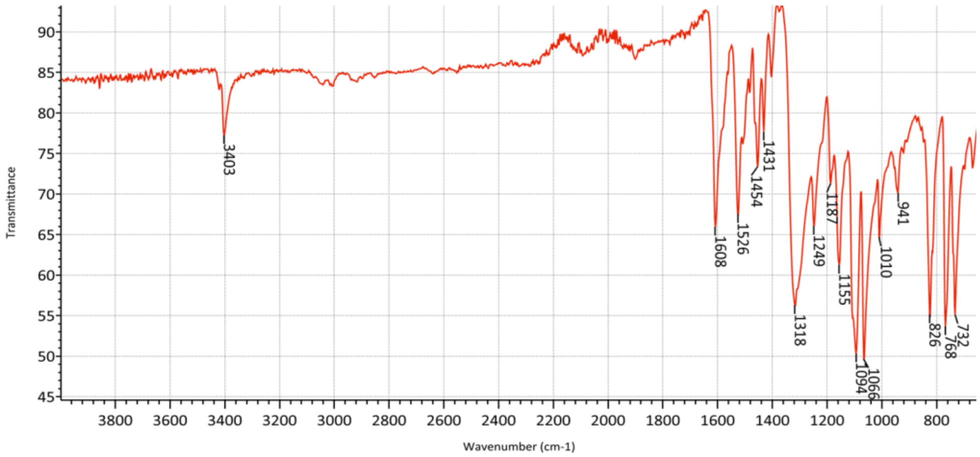

FT-IR (Diamond-ATR, neat, cm^{-1}) of P300

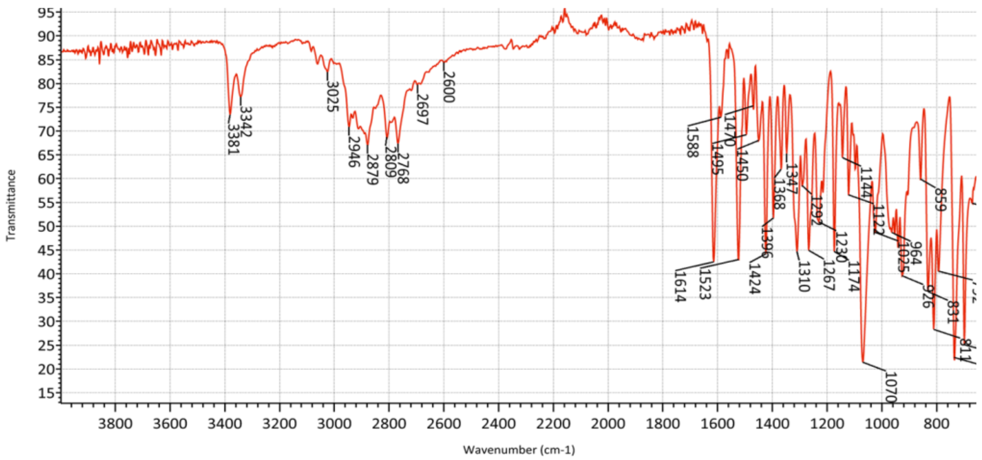

FT-IR (Diamond-ATR, neat, cm^{-1}) of P301

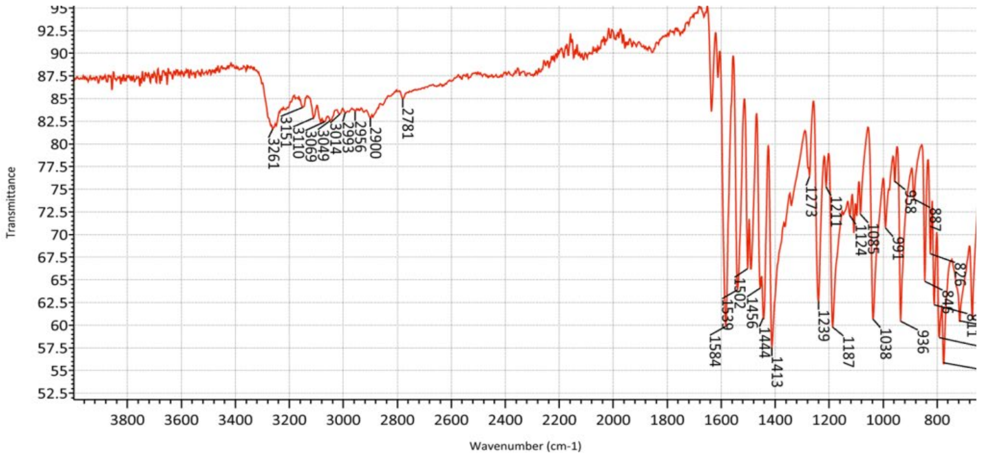

Mass Analysis Spectra of C–N Coupling Products

HRMS (TOF MS ES+) Spectra of P112

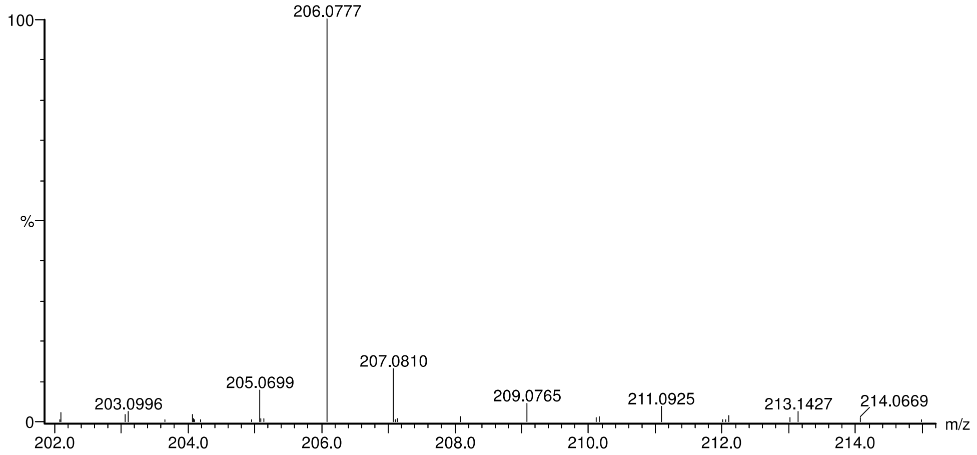

HRMS (TOF MS ES+) Spectra of P113

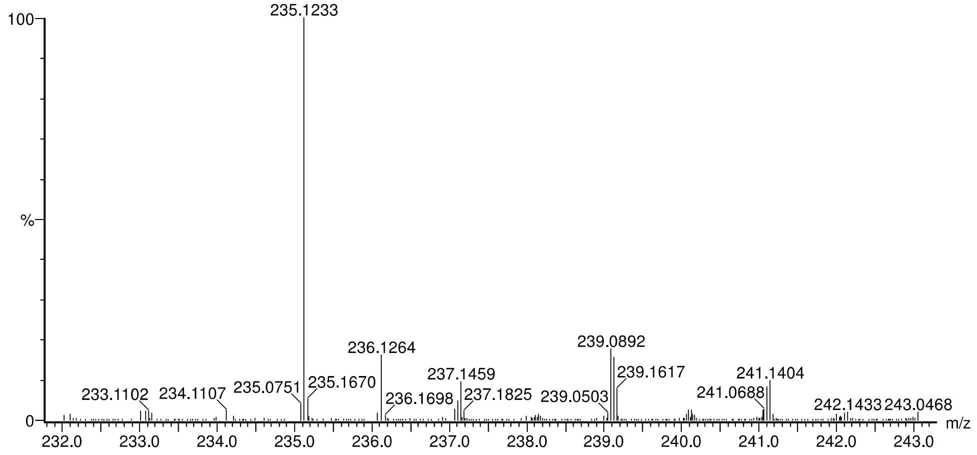

HRMS (TOF MS ES+) Spectra of P114

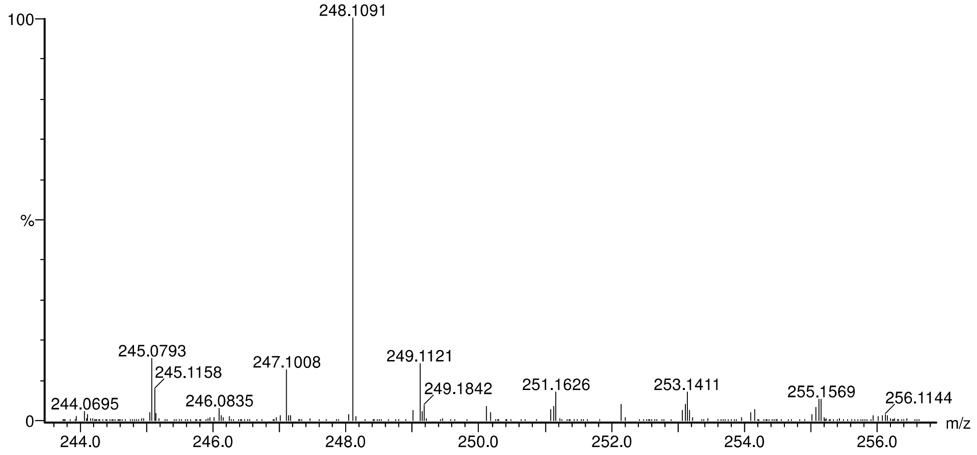

HRMS (TOF MS ES+) Spectra of P115

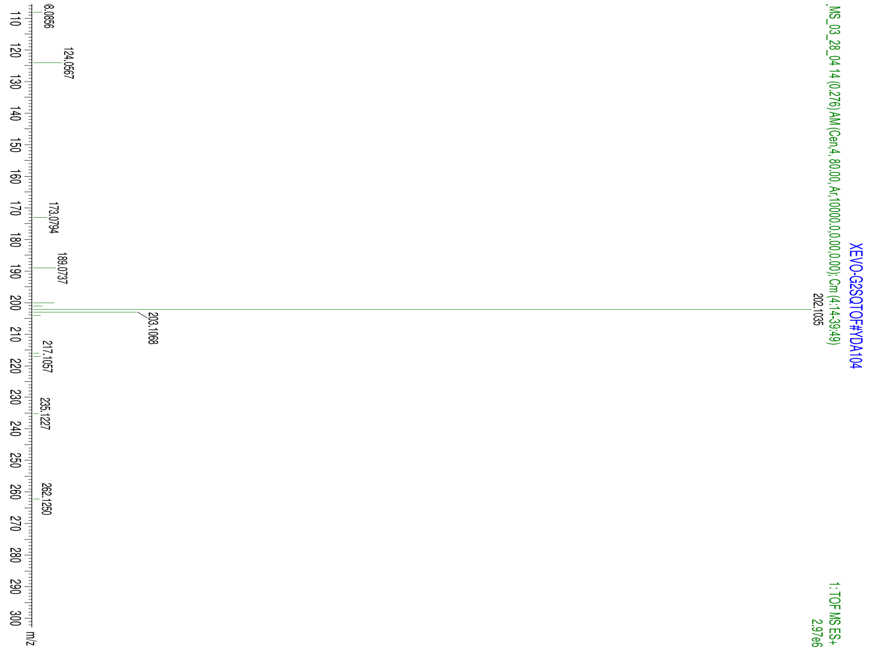

HRMS (TOF MS ES+) Spectra of P116

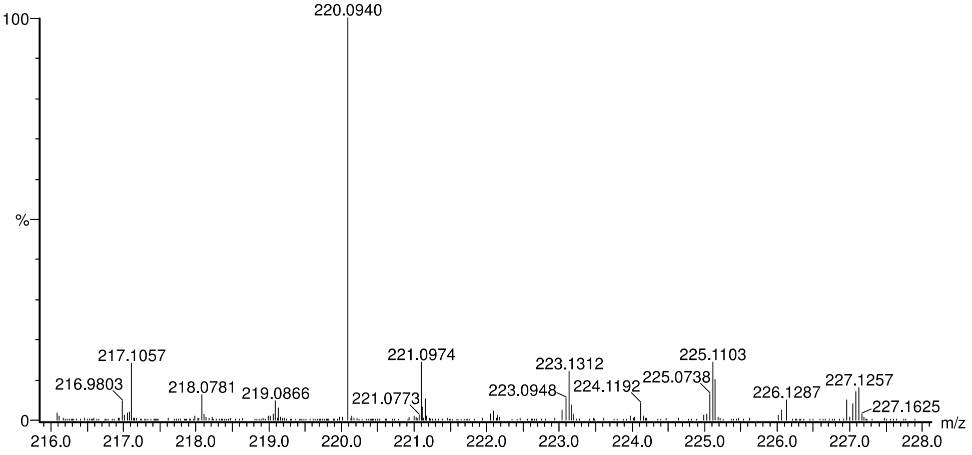

HRMS (TOF MS ES+) Spectra of P117

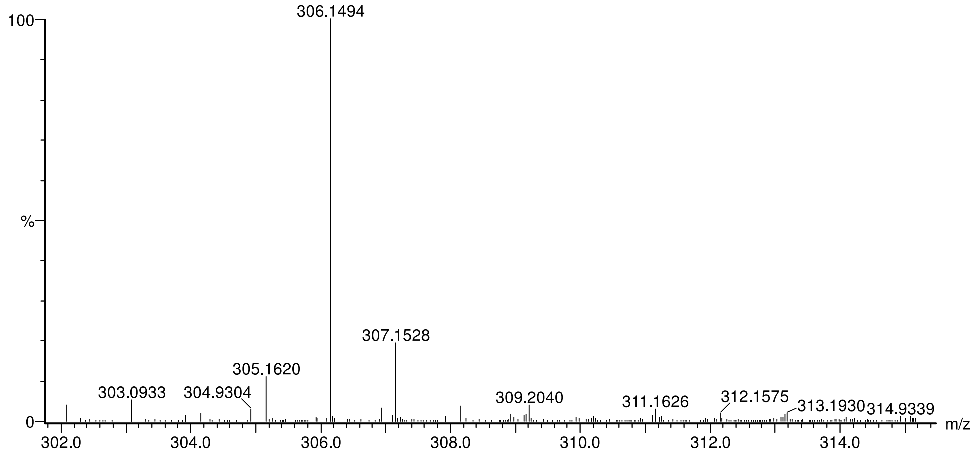

HRMS (TOF MS ES+) Spectra of P118

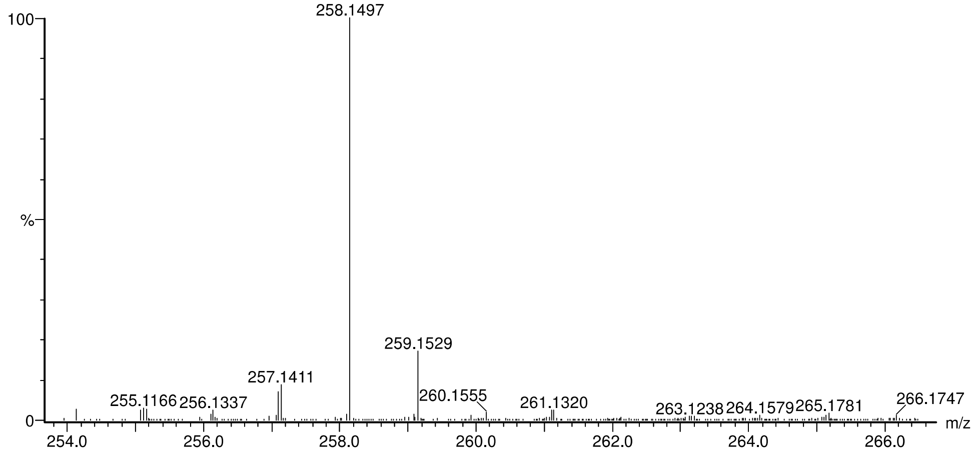

HRMS (TOF MS ES+) Spectra of P119

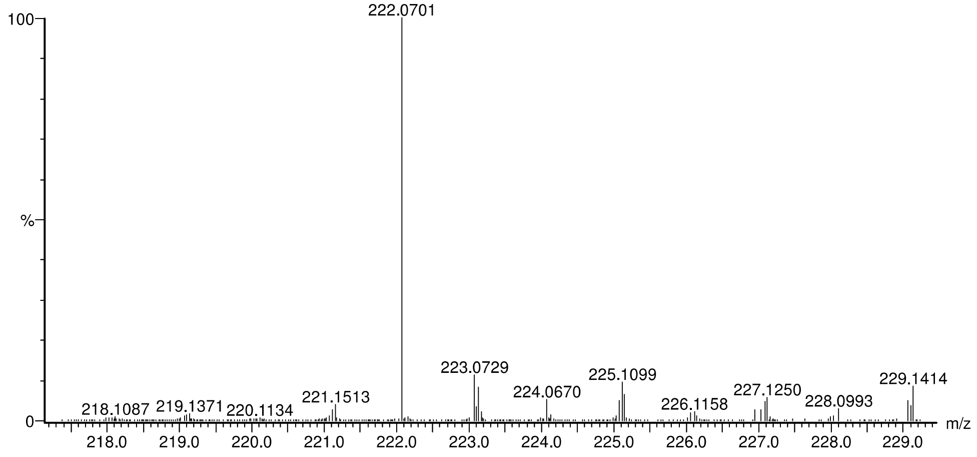

HRMS (TOF MS ES+) Spectra of P120

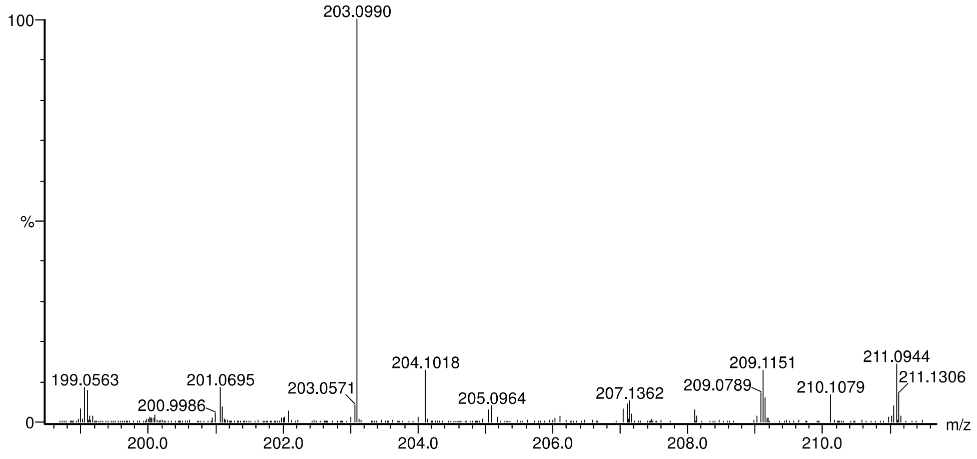

HRMS (TOF MS ES+) Spectra of P121

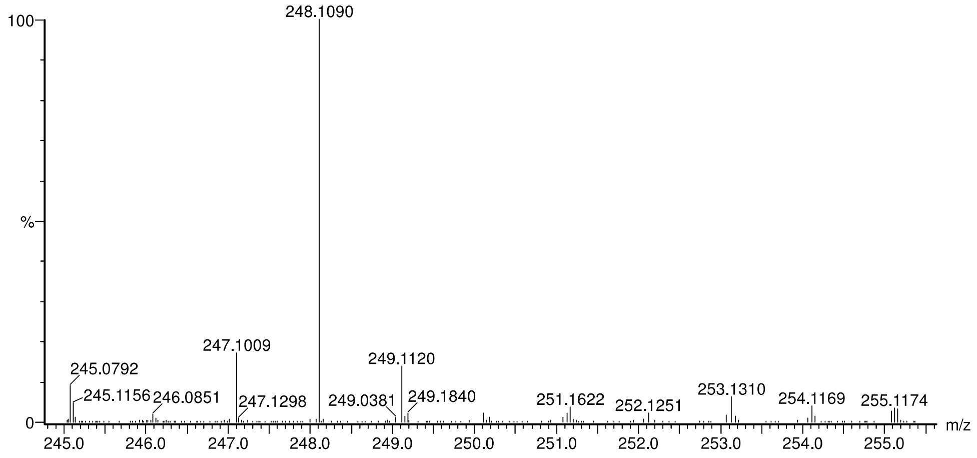

HRMS (TOF MS ES+) Spectra of P122

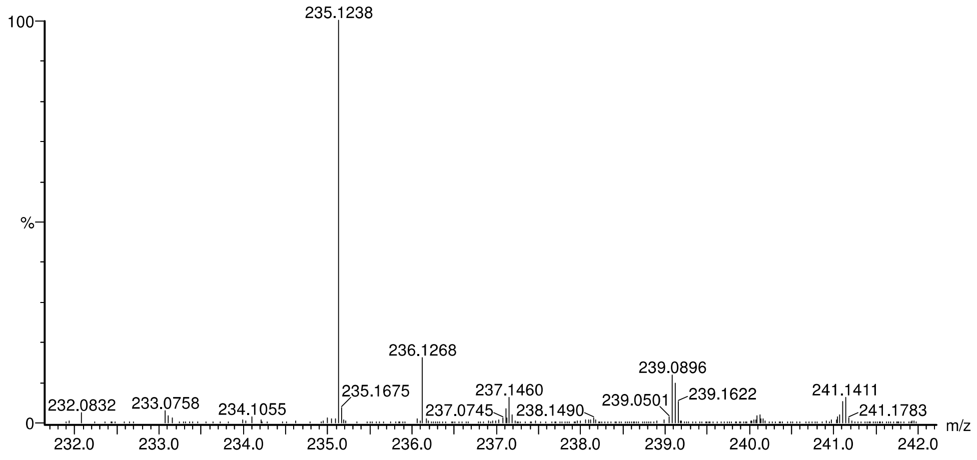

HRMS (TOF MS ES+) Spectra of P123

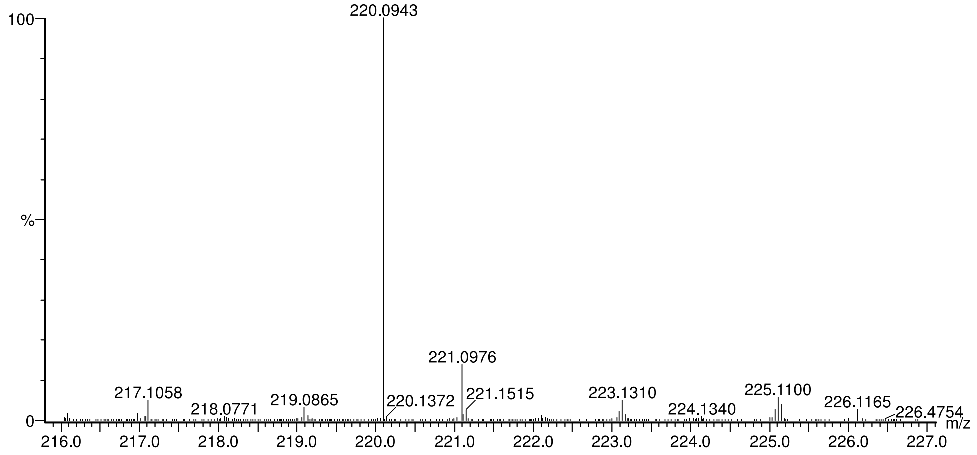

HRMS (TOF MS ES+) Spectra of P124

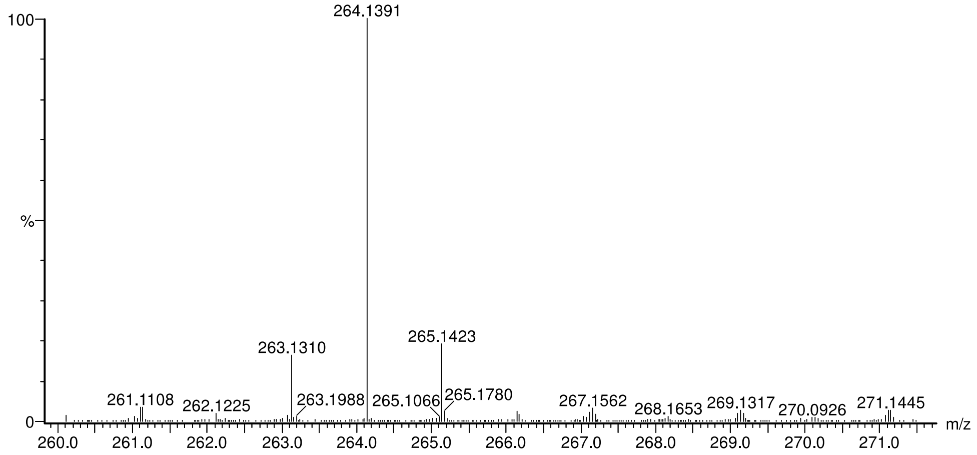

HRMS (TOF MS ES+) Spectra of P125

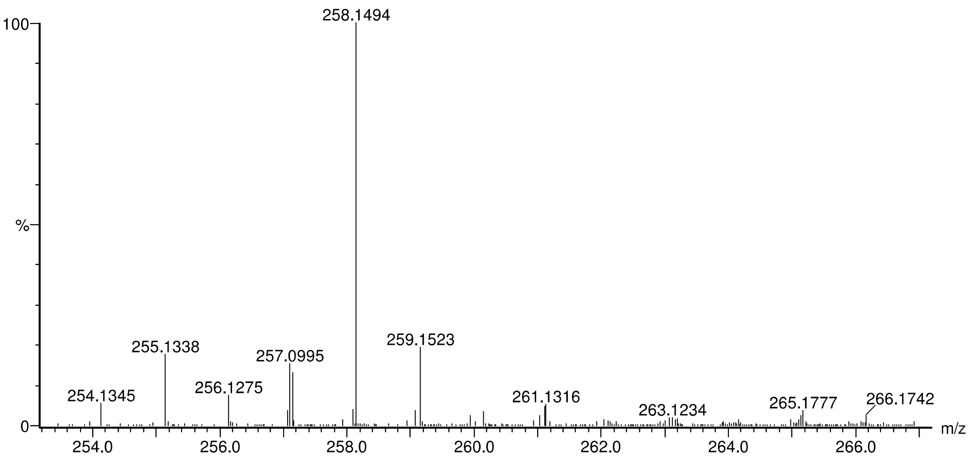

HRMS (TOF MS ES+) Spectra of P126

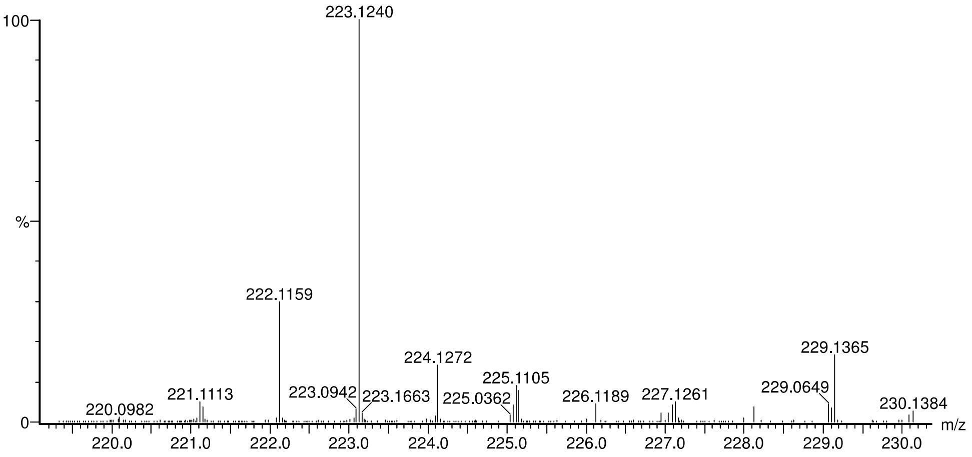

HRMS (TOF MS ES+) Spectra of P127

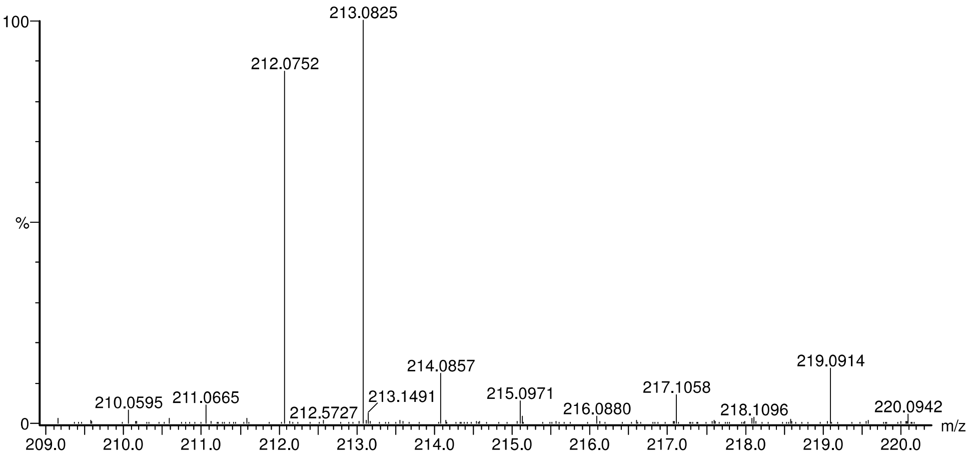

HRMS (TOF MS ES+) Spectra of P200

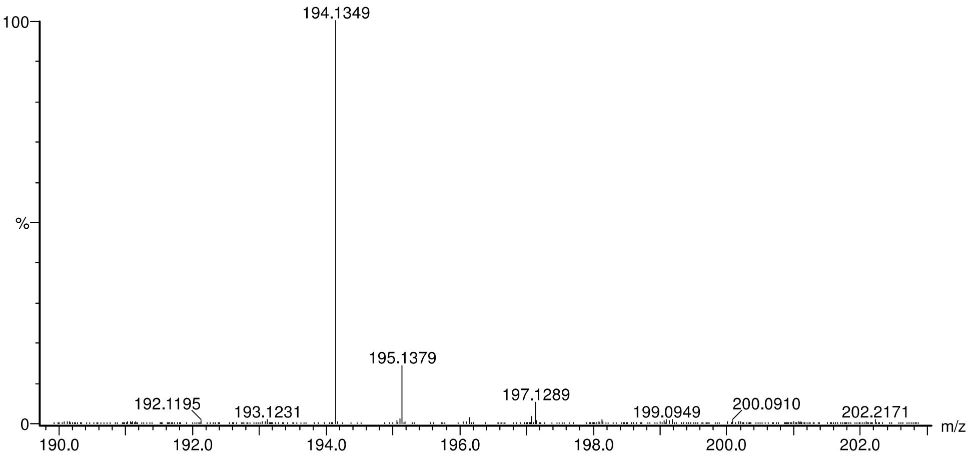

HRMS (TOF MS ES+) Spectra of P201

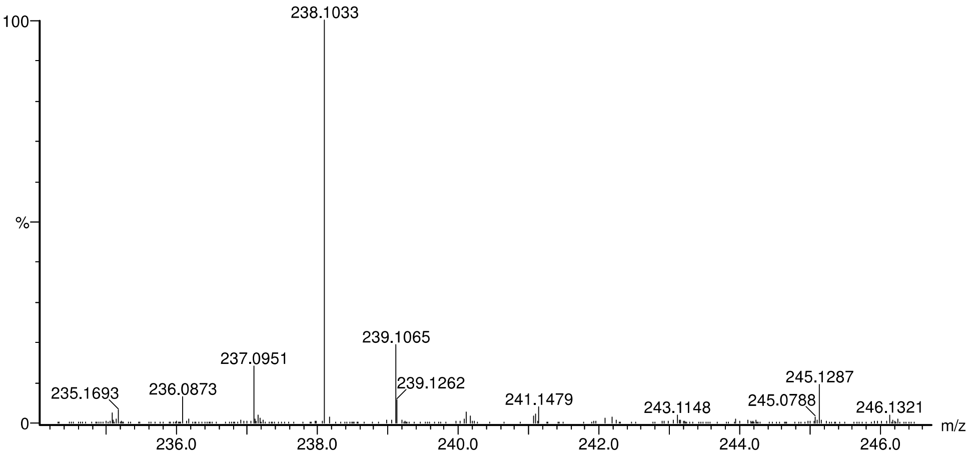

HRMS (TOF MS ES+) Spectra of P202

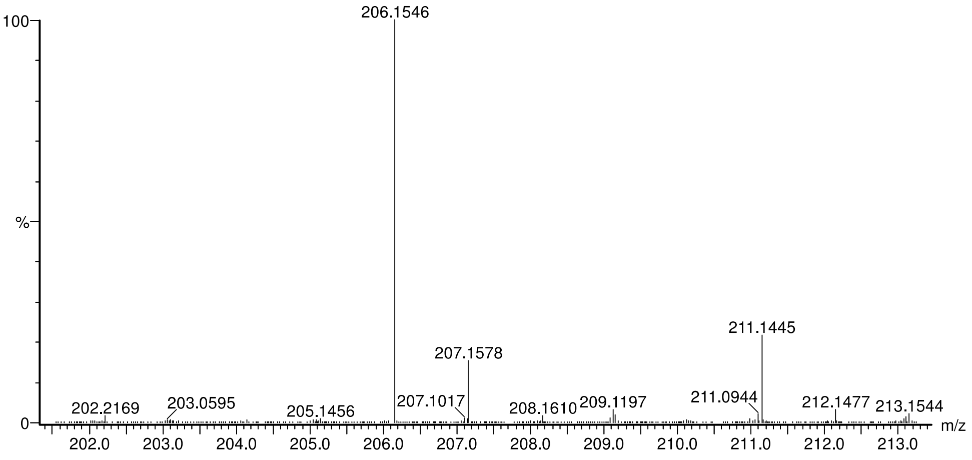

HRMS (TOF MS ES+) Spectra of P203

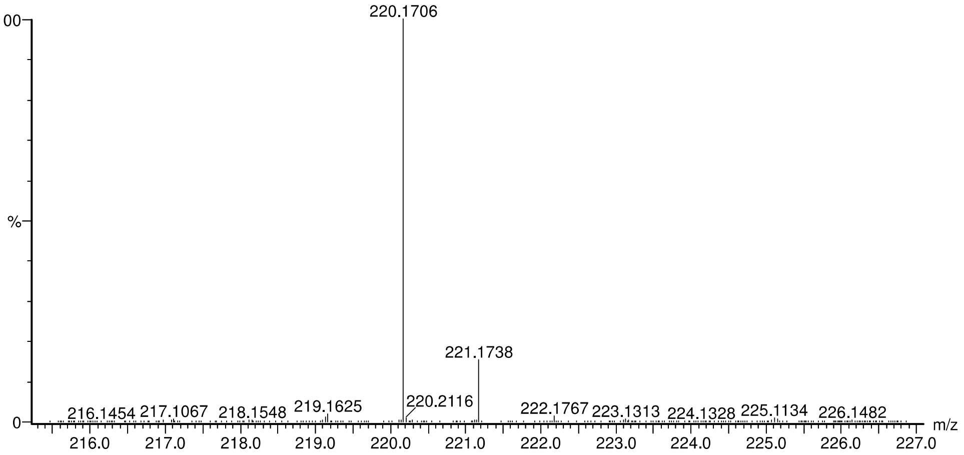

HRMS (TOF MS ES+) Spectra of P204

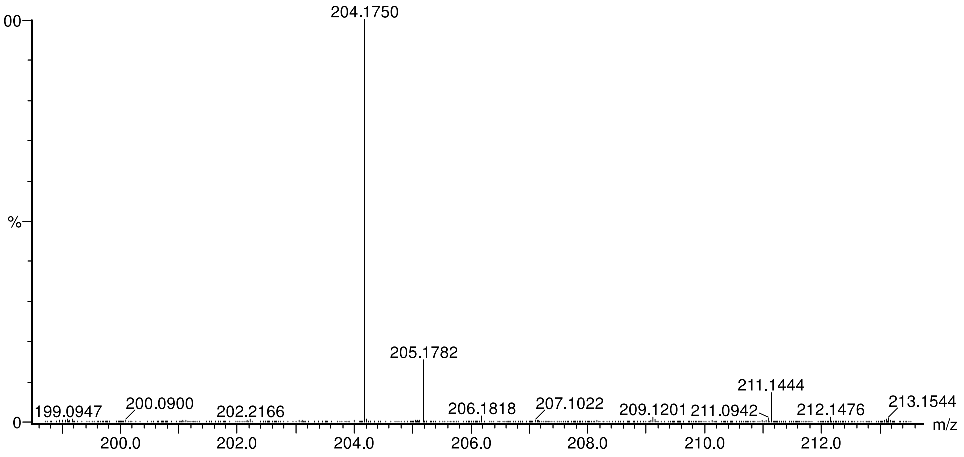

HRMS (TOF MS ES+) Spectra of P205

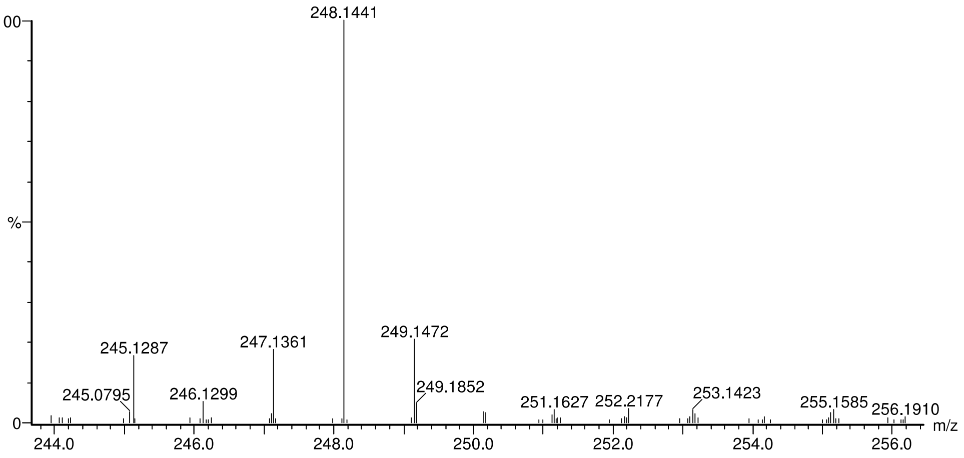

HRMS (TOF MS ES+) Spectra of P206

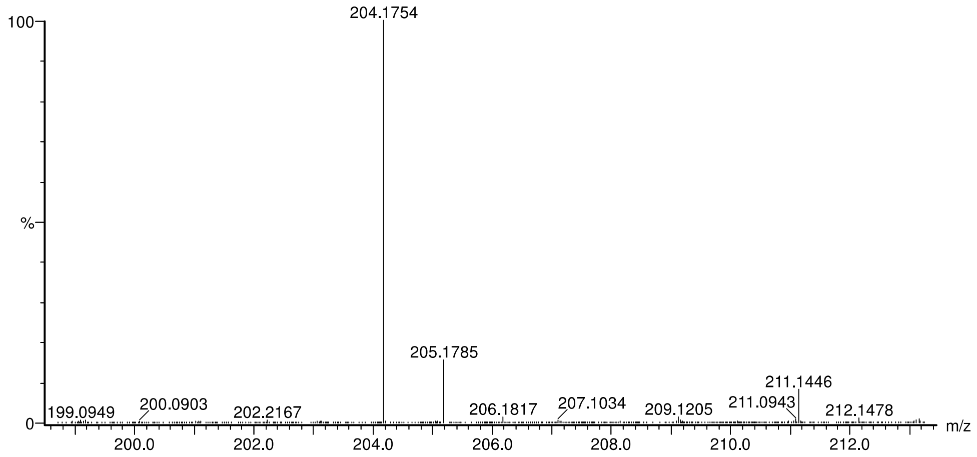

HRMS (TOF MS ES+) Spectra of P207

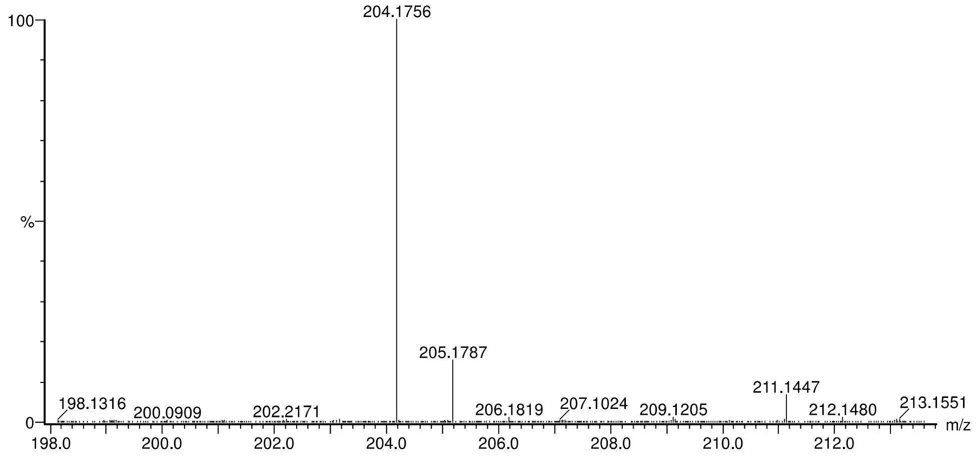

HRMS (TOF MS ES+) Spectra of P208

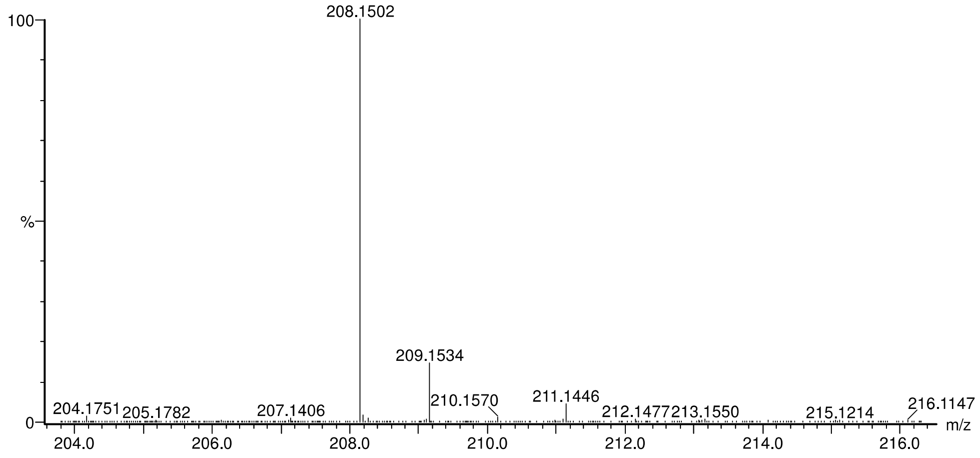

HRMS (TOF MS ES+) Spectra of P209

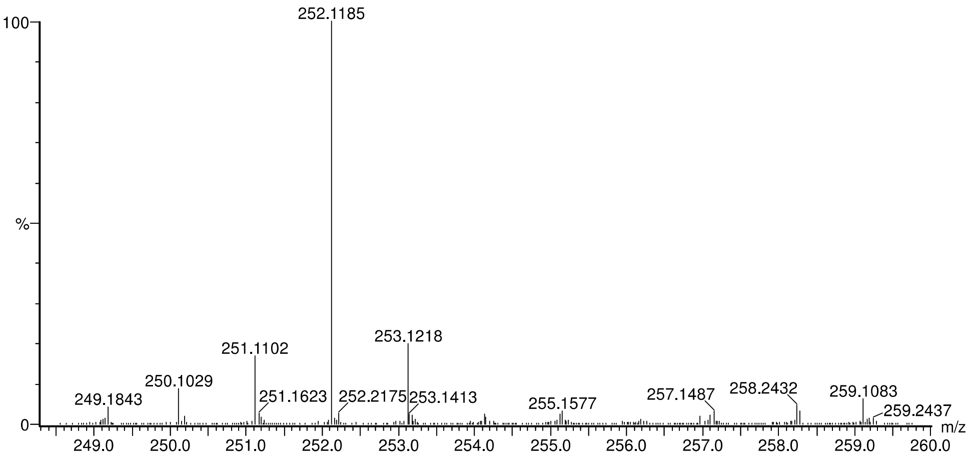

HRMS (TOF MS ES+) Spectra of P210

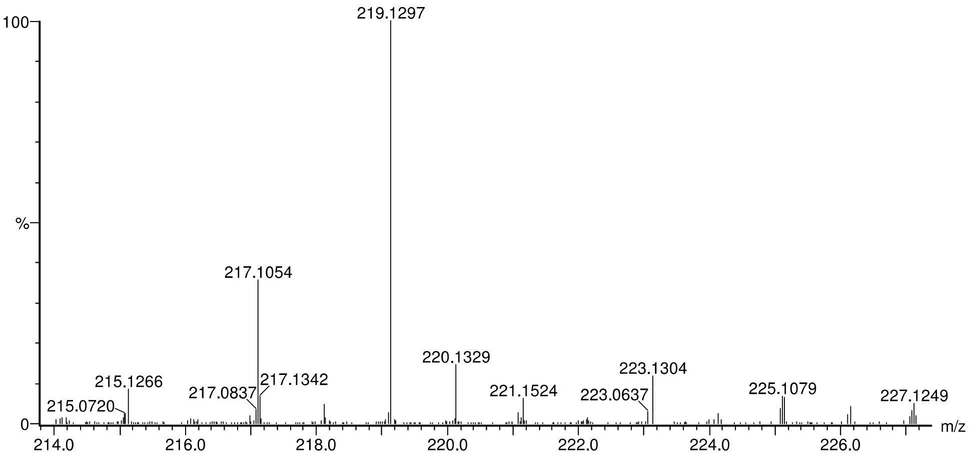

HRMS (TOF MS ES+) Spectra of P211

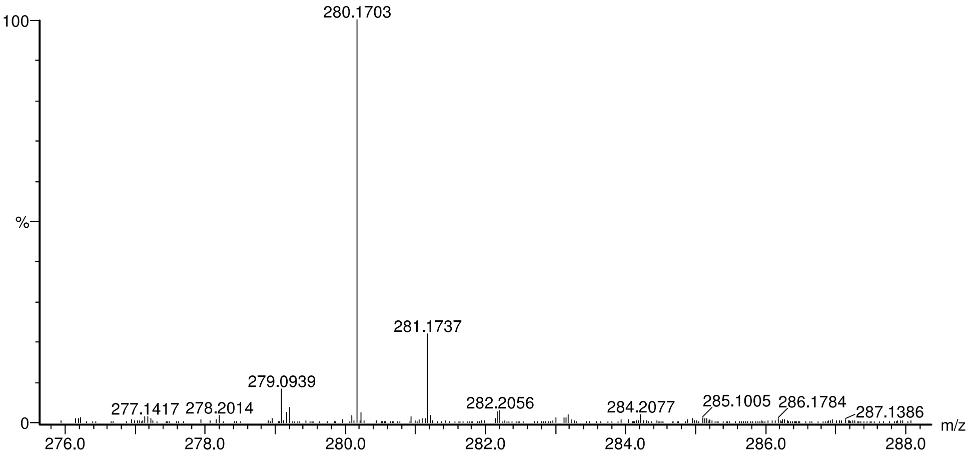

HRMS (TOF MS ES+) Spectra of P212

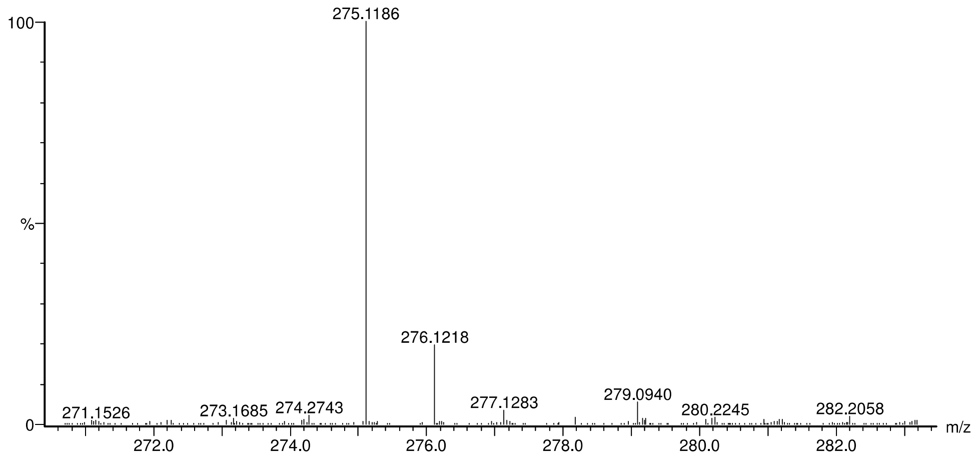

HRMS (TOF MS ES+) Spectra of P213

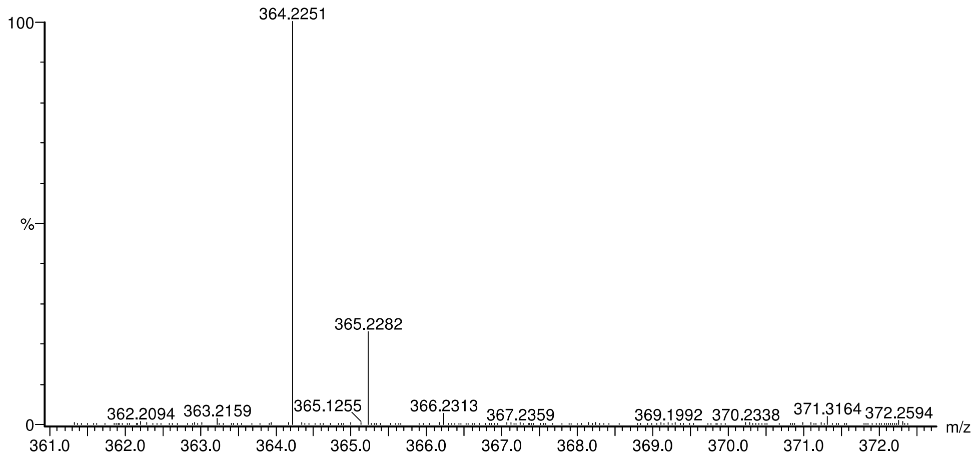

HRMS (TOF MS ES+) Spectra of P214

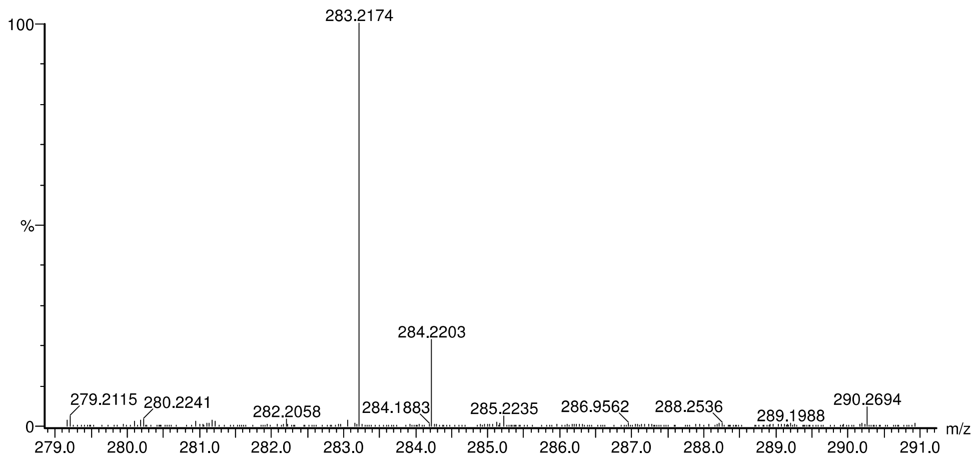

HRMS (TOF MS ES+) Spectra of P215

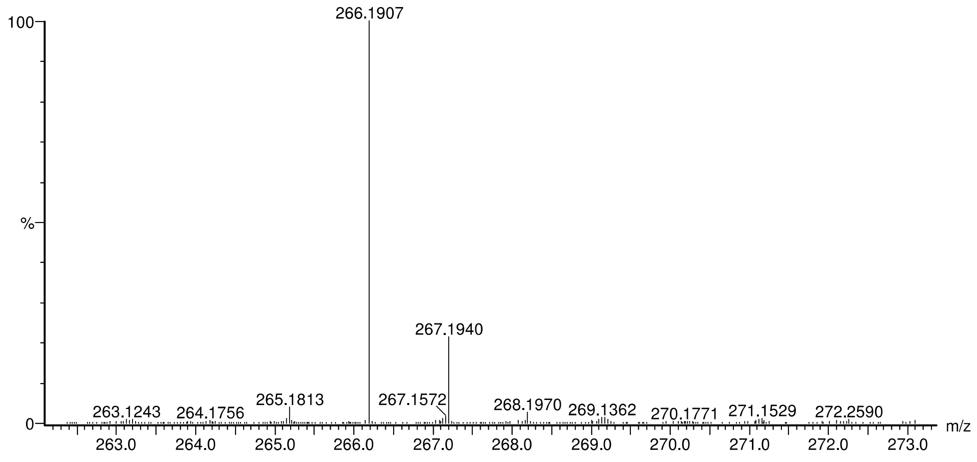

HRMS (TOF MS ES+) Spectra of P216

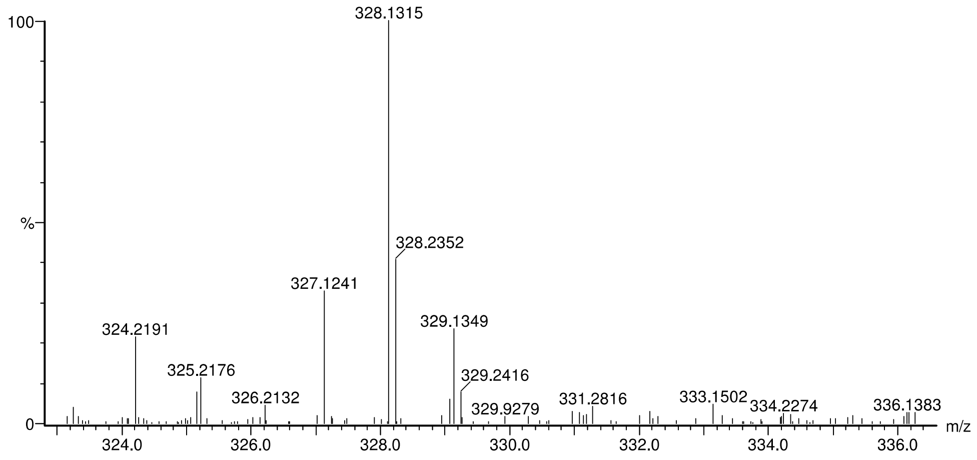

HRMS (TOF MS ES+) Spectra of P217

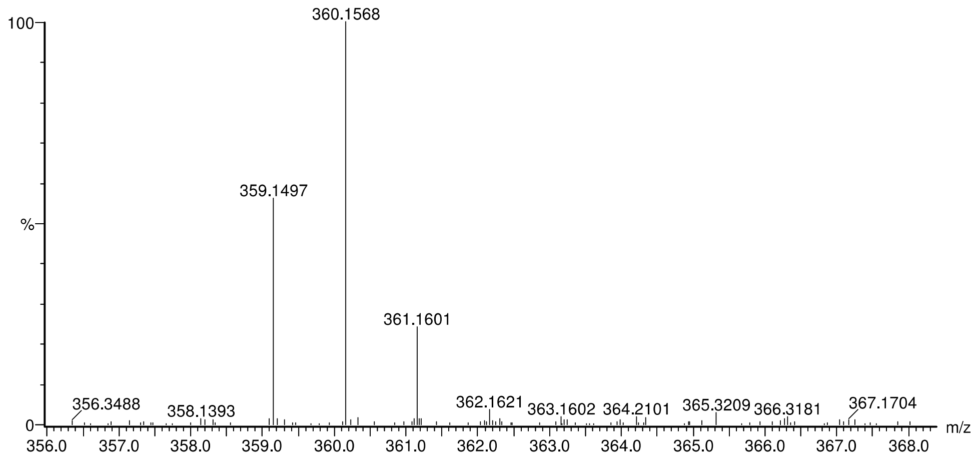

HRMS (TOF MS ES+) Spectra of P218

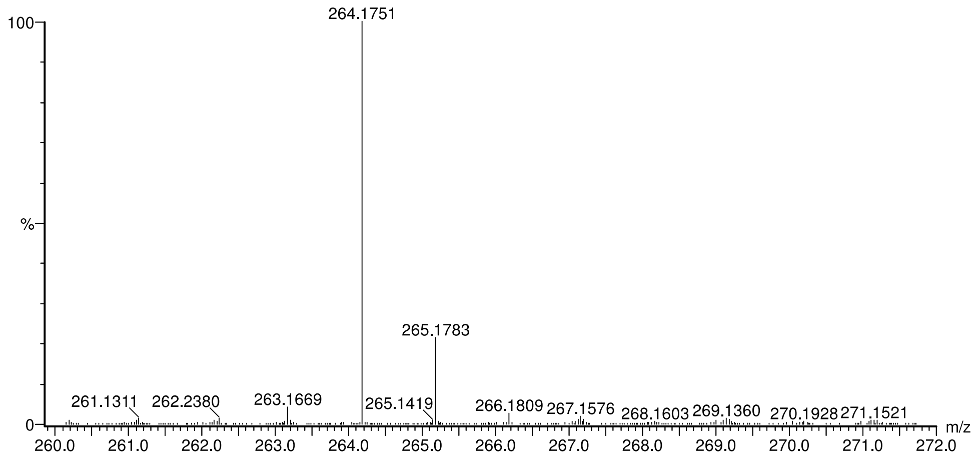

HRMS (TOF MS ES+) Spectra of P219

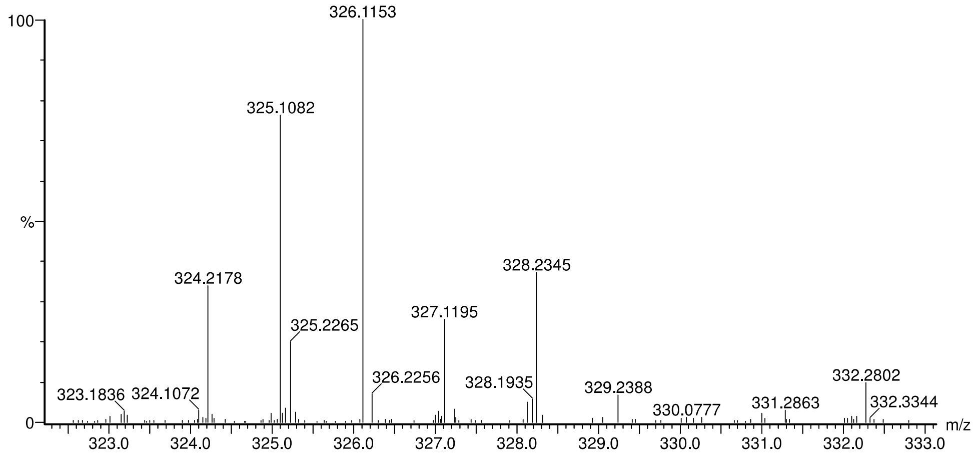

HRMS (TOF MS ES+) Spectra of P300

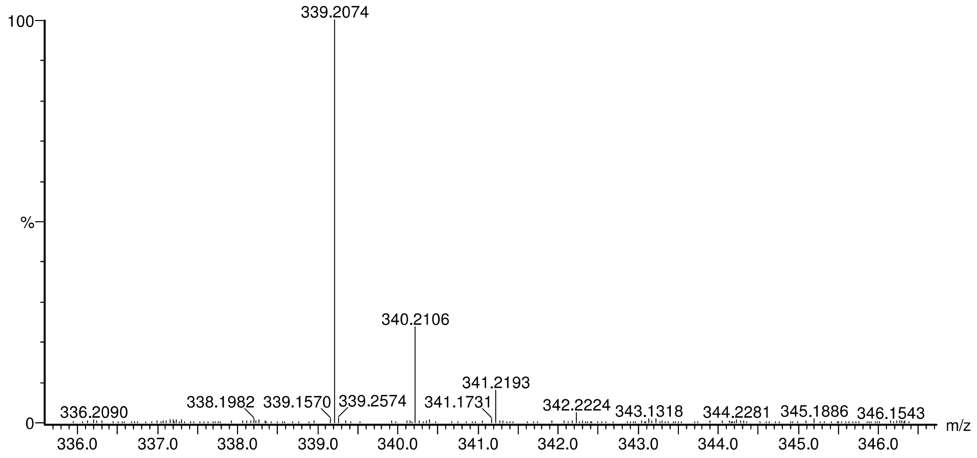

HRMS (TOF MS ES+) Spectra of P301

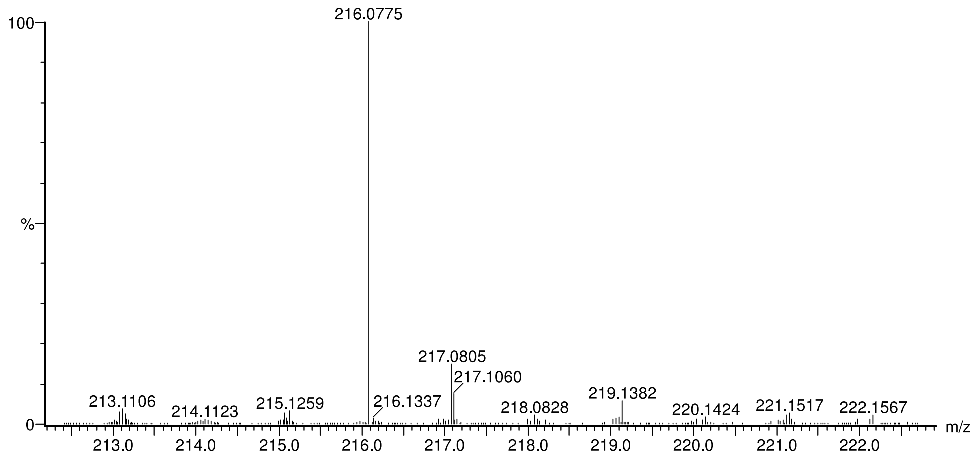

UPLC Chromatogram of Products

P100 Chromatogram with Ligand 36. Method: H_2O-MeCN 10% to 90%, 7 minutes. 1 µl injection on Acquity HSS Cyano 1.8µm 2.1mm x 100 mm column. PDA Spectrum obtained at 254 nm.

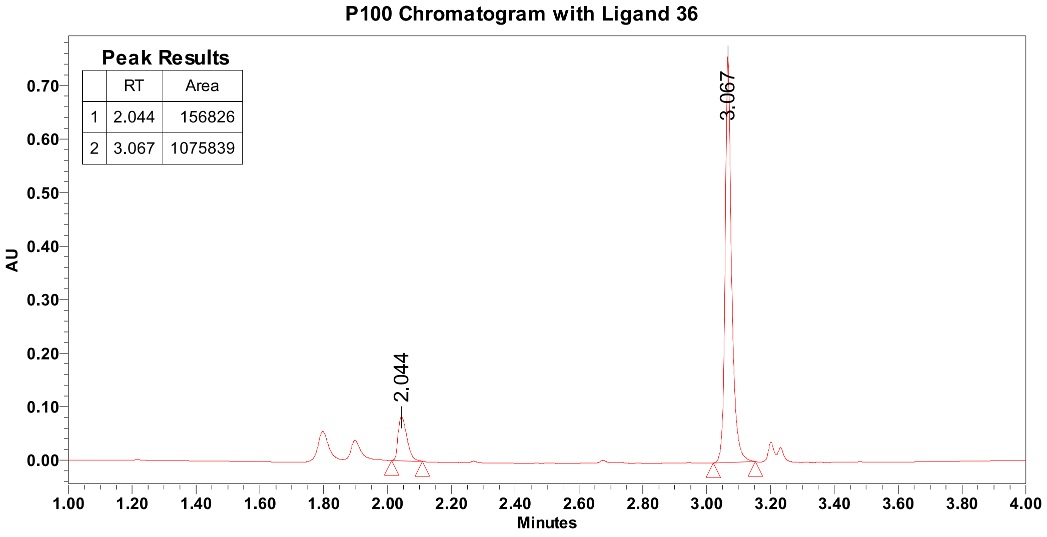

P101 Chromatogram with Ligand 36. Method: H_2O-MeCN 10% to 90%, 7 minutes. 1 µl injection on Acquity HSS Cyano 1.8µm 2.1mm x 100 mm column. PDA Spectrum obtained at 254 nm.

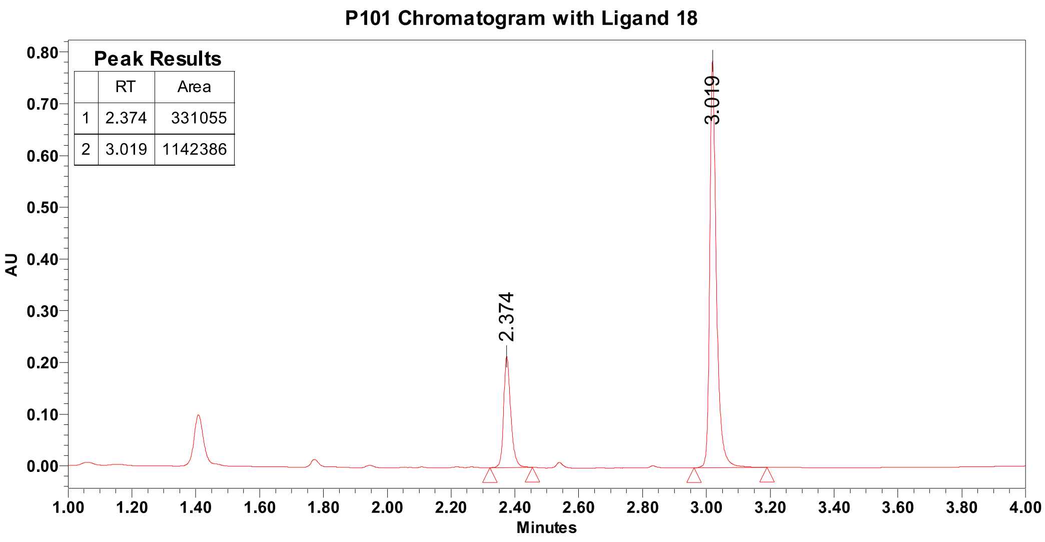

P102 Chromatogram with Ligand 36. Method: H_2O-MeCN 10% to 90%, 7 minutes. 1 µl injection on Acquity HSS Cyano 1.8µm 2.1mm x 100 mm column. PDA Spectrum obtained at 254 nm.

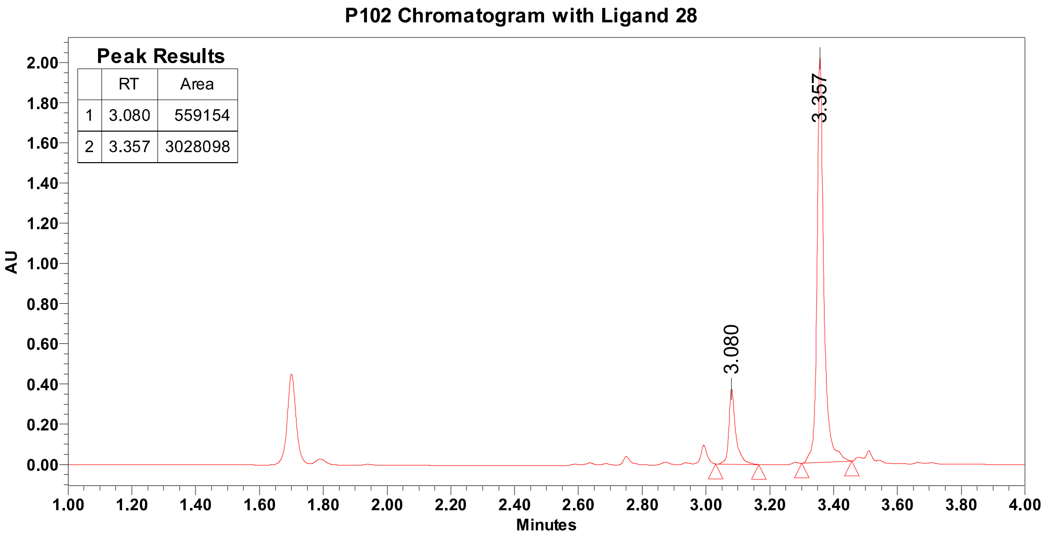

P103 Chromatogram with Ligand 36. Method: H_2O-MeCN 10% to 90%, 7 minutes. 1 µl injection on Acquity HSS Cyano 1.8µm 2.1mm x 100 mm column. PDA Spectrum obtained at 254 nm.

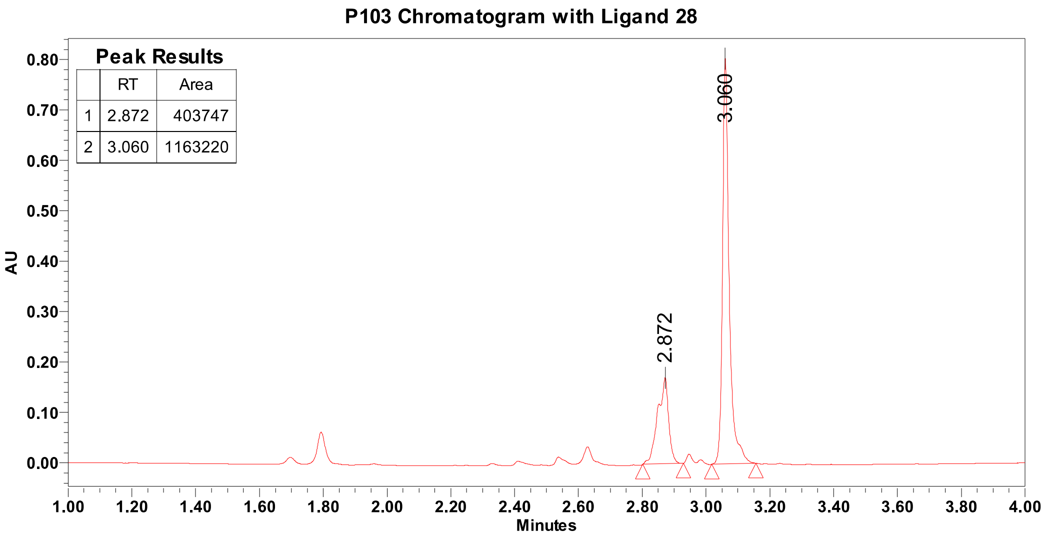

P104 Chromatogram with Ligand 36. Method: H_2O-MeCN 10% to 90%, 7 minutes. 1 µl injection on Acquity HSS Cyano 1.8µm 2.1mm x 100 mm column. PDA Spectrum obtained at 254 nm.

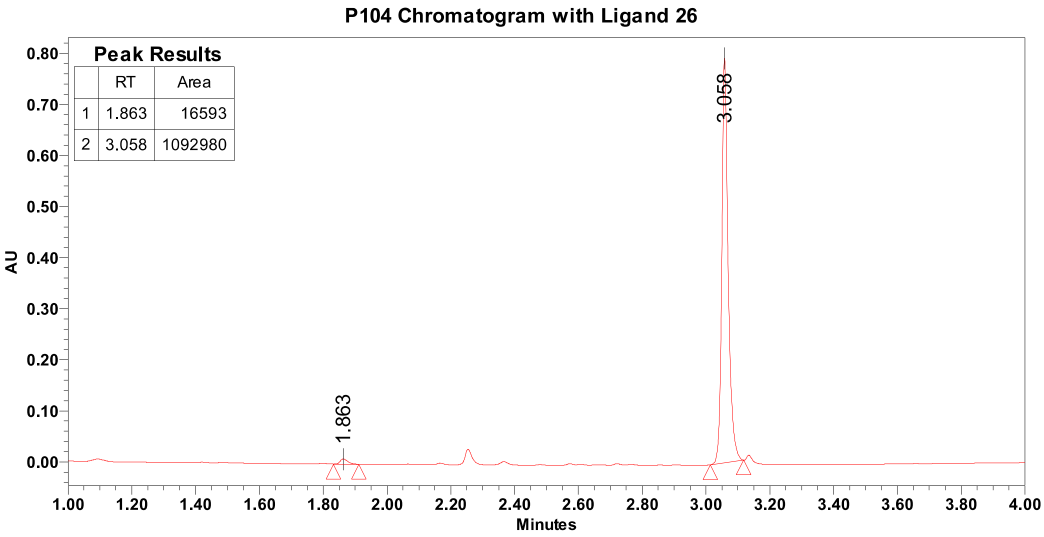

P105 Chromatogram with Ligand 36. Method: H_2O-MeCN 10% to 90%, 7 minutes. 1 µl injection on Acquity HSS Cyano 1.8µm 2.1mm x 100 mm column. PDA Spectrum obtained at 254 nm.

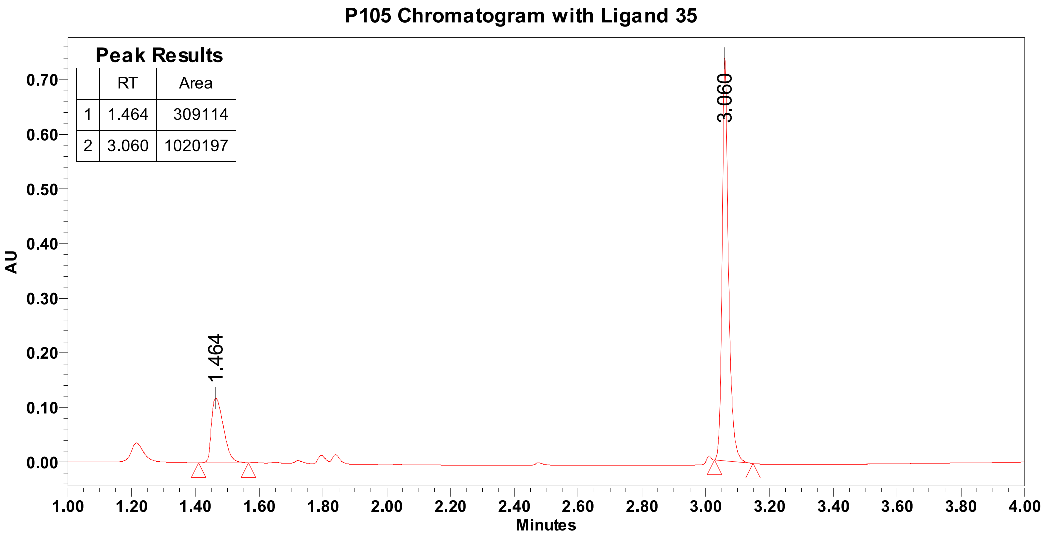

P106 Chromatogram with Ligand 36. Method: H_2O-MeCN 10% to 90%, 7 minutes. 1 µl injection on Acquity HSS Cyano 1.8µm 2.1mm x 100 mm column. PDA Spectrum obtained at 254 nm.

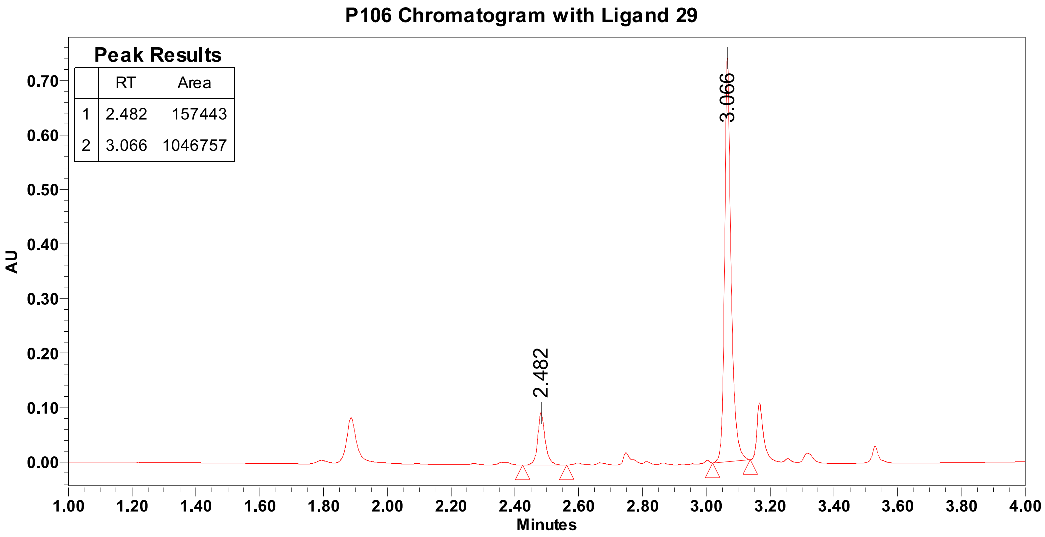

P107 Chromatogram with Ligand 36. Method: H_2O-MeCN 10% to 90%, 7 minutes. 1 µl injection on Acquity HSS Cyano 1.8µm 2.1mm x 100 mm column. PDA Spectrum obtained at 254 nm.

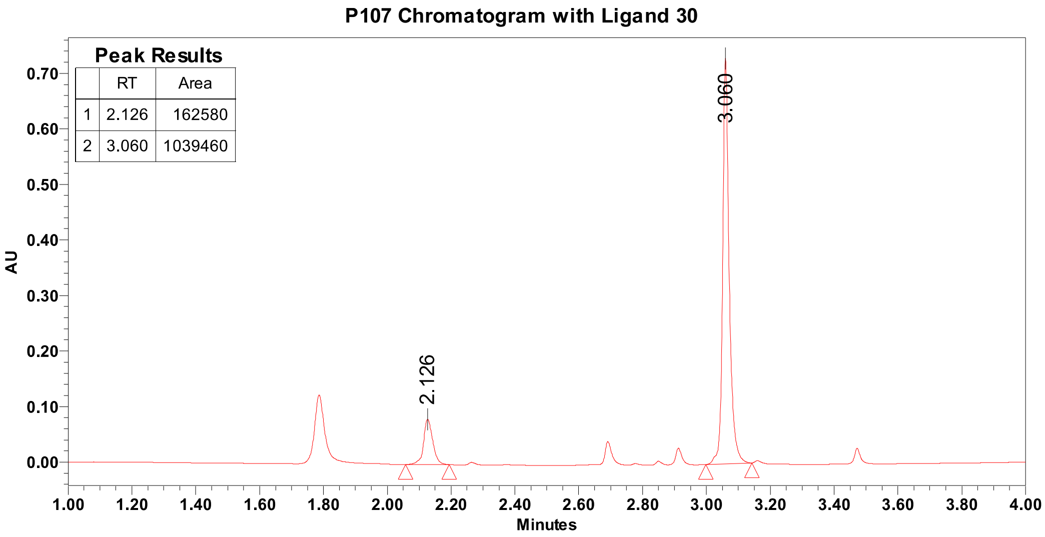

P108 Chromatogram with Ligand 36. Method: H_2O-MeCN 10% to 90%, 7 minutes. 1 µl injection on Acquity HSS Cyano 1.8µm 2.1mm x 100 mm column. PDA Spectrum obtained at 254 nm.

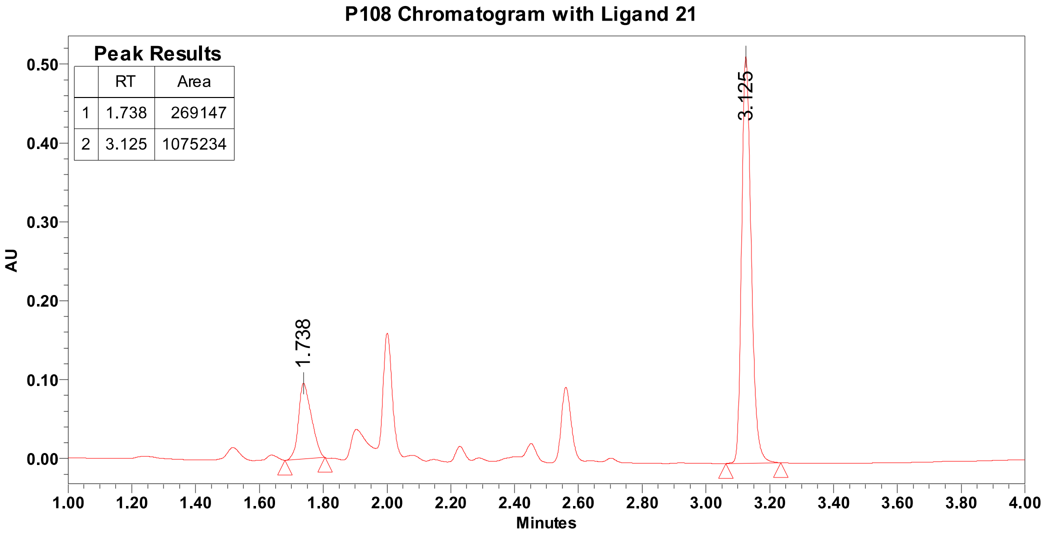

P109 Chromatogram with Ligand 36. Method: H_2O-MeCN 10% to 90%, 7 minutes. 1 µl injection on Acquity HSS Cyano 1.8µm 2.1mm x 100 mm column. PDA Spectrum obtained at 254 nm.

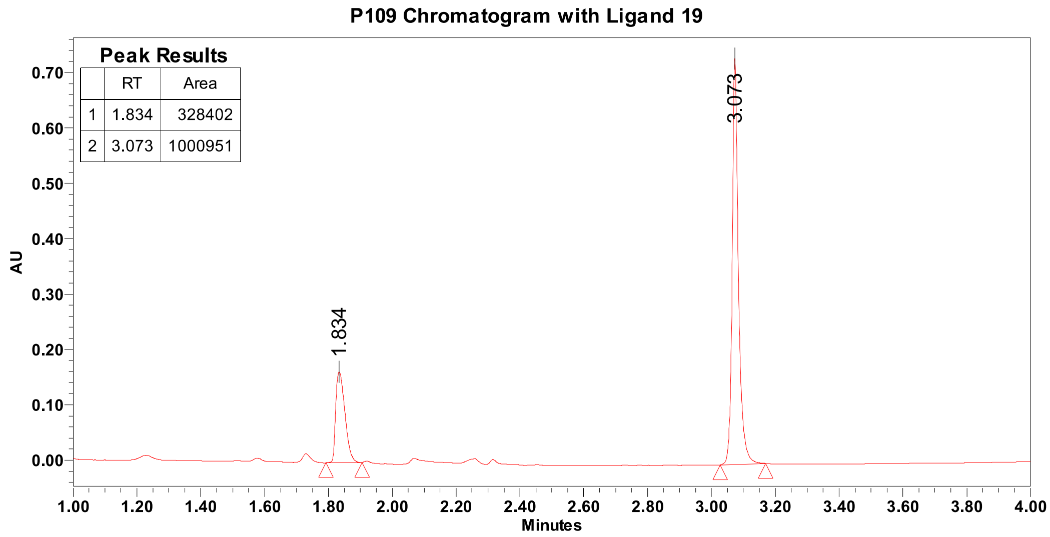

P110 Chromatogram with Ligand 36. Method: H_2O-MeCN 10% to 90%, 7 minutes. 1 µl injection on Acquity HSS Cyano 1.8µm 2.1mm x 100 mm column. PDA Spectrum obtained at 254 nm.

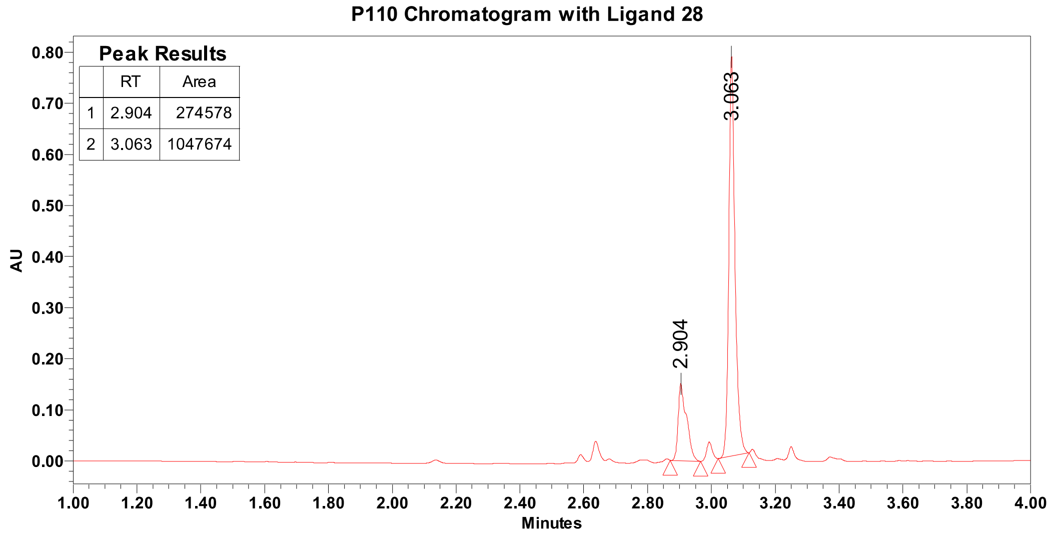

P111 Chromatogram with Ligand 36. Method: H_2O-MeCN 10% to 90%, 7 minutes. 1 µl injection on Acquity HSS Cyano 1.8µm 2.1mm x 100 mm column. PDA Spectrum obtained at 254 nm.

P111 Chromatogram with Ligand 29

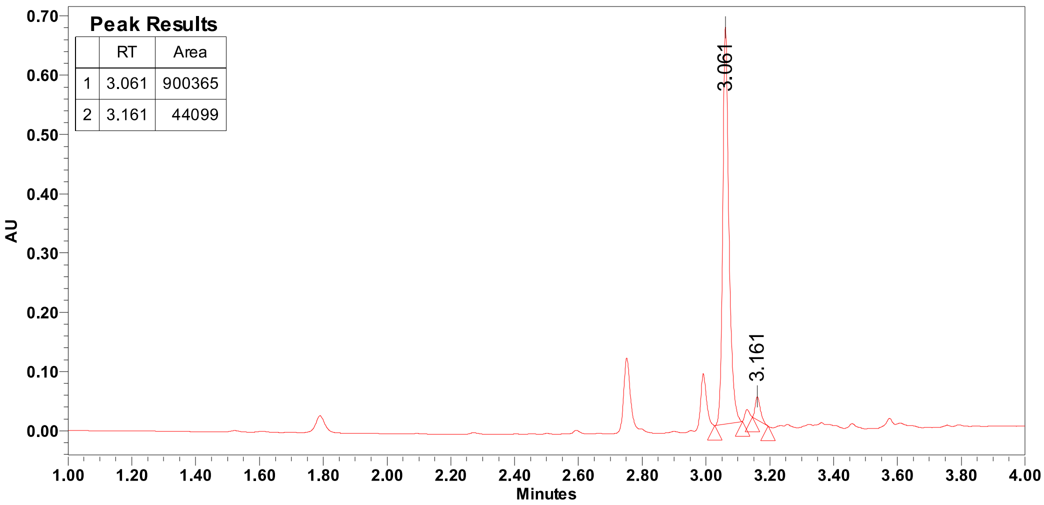

P112 Chromatogram with Ligand 36. Method: H_2O-MeCN 10% to 90%, 7 minutes. 1 µl injection on Acquity HSS Cyano 1.8µm 2.1mm x 100 mm column. PDA Spectrum obtained at 254 nm.

P112 Chromatogram with Ligand 24

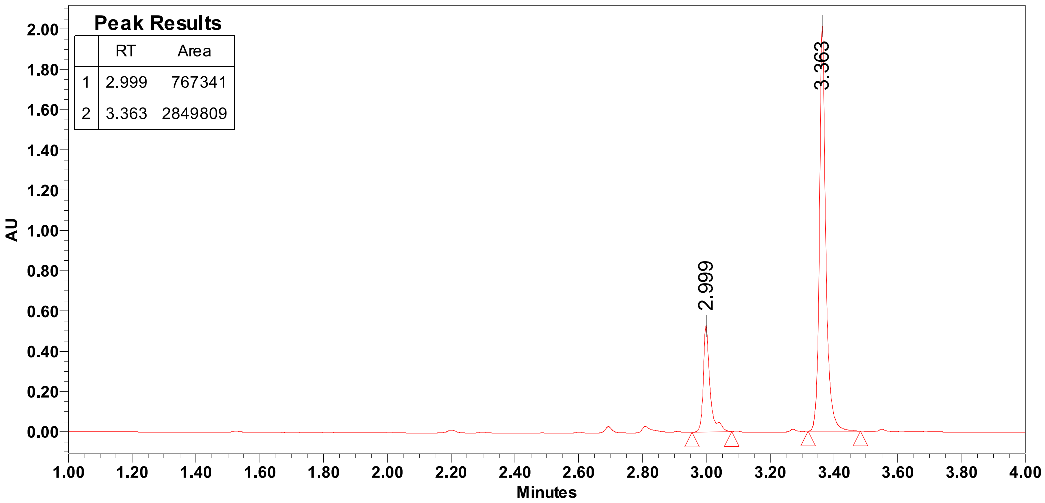

P113 Chromatogram with Ligand 36. Method: H_2O-MeCN 10% to 90%, 7 minutes. 1 µl injection on Acquity HSS Cyano 1.8µm 2.1mm x 100 mm column. PDA Spectrum obtained at 254 nm.

P113 Chromatogram with Ligand 28

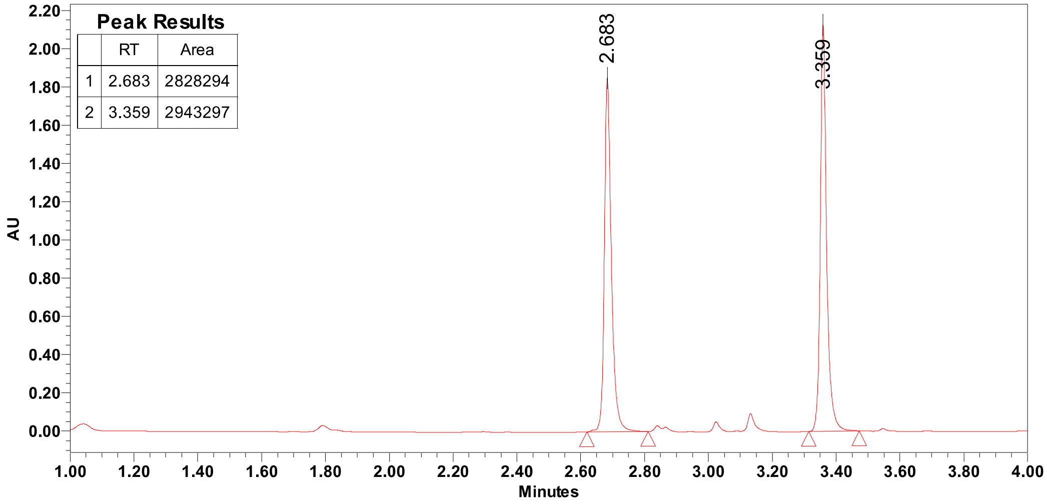

P114 Chromatogram with Ligand 36. Method: H_2O-MeCN 10% to 90%, 7 minutes. 1 µl injection on Acquity HSS Cyano 1.8µm 2.1mm x 100 mm column. PDA Spectrum obtained at 254 nm.

P114 Chromatogram with Ligand 30

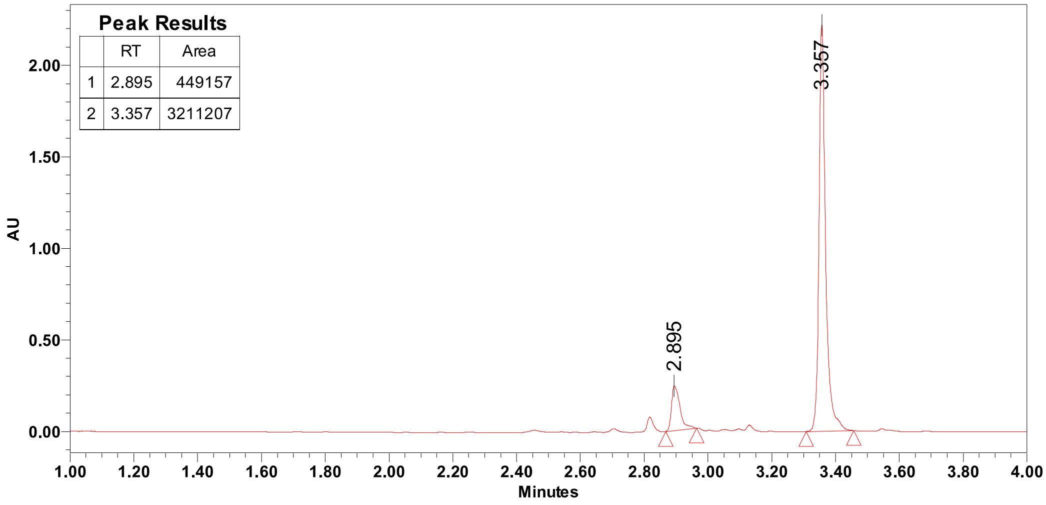

P115 Chromatogram with Ligand 36. Method: H_2O-MeCN 10% to 90%, 7 minutes. 1 µl injection on Acquity HSS Cyano 1.8µm 2.1mm x 100 mm column. PDA Spectrum obtained at 254 nm.

P115 Chromatogram with Ligand 30

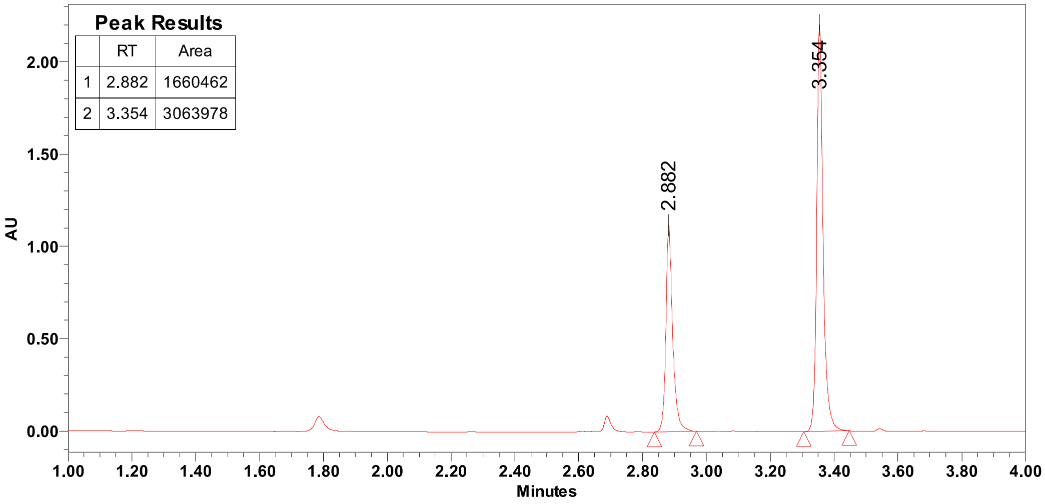

P116 Chromatogram with Ligand 36. Method: H_2O-MeCN 10% to 90%, 7 minutes. 1 µl injection on Acquity HSS Cyano 1.8µm 2.1mm x 100 mm column. PDA Spectrum obtained at 254 nm.

P116 Chromatogram with Ligand 30

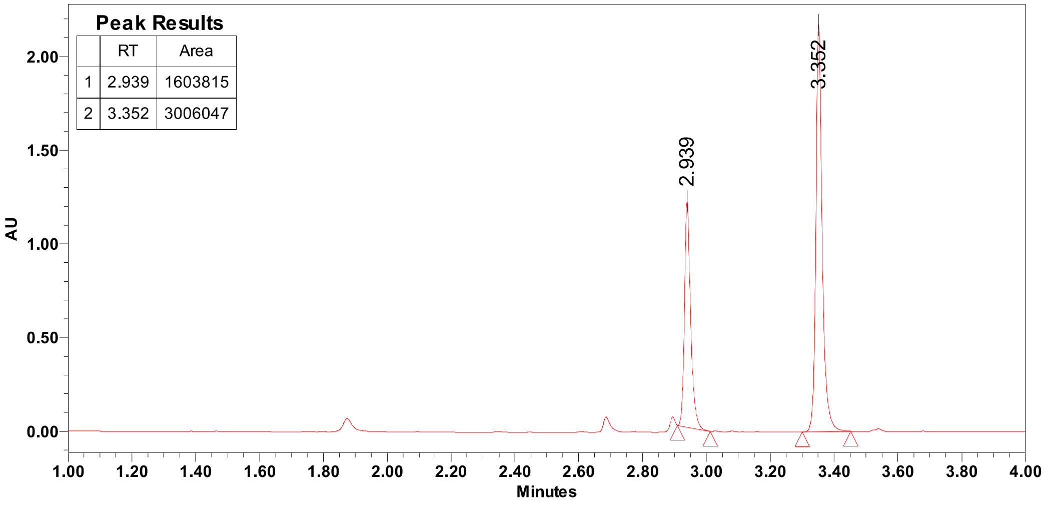

P117 Chromatogram with Ligand 36. Method: H_2O-MeCN 10% to 90%, 7 minutes. 1 µl injection on Acquity HSS Cyano 1.8µm 2.1mm x 100 mm column. PDA Spectrum obtained at 254 nm.

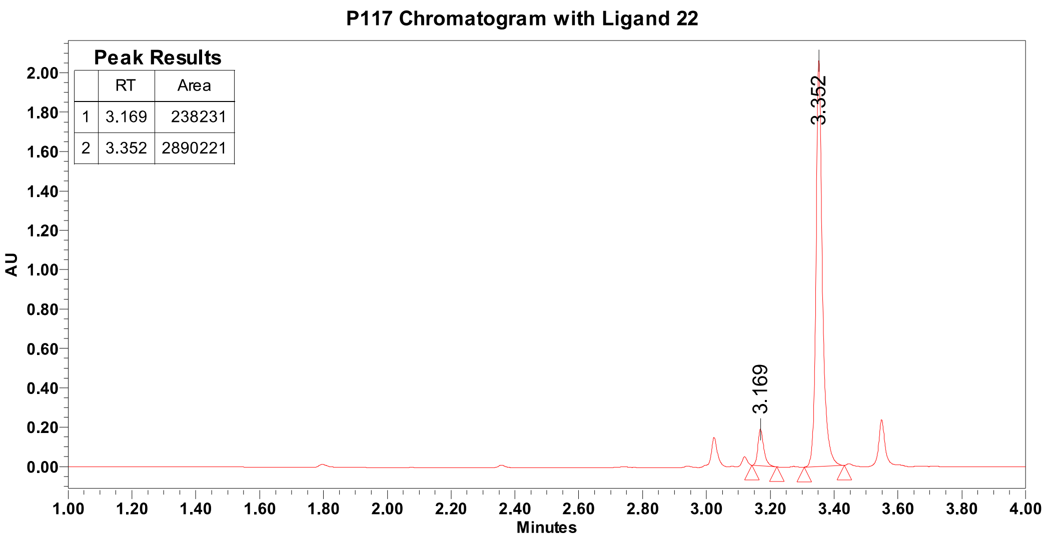

P118 Chromatogram with Ligand 36. Method: H_2O-MeCN 10% to 90%, 7 minutes. 1 µl injection on Acquity HSS Cyano 1.8µm 2.1mm x 100 mm column. PDA Spectrum obtained at 254 nm.

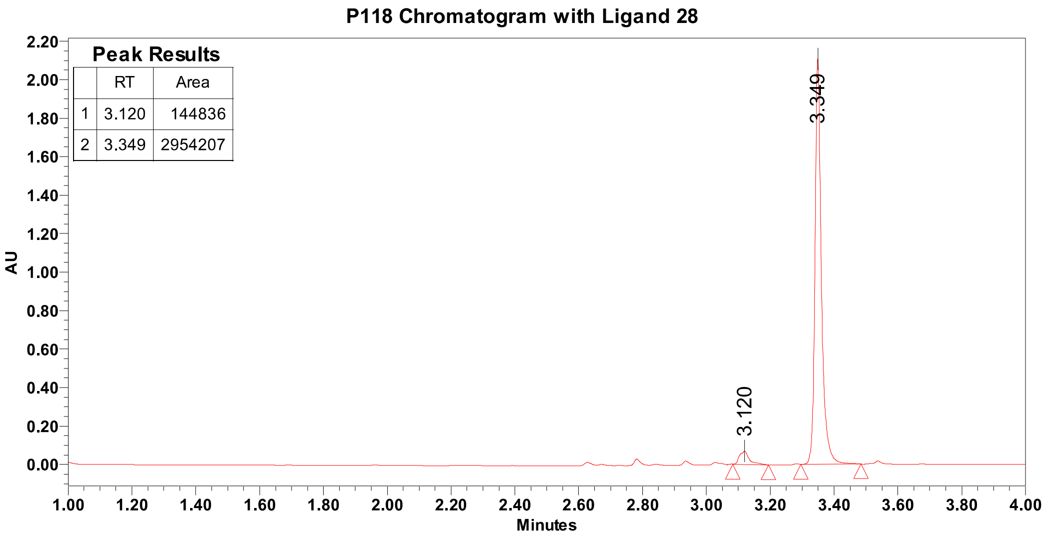

P119 Chromatogram with Ligand 36. Method: H_2O-MeCN 10% to 90%, 7 minutes. 1 µl injection on Acquity HSS Cyano 1.8µm 2.1mm x 100 mm column. PDA Spectrum obtained at 254 nm.

P119 Chromatogram with Ligand 29

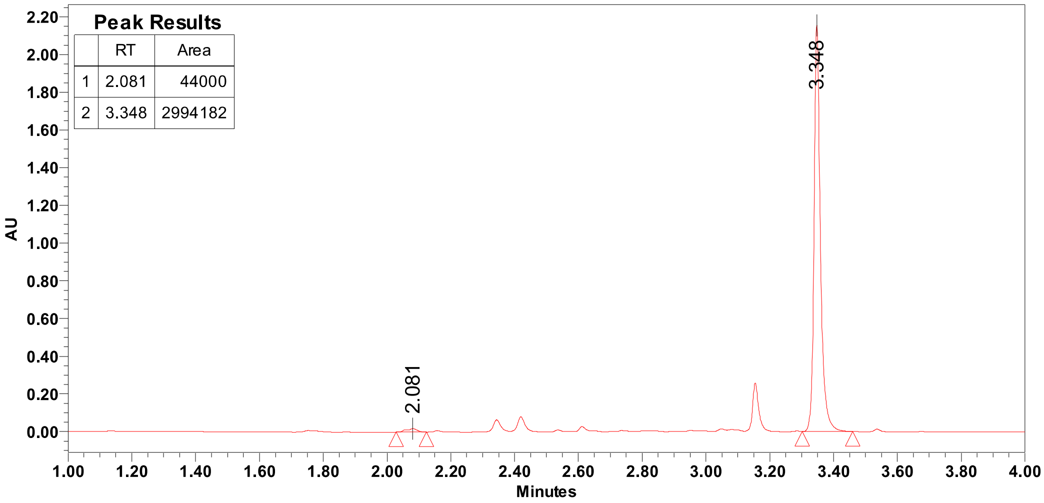

P120 Chromatogram with Ligand 36. Method: H_2O-MeCN 10% to 90%, 7 minutes. 1 µl injection on Acquity HSS Cyano 1.8µm 2.1mm x 100 mm column. PDA Spectrum obtained at 254 nm.

P120 Chromatogram with Ligand 33

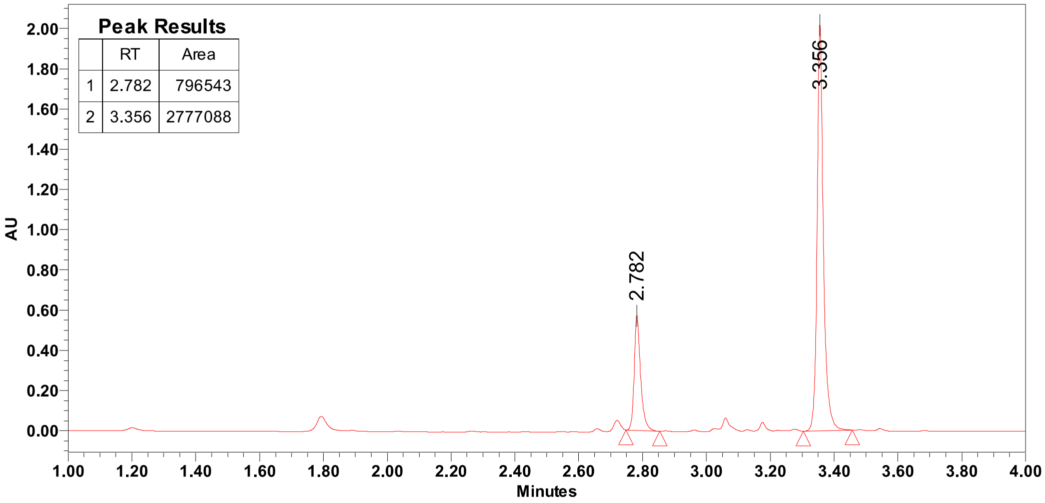

P121 Chromatogram with Ligand 36. Method: H_2O-MeCN 10% to 90%, 7 minutes. 1 µl injection on Acquity HSS Cyano 1.8µm 2.1mm x 100 mm column. PDA Spectrum obtained at 254 nm.

P121 Chromatogram with Ligand 30

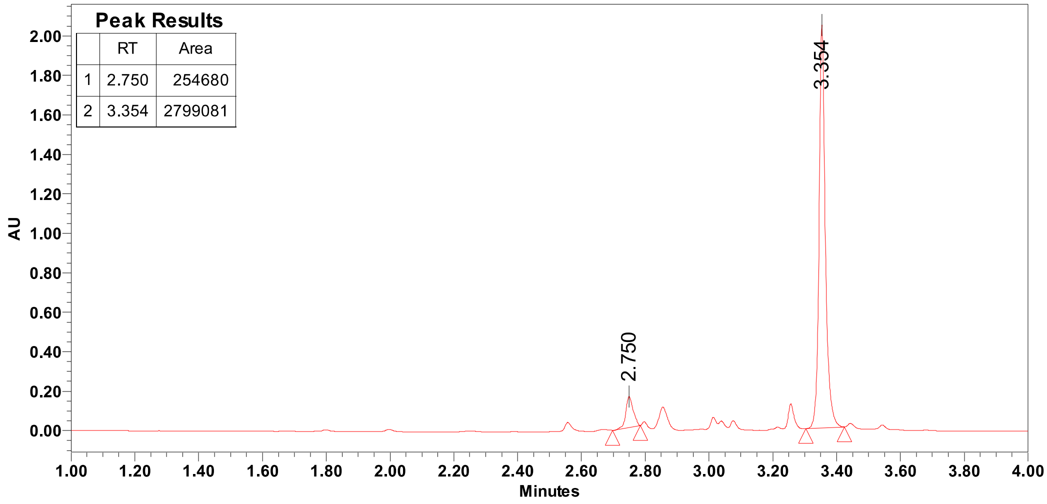

P122 Chromatogram with Ligand 36. Method: H_2O-MeCN 10% to 90%, 7 minutes. 1 µl injection on Acquity HSS Cyano 1.8µm 2.1mm x 100 mm column. PDA Spectrum obtained at 254 nm.

P122 Chromatogram with Ligand 30

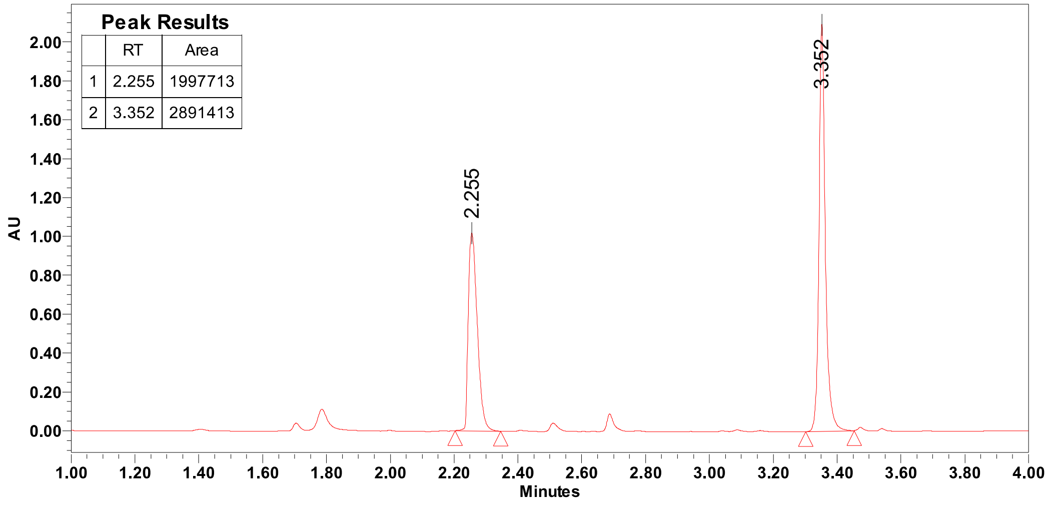

P123 Chromatogram with Ligand 36. Method: H_2O-MeCN 10% to 90%, 7 minutes. 1 µl injection on Acquity HSS Cyano 1.8µm 2.1mm x 100 mm column. PDA Spectrum obtained at 254 nm.

P123 Chromatogram with Ligand 26

P123 Chromatogram with Ligand 25

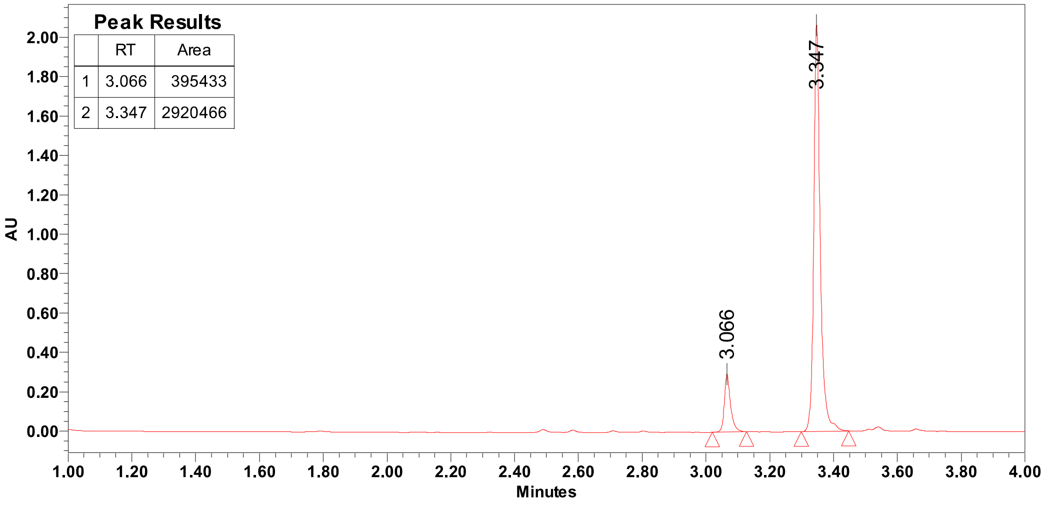

P124 Chromatogram with Ligand 36. Method: H_2O-MeCN 10% to 90%, 7 minutes. 1 µl injection on Acquity HSS Cyano 1.8µm 2.1mm x 100 mm column. PDA Spectrum obtained at 254 nm.

P124 Chromatogram with Ligand 28

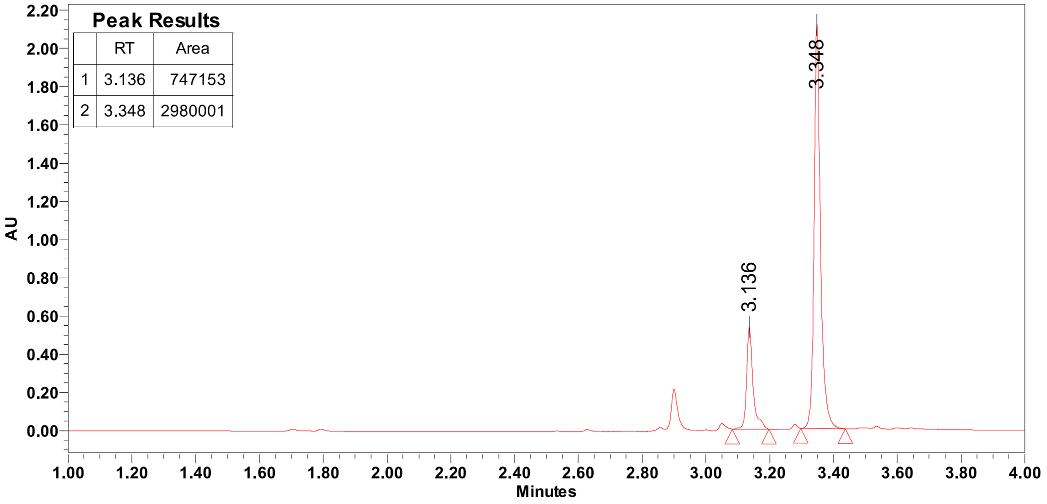

P125 Chromatogram with Ligand 36. Method: H_2O-MeCN 10% to 90%, 7 minutes. 1 µl injection on Acquity HSS Cyano 1.8µm 2.1mm x 100 mm column. PDA Spectrum obtained at 254 nm.

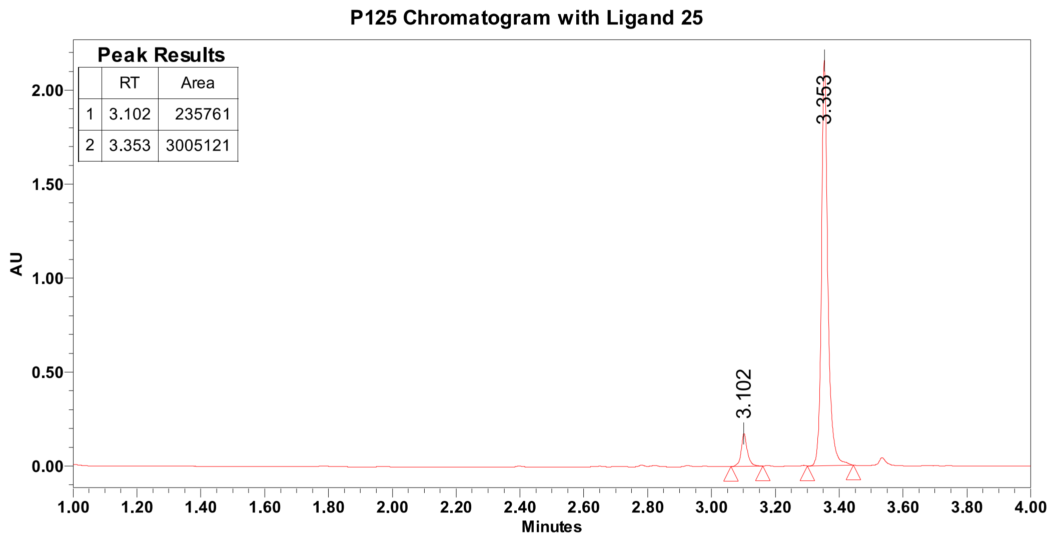

P126 Chromatogram with Ligand 36. Method: H_2O-MeCN 10% to 90%, 7 minutes. 1 µl injection on Acquity HSS Cyano 1.8µm 2.1mm x 100 mm column. PDA Spectrum obtained at 254 nm.

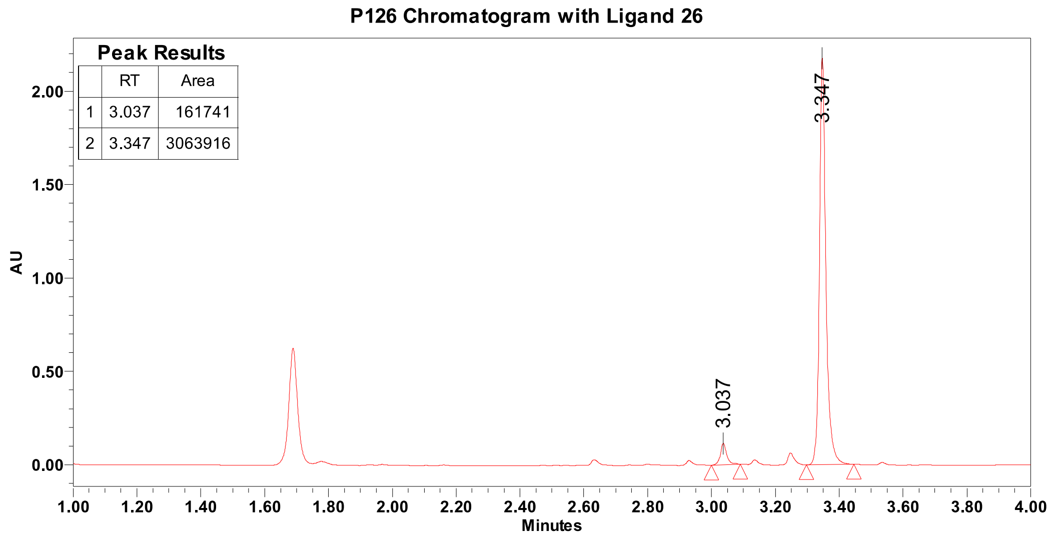

P127 Chromatogram with Ligand 36. Method: H_2O-MeCN 10% to 90%, 7 minutes. 1 µl injection on Acquity HSS Cyano 1.8µm 2.1mm x 100 mm column. PDA Spectrum obtained at 254 nm.

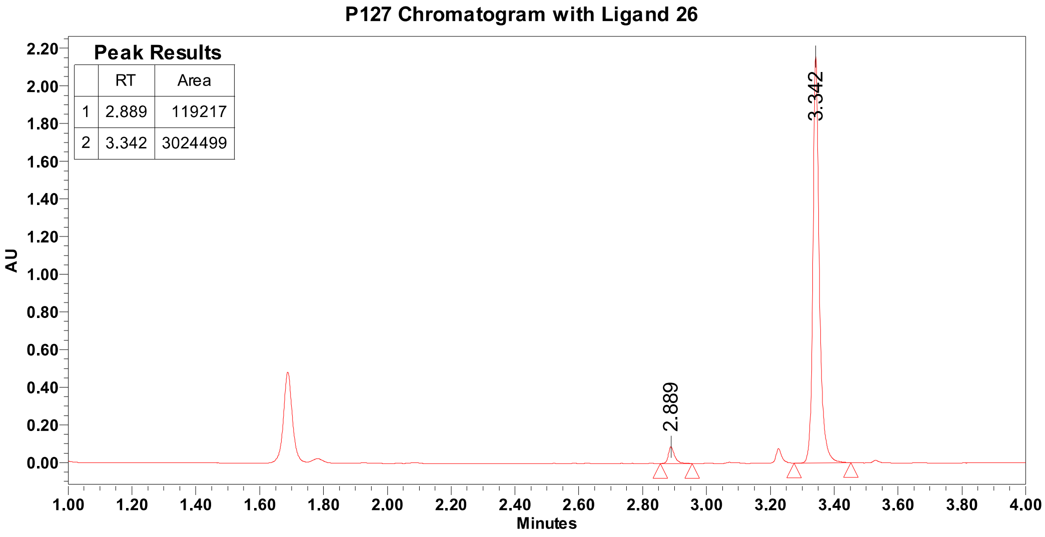

P200 Chromatogram with Ligand 36. Method: H_2O-MeCN 10% to 90%, 7 minutes. 1 µl injection on Acquity HSS Cyano 1.8µm 2.1mm x 100 mm column. PDA Spectrum obtained at 254 nm.

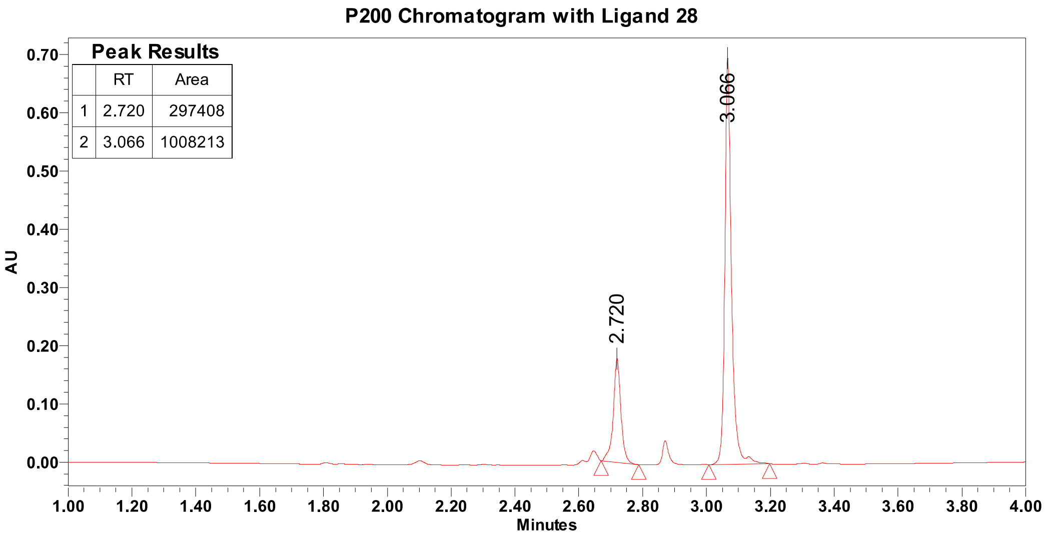

P201 Chromatogram with Ligand 36. Method: H_2O-MeCN 10% to 90%, 7 minutes. 1 µl injection on Acquity HSS Cyano 1.8µm 2.1mm x 100 mm column. PDA Spectrum obtained at 254 nm.

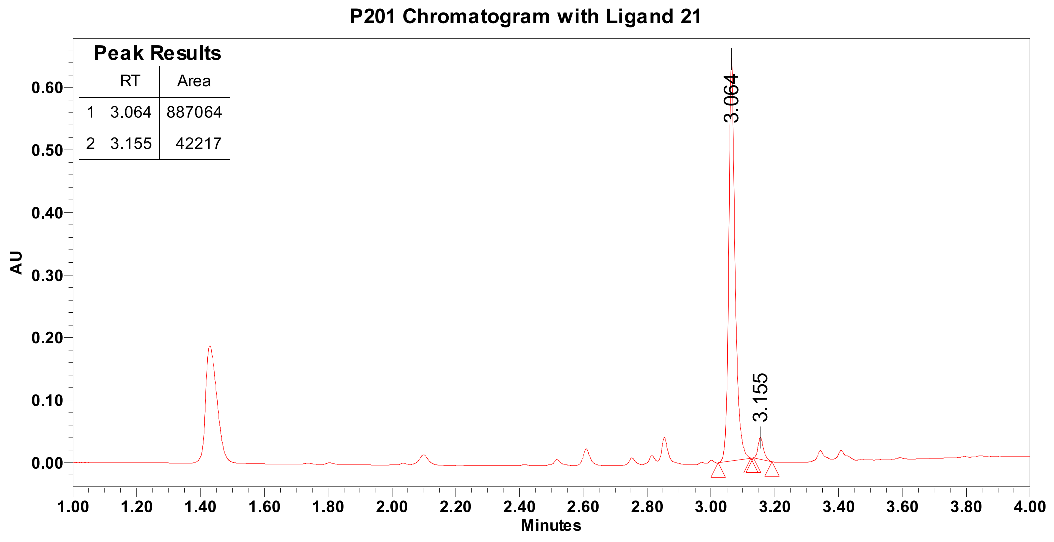

P202 Chromatogram with Ligand 36. Method: H_2O-MeCN 10% to 90%, 7 minutes. 1 µl injection on Acquity HSS Cyano 1.8µm 2.1mm x 100 mm column. PDA Spectrum obtained at 254 nm.

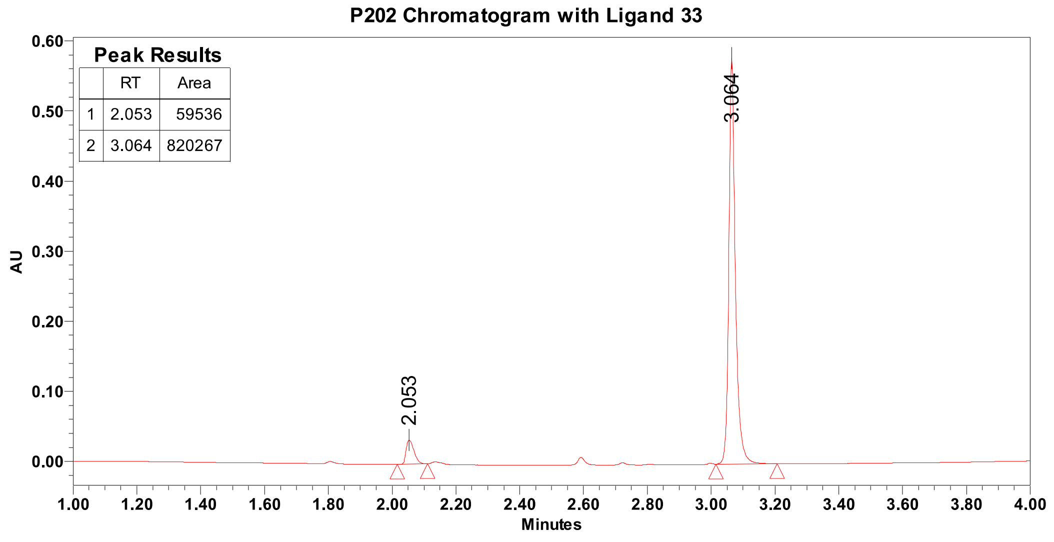

P203 Chromatogram with Ligand 36. Method: H_2O-MeCN 10% to 90%, 7 minutes. 1 µl injection on Acquity HSS Cyano 1.8µm 2.1mm x 100 mm column. PDA Spectrum obtained at 254 nm.

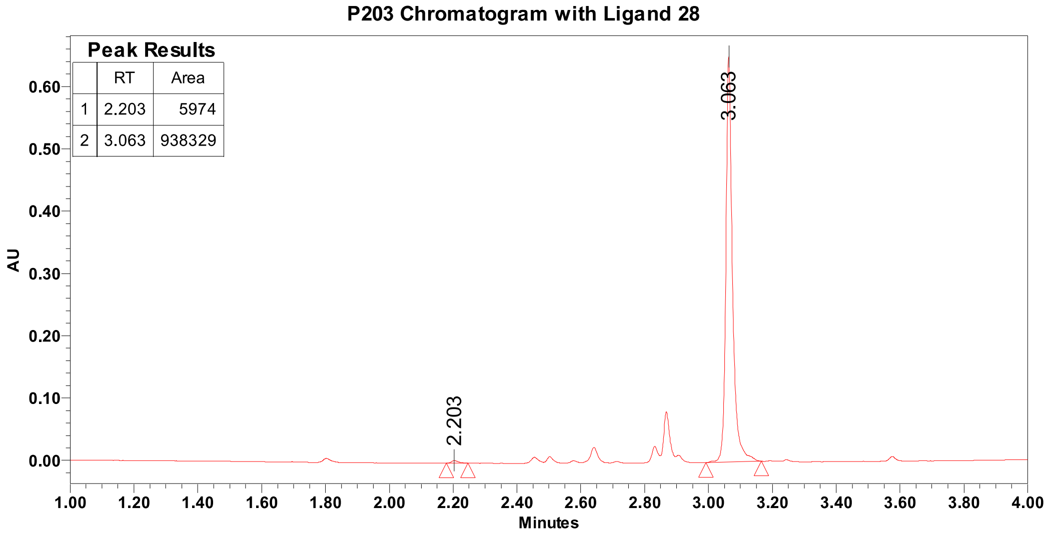

P204 Chromatogram with Ligand 36. Method: H_2O-MeCN 10% to 90%, 7 minutes. 1 µl injection on Acquity HSS Cyano 1.8µm 2.1mm x 100 mm column. PDA Spectrum obtained at 254 nm.

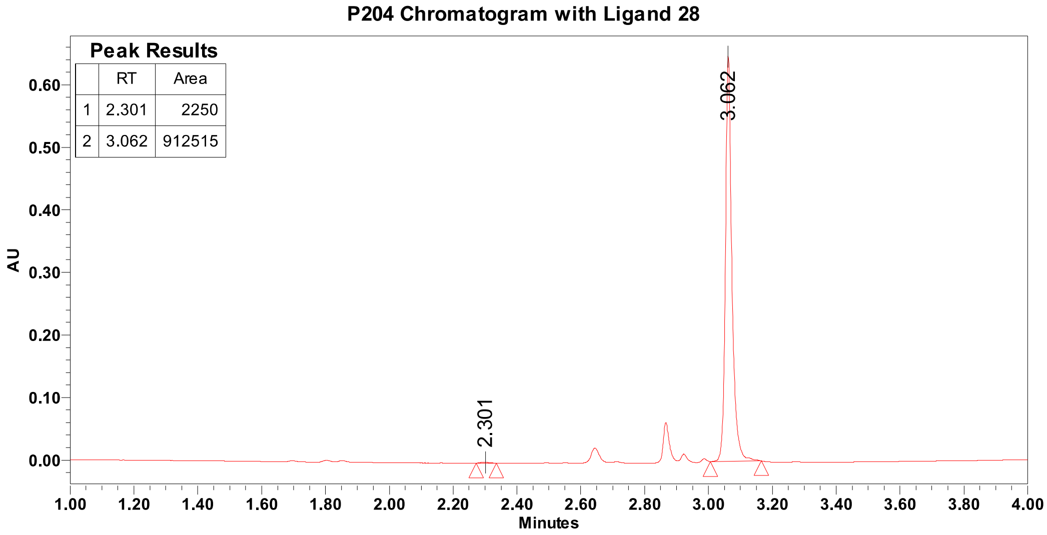

P205 Chromatogram with Ligand 36. Method: H_2O-MeCN 10% to 90%, 7 minutes. 1 µl injection on Acquity HSS Cyano 1.8µm 2.1mm x 100 mm column. PDA Spectrum obtained at 254 nm.

P205 Chromatogram with Ligand 21

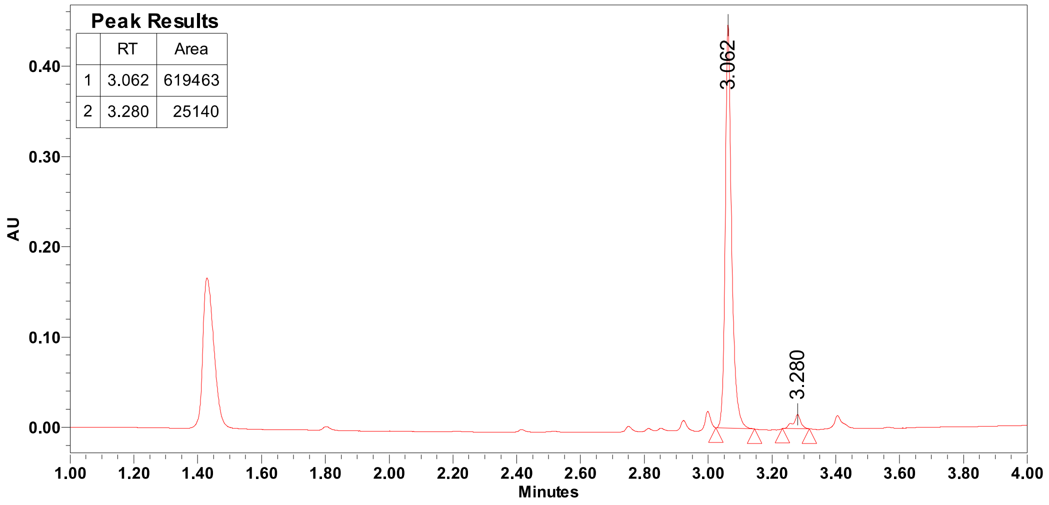

P206 Chromatogram with Ligand 36. Method: H_2O-MeCN 10% to 90%, 7 minutes. 1 µl injection on Acquity HSS Cyano 1.8µm 2.1mm x 100 mm column. PDA Spectrum obtained at 254 nm.

P206 Chromatogram with Ligand 28

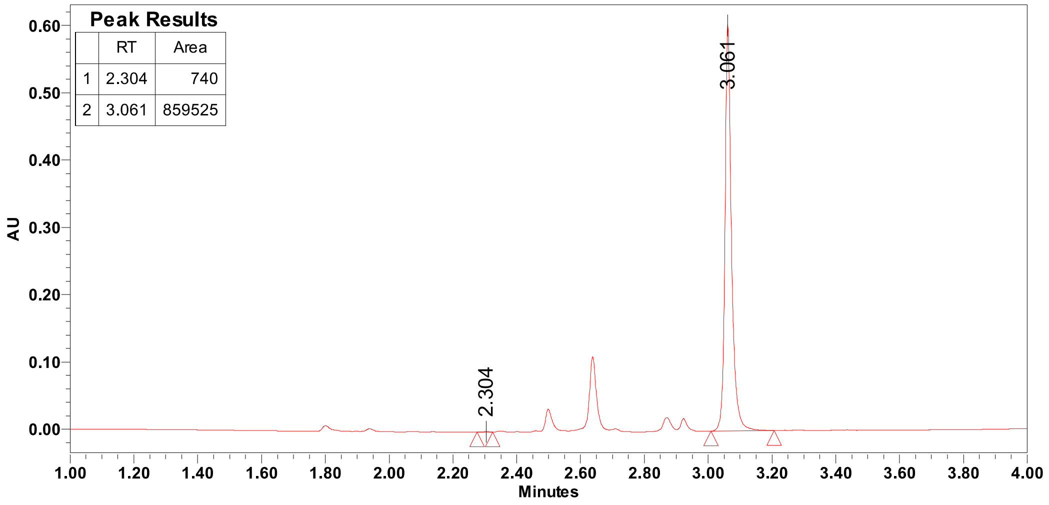

P207 Chromatogram with Ligand 36. Method: H_2O-MeCN 10% to 90%, 7 minutes. 1 µl injection on Acquity HSS Cyano 1.8µm 2.1mm x 100 mm column. PDA Spectrum obtained at 254 nm.

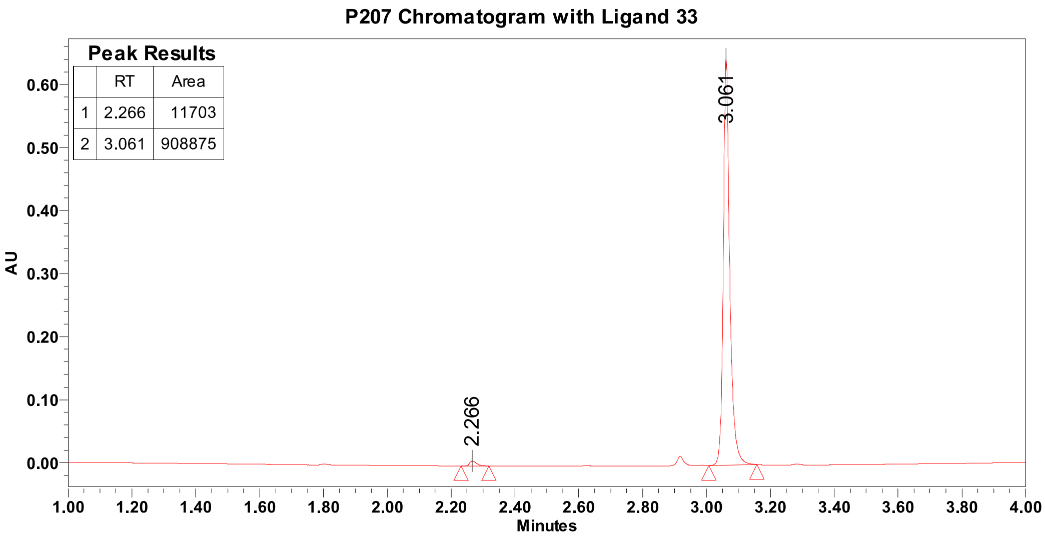

P208 Chromatogram with Ligand 36. Method: H_2O-MeCN 10% to 90%, 7 minutes. 1 µl injection on Acquity HSS Cyano 1.8µm 2.1mm x 100 mm column. PDA Spectrum obtained at 254 nm.

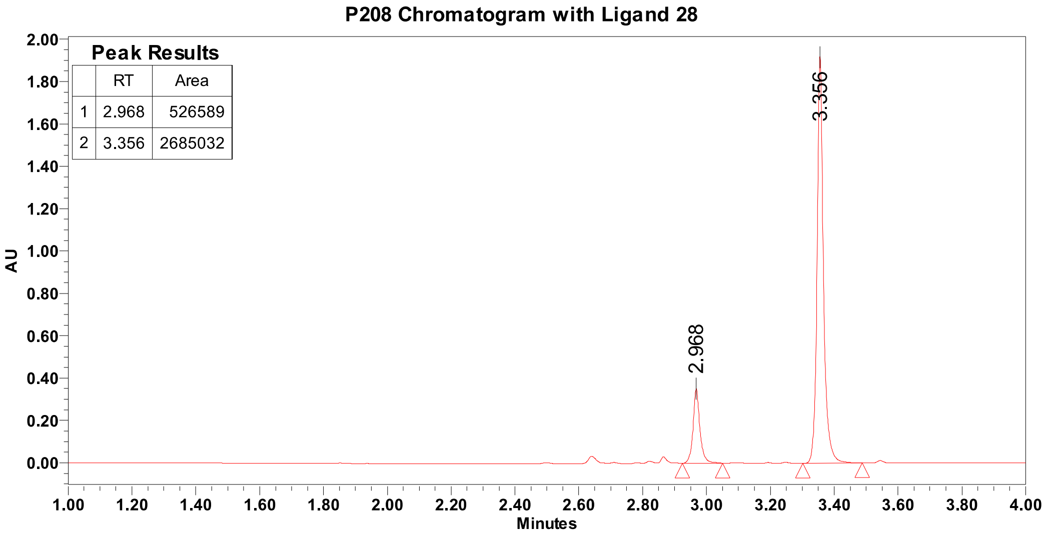

P209 Chromatogram with Ligand 36. Method: H_2O-MeCN 10% to 90%, 7 minutes. 1 µl injection on Acquity HSS Cyano 1.8µm 2.1mm x 100 mm column. PDA Spectrum obtained at 254 nm.

P209 Chromatogram with Ligand 28

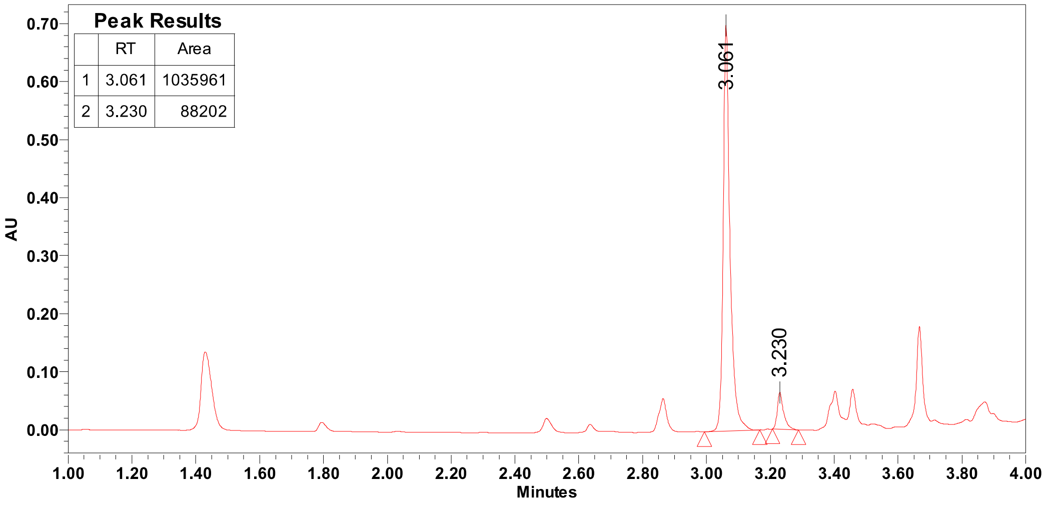

P210 Chromatogram with Ligand 36. Method: H_2O-MeCN 10% to 90%, 7 minutes. 1 µl injection on Acquity HSS Cyano 1.8µm 2.1mm x 100 mm column. PDA Spectrum obtained at 254 nm.

P210 Chromatogram with Ligand 33

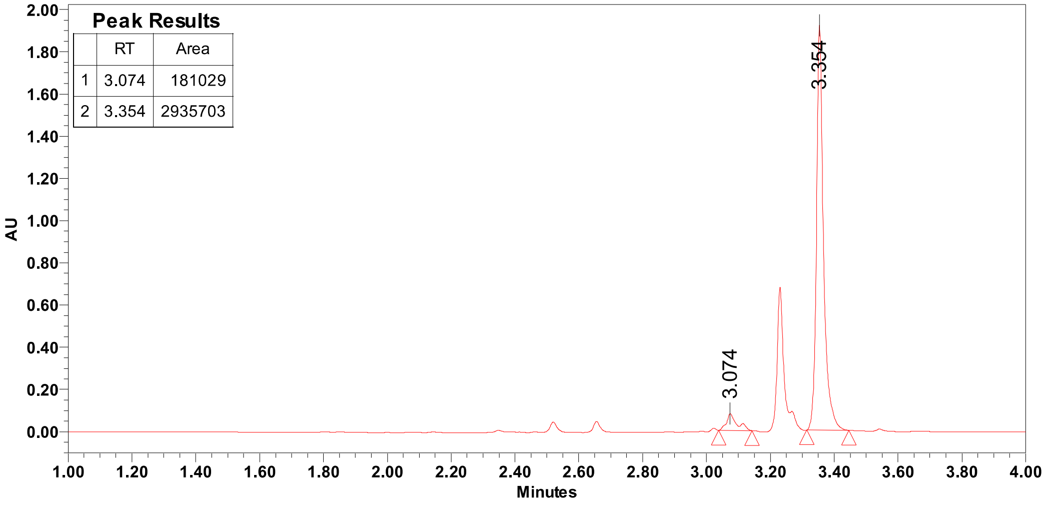

P211 Chromatogram with Ligand 36. Method: H_2O-MeCN 10% to 90%, 7 minutes. 1 µl injection on Acquity HSS Cyano 1.8µm 2.1mm x 100 mm column. PDA Spectrum obtained at 254 nm.

P211 Chromatogram with Ligand 28

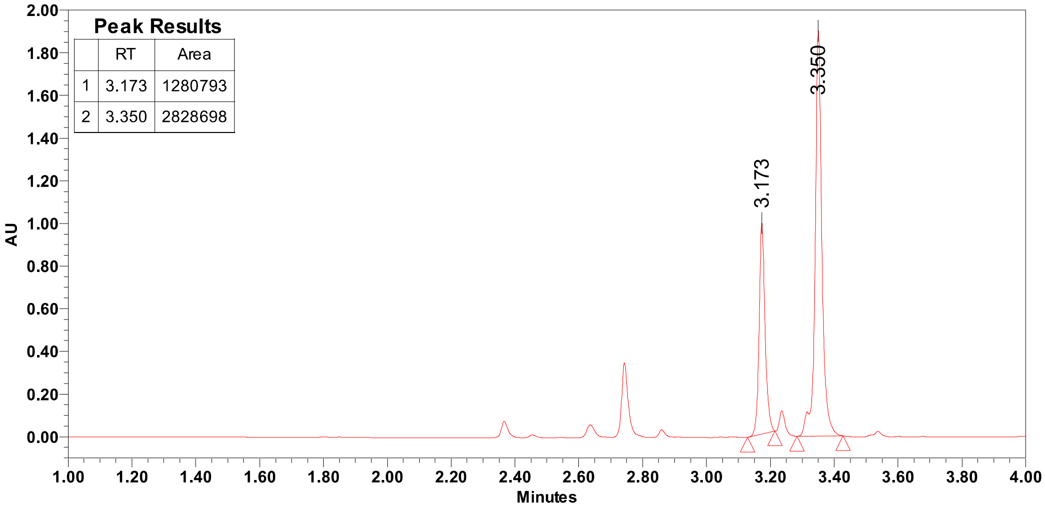

P212 Chromatogram with Ligand 36. Method: H_2O-MeCN 10% to 90%, 7 minutes. 1 µl injection on Acquity HSS Cyano 1.8µm 2.1mm x 100 mm column. PDA Spectrum obtained at 254 nm.

P212 Chromatogram with Ligand 33

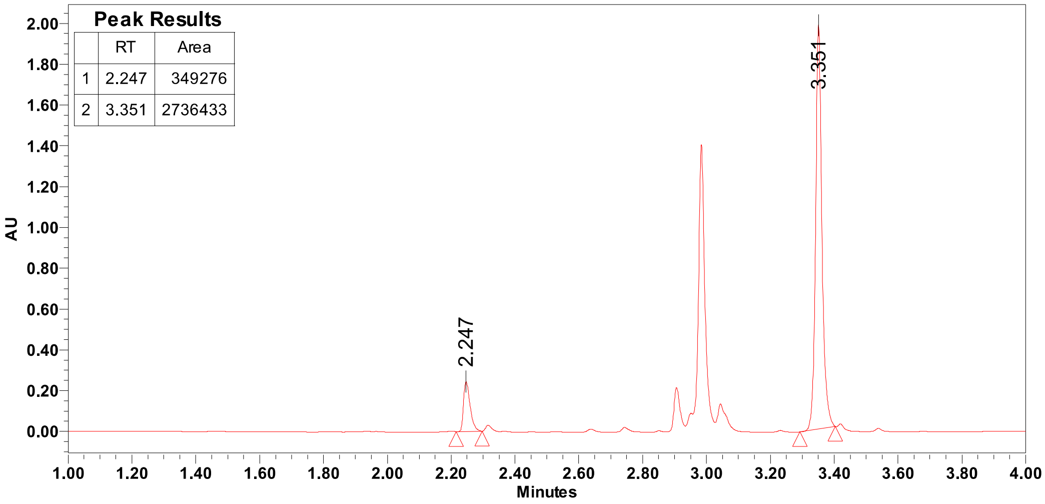

P213 Chromatogram with Ligand 36. Method: H_2O-MeCN 10% to 90%, 7 minutes. 1 µl injection on Acquity HSS Cyano 1.8µm 2.1mm x 100 mm column. PDA Spectrum obtained at 254 nm.

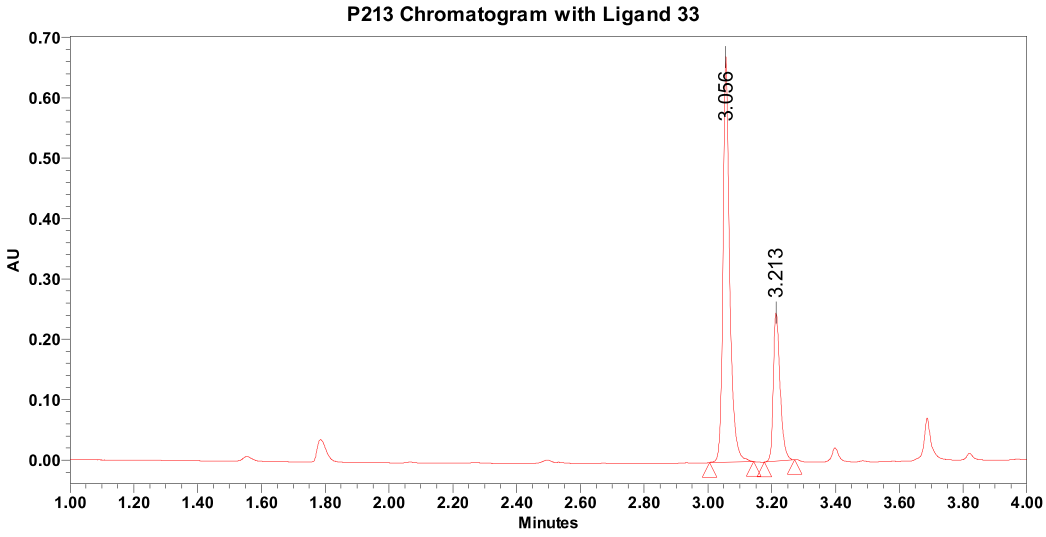

P214 Chromatogram with Ligand 36. Method: H_2O-MeCN 10% to 90%, 7 minutes. 1 µl injection on Acquity HSS Cyano 1.8µm 2.1mm x 100 mm column. PDA Spectrum obtained at 254 nm.

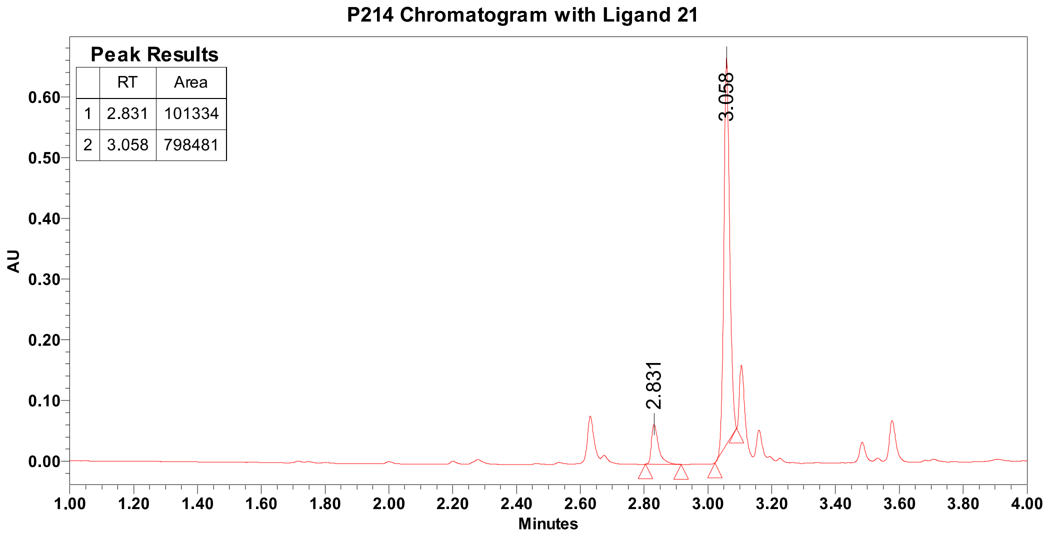

P215 Chromatogram with Ligand 36. Method: H_2O-MeCN 10% to 90%, 7 minutes. 1 µl injection on Acquity HSS Cyano 1.8µm 2.1mm x 100 mm column. PDA Spectrum obtained at 254 nm.

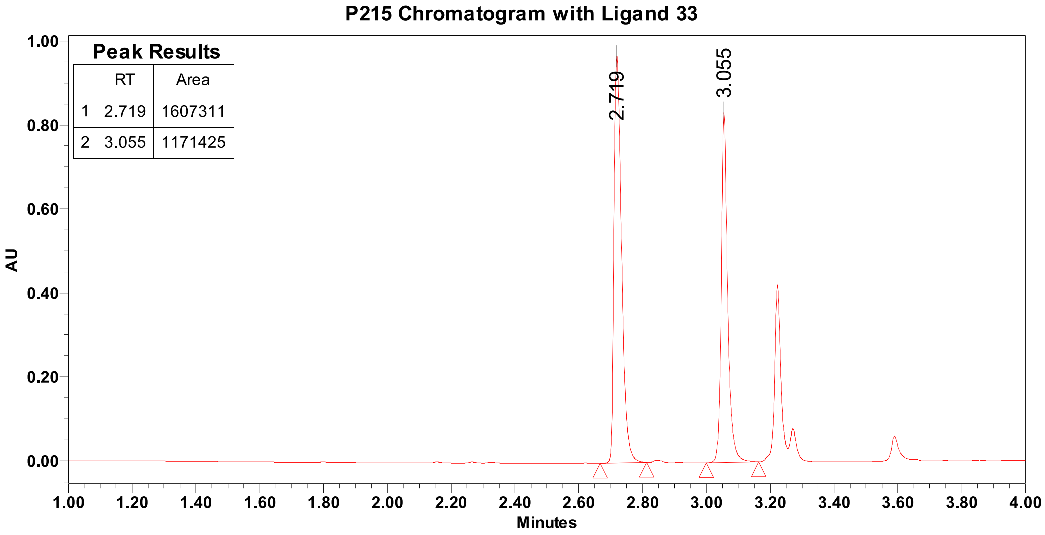

P216 Chromatogram with Ligand 36. Method: H_2O-MeCN 10% to 90%, 7 minutes. 1 µl injection on Acquity HSS Cyano 1.8µm 2.1mm x 100 mm column. PDA Spectrum obtained at 254 nm.

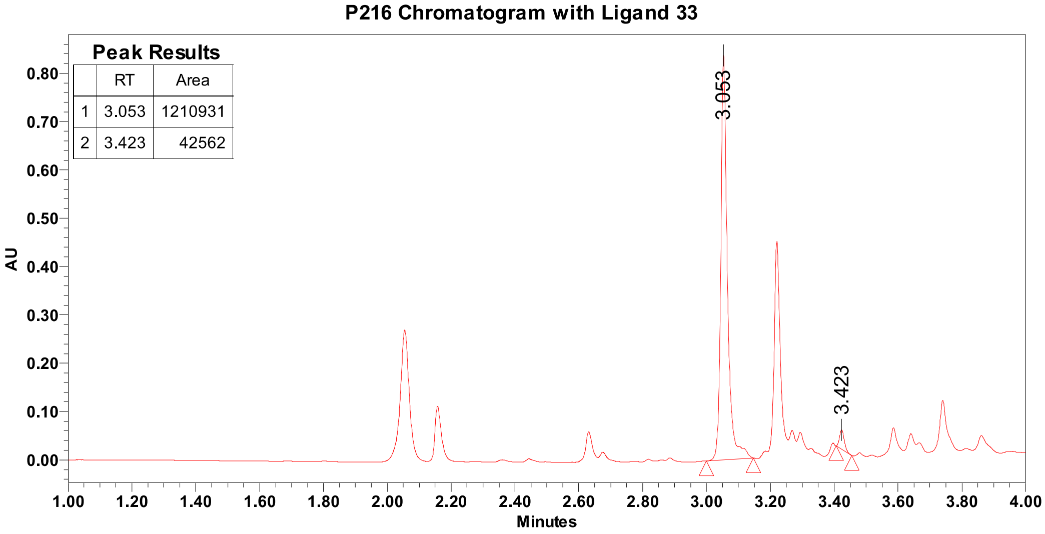

P217 Chromatogram with Ligand 36. Method: H_2O-MeCN 10% to 90%, 7 minutes. 1 µl injection on Acquity HSS Cyano 1.8µm 2.1mm x 100 mm column. PDA Spectrum obtained at 254 nm.

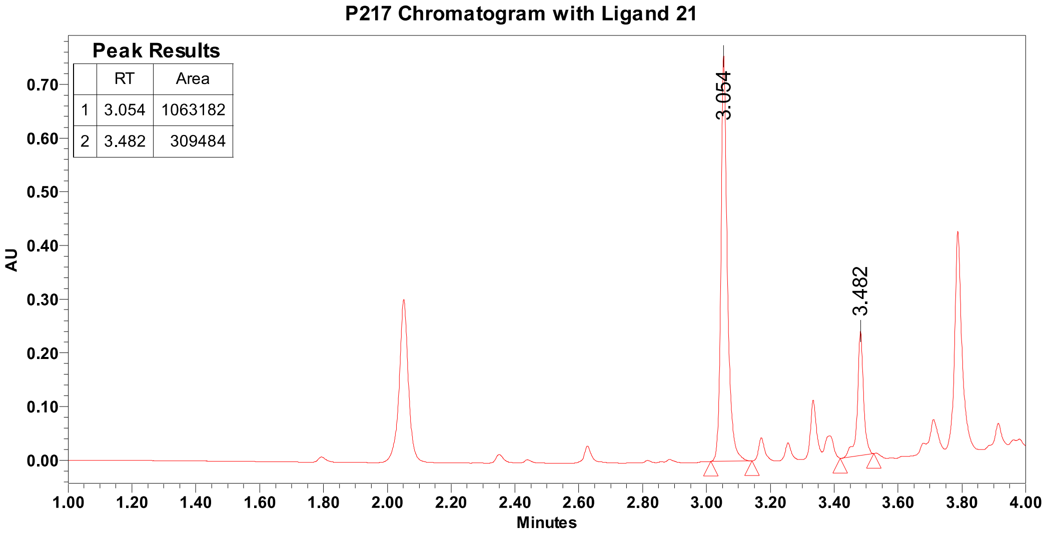

P218 Chromatogram with Ligand 36. Method: H_2O-MeCN 10% to 90%, 7 minutes. 1 µl injection on Acquity HSS Cyano 1.8µm 2.1mm x 100 mm column. PDA Spectrum obtained at 254 nm.

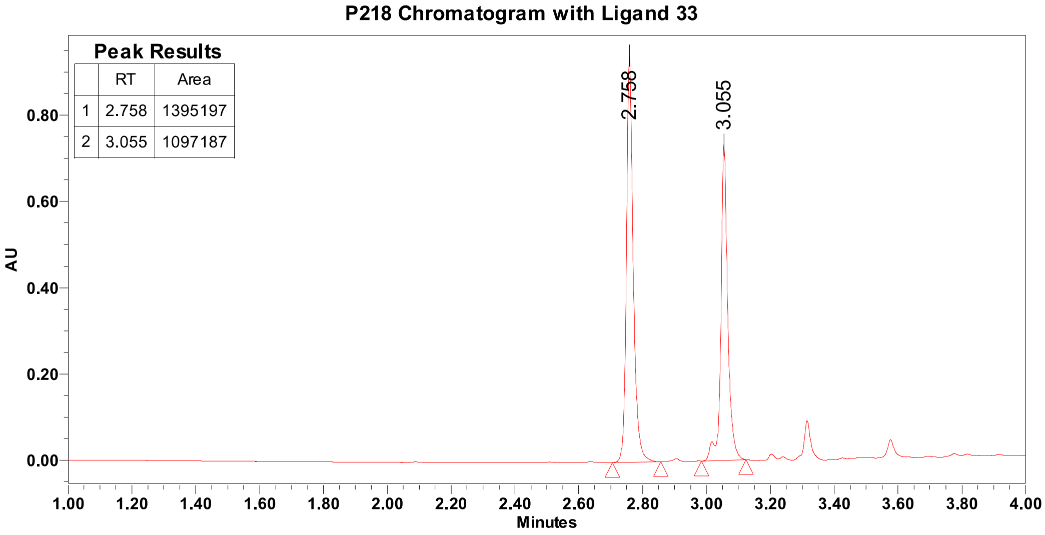

P219 Chromatogram with Ligand 36. Method: H_2O-MeCN 10% to 90%, 7 minutes. 1 µl injection on Acquity HSS Cyano 1.8µm 2.1mm x 100 mm column. PDA Spectrum obtained at 254 nm.

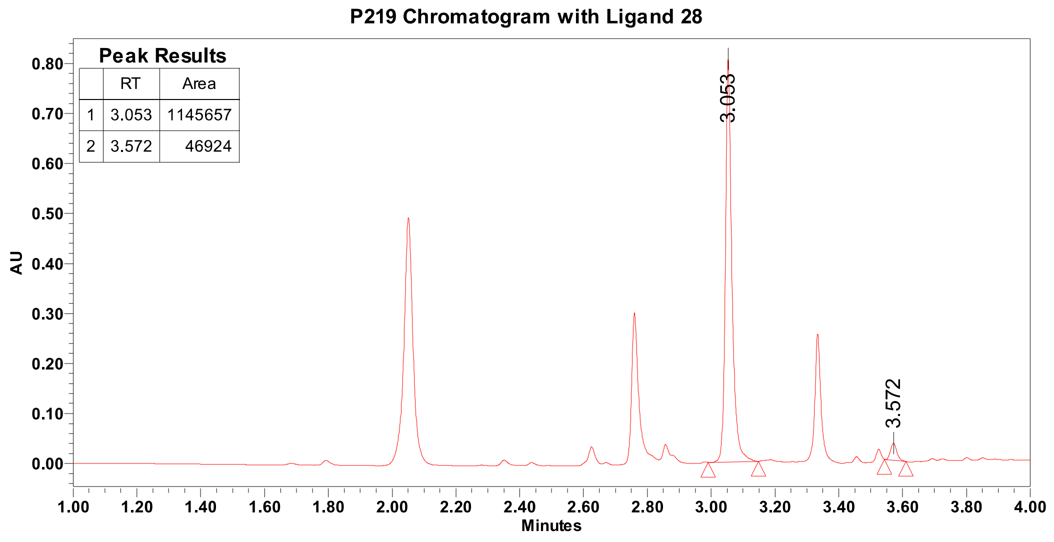

P300 Chromatogram with Ligand 36. Method: H_2O-MeCN 10% to 90%, 7 minutes. 1 µl injection on Acquity HSS Cyano 1.8µm 2.1mm x 100 mm column. PDA Spectrum obtained at 254 nm.

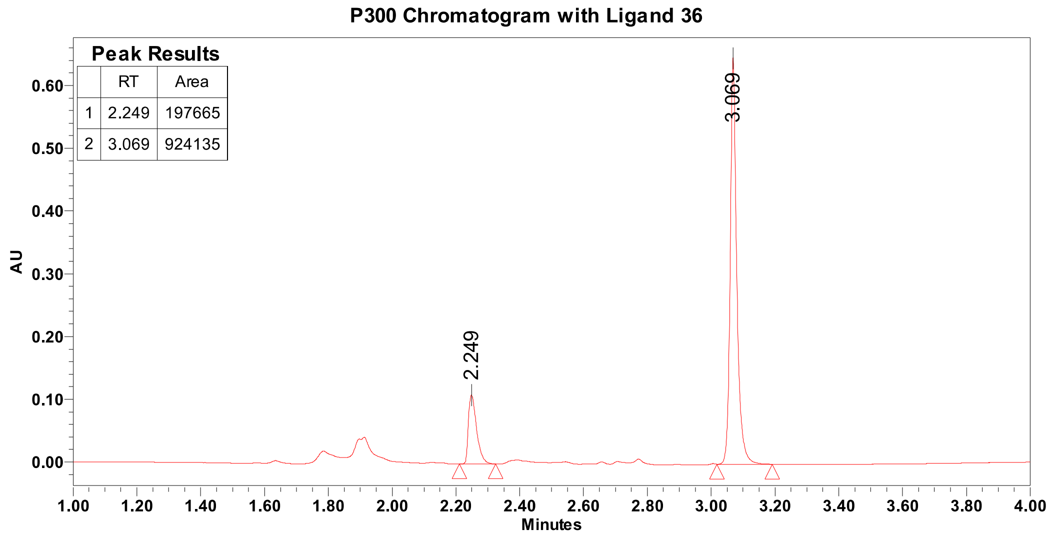

P301 Chromatogram with Ligand 36. Method: H_2O-MeCN 10% to 90%, 7 minutes. 1 µl injection on Acquity HSS Cyano 1.8µm 2.1mm x 100 mm column. PDA Spectrum obtained at 254 nm.

P301 Chromatogram with Ligand 27

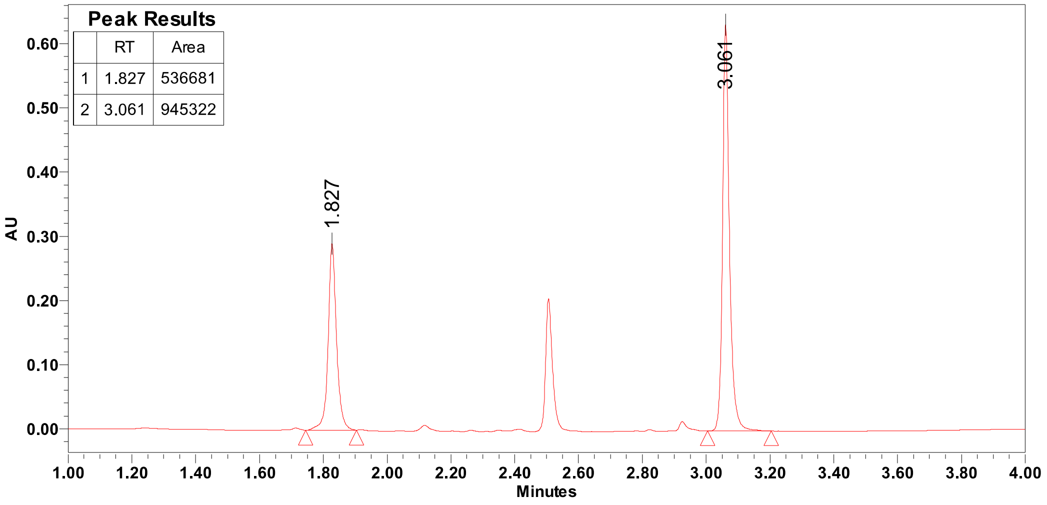

REFERENCES

1. K. D. Collins, T. Gensch, F. Glorius, Contemporary screening approaches to reaction discovery and development. *Nat. Chem.* **6**, 859–871 (2014).

2. A. B. Santanilla, E. L. Regalado, T. Pereira, M. Shevlin, K. Bateman, L. C. Campeau, J. Schneeweis, S. Berritt, Z. C. Shi, P. Nantermet, Y. Liu, R. Helmy, C. J. Welch, P. Vachal, I. W. Davies, T. Cernak, S. D. Dreher, Nanomole-scale high-throughput chemistry for the synthesis of complex molecules. *Science* **347**, 49–53 (2015).

3. D. T. Ahneman, J. G. Estrada, S. Lin, S. D. Dreher, A. G. Doyle, Predicting reaction performance in C–N cross-coupling using machine learning. *Science* **360**, 186–190 (2018).

4. N. I. Rinehart, R. K. Saunthwal, J. Wellauer, A. F. Zahrt, L. Schlemper, A. S. Shved, R. Bigler, S. Fantasia, S. E. Denmark, A machine-learning tool to predict substrate-adaptive conditions for Pd-catalyzed C–N couplings. *Science* **381**, 965–972 (2023).

5. M. S. Sigman, E. N. Jacobsen, Schiff base catalysts for the asymmetric Strecker reaction identified and optimized from parallel synthetic libraries. *J. Am. Chem. Soc.* **120**, 4901–4902 (1998).

6. P. Vachal, E. N. Jacobsen, Structure-based analysis and optimization of a highly enantioselective catalyst for the Strecker reaction. *J. Am. Chem. Soc.* **124**, 10012–10014 (2002).

7. B. J. Shields, J. Stevens, J. Li, M. Parasram, F. Damani, J. I. M. Alvarado, J. M. Janey, R. P. Adams, A. G. Doyle, Bayesian reaction optimization as a tool for chemical synthesis. *Nature* **590**, 89–96 (2021).

8. A. Milo, E. N. Bess, M. S. Sigman, Interrogating selectivity in catalysis using molecular vibrations. *Nature* **507**, 210–214 (2014).

9. E. N. Bess, A. J. Bischoff, M. S. Sigman, E. N. Jacobsen, Designer substrate library for quantitative, predictive modeling of reaction performance. *Proc. Natl. Acad. Sci. U.S.A.* **111**, 14698–14703 (2014).

10. A. Milo, A. J. Neel, F. D. Toste, M. S. Sigman, A data-intensive approach to mechanistic elucidation applied to chiral anion catalysis. *Science* **347**, 737–743 (2015).

11. C. D. Ritchie, W. F. Sager, An examination of structure-reactivity relationships. *Prog. Phys. Org. Chem.* **2**, 323–400 (1964).

12. M. W. Mullowney, K. R. Duncan, S. S. Elsayed, N. Garg, J. J. J. van der Hooft, N. I. Martin, D. Meijer, B. R. Terlouw, F. Biermann, K. Blin, J. Durairaj, M. Gorostiola González, E. J. N. Helfrich, F. Huber, S. Leopold-Messer, K. Rajan, T. de Rond, J. A. van Santen, M. Sorokina, M. J. Balunas, M. A. Beniddir, D. A. van Bergeijk, L. M. Carroll, C. M. Clark, D. A. Clevert, C. A. Dejong, C. Du, S. Ferrinho, F. Grisoni, A. Hofstetter, W. Jespers, O. V. Kalinina, S. A. Kautsar, H. Kim, T. F. Leao, J. Masschelein, E. R. Rees, R. Reher, D. Reker, P. Schwaller, M. Segler, M. A. Skinnider, A. S. Walker, E. L. Willighagen, B. Zdrazil, N. Ziemert, R. J. M. Goss, P. Guyomard, A. Volkamer, W. H. Gerwick, H. U. Kim, R. Müller, G. P. van Wezel, G. J. P. van Westen, A. K. H. Hirsch, R. G. Linington, S. L. Robinson, M. H. Medema, Artificial intelligence for natural product drug discovery. *Nat. Rev. Drug Discov.* **22**, 895–916 (2023).

13. M. Aldeghi, C. W. Coley, A focus on simulation and machine learning as complementary tools for chemical space navigation. *Chem. Sci.* **13**, 8221–8223 (2022).

14. F. Ullmann, J. Bielecki, Ueber synthesen in der biphenylreihe. *Chem. Ber.* **34**, 2174–2185 (1901).

15. I. Goldberg, Ueber phenylirungen bei gegenwart von kupfer als katalysator. *Ber. Dtsch. Chem. Ges.* **39**, 1691–1692 (1906).

16. J. Hassan, M. Sévignon, C. Gozzi, E. Schulz, M. Lemaire, Aryl-aryl bond formation one century after the discovery of the Ullmann reaction. *Chem. Rev.* **102**, 1359–1470 (2002).

17. E. R. Strieter, B. Bhayana, S. L. Buchwald, Mechanistic studies on the copper-catalyzed N-arylation of amides. *J. Am. Chem. Soc.* **131**, 78–88 (2009).

18. C. P. Delaney, E. Lin, Q. Huang, I. F. Yu, G. Rao, L. Tao, A. Jed, S. M. Fantasia, K. A. Püntener, R. D. Britt, J. F. Hartwig, Cross-coupling by a noncanonical mechanism involving the addition of aryl halide to Cu(II). *Science* **381**, 1079–1085 (2023).

19. Y. Luo, Y. Li, J. Wu, X. S. Xue, J. F. Hartwig, Q. Shen, Oxidative addition of an alkyl halide to form a stable Cu(III) product. *Science* **381**, 1072–1079 (2023).

20. Q. Yang, Y. Zhao, D. Ma, Cu-mediated Ullmann-type cross-coupling and industrial applications in route design, process development, and scale-up of pharmaceutical and agrochemical processes. *Org. Process Res. Dev.* **26**, 1690–1750 (2022).

21. S. K. Kariofillis, S. Jiang, A. M. Żurański, S. S. Gandhi, J. I. Martinez Alvarado, A. G. Doyle, Using data science to guide aryl bromide substrate scope analysis in a Ni/photoredox-catalyzed cross-coupling with acetals as alcohol-derived radical sources. *J. Am. Chem. Soc.* **144**, 1045–1055 (2022).

22. N. H. Angello, V. Rathore, W. Beker, A. Wołos, E. R. Jira, R. Roszak, T. C. Wu, C. M. Schroeder, A. Aspuru-Guzik, B. A. Grzybowski, M. D. Burke, Closed-loop optimization of general reaction conditions for heteroaryl Suzuki-Miyaura coupling. *Science* **378**, 399–405 (2022).

23. S. H. Newman-Stonebraker, S. R. Smith, E. Borowski, E. Peters, T. Gensch, H. C. Johnson, M. S. Sigman, A. G. Doyle, Univariate classification of phosphine ligation state and reactivity in cross-coupling catalysis. *Science* **374**, 301–308 (2021).

24. J. J. Irwin, K. G. Tang, J. Young, C. Dandarchuluun, B. R. Wong, M. Khurelbaatar, Y. S. Moroz, J. Mayfield, R. A. Sayle, ZINC20—A free ultralarge-scale chemical database for ligand discovery. *J. Chem. Inf. Model.* **60**, 6065–6073 (2020).

25. C. Sambiagio, S. P. Marsden, A. J. Blacker, P. C. McGowan, Copper catalysed Ullmann type chemistry: From mechanistic aspects to modern development. *Chem. Soc. Rev.* **43**, 3525–3550 (2014).

26. *Modern Nucleophilic Aromatic Substitution*, Terrier F., Ed. (Wiley-VCH, 2013).

27. J. P. Hughes, S. S. Rees, S. B. Kalindjian, K. L. Philpott, Principles of early drug discovery. *Br. J. Pharmacol.* **162**, 1239–1249 (2011).

28. S. T. Kim, M. J. Strauss, A. Cabré, S. L. Buchwald, Room-temperature Cu-catalyzed amination of aryl bromides enabled by DFT-guided ligand design. *J. Am. Chem. Soc.* **145**, 6966–6975 (2023).

29. B. Settles, “Active learning literature survey” (Tech. Rep. 1648, Univ. of Wisconsin-Madison, Madison, WI, 2009).

30. J. Rein, S. D. Rozema, O. C. Langner, S. B. Zacate, M. A. Hardy, J. C. Siu, B. Q. Mercado, M. S. Sigman, S. J. Miller, S. Lin, Generality-oriented optimization of enantioselective aminoxyl radical catalysis. *Science* **380**, 706–712 (2023).

31. E. Shim, A. Tewari, T. Cernak, P. M. Zimmerman, Machine learning strategies for reaction development: Toward the low-data limit. *J. Chem. Inf. Model.* **63**, 3659–3668 (2023).

32. A. Shafir, S. L. Buchwald, Highly selective room-temperature copper-catalyzed C–N coupling reactions. *J. Am. Chem. Soc.* **128**, 8742–8743 (2006).

33. R. Giri, A. Brusoe, K. Troshin, J. Y. Wang, M. Font, J. F. Hartwig, Mechanism of the Ullmann biaryl ether synthesis catalyzed by complexes of anionic ligands: Evidence for the reaction of iodoarenes with ligated anionic Cu^{I} intermediates. *J. Am. Chem. Soc.* **140**, 793–806 (2018).

34. Z. Chen, D. Ma, Cu/ *N*, *N′*–dibenzyloxalamide-catalyzed *N*-arylation of heteroanilines. *Org. Lett.* **21**, 6874–6878 (2019).

35. Y. Chen, S. Li, L. Xu, D. Ma, Cu/oxalic diamide-catalyzed coupling of terminal alkynes with aryl halides. *J. Org. Chem.* **88**, 3330–3334 (2023).

36. J. P. Reid, M. S. Sigman, Holistic prediction of enantioselectivity in asymmetric catalysis. *Nature* **571**, 343–348 (2019).

37. Y. Xie, C. Zhang, X. Hu, C. Zhang, S. P. Kelley, J. L. Atwood, J. Lin, Machine learning assisted synthesis of metal-organic nanocapsules. *J. Am. Chem. Soc.* **142**, 1475–1481 (2020).

38. J. J. Dotson, L. van Dijk, J. C. Timmerman, S. Grosslight, R. C. Walroth, F. Gosselin, K. Püntener, K. A. Mack, M. S. Sigman, Data-driven multi-objective optimization tactics for catalytic asymmetric reactions using bisphosphine ligands. *J. Am. Chem. Soc.* **145**, 110–121 (2023).

39. E. Heid, C. J. McGill, F. H. Vermeire, W. H. Green, Characterizing uncertainty in machine learning for chemistry. *J. Chem. Inf. Model.* **63**, 4012–4029 (2023).

40. C. E. Shannon, A mathematical theory of communication. *Bell Syst. Tech. J.* **27**, 379–423 (1948).

41. R. Franke, Scattered data interpolation: Tests of some methods. *Math. Comput.* **38**, 181–200 (1982).

42. A. M. Griffin, W. Brown, C. Walpole, M. Coupal, L. Adam, M. Gosselin, D. Salois, P. E. Morin, M. Roumi, Delta agonist hydroxy bioisosteres: The discovery of 3-((1-benzylpiperidin-4-yl){4-[(diethylamino)carbonyl]phenyl}amino)benzamide with improved delta agonist activity and in vitro metabolic stability. *Bioorg. Med. Chem. Lett.* **19**, 5999–6003 (2009).

43. P. Ruiz-Castillo, S. L. Buchwald, Applications of palladium-catalyzed C–N cross-coupling reactions. *Chem. Rev.* **116**, 12564–12649 (2016).

44. H. Jiang, S. Gao, J. Xu, X. Wu, A. Lin, H. Yao, Multiple roles of the pyrimidyl group in the rhodium-catalyzed regioselective synthesis and functionalization of indole-3-carboxylic acid esters. *Adv. Synth. Catal.* **358**, 188–194 (2016).

45. H. Shen, T. Liu, D. Cheng, X. Yi, Z. Wang, L. Liu, D. Song, F. Ling, W. Zhong, Ruthenium-catalyzed electrochemical synthesis of indolines through dehydrogenative [3 + 2] annulation with H_2 evolution. *J. Org. Chem.* **85**, 13735–13746 (2020).

46. S. K. Keshri, S. Madhavan, M. Kapur, Catalyst-controlled chemodivergent reactivity of vinyl cyclopropanes: A selective approach toward indoles and aniline derivatives. *Org. Lett.* **24**, 9043–9048 (2022).

47. Schrödinger Release 2023-2: Maestro, Schrödinger, LLC, New York, NY, 2023.

48. M. J. Frisch, G. W. Trucks, H. B. Schlegel, G. E. Scuseria, M. A. Robb, J. R. Cheeseman, G. Scalmani,V. Barone, G. A. Petersson, H. Nakatsuji, X. Li, M. Caricato, A. V. Marenich, J. Bloino, B. G. Janesko, R. Gomperts, B. Mennucci, H. P. Hratchian, J. V. Ortiz, A. F. Izmaylov, J. L. Sonnenberg, D. Williams-Young, F. Ding, F. Lipparini, F. Egidi, J. Goings, B. Peng, A. Petrone, T. Henderson, D. Ranasinghe, V. G. Zakrzewski, J. Gao, N. Rega, G. Zheng, W. Liang, M. Hada, M.

Ehara, K. Toyota, R. Fukuda, J. Hasegawa, M. Ishida, T. Nakajima, Y. Honda, O. Kitao, H. Nakai, T. Vreven, K. Throssell, J. A. Montgomery, Jr., J. E. Peralta, F. Ogliaro, M. J. Bearpark, J. J. Heyd, E. N. Brothers, K. N. Kudin, V. N. Staroverov, T. A. Keith, R. Kobayashi, J. Normand, K. Raghavachari, A. P. Rendell, J. C. Burant, S. S. Iyengar, J. Tomasi, M. Cossi, J. M. Millam, M. Klene, C. Adamo, R. Cammi, J. W. Ochterski, R. L. Martin, K. Morokuma, O. Farkas, J. B. Foresman, and D. J. Fox, *Gaussian 16, Revision C.01* (Gaussian, Inc., Wallingford, CT, 2016).

49. K. Jorner, MORFEUS. https://github.com/kjelljorner/morfeus.

50. G. Luchini, J. V Alegre-Requena, I. Funes-Ardoiz, R. S. Paton, GoodVibes: Automated thermochemistry for heterogeneous computational chemistry data. *F1000Research 2020* **9**, 291 (2020).

51. X. J. Yu, H. Y. He, L. Yang, H. Y. Fu, X. L. Zheng, H. Chen, R. X. Li, Hemilabile *N*-heterocyclic carbene (NHC)-nitrogen-phosphine mediated Ru (II)-catalyzed *N*-alkylation of aromatic amine with alcohol efficiently. *Catal. Commun.* **95**, 54–57 (2017).

52. E. P. K. Olsen, P. L. Arrechea, S. L. Buchwald, Mechanistic insight leads to a ligand which facilitates the palladium-catalyzed formation of 2-(hetero)arylaminooxazoles and 4-(hetero)arylaminothiazoles. *Angew. Chem. Int. Ed.* **56**, 10569–10572 (2017).

53. W. Chen, K. Chen, W. Chen, M. Liu, H. Wu, Well-designed *N*-heterocyclic carbene ligands for palladium-catalyzed denitrative C-N coupling of nitroarenes with amines. *ACS Catal.* **9**, 8110–8115 (2019).

54. Y. Zhao, B. Huang, C. Yang, Q. Chen, W. Xia, Sunlight-driven forging of amide/ester bonds from three independent components: An approach to carbamates. *Org. Lett.* **18**, 5572–5575 (2016).

55. K. I. Shimizu, N. Imaiida, K. Kon, S. M. A. Hakim Siddiki, A. Satsuma, Heterogeneous Ni catalysts for N-alkylation of amines with alcohols. *ACS Catal.* **3**, 998–1005 (2013).
